# Space filling shapes the interaction networks in mixed pyrrole-benzene trimers and tetramers

**DOI:** 10.1038/s42004-026-02027-1

**Published:** 2026-04-17

**Authors:** Simon Lobsiger, Zbigniew Kisiel, Caroline S. Glick, George C. Shields, Brooks H. Pate, Cristóbal Pérez

**Affiliations:** 1 Department of Chemistry, University of Virginia, Charlottesville, VA USA; 2 Federal Institute of Metrology METAS, Bern-Wabern, Switzerland; 3 Institute of Physics, Polish Academy of Sciences, Warszawa, Poland; 4 Department of Chemistry, Furman University, Greenville, SC USA; 5 Departamento de Química Física y Química Inorgánica, Facultad de Ciencias-I.U. CINQUIMA, Universidad de Valladolid, Valladolid, Spain; 6 Centro Singular de Investigación en Química Biolóxica e Materiais Moleculares (CiQUS) and Departamento de Química Física, Universidade de Santiago de Compostela, Santiago de Compostela, Spain

**Keywords:** Chemical physics, Self-assembly

## Abstract

The structural preferences of molecular assemblies are governed by a delicate balance between strong directional forces and diffuse dispersion contacts. Mixed trimers of pyrrole (Py) and benzene (Bz) provide an ideal benchmark to probe this interplay: the robust N-H⋯*π* interaction anchoring the Py-Bz dimer competes with the drive toward compact, dispersion-stabilized arrangements in larger clusters. Here, we report the first high-resolution structural characterization of the Py-(Bz)_2_ and (Py)_2_-Bz trimers and the (Py)_2_-(Bz)_2_ tetramer using chirped-pulse Fourier transform microwave spectroscopy combined with dispersion-corrected DFT calculations and intensity-based cross-correlation analysis. The results show that while N-H⋯*π* and C-H⋯*π* interactions serve as primary anchors, the overall geometries are dictated by space-filling principles that maximize dispersion contacts. These findings establish pyrrole-benzene hetero clusters as a rigorous benchmark for theoretical methods and provide fundamental insight into the forces guiding aromatic aggregation and self-assembly in complex molecular systems.

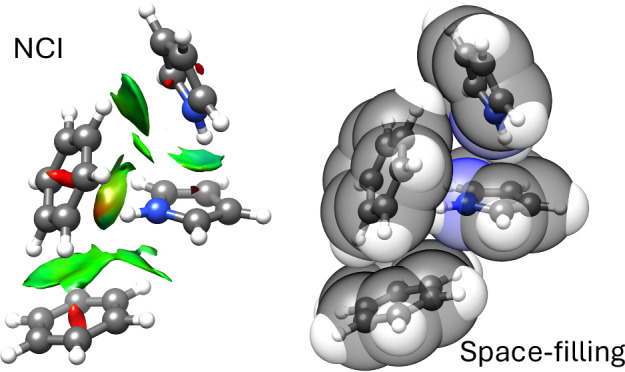

## Abstract (web summary)

Mixed trimers of pyrrole (Py) and benzene (Bz) provide an ideal benchmark to probe the interplay between strong directional forces and diffuse dispersion contacts that governs structural preferences in molecular assemblies. Here, the authors characterize Py-(Bz)_2_ and (Py)_2_-Bz trimers and the (Py)_2_-(Bz)_2_ tetramer using chirped-pulse Fourier transform microwave spectroscopy combined with dispersion-corrected DFT calculations and intensity-based cross-correlation analysis, showing that the overall geometries are dictated by space-filling principles that maximize dispersion contacts.

## Introduction

Noncovalent interactions are the subtle forces that organize matter across fields, from protein folding and molecular recognition in biology to the design of advanced supramolecular materials^1–3^. Aromatic ring interactions are especially important within this framework. The balance between directional forces such as hydrogen bonding and more diffuse *π*-*π* and London dispersion interactions creates a delicate equilibrium that dictates the geometry of supramolecular assemblies^4–7^. While a strong hydrogen bond can serve as a structural anchor, the collective effect of many weak dispersion contacts often drives molecules toward compact, space-filling arrangements.

Capturing this balance remains a challenge for both theory and experiment. Early implementations of density functional theory (DFT) underestimated dispersion forces; however, modern dispersion-corrected approaches, such as the DFT-D family, now provide accurate descriptions of weakly bound systems^8–10^. Benchmarking these methods requires experimental data that reveal the intrinsic structures of molecular clusters of increasing size in the absence of perturbing environments.

Gas-phase rotational spectroscopy, particularly chirped-pulse Fourier transform microwave (CP-FTMW) spectroscopy, has emerged as a uniquely powerful probe of such structures. Its broadband character enables simultaneous observation of thousands of rotational transitions, while its resolution and sensitivity provide structural accuracy on the order of picometers^11–13^. For larger systems, however, the spectra rapidly become congested due to overlapping signatures of multiple isomers, conformers, isotopologues, and cluster compositions^14^. Despite this complexity, several studies have successfully targeted *π*-stacked complexes^15–17^, and more recently, clusters involving water molecules^18–20^.

To address this spectral congestion, advanced techniques including two-dimensional CP-FTMW spectroscopy^21^, microwave spectral taxonomy^22^, and double-resonance methods^23,24^ have been developed to disentangle overlapping signals. Automated approaches for spectral analysis provide further support^25–27^, although these rely primarily on transition frequencies. A complementary strategy based on cross-correlation of spectral intensities across measurements has shown remarkable potential for filtering rotational spectra by size or composition, adding a new dimension of analysis for complex mixtures^28–31^.

Mixed trimers of pyrrole (Py) and benzene (Bz) provide an excellent system to probe the interplay between strong directional interactions and more dispersive, space-filling forces that are less accessible in the smaller dimers. Homo dimers and trimers of pyrrole and benzene have been investigated by microwave, FTIR, and double-resonance spectroscopies^32–36^, and hetero complexes such as Py-Bz and (Py)_2_-Bz have been characterized in supersonic-jet IR/UV experiments^37^, but no high-resolution rotational studies of the pyrrole-benzene hetero trimers have yet been reported.

Here, we present the first high-resolution structural characterization of the Py-(Bz)_2_ and (Py)_2_-Bz trimers and the (Py)_2_-(Bz)_2_ tetramer. By combining CP-FTMW spectroscopy with dispersion-corrected DFT calculations and cross-correlation techniques for spectral analysis, we demonstrate that while the N-H⋯*π* and C-H⋯*π* bonds act as primary anchors, the global minimum structures are ultimately governed by space-filling principles that maximize dispersion contacts. Our results provide a rigorous, high-resolution benchmark for understanding the hierarchy of interactions in complex aromatic assemblies, showing that even in the presence of strong, directional forces, the drive for optimal space-filling is the decisive structure-directing factor.

## Results and discussion

The broadband rotational spectra of the mixed Py-Bz complexes were recorded in the 2-8 GHz frequency range using a chirped-pulse Fourier transform microwave (CP-FTMW) spectrometer at the University of Virginia, as described previously^11,12^. The complexes were generated by co-expanding Bz in neon (backing pressure: 0.7 bar) over a heated reservoir (40 ^∘^C) containing Py, followed by pulsed supersonic expansion into the vacuum chamber using Parker Series 9 solenoid valves. To probe both the parent complexes and their heavy-atom (^13^C, ^15^N) isotopologues, two 10-million-shot free-induction decay (FID) averaged spectra were acquired with benzene-neon mixtures of 1.2% and 0.4%, respectively. Similarly, rotational spectra (10 million averaged FIDs) of mono-deuterated isotopologues were obtained using a 0.5% benzene-*d*_0_ and 0.5% benzene-*d*_1_ mixture in neon.

**Fig. 1:**
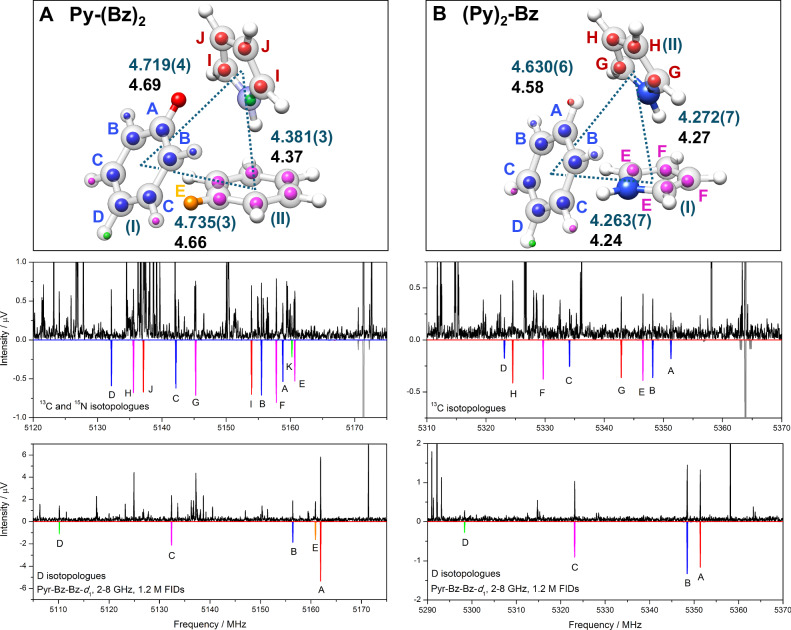
Selected regions of the broadband microwave spectrum of Py-(Bz)_2_ and (Py)_2_-Bz with assignments and corresponding structures. Selected regions of the broadband microwave spectrum of Py-(Bz)_2_ (**A**) and (Py)_2_-Bz (**B**), respectively. The top spectral panels show the assignment of the corresponding mono-substituted ^13^C or ^15^N in natural abundance, while the bottom spectral panels display the mono-deuterated isotopologues (enriched sample). The spectra are labeled and color-coded according to the labeling in the corresponding structures, referring to both the heavy atom and the position of the substituted hydrogen. Each ring and its isotopologues are colored in blue, magenta, and red, respectively. The positions corresponding to the A and E hydrogen atoms of the Py-(Bz)_2_ complex are not shown, as the positions from the Kraitchman analysis are unreliable due to their location in the inertial plane. The simulations are based on fitted rotational parameters in Table 1, considering a rotational temperature of 1.0 K. The faded, whole structures are the results of quantum-chemical calculations at the DLPNO-CCSD(T)/haug-cc-pVQZ//*ω*B97X-D/6-31++G** level of theory. The colored, solid spheres represent the experimentally derived atom positions using the Kraitchman method (see text). The distances (in Angstrom) between rings are determined from the preferred least-squares fits to all available rotational constants (dark blue) and from theory (black).

The co-expansion of Py and Bz produces a diverse set of polar complexes, many of which contribute to the observed broadband spectra. Intense spectral features corresponding to the Py monomer and dimer^32^, both reported previously, were readily identified. Another dominant spectrum was assigned to the mixed Py-Bz dimer, though it will not be discussed further here. In total, the two normal species observed exhibit exceptionally rich spectra, with more than 9000 transitions exceeding a 3:1 signal-to-noise threshold. Representative spectral regions are shown in Fig. 1. To address the challenges posed by such densely populated spectra, we employed a combined approach of advanced quantum-chemical sampling procedures and cross-correlation techniques, as outlined below.

The determination of the most stable structures of mixed Py-Bz trimers and tetramers presents a significant challenge, as it requires both a computationally efficient yet sufficiently accurate description of the potential energy surface (PES) and an effective global optimization strategy capable of exploring this complex landscape. The weak intermolecular interactions and the prevalence of numerous shallow local minima also complicate this task. In this work, we address these challenges by employing a funnel method consisting of three steps. In step one, a genetic algorithm (GA)^38^ was applied to exhaustively explore the GFN2-XTB and PM7 semi-empirical PESs, utilizing the OGOLEM package^39^ in conjunction with both PM7^40^ and the GFN2 implementation of the XTB method^41,42^. The many hundreds of low-energy isomers identified in this step were further optimized using Gaussian 16, Revision C.01, with the *ω*B97X-D density functional and the 6-31++G** basis set, a level of theory that has been shown to perform well in geometry optimization benchmarks of various organic clusters.^43–46^. This method is especially practical due to its double-zeta basis set, which allows for the efficient optimization of thousands of candidate structures. Because large energy errors are not uncommon with DFT,^43–45,47,48^, all isomers within 8 kcal mol^−1^ of the *ω*B97X-D/6-31++G** global minimum were subsequently refined by electronic energy corrections at the DLPNO-CCSD(T) level of theory, as implemented in ORCA^49,50^, with single, double, and perturbative triple excitations. The DLPNO-CCSD(T)/haug-cc-pVQZ approach, using Dunning’s quadruple-*ζ* basis sets (augmented for oxygen atoms but not for hydrogens, denoted as haug), was employed^46^. We thoroughly explored the PES for the mixed (Py)-(Bz)_2_ and (Py)_2_-(Bz) trimers and the (Py)_2_-(Bz)_2_, (Py)-(Bz)_3_, and (Py)_3_-(Bz) tetramers, as well as performed population calculations. Notably, the global minima of (Py)-(Bz)_2_, (Py)_2_-(Bz), and (Py)_2_-(Bz)_2_ show high Boltzmann populations at 100 K between 75 and 100%, which makes then the most likely clusters to be detected experimentally. All these results are reported in detail in the Supporting Information in section 4 (Theoretical calculations).

**Fig. 2:**
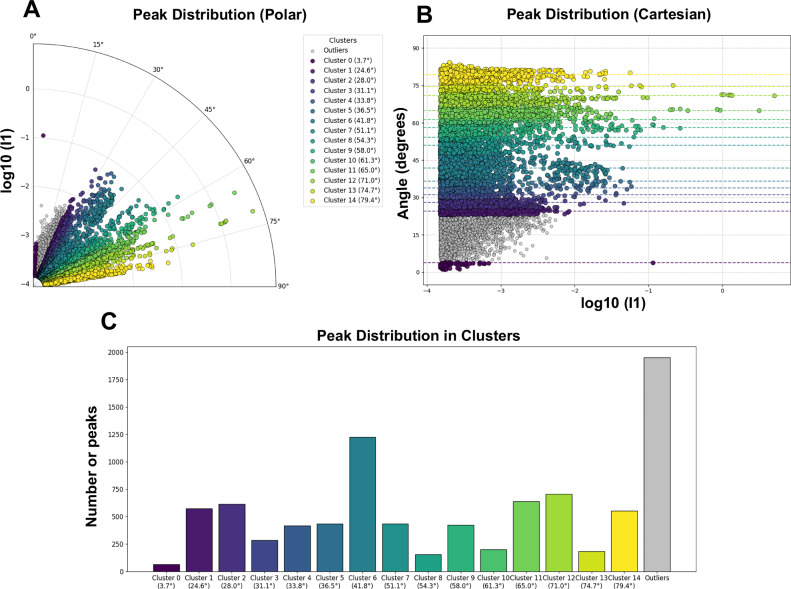
Intensity analysis based on cross-correlation of the intensities in two normal species spectra upon changes in the Bz concentration. Intensity analysis based on cross-correlation of the intensities (*I*_1_, *I*_2_) in the two normal species spectra upon changes in the Bz concentration (Bz 0.4 and 1.2 %, respectively). **A** and **B** show the peak clustering distribution in both polar and Cartesian representations. The clustering of peaks around a particular intensity ratio given by $\arctan ({I}_{2}/{I}_{1})$ is shown following the color code. **C** shows a histogram with the number of peaks corresponding to each cluster.

On the experimental side, a common challenge in rotational spectroscopy is the quantitative analysis of dense spectra, where multiple species are measured simultaneously. Although other approaches are possible, here we developed a cross-correlation analysis^28,30,31^ of the intensity variations upon changes in the Bz concentration. To quantitatively compare the two spectra and classify peaks based on their relative intensity changes, we implemented a custom analysis pipeline in Python. First, peaks were identified in the primary spectrum (Bz 1.2 %) using an intensity threshold, leveraging the *find peaks* function from the SciPy library. For each identified peak frequency, *I*_1_, the corresponding intensity in the secondary spectrum (Bz 0.4 %), *I*_2_, was determined using linear interpolation. The resulting intensity pairs, (*I*_1_, *I*_2_), were then transformed into a polar coordinate system, where the angle, $\theta =\arctan ({I}_{2}/{I}_{1})$, serves as a robust metric for the relative change in intensity. To group peaks exhibiting similar behavior, we developed a clustering algorithm based on an intensity-weighted angle histogram, which identifies significant cluster centers by finding maxima in the angle distribution weighted by peak intensity. This approach effectively partitions the peaks into distinct subpopulations, each corresponding to a specific pattern of intensity modulation. The results of this classification are visualized in an angle-versus-log(intensity) plot (both polar and cartesian, as shown in Fig. 2), where each cluster is distinctly color-coded, providing an immediate qualitative assessment of the dominant changes. Furthermore, each cluster is plotted in a histogram indicating the number of constituent peaks of each cluster, along with any unclassified outliers. This allows a clear visualization of the relevant clustering ratios. Lastly, the peaks of each cluster are exported to separate data files, enabling detailed quantitative interrogation and subsequent analysis of each subpopulation of transitions. As shown in Fig. 2, there is a clear clustering of rotational transitions for angles 24.6^∘^ to 28^∘^, 41.8^∘^, 65^∘^ to 71^∘^, and 79.4^∘^. In the following, we focus on the four distinct groups of transitions identified by the clustering-based filtering analysis. The subset at 24.6^∘^ to 28.0^∘^ corresponds to the Py-Bz dimer, which is cleanly extracted as a strong *a*-type sequence with rich fine structure arising from both the quadrupolar nucleus and the internal dynamics of the complex. The selective filtering in this region effectively disentangles the Py-Bz signature from surrounding congestion. The remaining transitions in this window remain unassigned, and their analysis is ongoing. No evidence for larger Py-Bz clusters was observed. The next grouping, centered at 41.8^∘^, contains the largest number of features, with approximately 1250 transitions.

**Fig. 3:**
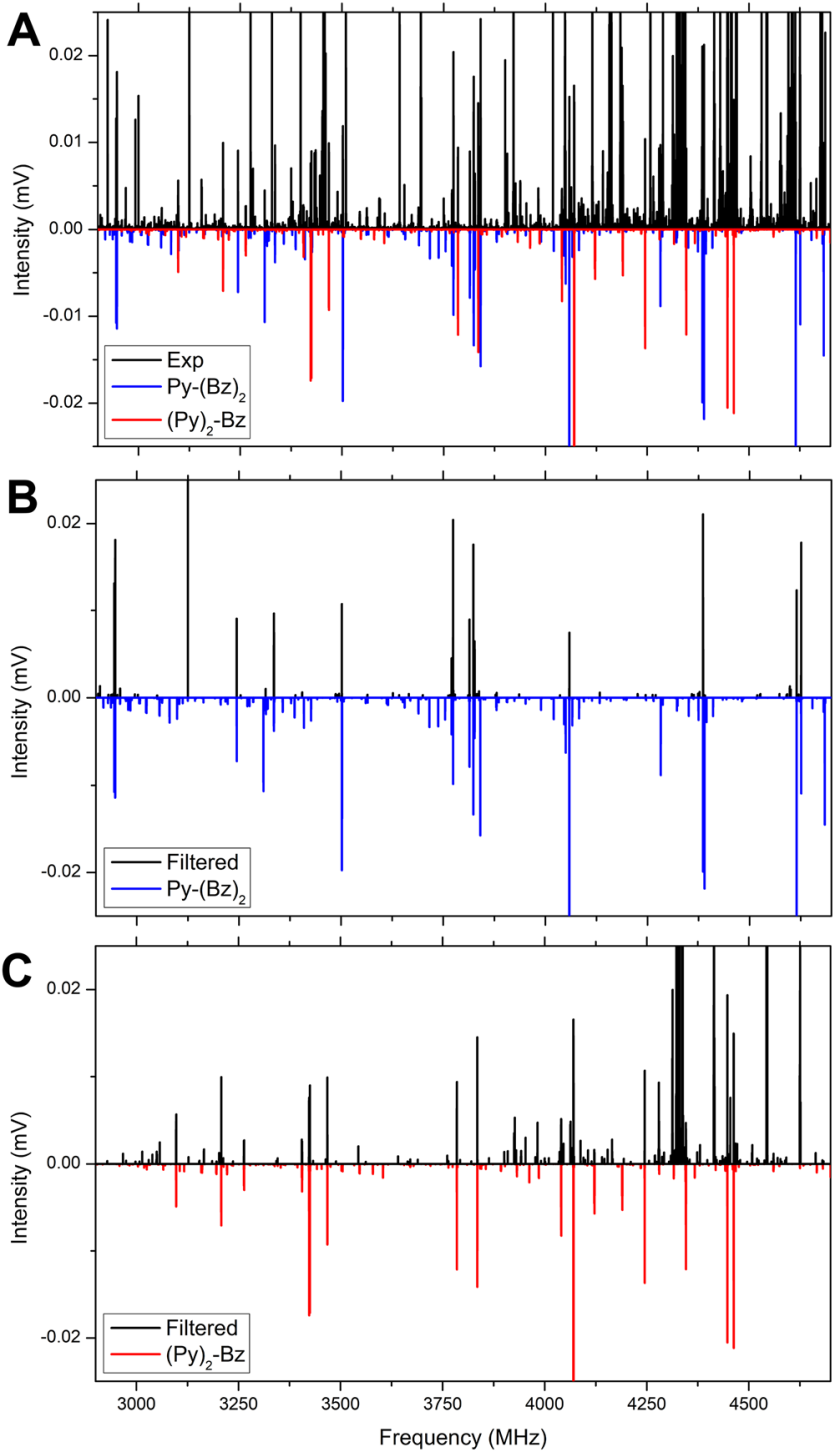
Raw spectrum and filtered spectra isolating mixed trimers. **A** Segment of the raw broadband spectrum (black), overlaid with inverted simulations of both mixed trimers, illustrating the severe line congestion. **B** Intensity-filtered spectrum isolating the Py-(Bz)_2_ trimer, revealing its predominantly *b*-type rotational pattern. **C** Corresponding filtered spectrum isolating the (Py)_2_-Bz trimer and recovering its characteristic *a*-type transitions. The filtering efficiently decomposes the raw data into chemically distinct spectral subsets.

Guided by quantum-chemical predictions, the filtered spectrum reveals a predominantly *b*-type rotational pattern, confidently assigned to the Py-(Bz)_2_ trimer. The results of the filtering and assignments are shown in Fig. 3B, highlighting both the efficiency and clarity gained from this approach. The third group, at 65^∘^ to 71^∘^, isolates the carrier of a strong *a*-type spectrum that increases in relative intensity at lower benzene concentrations, consistent with (Py)_2_-Bz; this assignment is shown in Fig. 3C alongside the raw spectrum for comparison. Finally, the group at 79.4^∘^ filters to a cluster containing only Py units, corresponding to the Py dimer. The rotational parameters for the two mixed trimers are reported in Table 1, while the transitions are tabulated in the Supporting Information. Measured transition frequencies in all spectra were fit using Watson’s A-reduced Hamiltonian in the I^*r*^ representation as implemented in the SPFIT/SPCAT program suite^51^. Together, these results show that clustering-based filtering provides a powerful means to decompose congested spectra into chemically interpretable subsets, effectively isolating species by their chemical composition.

**Table 1:** Experimentally determined rotational parameters for the Py-(Bz)_2_ and (Py)_2_-Bz trimers and the (Py)_2_-(Bz)_2_ tetramer

	Py-(Bz)_2_		(Py)_2_-Bz		(Py)_2_-(Bz)_2_	
	Exp.	Theo.	Exp.	Theo.	Exp.	Theo.
A (MHz)	491.42580(11)	493.06	575.75643(16)	577.4	477.877(57)	477.8
B (MHz)	404.123446(88)	411.4	467.37093(12)	472.6	173.1012(57)	176.6
C (MHz)	278.195243(97)	282.4	323.31877(11)	326.4	147.73408(90)	151.1
*Δ*_*J*_ (kHz)	0.04476(57)	-	720.05231(64)	-	0.1789(80)	-
*Δ*_*JK*_ (kHz)	0.0619(20)	-	[0]	-	-2.28(64)	-
*Δ*_*K*_ (kHz)	-0.0396(14)	-	0.0400(45)	-	105.7(78)	-
*δ*_*J*_ (kHz)	20.01319(28)	-	0.01615(46)	-	0.0888(42)	-
*δ*_*K*_ (kHz)	[0]	-	−0.0102(24)	-	1.36(37)	-
*χ*_*aa*_ (MHz)	−2.2464(23)	−2.49	−0.3643(41)	−0.35	-	−1.96
*χ*_*bb*_ (MHz)	0.9352(51)	1.11	−0.9591(51)	−1.02	-	0.55
*χ*_*cc*_ (MHz)	1.3112(51)	1.37	1.3233(51)	1.37	-	1.41
*χ*_*aa*_ (MHz)	-	-	-2.0039(33)	−2.13	-	−0.78
*χ*_*bb*_ (MHz)	-	-	0.6676(51)	0.74	-	−0.60
*χ*_*cc*_ (MHz)	-	-	1.3363(51)	1.39	-	1.38
*σ* (kHz)	6.3		7.2		15.5	
*N*	317		395		40	

A, B, and C are the rotational constants. *Δ*_*J*_, *Δ*_*JK*_, *Δ*_*K*_, *δ*_*J*_, *δ*_*K*_ are the centrifugal distortion constants in Watson’s A-reduction. *χ*_*aa*_, *χ*_*bb*_, and *χ*_*cc*_ are the quadrupole coupling constants. *σ* is the deviation of the fit, and *N* is the number of transitions in the fit. Theoretical parameters correspond to the *C*_*s*_ geometries at the *ω*B97X-D/6-31++G** level.

Once the two trimers were identified, we leveraged the high resolution and sensitivity of our technique to characterize the three-dimensional structures of the observed clusters accurately. The experimental signal-to-noise ratio was sufficient to observe the ^13^C and ^15^N mono-substituted spectra at natural abundance, as illustrated in Fig. 1 for both trimers. Although the Py-(Bz)_2_ complex contains 16 carbon atoms, only 10 distinct single-^13^C spectra were observed, indicating that pairs of carbon atoms are equivalent in a vibrationally averaged *C*_*s*_ symmetric structure. Similarly, the (Py)_2_-Bz complex has 14 carbon atoms, but only 8 single-^13^C spectra were detected, consistent with a *C*_*s*_-symmetric vibrationally averaged structure. For both mixed trimers, quantum-chemical calculations predict slightly asymmetric structures representing a double-well potential. However, the calculated barrier to interconversion lies below the zero-point energy (ZPE), and the symmetric transition state closely resembles the ground-state geometry. In this regime, the system undergoes rapid tunneling that effectively averages the structure to *C*_*s*_ symmetry. This dynamical averaging is supported by the observation of only 10 and 8 distinct ^13^C isotopologues for the Py-(Bz)_2_ and (Py)_2_-Bz trimers, respectively, which is consistent with vibrationally averaged equivalence. Because the ZPE resides above the barrier, the rotational transitions do not exhibit resolvable tunneling splittings, allowing the spectra to be fit to experimental accuracy using a standard Watson’s A-reduced Hamiltonian. *C*_*s*_ geometries were obtained using *ω*B97X-D/6-31++G** transition state optimizations. Further calculations at the MP2/haug-cc-pVDZ level, enforcing *C*_*s*_ symmetry, were also performed and confirmed a very shallow double-well potential with barriers of about 0.02 kJ mol^−1^. See the Supporting Information for more details. For the Py-(Bz)_2_ complex, we observed the single ^15^N isotopologue. However, the signal-to-noise ratio was insufficient to detect the two single ^15^N isotopic species for the (Py)_2_-Bz complex. Additionally, we observed the single-D substitution for both clusters under the conditions described previously.

An initial analysis employed the Kraitchman method^52^, which enables the determination of the magnitude of the coordinates of the substituted atom in the principal axis system. The results from this analysis are reported in the Supporting Information (Table S24–25 and Table S44–45). A comparison of the experimental atom positions (solid, colored spheres) with the structures from theory (shaded structures) is shown in Fig. 1. As shown, there is a remarkable agreement with the predicted structures from quantum-chemical calculations, which allows a confident identification of the observed clusters. Note that this method encounters issues when the substituted atom is close to one of the inertial axes.

Spectra of the mono-deuterated isotopologues were also recorded under conditions designed to maximize single deuterium incorporation, namely a 0.5 % benzene-d_0_ and 0.5 % benzene-d_1_ mixture in neon. Key regions of these spectra are presented in Fig. 1. The relative intensities of the substitution positions vary, reflecting the relative stabilities of the corresponding isotopologues. In particular, the bottom-left panel of Fig. 1 shows that substitution at position A produces a signal intensity approximately threefold higher than at the other sites. This site corresponds to the CH⋯*π* interaction between the benzene ring (I) and the Py unit, identifying it as the strongest stabilizing interaction within the cluster. For the second benzene ring (II), deuterium incorporation was observed exclusively at position E, which corresponds to the CH⋯*π* interaction between rings II and I. These findings highlight the dominant role of CH⋯*π* contacts in defining the stability of the cluster. The enhanced stability of these configurations can be attributed to differences in zero-point vibrational energy between hydrogen and deuterium, with deuterium lowering the vibrational energy contribution and thereby favoring substitution at these particular positions.

**Fig. 4:**
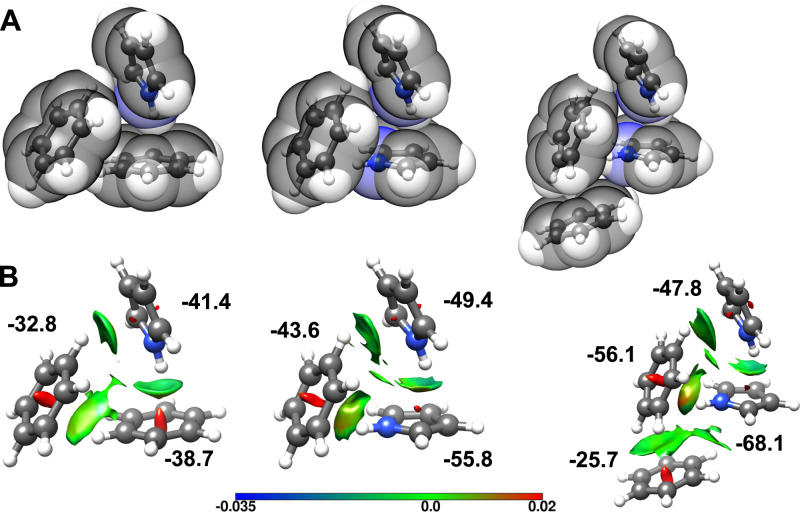
Space-filling representations, NCI analysis, and binding energies of Py-Bz clusters. **A** Van der Waals surfaces illustrating the space-filling representations of the Py-(Bz)_2_ and (Py)_2_-Bz trimers, as well as the (Py)_2_-(Bz)_2_ tetramer, computed at the CCSD(T)/aug-cc-pVQZ//*ω*B97X-D/6-31++G** level of theory. These models highlight the overall molecular shapes and intermolecular packing within the clusters. **B** NCI analysis, mapping the location and strength of intermolecular interactions. Interactions range from attractive in blue to repulsive interactions in red based on the sign of (*λ*_2_)*ρ*. *λ*_2_ is the second eigenvalue of the electron density Hessian, and *ρ* is the electron density in atomic units. The binding energies of each molecular unit are also displayed. Values are in kJ/mol and are calculated at the DLPNO-CCSD(T)/haug-cc-pVQZ//*ω*B97X-D/6-31++G** level of theory.

**Fig. 5:**
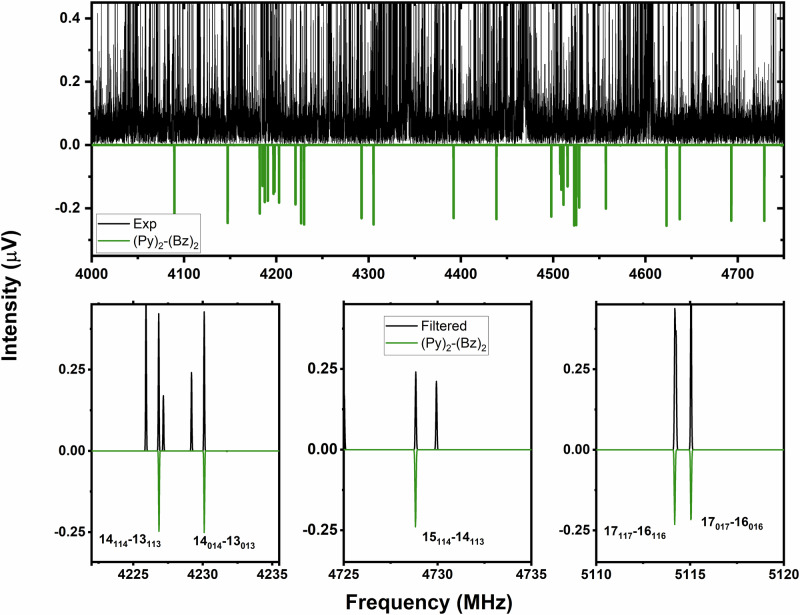
High-density spectrum and filtered signal of the (Py)_2_-(Bz)_2_ tetramer. The top panel shows a key portion of the experimental spectrum, highlighting the high spectral density and intensity of the tetramer signal. The green trace (negative scale) is the simulated spectrum for the mixed tetramer (Py)_2_-(Bz)_2_, based on the fitted rotational parameters reported in Table 1. The second row displays relevant sections of the spectrum after applying the size-filtering procedure that enabled identification of the tetramer. Rotational levels involved in each transition are labeled using standard asymmetric-top notation, ${{{{{\rm{J}}}}}}_{{K}_{a}{K}_{c}}$, where J is the total angular momentum quantum number and K_*a*_, K_*c*_ are the pseudo quantum numbers corresponding to projections of the angular momentum on the a- and c-axes in the prolate and oblate symmetric-top limits, respectively.

Given the high spectral density, we investigated the possible formation of larger Py-Bz aggregates, focusing specifically on the tetramers Py-(Bz)_3_, (Py)_3_-Bz, and (Py)_2_-(Bz)_2_. The same theoretical protocol employed for the trimers was applied, and the corresponding results are provided in the Supporting Information. After applying the size-filtering criteria, extensive searches were carried out for tetramers of each composition. The non-observation of Py-(Bz)_3_ and (Py)_3_-Bz is likely a consequence of the detection limit, as these larger clusters fall near the sensitivity threshold of the experiment. In contrast, the filter successfully extracted sufficient transitions to enable a conclusive assignment of the mixed tetramer (Py)_2_-(Bz)_2_. The experimental rotational parameters are reported in Table 1, and the structure is displayed along the mixed trimers in Fig. 4. The high line density at the signal level of this species, as well as the effect of the filtering procedure that allowed the detection of the tetramer, is illustrated in Fig. 5.

Preliminary analysis using the substitution Kraitchman method was invaluable for confirmation of the predicted cluster geometries and for the assignment of isotopic species. At the same time, the chemically relevant relative orientation of the molecules in each cluster was more conveniently determined using least squares fits to all available rotational constants. This was carried out using the program STRFIT^53^, with the analysis aimed at determining intermolecular center of mass distances (marked in Fig. 1) and orientation angles between the molecules. Two sets of fits were explored by assuming monomer structures from the supermolecule computation and, alternatively, by using the experimentally based *r*_*e*_ structures for pyrrole^54^ and benzene^55^. Relative orientation of a pair of molecules under the constraint of *C*_*s*_ cluster symmetry requires only the center of mass separation and two orientation angles. In consequence, only six parameters of fit are required for each trimer, although some refinements were necessary. It was first necessary to deal with several persistent outliers in the fits, specifically the deuterated species A in both clusters and E for Py-(Bz)_2_ (see Fig. 1). These were successfully accounted for once it was realized that the relevant C-H bonds were directed close to the intermolecular axis joining two monomers with hydrogen atoms pointing towards the pertinent neighbor. In such cases, deuterium substitution was likely to lead to the Ubbelohde^56^ shortening of the intermolecular distance. A shortening of 0.003 Å was sufficient to ensure a satisfactory fit for the problematic deuterium species, and this value is close to 0.0025 Å found for the H_2_O⋯HCl cluster^57^. Another problem to be tackled was fit degradation due to small deviations from *C*_*s*_ symmetry. This was due to unaccounted-for vibrational contributions to ground state constants, and their magnitude was exploited with parameters specific to Watson’s *r*_*m*_ geometry^58^. It turned out that the fit based on experimental monomer geometries required minimal corrections of this type. The reported least squares structural results are estimated to be essentially of *r*_0_ quality, as evident from systematically larger fitted center of mass separations than those from computation. The uncertainties marked in Fig. 1 arise from statistical errors in the fits and are likely underestimated. Nonetheless, comparison of various fits leads us to expect realistic uncertainties on these quantities to be at the 0.01 Å level. These results are discussed in detail in the Supporting Information.

This detailed analysis enables an in-depth discussion of the geometrical arrangements of the observed clusters. Both mixed trimers adopt triangular-like configurations (Figs. 1 and 4), in which the Py and Bz monomers are connected through N-H⋯*π* and C-H⋯*π* interactions, respectively. The relative orientation of a pair of monomers within the *C*_*s*_-symmetric clusters can be fully described by the center-of-mass separation and two orientation angles. Figure 1 shows the center-of-mass distances derived from the structural analysis, while a more comprehensive representation of each trimer, including the corresponding orientation angles, is provided in the supporting information. For the (Py)-(Bz)_2_ trimer, the distances between the ring centers of mass are 4.719(4) Å for Py-Bz(I), 4.381(3) Å for Py-Bz(II), and 4.735(3) Å between the two Bz rings, forming a nearly equilateral triangle in the a-b inertial plane of the cluster. Similarly, the (Py)_2_-(Bz) trimer exhibits separations of 4.263(7) Å for Bz-Py(I), 4.630(6) Å for Bz-Py(II), and 4.272(7) Å between the two Py rings. As shown in Fig. 1, there is a good agreement with the distances obtained by quantum chemistry (black values). The observed orientations and intermolecular separations indicate that the overall geometries can be rationalized in terms of a space-filling model, in which efficient packing modulates-and ultimately prevails over-the specific interactions between individual aromatic moieties. The structural hierarchy of the mixed Py-Bz clusters reflects a compromise between the more directional anchors of homo-pyrrole assemblies and the flexible, dispersion-driven motifs of pure benzene. While benzene trimers typically adopt propeller-like or cyclic T-shaped configurations to maximize C-H⋅*π* dispersive contacts, and pyrrole homo-trimers are anchored by robust N-H⋅*π* hydrogen bonds, the hetero-clusters studied here demonstrate a distinct priority. Although the N-H bond acts as a high-stability primary anchor, the specific intermolecular separations, such as the 4.719(4) Å distance in (Py)-(Bz)_2_ and the 4.272(7) Å distance between Py units in (Py)_2_-(Bz), reveal that the system does not exclusively optimize this single directional force. Instead, the molecules adopt triangular, nearly equilateral arrangements that maximize the total molecular envelope contact. This confirms that the presence of the N-H group serves to orient the cluster, but the final three-dimensional architecture is ultimately dictated by global space-filling principles that favor dense, dispersion-stabilized packing over purely directional motifs.

To further assess the validity of this space-filling description, van der Waals surface models were computed at the CCSD(T)/aug-cc-pVQZ//*ω*B97X-D/6-31++G** level of theory. These representations depict the overall molecular envelopes and highlight how efficient packing and complementary intermolecular contacts contribute to the stability of the clusters (Fig. 4A). Additionally, to characterize the noncovalent interactions within the system, Noncovalent Interaction (NCI) analysis^59^ was performed using the computational package Multiwfn^60,61^. The results of this analysis, which provide a qualitative three-dimensional visualization of the interaction regions, are shown in Fig. 4B. This visualization clearly delineates the regions where localized N-H⋯*π* and C-H⋯*π* interactions are established between the rings.

A similar packing principle extends to the (Py)_2_-(Bz)_2_ tetramer. In this larger aggregate, the additional monomers complete an assembly that preserves the key N-H⋯*π* and C-H⋯*π* interactions observed in the trimers while maintaining efficient overall packing. The resulting structure can be viewed as the (Py)_2_-(Bz) motif augmented by a second Bz ring, which establishes a C-H⋯*π* contact with the Py(I) unit of the trimer and a *π*⋯C−H interaction with the other Bz ring. The optimized structure of the tetramer, shown with van der Waals surfaces in Fig. 4A, further illustrates this compact packing. Interestingly, the tetrameric arrangement could also be generated starting from the (Py)-(Bz)_2_ trimer through the addition of a Py monomer; however, this pathway would require a partial structural rearrangement to accommodate the new hydrogen-bonding and packing interactions.

To elucidate the energetic factors governing these assemblies, an energy decomposition analysis was performed to quantify the individual contributions of each monomer to the total binding energy of the cluster according to the expression *E*_bind_(*A*) = *E*_el_(*ABC*) − *E*_el_(*BC*) − *E*_el_(*A*) for a trimer composed of monomers A, B and C. This expression can similarly be written for the tetramers, composed of monomers A, B, C, and D. Single-point calculations for the corresponding fragments were carried out at the DLPNO-CCSD(T)/haug-cc-pVQZ//*ω*B97X-D/6-31++G** level of theory. The resulting binding energies, expressed in kJ mol^−1^, are displayed in Fig. 4B. For the (Py)-(Bz)_2_ trimer, the Py unit contributes most strongly (41.5 kJ mol^−1^), consistent with its dual interaction with both Bz rings. The somewhat weaker stabilization of the Bz monomers reflects the absence of a heteroatom donor, highlighting the reinforcing role of the pyrrole nitrogen in the N-H⋯*π* contacts. In the (Py)_2_-(Bz) trimer, the Py units again dominate the binding, contributing 49.4 kJ mol^−1^ and 55.8 kJ mol^−1^, while the Bz monomer binds more weakly (43.6 kJ mol^−1^). These values indicate that the strongest interaction within this cluster arises from the N-H⋯*π* contact between the two Py rings.

In the (Py)_2_-(Bz)_2_ tetramer, the energetic landscape becomes more intricate due to the larger number of distinct trimers and the four-body interaction that contributes to the shape of the potential energy surface. Relative to the smaller (Py)_2_-(Bz) trimer motif embedded within the tetramer, the binding energy of the original Bz ring increases from 43.6 kJ mol^−1^ to 56.1 kJ mol^−1^, driven by additional stabilization from the newly incorporated Bz unit. This second Bz monomer, in turn, contributes more modestly (25.7 kJ mol^−1^), consistent with its weaker interaction network involving only one Bz and one Py partner. The central Py unit exhibits the largest contribution, 68.1 kJ mol^−1^, as it engages simultaneously with all three neighboring rings. Additionally, we also computed a many-body energy expansion to evaluate the non-additive three- and four-body contributions. The outcome of this analysis is reported in the SI. Overall, these results reveal a cooperative stabilization pattern in which the nitrogen-containing Py units serve as energetic anchors within the aromatic assembly, mediating and reinforcing the network of *π*- and C-H⋯*π* interactions that define the compact packing motif.

## Conclusions

Broadband rotational spectroscopy, combined with high-level quantum-chemical calculations, provides a detailed structural and energetic picture of pyrrole-benzene clusters up to the tetramer. The results reveal how directional N-H⋯*π* and C-H⋯*π* interactions cooperate with dispersion forces to drive aromatic aggregation into compact and efficiently packed assemblies.

Both mixed trimers adopt triangular configurations, with intermolecular separations between 4.26 Å and 4.73 Å, reflecting the balance between specific hydrogen-bonding motifs and global packing efficiency within the aromatic framework. Van der Waals surface models and NCI analyses confirm that, beyond localized N-H⋯*π* and C-H⋯*π* contacts, extensive dispersion interactions dominate the stabilization of the clusters-consistent with a space-filling model of aromatic aggregation, where efficient molecular packing prevails over individual pairwise interactions.

This packing principle extends naturally to the (Py)_2_-(Bz)_2_ tetramer, which preserves the key hydrogen-bond motifs of the trimers while forming a denser and more cooperative network of interactions. The additional benzene ring stabilizes the aggregate through new C-H⋯*π* and *π*⋯C−H contacts, illustrating how weak, cumulative forces underpin the growth of larger aromatic assemblies. Although alternative growth pathways, such as the addition of pyrrole to the (Py)-(Bz)_2_ trimer, are energetically plausible, they would entail structural rearrangements, highlighting the delicate energy landscape governing aggregation.

Energy-decomposition analysis at the DLPNO-CCSD(T)/haug-cc-pVQZ//*ω*B97X-D/6-31++G** level further elucidates the cooperative nature of these interactions. The pyrrole monomers contribute most strongly to the stabilization, with binding energies of 41 kJ mol^−1^ to 56 kJ mol^−1^ in the trimers and up to 68 kJ mol^−1^ in the tetramer. The additional benzene ring, although less tightly bound (25 kJ mol^−1^), enhances the stabilization of neighboring units through three-body dispersion and collective packing effects. These energetic trends confirm that efficient stacking and dispersion-driven packing, rather than purely directional hydrogen bonding, dictate the geometries of these aromatic clusters.

Methodologically, this work demonstrates that recently developed intensity-based spectral filtering through cross-correlation provides a powerful route to disentangle overlapping rotational spectra by size and composition, enabling the isolation of individual cluster signatures within congested broadband data. This capability extends the scope of rotational spectroscopy to increasingly complex molecular aggregates and heterogeneous aromatic mixtures, where traditional line-by-line analysis becomes intractable.

In summary, this study establishes a quantitative and conceptual link between hydrogen bonding, dispersion, and *π*-stacking in weakly bound aromatic clusters. The cooperative interplay between these forces reveals the microscopic principles governing aromatic aggregation, providing molecular-level insight into how such interactions may guide the assembly and packing of increasingly more complex chemical and biological environments.

## Methods

### Experimental details

Broadband rotational spectra were recorded in the 2–8 GHz range using a chirped-pulse Fourier transform microwave (CP-FTMW) spectrometer. The Py-Bz complexes were generated via co-expansion of benzene (Bz) in neon (0.7 bar backing pressure) over a reservoir of heated pyrrole (Py) (40 ^∘^C), using a pulsed supersonic expansion. Spectra of the parent complexes and their heavy-atom isotopologues (^13^C, ^15^N) were acquired with 1.2% and 0.4% Bz in neon. Mono-deuterated isotopologues were studied using a 0.5% benzene-*d*_0_ and 0.5 % benzene-*d*_1_ mixture in neon. Measured transition frequencies were fit using Watson’s A-reduced Hamiltonian in the *I*^*r*^ representation as implemented in the SPFIT/SPCAT program suite^51^.

### Theoretical calculations

The potential energy surfaces (PESs) for the mixed complexes were explored using a three-step funnel method. Global geometry searches were performed using a genetic algorithm (GA)^38^ and the OGOLEM package^39^, leveraging GFN2-xTB^41,42^ and PM7^40^ semi-empirical methods. Low-energy isomers were optimized using DFT with the *ω*B97X-D functional and the 6−31++G^**^ basis set (Gaussian 16, Revision C.01). Final electronic energy corrections for isomers within 8 kcal mol^−1^ of the global minimum were performed at the DLPNO-CCSD(T) level of theory, employing the haug-cc-pVQZ basis set (ORCA)^46,49,50^. Noncovalent Interaction (NCI) analysis^59^ was performed using Multiwfn^60,61^.

## Supplementary information

Supplementary Information

Description of Additional Supplementary Files

Supplementary Data 1

## Data Availability

The datasets generated and analyzed during the current study are available from the corresponding authors upon reasonable request. The coordinates of the structures from quantum-chemical calculations are compiled in the Supplementary Data 1.

## Code availability

The custom Python analysis pipeline developed for intensity cross-correlation is available from the corresponding authors upon reasonable request.
