# Supplementary material for: Space filling shapes the interaction networks in mixed pyrrole-benzene trimers and tetramers
Source: Commun Chem. 2026 Apr 17;9:213. doi: 10.1038/s42004-026-02027-1 (PMC13272942; doi:10.1038/s42004-026-02027-1)
Supplement: Supplementary file 1 — Supplementary Information [file 42004_2026_2027_MOESM1_ESM.pdf]

Supporting Information

Space filling shapes the interaction networks in the mixed pyrrole–benzene trimers and tetramers

Simon Lobsiger,[†,‡] Zbigniew Kisiel,[¶] Caroline S. Glick,[§] George C. Shields,[§] Brooks H. Pate,[*,†] and Cristóbal Pérez[*,‖,†]

†Department of Chemistry, University of Virginia, Charlottesville, Virginia 22904-4319, USA
‡Federal Institute of Metrology METAS, Lindenweg 50, 3003 Bern-Wabern, Switzerland
¶Institute of Physics, Polish Academy of Sciences, 02-668 Warszawa, Poland
§Department of Chemistry, Furman University, Greenville, SC 29613, USA
‖Departamento de Química Física y Quíımica Inorgánica, Facultad de Ciencias-I.U. CINQUIMA, Universidad de Valladolid, E-47011 Valladolid, Spain.
Centro Singular de Investigación en Química Biolóxica e Materiais Moleculares (CiQUS) and Departamento de Química Física, Universidade de Santiago de Compostela, 15782 Santiago de Compostela, Spain.
E-mail: brookspate@virginia.edu; cristobal.perez@uva.es

Table of contents

1 Py-$(Bz)_2$ trimer 5
1.1 Measured rotational transitions and fits 5
1.2 Experimental rotational parameters 41
1.3 Least Squares Structural Analysis for the Py-$(Bz)_2$ cluster 43
2 $(Py)_2$-Bz trimer 56
2.1 Measured rotational transitions and fits 56
2.2 Experimental rotational parameters 79
2.3 Least Squares Structural Analysis for the $(Py)_2$-Bz cluster 82
3 $(Py)_2$-$(Bz)_2$ tetramer 93
3.1 Measured rotational transitions and fits 93
4 Theoretical calculations 95
4.1 Methodology 95
4.2 Py-$(Bz)_2$ 98
4.3 $(Py)_2$-Bz 100
4.4 $(Py)_2$-$(Bz)_2$ 102
4.5 Py-$(Bz)_3$ 104
4.6 $(Py)_3$-Bz 105
4.7 Many-Body Expansion 106

List of Tables and Figures

Py-$(Bz)_2$ trimer

Measured rotational transitions (ν_{obs}) and residuals (ν_{obs} - ν_{calc}) (frequencies in MHz):

Table S1	$^{12}C/^{14}N/^{1}H$ isotopic species
Table S2	^{13}C (1,3) isotopic species
Table S3	^{13}C (4,5) isotopic species
Table S4	^{13}C (6,10) isotopic species
Table S5	^{13}C (7,9) isotopic species
Table S6	^{13}C (8) isotopic species
Table S7	^{13}C (11) isotopic species
Table S8	^{13}C (23) isotopic species
Table S9	^{13}C (24,28) isotopic species
Table S10	^{13}C (25,27) isotopic species
Table S11	^{13}C (26) isotopic species
Table S12	^{15}N isotopic species
Table S13	D (22) isotopic species
Table S14	D (29) isotopic species
Table S15	D (30,34) isotopic species
Table S16	D (31,33) isotopic species
Table S17	D (32) isotopic species

Experimental rotational parameters:

Table S18	$^{12}C/^{14}N/^{1}H$ (normal) isotopic species
Table S19	mono ^{13}C substituted isotopic species
Table S20	^{15}N isotopic species
Table S21	mono D substituted isotopic species

Structural analysis and parameters:

Table S22	Comparison of the results of least squares structural fits for the Py-$(Bz)_2$ cluster with computed values.
Table S23	The abbreviated results of the least-squares fit of the partial geometry of the Py-$(Bz)_2$ cluster with the STRFIT program
Figure S1	Atom numbering
Figure S2	Structural parameters defining the relative orientation of the three molecules in the Py-$(Bz)_2$ cluster
Table S24	Substitution coordinates (r_s) of the heavy atoms (N, C) from the general Kraitchman equations using the rotational constants of the single ^{13}C and ^{15}N isotopologues.
Table S25	Substitution coordinates (r_s) of the H atoms from the general Kraitchman equations using the rotational constants of the single D isotopologues.

$(Py)_2$-Bz trimer

Measured rotational transitions (ν_{obs}) and residuals (ν_{obs} - ν_{calc}) (frequencies in MHz):

Table S26	$^{12}C/^{14}N/^{1}H$ isotopic species
Table S27	^{13}C (1,3) isotopic species
Table S28	^{13}C (4,5) isotopic species
Table S29	13C (6,10) isotopic species
Table S30	^{13}C (7,9) isotopic species
Table S31	^{13}C (8) isotopic species
Table S32	^{13}C (11) isotopic species
Table S33	^{13}C (23,27) isotopic species
Table S34	^{13}C (25,26) isotopic species
Table S35	D (12) isotopic species
Table S36	D (13,15) isotopic species
Table S37	D (14,16) isotopic species
Table S38	D (24) isotopic species

Experimental rotational parameters:

Table S39	$^{12}C/^{14}N/^{1}H$ (normal) isotopic species
Table S40	^{13}C substituted isotopic species
Table S41	mono D substituted isotopic species

Structural analysis and parameters:

Table S42	Comparison of the results of least squares structural fits for the $(Py)_2$-Bz cluster with computed values.
Table S43	The abbreviated results of the least-squares fit of the partial geometry of the $(Py)_2$-Bz cluster with the STRFIT program.
Figure S3	Atom numbering
Figure S4	Structural parameters defining the relative orientation of the three molecules in the $(Py)_2$-Bz cluster
Table S44	Substitution coordinates (r_s) of the heavy atoms (C) from the general Kraitchman equations using the rotational constants of the single ^{13}C isotopologues.
Table S45	Substitution coordinates (r_s) of the H from the H atoms from the general Kraitchman equations using the rotational constants of the single D isotopologues.

$(Py)_2$-$(Bz)_2$ tetramer

Measured rotational transitions (ν_{obs}) and residuals (ν_{obs} - ν_{calc}) (frequencies in MHz):

Table S46	$^{12}C/^{14}N/^{1}H$ isotopic species

Experimental rotational parameters:

Table S47	$^{12}C/^{14}N/^{1}H$ (normal) isotopic species

Theoretical calculations

Py-$(Bz)_2$:

Figure S5	The six lowest-energy isomers of Py-$(Bz)_2$.
**Table S48**	**Table S48**. Calculated energies, rotational constants, and principal axis dipole moments of Py-$(Bz)_2$. Electronic energies, Gibbs free energies, and Boltzmann populations at 100 K are relative to the global-minimum isomer sampled.

$(Py)_2$-Bz:

Figure S6 The six lowest-energy isomers of $(Py)_2$-Bz.

Table S49 Calculated energies, rotational constants, and principal axis dipole moments of $(Py)_2$-Bz. Electronic energies, Gibbs free energies, and Boltzmann populations at 100 K are relative to the global-minimum isomer sampled.

$(Py)_2$-$(Bz)_2$:

Figure S7 The isomers of $(Py)_2$-$(Bz)_2$ with relative Boltzmann populations above 0.5%.

Table S50 Calculated energies, rotational constants, and principal axis dipole moments of $(Py)_2$-$(Bz)_2$. Electronic energies, Gibbs free energies, and Boltzmann populations at 100 K are relative to the global-minimum isomer sampled.

Py-$(Bz)_3$:

Figure S8 The isomers of Py-$(Bz)_3$ with relative Boltzmann populations above 0.5%.

Table S51 Calculated energies, rotational constants, and principal axis dipole moments of Py-$(Bz)_3$. Electronic energies, Gibbs free energies, and Boltzmann populations at 100 K are relative to the global-minimum isomer sampled.

$(Py)_3$-Bz:

Figure S9 The isomers of $(Py)_3$-Bz with relative Boltzmann populations above 0.5%.

Table S52 Calculated energies, rotational constants, and principal axis dipole moments of $(Py)_3$-Bz. Electronic energies, Gibbs free energies, and Boltzmann populations at 100 K are relative to the global-minimum isomer sampled.

Table S53. Non-additive two-, three-, and four-body interaction energies computed with DLPNO-CCSD(T)/haug-cc-pVQZ, in kJ/mol.

1 Py-(Bz)$_2$ trimer

1.1 Measured rotational transitions and fits

Table S1. Measured rotational transitions (ν_{obs}) of the $^{12}C/^{14}N/^{1}H$ isotopic species of the Py-(Bz)$_2$ trimer and residuals (ν_{obs} - ν_{calc}) (frequencies in MHz).

J'	K_a'	K_c'	F'	←	J''	K_a''	K_c''	F''	ν_{obs}	$\nu_{obs}-\nu_{calc}$
11	6	6	10	←	11	5	7	10	2014.8112	-0.0013
10	5	6	9	←	10	4	7	9	2034.9738	0.0042
10	5	6	10	←	10	4	7	10	2035.0931	0.0019
9	3	6	10	←	9	2	7	10	2045.5435	-0.0001
9	3	6	9	←	9	2	7	9	2045.6629	0.0033
9	4	6	10	←	9	3	7	10	2064.3953	0.0000
9	4	6	9	←	9	3	7	9	2064.5310	-0.0027
8	2	6	7	←	8	1	7	7	2088.0840	-0.0189
8	2	6	9	←	8	1	7	9	2088.1373	0.0118
8	2	6	8	←	8	1	7	8	2088.3028	-0.0009
8	3	6	7	←	8	2	7	7	2092.3119	-0.0050
8	3	6	9	←	8	2	7	9	2092.3459	0.0056
8	3	6	8	←	8	2	7	8	2092.5234	-0.0017
7	1	6	7	←	7	0	7	7	2113.7324	0.0015
7	2	6	7	←	7	1	7	7	2114.3828	0.0004
11	9	2	10	←	11	8	3	10	2139.1197	0.0025
11	9	3	10	←	11	8	4	10	2181.4937	0.0033
10	9	1	9	←	10	8	2	9	2228.4902	0.0026
10	9	1	10	←	10	8	2	10	2228.9425	-0.0042
10	9	2	9	←	10	8	3	9	2236.8681	0.0148
10	9	2	10	←	10	8	3	10	2237.2935	-0.0110
9	9	0	8	←	9	8	1	8	2282.1613	-0.0012
9	9	0	10	←	9	8	1	10	2282.2097	-0.0082
9	9	1	8	←	9	8	2	8	2283.2485	-0.0008
9	9	1	9	←	9	8	2	9	2283.7947	-0.0040
3	2	2	2	←	2	1	1	1	2308.6203	-0.0024
3	2	2	3	←	2	1	1	3	2308.7917	0.0016
3	2	2	3	←	2	1	1	2	2309.0936	0.0030
11	4	7	10	←	11	3	8	10	2338.8633	0.0028
11	5	7	10	←	11	4	8	10	2358.3052	0.0022
4	0	4	4	←	3	1	3	4	2384.4114	-0.0010
4	0	4	4	←	3	1	3	3	2385.0196	0.0059
4	0	4	5	←	3	1	3	4	2385.0874	-0.0105
4	0	4	3	←	3	1	3	3	2385.8773	0.0014
10	3	7	9	←	10	2	8	9	2388.4963	0.0067
10	3	7	10	←	10	2	8	10	2388.6259	-0.0031
10	4	7	9	←	10	3	8	9	2393.3741	0.0113
10	4	7	10	←	10	3	8	10	2393.5060	-0.0023
4	1	4	4	←	3	0	3	4	2394.0988	-0.0021
4	1	4	3	←	3	0	3	3	2395.5929	-0.0017
9	2	7	8	←	9	1	8	8	2420.5992	-0.0161
9	2	7	10	←	9	1	8	10	2420.6458	0.0111
9	2	7	9	←	9	1	8	9	2420.8091	0.0017
9	3	7	8	←	9	2	8	8	2421.5355	-0.0186
9	3	7	10	←	9	2	8	10	2421.5856	0.0119
9	3	7	9	←	9	2	8	9	2421.7458	-0.0018
8	1	7	9	←	8	0	8	9	2442.3040	-0.0069
8	2	7	9	←	8	1	8	9	2442.4323	-0.0037
8	1	7	8	←	8	0	8	8	2442.5414	0.0016
8	2	7	8	←	8	1	8	8	2442.6656	0.0004
4	2	2	4	←	3	3	1	3	2478.6604	-0.0125
4	2	2	5	←	3	3	1	4	2479.9223	0.0007
11	10	1	10	←	11	9	2	10	2508.7399	0.0031
11	10	2	10	←	11	9	3	10	2511.5487	0.0055
4	1	3	4	←	3	2	2	3	2618.1107	-0.0008
4	1	3	5	←	3	2	2	4	2618.5277	0.0000
4	1	3	3	←	3	2	2	3	2618.6346	-0.0001
11	4	8	10	←	11	3	9	10	2724.6629	0.0100

J'	K_a'	K_c'	F'	←	J''	K_a''	K_c''	F''	ν_{obs}	$\nu_{obs}-\nu_{calc}$
10	2	8	9	←	10	1	9	9	2750.8680	0.0156
10	2	8	10	←	10	1	9	10	2751.0280	-0.0052
10	3	8	10	←	10	2	9	10	2751.2278	-0.0001
3	3	1	2	←	2	2	0	2	2763.2208	-0.0013
3	3	1	4	←	2	2	0	3	2764.1683	0.0012
3	3	1	3	←	2	2	0	2	2764.4578	0.0029
3	3	1	3	←	2	2	0	3	2765.0829	0.0027
9	1	8	9	←	9	0	9	9	2770.8669	-0.0096
9	2	8	9	←	9	1	9	9	2770.9103	0.0109
4	2	3	3	←	3	1	2	2	2809.3179	0.0039
4	2	3	5	←	3	1	2	4	2809.3911	0.0010
4	2	3	4	←	3	1	2	3	2809.5370	0.0027
4	2	3	3	←	3	1	2	3	2809.8831	0.0024
3	3	0	2	←	2	2	1	2	2863.9423	0.0013
3	3	0	4	←	2	2	1	3	2864.9642	0.0016
3	3	0	3	←	2	2	1	2	2865.0902	-0.0070
3	3	0	3	←	2	2	1	3	2865.8233	0.0041
7	5	2	6	←	7	2	5	6	2938.8021	-0.0051
5	3	2	5	←	4	4	1	4	2939.6072	-0.0054
5	3	2	6	←	4	4	1	5	2940.8851	-0.0058
5	3	2	4	←	4	4	1	3	2941.2099	0.0026
5	0	5	5	←	4	1	4	5	2944.3770	-0.0001
5	0	5	5	←	4	1	4	4	2945.0478	-0.0085
5	0	5	6	←	4	1	4	5	2945.1074	0.0028
5	0	5	4	←	4	1	4	4	2945.9315	-0.0006
5	1	5	5	←	4	0	4	5	2946.3367	0.0056
5	1	5	6	←	4	0	4	5	2947.0431	-0.0143
5	1	5	4	←	4	0	4	4	2947.8930	0.0018
3	2	1	2	←	2	1	2	1	2958.7305	0.0095
3	2	1	4	←	2	1	2	3	2958.9184	0.0012
3	2	1	3	←	2	1	2	2	2959.2287	-0.0008
6	3	4	7	←	5	4	1	6	2972.4068	0.0023
6	4	3	6	←	5	5	0	5	3049.4918	-0.0063
11	3	9	10	←	11	2	10	10	3080.2324	-0.0056
10	1	9	9	←	10	0	10	9	3098.7926	-0.0048
10	1	9	10	←	10	0	10	10	3099.0155	0.0048
10	2	9	10	←	10	1	10	10	3099.0155	0.0007
8	6	2	8	←	8	3	5	8	3100.5825	-0.0040
5	1	4	5	←	4	2	3	5	3244.3785	-0.0010
5	1	4	5	←	4	2	3	4	3244.6557	0.0008
5	1	4	6	←	4	2	3	5	3244.8085	-0.0099
5	1	4	4	←	4	2	3	4	3245.1843	0.0009
5	2	4	5	←	4	1	3	5	3309.5914	0.0000
5	2	4	6	←	4	1	3	5	3310.0024	0.0070
5	2	3	5	←	4	3	2	4	3315.4671	-0.0031
5	2	3	6	←	4	3	2	5	3316.0706	-0.0014
5	2	3	4	←	4	3	2	3	3316.2049	-0.0020
4	3	2	3	←	3	2	1	2	3335.5374	0.0007
4	3	2	5	←	3	2	1	4	3335.6367	0.0020
4	3	2	4	←	3	2	1	4	3335.8701	0.0029
4	3	2	4	←	3	2	1	3	3335.9797	0.0035
11	1	10	10	←	11	0	11	10	3426.8516	0.0189
11	2	10	10	←	11	1	11	10	3426.8516	0.0182
6	0	6	6	←	5	1	5	6	3501.4134	0.0001
6	1	6	6	←	5	0	5	6	3501.7758	0.0052
6	0	6	5	←	5	1	5	4	3502.1569	0.0030
6	1	6	5	←	5	0	5	4	3502.5045	-0.0063
6	0	6	5	←	5	1	5	5	3503.0341	0.0056
6	1	6	5	←	5	0	5	5	3503.3855	-0.0010
7	4	4	7	←	6	5	1	6	3669.1233	0.0039
4	3	1	5	←	3	2	2	4	3770.2559	0.0076
4	3	1	4	←	3	2	2	4	3770.3442	-0.0013
4	4	1	3	←	3	3	0	3	3772.7891	-0.0003
4	4	1	5	←	3	3	0	4	3773.9212	0.0075
4	4	1	4	←	3	3	0	3	3774.1001	0.0018
4	4	1	4	←	3	3	0	4	3774.9575	0.0026
4	4	0	3	←	3	3	1	3	3813.1596	-0.0015

J'	K_a'	K_c'	F'	←	J''	K_a''	K_c''	F''	ν_{obs}	$\nu_{obs}-\nu_{calc}$
4	4	0	5	←	3	3	1	4	3814.3359	-0.0008
4	4	0	4	←	3	3	1	3	3814.4405	-0.0041
4	4	0	4	←	3	3	1	4	3815.3580	0.0000
6	1	5	6	←	5	2	4	6	3823.2950	0.0001
6	1	5	6	←	5	2	4	5	3823.7012	0.0023
6	1	5	7	←	5	2	4	6	3823.7814	-0.0003
6	1	5	5	←	5	2	4	5	3824.2685	0.0003
5	3	3	5	←	4	2	2	5	3826.7997	0.0147
5	3	3	6	←	4	2	2	5	3826.8736	0.0030
5	3	3	5	←	4	2	2	4	3827.1231	0.0027
6	2	5	6	←	5	1	4	6	3840.4598	0.0007
6	2	5	5	←	5	1	4	5	3841.4588	0.0000
6	3	3	6	←	5	4	2	5	3879.6249	-0.0042
6	3	3	7	←	5	4	2	6	3880.3823	-0.0006
6	3	3	5	←	5	4	2	4	3880.5204	-0.0037
6	2	4	6	←	5	3	3	5	4049.3727	-0.0001
7	0	7	7	←	6	1	6	7	4057.8556	-0.0035
7	0	7	6	←	6	1	6	6	4059.5169	-0.0017
8	4	5	8	←	7	5	2	7	4093.9382	0.0091
8	4	5	9	←	7	5	2	8	4094.2654	0.0193
4	2	2	4	←	3	1	3	3	4133.2788	-0.0071
6	3	4	6	←	5	2	3	6	4282.1992	0.0003
6	3	4	7	←	5	2	3	6	4282.4367	-0.0070
6	3	4	6	←	5	2	3	5	4282.5717	0.0034
6	3	4	5	←	5	2	3	5	4282.8541	-0.0005
7	4	3	8	←	6	5	2	7	4323.8290	0.0061
7	1	6	7	←	6	2	5	7	4385.2613	0.0043
7	1	6	8	←	6	2	5	7	4385.7873	-0.0049
5	4	2	5	←	4	3	1	4	4386.3817	0.0014
5	4	2	6	←	4	3	1	5	4386.0755	0.0021
7	2	6	6	←	6	1	5	5	4389.6354	-0.0076
7	2	6	6	←	6	1	5	6	4390.2115	-0.0009
4	3	2	3	←	3	0	3	2	4480.9770	0.0088
4	3	2	5	←	3	0	3	4	4481.2471	-0.0047
4	3	2	4	←	3	0	3	3	4482.1251	0.0013
8	1	8	8	←	7	0	7	7	4615.0006	-0.0036
8	0	8	7	←	7	1	7	7	4615.9141	0.0120
5	4	1	4	←	4	3	2	4	4625.8112	-0.0029
5	4	1	6	←	4	3	2	5	4626.1088	-0.0024
5	4	1	5	←	4	3	2	4	4626.1948	-0.0034
7	2	5	7	←	6	3	4	7	4683.3406	0.0018
7	2	5	7	←	6	3	4	6	4683.5842	0.0005
7	2	5	6	←	6	3	4	5	4683.7064	-0.0058
7	2	5	6	←	6	3	4	6	4683.9999	0.0012
8	5	3	8	←	7	6	2	7	4684.1920	0.0005
8	5	3	9	←	7	6	2	8	4685.0603	0.0043
8	5	3	7	←	7	6	2	6	4685.1792	0.0022
7	3	4	7	←	6	4	3	6	4749.7059	-0.0003
7	3	4	6	←	6	4	3	6	4750.1082	0.0063
7	3	4	6	←	6	4	3	5	4750.1709	0.0015
7	3	5	7	←	6	2	4	7	4763.3125	0.0010
7	3	5	8	←	6	2	4	7	4763.6325	-0.0122
7	3	5	6	←	6	2	4	6	4764.0432	-0.0001
5	5	1	4	←	4	4	0	4	4768.9649	-0.0021
5	5	1	6	←	4	4	0	5	4770.2236	0.0040
5	5	1	5	←	4	4	0	4	4770.3339	-0.0004
5	5	0	4	←	4	4	1	4	4782.6650	-0.0030
5	5	0	6	←	4	4	1	5	4783.9528	0.0135
5	5	0	5	←	4	4	1	4	4784.0193	-0.0089
5	5	0	5	←	4	4	1	5	4785.0697	0.0003
9	5	5	9	←	8	6	2	8	4852.0247	0.0035
6	4	3	5	←	5	3	2	4	4893.5739	0.0135
6	4	3	7	←	5	3	2	6	4893.6176	-0.0009
6	4	3	6	←	5	3	2	5	4893.9170	0.0032
8	1	7	9	←	7	2	6	8	4943.1862	-0.0085
8	2	7	9	←	7	1	6	8	4943.9707	-0.0095
9	6	3	9	←	8	7	2	8	5017.3064	0.0017

J'	K_a'	K_c'	F'	←	J''	K_a''	K_c''	F''	ν_{obs}	$\nu_{obs}-\nu_{calc}$
9	0	9	9	←	8	1	8	9	5170.5472	0.0000
9	1	9	10	←	8	0	8	9	5171.3635	-0.0083
8	2	6	8	←	7	3	5	8	5264.9151	0.0009
8	2	6	8	←	7	3	5	7	5265.2430	-0.0043
8	2	6	9	←	7	3	5	8	5265.3183	0.0050
8	3	6	9	←	7	2	5	8	5287.3962	0.0038
8	4	4	8	←	7	5	3	7	5311.7856	0.0018
8	4	4	9	←	7	5	3	8	5312.3221	-0.0011
8	4	4	7	←	7	5	3	6	5312.4007	0.0061
7	4	4	8	←	6	3	3	7	5326.5496	-0.0099
7	4	4	7	←	6	3	3	6	5326.7796	0.0023
10	7	3	10	←	9	8	2	9	5368.6048	-0.0034
5	2	3	6	←	4	1	4	5	5402.5424	-0.0013
5	2	3	5	←	4	1	4	4	5402.8673	0.0137
6	5	2	5	←	5	4	1	5	5427.8893	0.0023
6	5	2	7	←	5	4	1	6	5428.3036	0.0051
6	5	2	6	←	5	4	1	5	5428.5262	0.0009
6	5	2	6	←	5	4	1	6	5428.8636	0.0189
10	6	5	9	←	9	7	2	8	5489.2342	-0.0081
8	3	5	8	←	7	4	4	7	5492.2915	0.0022
8	3	5	9	←	7	4	4	8	5492.4644	-0.0117
9	1	8	9	←	8	2	7	9	5498.9929	0.0051
9	2	8	9	←	8	1	7	9	5499.1184	-0.0189
9	1	8	10	←	8	2	7	9	5499.5962	-0.0044
9	2	8	10	←	8	1	7	9	5499.7477	-0.0025
6	5	1	5	←	5	4	2	5	5536.6902	-0.0016
6	5	1	5	←	5	4	2	4	5537.1802	0.0020
6	5	1	6	←	5	4	2	5	5537.2867	-0.0003
5	4	2	4	←	4	1	3	3	5537.6065	0.0018
6	5	1	6	←	5	4	2	6	5537.6870	-0.0042
5	4	2	6	←	4	1	3	5	5537.7952	0.0012
5	4	2	5	←	4	1	3	4	5538.6162	0.0022
5	3	3	6	←	4	0	4	5	5574.7900	-0.0027
6	4	2	7	←	5	3	3	6	5606.5815	-0.0045
10	0	10	10	←	9	1	9	10	5726.8980	0.0102
10	1	10	10	←	9	0	9	9	5727.7186	0.0076
9	5	4	9	←	8	6	3	8	5741.7948	0.0049
9	5	4	10	←	8	6	3	9	5742.4271	0.0028
9	5	4	8	←	8	6	3	7	5742.5079	0.0085
8	4	5	9	←	7	3	4	8	5746.9641	0.0100
8	4	5	8	←	7	3	4	7	5747.0661	0.0032
6	6	1	6	←	5	5	0	6	5759.2768	0.0046
6	6	0	5	←	5	5	1	5	5760.9383	-0.0008
6	6	0	7	←	5	5	1	6	5762.2708	-0.0079
6	6	0	6	←	5	5	1	5	5762.3419	-0.0074
6	6	0	6	←	5	5	1	6	5763.4872	0.0018
9	2	7	8	←	8	3	6	7	5827.8880	-0.0068
9	3	7	8	←	8	2	6	7	5833.1928	-0.0043
7	5	3	8	←	6	4	2	7	5984.1087	-0.0145
7	5	3	7	←	6	4	2	6	5984.4071	0.0026
10	1	9	9	←	9	2	8	8	6055.8497	0.0015
10	6	4	10	←	9	7	3	9	6077.8959	0.0096
9	3	6	9	←	8	4	5	8	6127.6291	0.0027
9	3	6	8	←	8	4	5	7	6127.7193	-0.0009
9	4	5	9	←	8	5	4	8	6200.3421	0.0036
9	4	5	10	←	8	5	4	9	6200.6457	0.0109
9	4	6	8	←	8	3	5	7	6215.1151	-0.0051
11	0	11	10	←	10	1	10	9	6284.0734	0.0027
11	1	11	10	←	10	0	10	9	6284.0734	0.0027
7	5	2	6	←	6	4	3	5	6402.8054	-0.0105
8	5	4	9	←	7	4	3	8	6428.6009	-0.0116
8	5	4	8	←	7	4	3	7	6428.8773	0.0009
7	6	2	6	←	6	5	1	5	6445.5057	0.0053
7	6	2	7	←	6	5	1	6	6445.6657	-0.0015
7	6	2	7	←	6	5	1	7	6446.1726	-0.0040
7	6	1	6	←	6	5	2	6	6486.9519	0.0045
7	6	1	6	←	6	5	2	5	6487.5853	-0.0003

J′	*K*a′	*K*c′	*F*′	←	*J*″	*K*a″	*K*c″	*F*″	ν_{obs}	$\nu_{obs}-\nu_{calc}$
7	6	1	7	←	6	5	2	6	6487.6933	-0.0011
7	6	1	7	←	6	5	2	7	6488.2401	-0.0006
6	4	3	5	←	5	1	4	4	6511.6143	0.0009
6	4	3	5	←	5	1	4	4	6511.6143	0.0009
6	4	3	7	←	5	1	4	6	6511.7122	-0.0002
6	4	3	6	←	5	1	4	5	6512.2098	0.0003
11	1	10	10	←	10	2	9	9	6612.1190	0.0170
11	2	10	10	←	10	1	9	9	6612.1190	0.0122
6	2	4	5	←	5	1	5	4	6677.2795	-0.0043
6	2	4	7	←	5	1	5	6	6677.3694	-0.0033
6	2	4	6	←	5	1	5	5	6677.7516	0.0026
6	5	2	5	←	5	2	3	4	6738.1705	0.0003
6	5	2	7	←	5	2	3	6	6738.3388	0.0011
6	5	2	6	←	5	2	3	5	6739.2555	0.0021
6	3	4	5	←	5	0	5	4	6739.7665	-0.0096
6	3	4	7	←	5	0	5	6	6739.8903	0.0072
6	3	4	6	←	5	0	5	5	6740.3662	0.0007
7	7	1	6	←	6	6	0	6	6741.1513	0.0106
7	7	0	6	←	6	6	1	6	6742.3697	0.0140
10	5	5	10	←	9	6	4	9	6766.5662	0.0091
10	5	5	9	←	9	6	4	8	6766.9993	-0.0163
7	4	3	8	←	6	3	4	7	6779.7086	-0.0082
9	5	5	8	←	8	4	4	7	6810.4360	-0.0175
9	5	5	10	←	8	4	4	9	6810.4915	0.0113
9	5	5	9	←	8	4	4	8	6810.6731	0.0002
11	2	9	10	←	10	3	8	9	6941.2572	0.0022
11	3	9	10	←	10	2	8	9	6941.4954	0.0030
10	4	6	10	←	9	5	5	9	6942.7519	0.0041
10	4	6	9	←	9	5	5	8	6942.8959	-0.0030
8	6	3	9	←	7	5	2	8	7063.4432	0.0100
8	6	3	8	←	7	5	2	7	7063.6557	-0.0010
11	6	5	10	←	10	7	4	9	7189.4801	-0.0136
10	5	6	9	←	9	4	5	8	7204.6835	0.0073
10	5	6	10	←	9	4	5	9	7204.7822	-0.0013
8	6	2	9	←	7	5	3	8	7270.3630	-0.0064
8	6	2	8	←	7	5	3	7	7270.4285	-0.0070
8	6	2	8	←	7	5	3	8	7270.6171	-0.0037
11	3	8	10	←	10	4	7	9	7271.3971	-0.0032
11	4	8	10	←	10	3	7	9	7277.6615	0.0058
7	3	4	8	←	6	2	5	7	7420.8921	0.0085
7	3	4	7	←	6	2	5	6	7421.0159	-0.0018
8	7	2	9	←	7	6	1	8	7443.4054	-0.0054
8	7	2	8	←	7	6	1	7	7443.5199	-0.0044
8	7	1	9	←	7	6	2	8	7457.9788	-0.0049
8	7	1	8	←	7	6	2	7	7458.0739	-0.0061
8	5	3	8	←	7	4	4	7	7460.7359	-0.0035
8	5	3	9	←	7	4	4	8	7460.8081	0.0022
7	5	3	6	←	6	2	4	5	7540.9776	-0.0083
7	5	3	8	←	6	2	4	7	7541.0723	0.0003
7	5	3	7	←	6	2	4	6	7541.6062	-0.0012
9	6	4	10	←	8	5	3	9	7558.7654	-0.0012
9	6	4	9	←	8	5	3	8	7559.0257	-0.0020
11	4	7	10	←	10	5	6	9	7575.2930	0.0018
7	4	4	6	←	6	1	5	5	7610.8941	-0.0019
7	4	4	8	←	6	1	5	7	7610.9571	-0.0021
7	4	4	7	←	6	1	5	6	7611.3126	-0.0016
11	5	6	10	←	10	6	5	9	7662.1918	-0.0069
11	5	7	10	←	10	4	6	9	7665.0055	-0.0023
8	8	1	9	←	7	7	0	8	7725.8581	-0.0007
8	8	0	9	←	7	7	1	8	7726.1917	-0.0036
7	3	5	8	←	6	0	6	7	7938.8461	0.0019
7	3	5	7	←	6	0	6	6	7939.2711	0.0001
10	6	5	10	←	9	5	4	9	7942.9191	-0.0041

Table S2. Measured rotational transitions (ν_{obs}) of the ^{13}C (1,3) isotopic species of the Py-(Bz)$_2$ trimer and residuals (ν_{obs} - ν_{calc}) (frequencies in MHz).

J'	K_a'	K_c'	F'	←	J''	K_a''	K_c''	F''	ν_{obs}	ν_{obs}-ν_{calc}
4	0	4	5	←	3	1	3	4	2377.1616	0.0032
5	0	5	6	←	4	1	4	5	2935.1636	-0.0017
5	2	4	6	←	4	1	3	5	3297.2794	0.0007
6	0	6	6	←	5	1	5	5	3490.3194	0.0137
6	1	6	6	←	5	0	5	5	3490.6534	0.0121
7	0	7	8	←	6	1	6	7	4044.9568	0.0115
7	1	7	8	←	6	0	6	7	4045.0146	0.0124
7	1	6	8	←	6	2	5	7	4370.7836	-0.0049
7	2	6	8	←	6	1	5	7	4374.3887	-0.0133
8	0	8	9	←	7	1	7	8	4599.4612	0.0056
8	1	7	9	←	7	2	6	8	4926.2501	-0.0064
8	2	7	9	←	7	1	6	8	4926.9728	-0.0109
9	1	8	10	←	8	2	7	9	5480.7798	-0.0102
9	2	8	9	←	8	1	7	8	5480.8869	-0.0043
10	2	9	10	←	9	1	8	9	6035.1744	-0.0033
7	6	1	6	←	6	5	2	5	6458.1682	0.0013
5	1	5	4	←	4	0	4	3	2936.9987	0.0109
4	4	0	5	←	3	3	1	4	3796.3391	0.0036
9	3	7	10	←	8	2	6	9	5812.6977	-0.0075
11	2	9	10	←	10	3	8	9	6917.3136	0.0021
11	3	9	10	←	10	2	8	9	6917.5208	-0.0054
4	1	4	5	←	3	0	3	4	2386.4332	-0.0113
3	3	0	4	←	2	2	1	3	2852.1418	0.0013
5	1	4	5	←	4	2	3	4	3234.5130	0.0088
6	1	5	5	←	5	2	4	4	3810.9251	-0.0004
5	5	1	6	←	4	4	0	5	4746.6079	0.0051
5	5	1	5	←	4	4	0	4	4746.7238	0.0054
9	0	9	9	←	8	1	8	8	5153.9413	0.0046
9	1	9	9	←	8	0	8	8	5153.9413	0.0032
9	0	9	8	←	8	1	8	7	5153.9413	-0.0024
9	1	9	8	←	8	0	8	7	5153.9413	-0.0038
9	0	9	10	←	8	1	8	9	5153.9413	-0.0120
9	1	9	10	←	8	0	8	9	5153.9413	-0.0134
6	5	1	7	←	5	4	2	6	5513.4881	0.0029
10	0	10	10	←	9	1	9	9	5708.4386	0.0027
10	1	10	10	←	9	0	9	9	5708.4386	0.0024
10	0	10	9	←	9	1	9	8	5708.4386	-0.0031
10	1	10	9	←	9	0	9	8	5708.4386	-0.0034
10	0	10	11	←	9	1	9	10	5708.4386	-0.0110
10	1	10	11	←	9	0	9	10	5708.4386	-0.0113
11	0	11	11	←	10	1	10	10	6262.9389	0.0059
11	1	11	11	←	10	0	10	10	6262.9389	0.0059
11	0	11	10	←	10	1	10	9	6262.9389	0.0009
11	1	11	10	←	10	0	10	9	6262.9389	0.0009
11	0	11	12	←	10	1	10	11	6262.9389	-0.0057
11	1	11	12	←	10	0	10	11	6262.9389	-0.0057
10	2	8	9	←	9	3	7	8	6363.0705	-0.0059
10	3	8	9	←	9	2	7	8	6364.1320	-0.0068
7	6	1	7	←	6	5	2	6	6458.2666	-0.0081
10	3	7	11	←	9	4	6	10	6686.3549	-0.0073
10	4	7	9	←	9	3	6	8	6709.4925	0.0164
12	0	12	12	←	11	1	11	11	6817.4296	0.0029
12	1	12	12	←	11	0	11	11	6817.4296	0.0028
12	0	12	11	←	11	1	11	10	6817.4296	-0.0013
12	1	12	11	←	11	0	11	10	6817.4296	-0.0013
12	0	12	13	←	11	1	11	12	6817.4296	-0.0070
12	1	12	13	←	11	0	11	12	6817.4296	-0.0070
12	1	11	12	←	11	2	10	11	7143.9953	0.0203
12	2	11	12	←	11	1	10	11	7143.9953	0.0196
12	1	11	11	←	11	2	10	10	7143.9953	0.0014
12	2	11	11	←	11	1	10	10	7143.9953	0.0007
12	1	11	13	←	11	2	10	12	7143.9953	-0.0018
12	2	11	13	←	11	1	10	12	7143.9953	-0.0025

J'	K_a'	K_c'	F'	←	J''	K_a''	K_c''	F''	ν_{obs}	$\nu_{obs}-\nu_{calc}$
11	3	8	12	←	10	4	7	11	7246.1455	0.0004
11	4	8	10	←	10	3	7	9	7251.8939	0.0119
13	0	13	13	←	12	1	12	12	7371.9154	-0.0006
13	1	13	13	←	12	0	12	12	7371.9154	-0.0006
13	0	13	12	←	12	1	12	11	7371.9154	-0.0042
13	1	13	12	←	12	0	12	11	7371.9154	-0.0042
13	0	13	13	←	12	1	12	12	7371.9154	-0.0006
13	0	13	13	←	12	1	12	12	7371.9154	-0.0006
8	7	1	9	←	7	6	2	8	7423.1294	-0.0020
8	7	1	8	←	7	6	2	7	7423.2336	0.0060
12	2	10	13	←	11	3	9	12	7471.4508	0.0087
8	8	1	9	←	7	7	0	8	7687.4112	-0.0003
8	8	0	9	←	7	7	1	8	7687.7650	-0.0035
13	1	12	12	←	12	2	11	11	7698.4286	0.0054
13	2	12	12	←	12	1	11	11	7698.4286	0.0053
13	1	12	14	←	12	2	11	13	7698.4286	0.0024
13	2	12	14	←	12	1	11	13	7698.4286	0.0023
12	3	9	13	←	11	4	8	12	7800.8700	0.0013
14	0	14	14	←	13	1	13	13	7926.3978	-0.0023
14	1	14	14	←	13	0	13	13	7926.3978	-0.0023
14	0	14	14	←	13	1	13	13	7926.3978	-0.0023
14	1	14	14	←	13	0	13	13	7926.3978	-0.0023
14	0	14	14	←	13	1	13	13	7926.3978	-0.0023
14	0	14	14	←	13	1	13	13	7926.3978	-0.0023
14	0	14	14	←	13	1	13	13	7926.3978	-0.0023
14	0	14	14	←	13	1	13	13	7926.3978	-0.0023
3	3	1	4	←	2	2	0	3	2750.4085	-0.0001
3	3	1	3	←	2	2	0	2	2750.7069	0.0091
5	1	4	6	←	4	2	3	5	3234.6513	-0.0150
5	3	3	6	←	4	2	2	5	3809.0224	-0.0041
5	3	3	5	←	4	2	2	4	3809.2789	0.0031
6	1	5	6	←	5	2	4	5	3810.8419	-0.0012
6	2	4	6	←	5	3	3	5	4039.1523	0.0011
6	3	4	6	←	5	2	3	5	4263.8562	-0.0035
7	2	5	8	←	6	3	4	7	4668.9500	0.0046
8	2	6	9	←	7	3	5	8	5247.4823	0.0027
7	4	4	8	←	6	3	3	7	5300.6756	-0.0039
7	4	4	7	←	6	3	3	6	5300.9108	0.0146
6	5	2	7	←	5	4	1	6	5401.7919	0.0073
6	5	1	6	←	5	4	2	5	5513.5918	0.0034
6	4	2	7	←	5	3	3	6	5589.9295	0.0057
8	4	5	9	←	7	3	4	8	5721.2616	-0.0154
6	6	0	7	←	5	5	1	6	5733.9359	-0.0043
6	6	0	6	←	5	5	1	5	5734.0018	-0.0089
6	6	1	6	←	5	5	0	5	5729.6376	0.0019
7	5	3	8	←	6	4	2	7	5953.8204	-0.0056
7	5	3	7	←	6	4	2	6	5954.1245	0.0153
9	4	6	10	←	8	3	5	9	6190.4882	-0.0044
7	6	2	8	←	6	5	1	7	6414.5269	-0.0071
7	6	2	7	←	6	5	1	6	6414.6882	-0.0041
7	7	0	8	←	6	6	1	7	6710.3414	-0.0057
8	6	3	9	←	7	5	2	8	7028.3920	0.0091
8	6	3	8	←	7	5	2	7	7028.6086	-0.0007
8	6	2	9	←	7	5	3	8	7241.8408	-0.0025
8	7	2	7	←	7	6	1	6	7407.8612	0.0048
8	7	2	8	←	7	6	1	7	7407.9639	-0.0093

Table S3. Measured rotational transitions (ν_{obs}) of the ^{13}C (4,5) isotopic species of the Py-(Bz)$_2$ trimer and residuals (ν_{obs} - ν_{calc}) (frequencies in MHz).

J'	K_a'	K_c'	F'	←	J''	K_a''	K_c''	F''	ν_{obs}	ν_{obs}- ν_{calc}
5	1	5	6	←	4	0	4	5	2927.5747	-0.0096
5	2	4	6	←	4	1	3	5	3284.7172	0.0097
6	0	6	7	←	5	1	5	6	3479.1968	-0.0014
6	1	6	7	←	5	0	5	6	3479.4765	0.0039
6	1	5	7	←	5	2	4	6	3801.0374	-0.0043
6	2	5	7	←	5	1	4	6	3814.9591	-0.0117
7	1	7	8	←	6	0	6	7	4031.9566	0.0116
7	1	6	7	←	6	2	5	6	4358.1820	-0.0069
7	1	6	8	←	6	2	5	7	4358.2344	-0.0103
7	2	6	7	←	6	1	5	6	4361.1814	0.0130
8	0	8	8	←	7	1	7	7	4584.5099	0.0065
5	5	0	6	←	4	4	1	5	4736.0702	0.0181
8	2	7	7	←	7	1	6	6	4912.1908	-0.0056
9	1	8	8	←	8	2	7	7	5464.2293	-0.0048
9	2	8	10	←	8	1	7	9	5464.3372	-0.0050
9	2	7	8	←	8	3	6	7	5791.7524	-0.0079
9	3	7	8	←	8	2	6	7	5795.6551	-0.0072
10	2	9	9	←	9	1	8	8	6016.7556	-0.0068
10	2	8	9	←	9	3	7	8	6344.8838	-0.0003
10	3	8	9	←	9	2	7	8	6345.6902	-0.0045
7	6	1	8	←	6	5	2	7	6430.5294	-0.0060
7	6	1	7	←	6	5	2	6	6430.6398	0.0016
10	4	7	9	←	9	3	6	8	6688.4178	0.0123
11	2	9	10	←	10	3	8	9	6897.1849	-0.0118
11	3	8	10	←	10	4	7	9	7226.4933	-0.0105
5	1	4	5	←	4	2	3	4	3228.7500	-0.0078
5	1	4	4	←	4	2	3	3	3228.9261	-0.0070
4	4	1	5	←	3	3	0	4	3733.8925	0.0004
4	4	1	4	←	3	3	0	3	3734.0923	0.0100
7	2	5	7	←	6	3	4	6	4660.9552	-0.0002
7	2	5	6	←	6	3	4	5	4661.0625	-0.0145
8	2	6	8	←	7	3	5	7	5234.3763	0.0003
8	2	6	7	←	7	3	5	6	5234.4497	0.0074
8	3	6	9	←	7	2	5	8	5251.5910	0.0192
6	5	2	6	←	5	4	1	5	5371.8750	0.0051
6	5	1	7	←	5	4	2	6	5494.4222	0.0063
6	5	1	6	←	5	4	2	5	5494.5098	-0.0024
9	3	6	8	←	8	4	5	7	6097.7490	0.0054
11	0	11	10	←	10	1	10	9	6242.3579	-0.0006
11	1	11	10	←	10	0	10	9	6242.3579	-0.0006
7	6	2	6	←	6	5	1	5	6380.8840	0.0019
7	6	2	7	←	6	5	1	6	6381.0669	0.0100
11	1	10	10	←	10	2	9	9	6569.2828	0.0172
11	2	10	10	←	10	1	9	9	6569.2828	0.0143
10	3	7	9	←	9	4	6	8	6669.9385	-0.0036
11	3	9	10	←	10	2	8	9	6897.3658	0.0114
8	7	2	9	←	7	6	1	8	7370.1639	-0.0018
8	7	2	8	←	7	6	1	7	7370.2767	-0.0060
8	7	1	7	←	7	6	2	6	7388.1328	-0.0016
8	7	1	8	←	7	6	2	7	7388.2197	-0.0097
11	4	7	10	←	10	5	6	9	7537.3314	0.0067
11	5	7	10	←	10	4	6	9	7604.8213	0.0129
8	8	1	9	←	7	7	0	8	7644.7253	-0.0021
4	4	0	5	←	3	3	1	4	3778.2526	0.0015
5	3	3	6	←	4	2	2	5	3787.5925	0.0027
5	3	3	5	←	4	2	2	4	3787.8419	0.0047
5	5	1	6	←	4	4	0	5	4720.3541	0.0084
5	5	0	5	←	4	4	1	4	4736.1290	-0.0103
6	4	3	6	←	5	3	2	5	4839.0677	0.0085
6	5	2	7	←	5	4	1	6	5371.6268	-0.0067
8	3	5	8	←	7	4	4	7	5478.8816	-0.0070
10	0	10	10	←	9	1	9	9	5689.7464	0.0076
10	1	10	10	←	9	0	9	9	5689.7464	0.0075

J'	K_a'	K_c'	F'	←	J''	K_a''	K_c''	F''	ν_{obs}	$\nu_{obs}-\nu_{calc}$
10	0	10	9	←	9	1	9	8	5689.7464	0.0016
10	1	10	9	←	9	0	9	8	5689.7464	0.0015
8	4	5	8	←	7	3	4	7	5691.2995	0.0094
6	6	1	7	←	5	5	0	6	5698.0305	-0.0086
6	6	1	6	←	5	5	0	5	5698.1180	-0.0006
6	6	0	7	←	5	5	1	6	5703.0667	-0.0052
6	6	0	6	←	5	5	1	5	5703.1347	-0.0075
9	4	6	10	←	8	3	5	9	6166.3832	0.0027
8	5	4	8	←	7	4	3	7	6350.7049	0.0041
7	5	2	7	←	6	4	3	6	6376.4685	0.0053
7	7	0	7	←	6	6	1	6	6673.6053	0.0011
8	6	3	8	←	7	5	2	7	6986.6125	0.0008
8	6	2	9	←	7	5	3	8	7224.9773	0.0074
10	6	5	10	←	9	5	4	9	7838.9560	-0.0069
4	0	4	5	←	3	1	3	4	2370.2497	-0.0092
3	3	0	4	←	2	2	1	3	2840.7994	-0.0024
7	5	3	7	←	6	4	2	6	5915.9399	-0.0021
7	7	1	7	←	6	6	0	6	6672.0814	-0.0105
12	0	12	12	←	11	1	11	11	6794.9682	0.0031
12	1	12	12	←	11	0	11	11	6794.9682	0.0031
12	0	12	11	←	11	1	11	10	6794.9682	-0.0012
12	1	12	11	←	11	0	11	10	6794.9682	-0.0012
12	0	12	13	←	11	1	11	12	6794.9682	-0.0068
12	1	12	13	←	11	0	11	12	6794.9682	-0.0068
12	1	11	13	←	11	2	10	12	7121.8128	0.0047
12	2	11	13	←	11	1	10	12	7121.8128	0.0042
13	0	13	13	←	12	1	12	12	7347.5735	0.0009
13	1	13	13	←	12	0	12	12	7347.5735	0.0009
13	0	13	12	←	12	1	12	11	7347.5735	-0.0027
13	1	13	12	←	12	0	12	11	7347.5735	-0.0027
13	0	13	14	←	12	1	12	13	7347.5735	-0.0076
13	1	13	14	←	12	0	12	13	7347.5735	-0.0076
12	2	10	13	←	11	3	9	12	7449.4516	-0.0069
13	2	12	13	←	12	1	11	12	7674.3377	-0.0019
12	3	9	13	←	11	4	8	12	7779.0394	0.0123
12	4	9	11	←	11	3	8	10	7779.9865	-0.0021
14	0	14	14	←	13	1	13	13	7900.1722	-0.0028
14	1	14	14	←	13	0	13	13	7900.1722	-0.0028
14	0	14	13	←	13	1	13	12	7900.1722	-0.0060
14	1	14	13	←	13	0	13	12	7900.1722	-0.0060
14	0	14	15	←	13	1	13	14	7900.1722	-0.0103
14	1	14	15	←	13	0	13	14	7900.1722	-0.0103

Table S4. Measured rotational transitions (ν_{obs}) of the ^{13}C (6,10) isotopic species of the Py-(Bz)$_2$ timer and residuals (ν_{obs} - ν_{calc}) (frequencies in MHz).

J'	K_a'	K_c'	F'	←	J''	K_a''	K_c''	F''	ν_{obs}	ν_{obs}- ν_{calc}
4	0	4	5	←	3	1	3	4	2378.9297	0.0025
4	1	4	4	←	3	0	3	3	2388.0505	0.0112
3	3	1	4	←	2	2	0	3	2750.2758	0.0016
3	3	1	3	←	2	2	0	2	2750.5644	0.0007
5	0	5	6	←	4	1	4	5	2937.3254	0.0094
5	1	5	6	←	4	0	4	5	2939.1203	-0.0112
5	1	4	5	←	4	2	3	4	3237.0265	-0.0046
5	1	4	6	←	4	2	3	5	3237.1937	0.0009
5	2	4	6	←	4	1	3	5	3298.9548	0.0125
6	0	6	7	←	5	1	5	6	3492.9081	0.0009
6	1	6	7	←	5	0	5	6	3493.2309	-0.0028
4	4	0	5	←	3	3	1	4	3796.4856	-0.0064
4	4	0	4	←	3	3	1	3	3796.5972	-0.0014
6	1	5	6	←	5	2	4	5	3813.5296	0.0137
6	1	5	7	←	5	2	4	6	3813.6015	0.0032
6	2	5	7	←	5	1	4	6	3829.5630	-0.0103
6	2	4	7	←	5	3	3	6	4043.4260	0.0084
7	1	7	7	←	6	0	6	6	4047.9642	-0.0055
5	4	2	6	←	4	3	1	5	4364.3923	0.0062
5	4	2	5	←	4	3	1	4	4364.6823	-0.0130
7	1	6	8	←	6	2	5	7	4373.7899	-0.0017
7	2	6	8	←	6	1	5	7	4377.3143	-0.0071
8	0	8	8	←	7	1	7	7	4602.8772	0.0176
8	0	8	7	←	7	1	7	6	4602.8772	0.0088
8	1	8	8	←	7	0	7	7	4602.8772	0.0086
8	1	8	7	←	7	0	7	6	4602.8772	0.0000
8	0	8	9	←	7	1	7	8	4602.8772	-0.0029
8	1	8	9	←	7	0	7	8	4602.8772	-0.0118
5	4	1	6	←	4	3	2	5	4609.8915	0.0070
7	2	5	8	←	6	3	4	7	4672.4977	0.0075
5	5	1	6	←	4	4	0	5	4746.3764	0.0056
5	5	1	5	←	4	4	0	4	4746.4969	0.0103
7	3	5	8	←	6	2	4	7	4747.1643	-0.0098
5	5	0	6	←	4	4	1	5	4760.6833	0.0089
5	5	0	5	←	4	4	1	4	4760.7605	-0.0023
8	1	7	9	←	7	2	6	8	4929.6484	-0.0148
8	2	7	9	←	7	1	6	8	4930.3659	-0.0044
9	0	9	9	←	8	1	8	8	5157.8029	0.0110
9	1	9	9	←	8	0	8	8	5157.8029	0.0096
9	0	9	8	←	8	1	8	7	5157.8029	0.0039
9	1	9	8	←	8	0	8	7	5157.8029	0.0025
9	0	9	10	←	8	1	8	9	5157.8029	-0.0056
9	1	9	10	←	8	0	8	9	5157.8029	-0.0070
8	2	6	8	←	7	3	5	7	5250.9731	0.0011
8	2	6	7	←	7	3	5	6	5251.0462	0.0068
8	3	6	9	←	7	2	5	8	5271.2958	0.0013
7	4	4	7	←	6	3	3	6	5301.2172	-0.0056
8	3	5	8	←	7	4	4	7	5483.3745	0.0116
9	1	8	10	←	8	2	7	9	5484.6208	-0.0013
9	2	8	10	←	8	1	7	9	5484.7521	-0.0024
6	5	1	7	←	5	4	2	6	5514.7048	-0.0005
10	0	10	10	←	9	1	9	9	5712.7310	0.0088
10	1	10	10	←	9	0	9	9	5712.7310	0.0086
10	0	10	9	←	9	1	9	8	5712.7310	0.0029
10	1	10	9	←	9	0	9	8	5712.7310	0.0027
10	0	10	11	←	9	1	9	10	5712.7310	-0.0049
10	1	10	11	←	9	0	9	10	5712.7310	-0.0051
8	4	5	8	←	7	3	4	7	5722.7121	-0.0175
6	6	1	6	←	5	5	0	5	5729.3412	-0.0069
6	6	0	7	←	5	5	1	6	5733.7210	-0.0045
6	6	0	6	←	5	5	1	5	5733.7800	-0.0160
9	2	7	10	←	8	3	6	9	5811.6250	-0.0090
9	3	7	10	←	8	2	6	9	5816.4099	-0.0060

J'	K_a'	K_c'	F'	←	J''	K_a''	K_c''	F''	ν_{obs}	$\nu_{obs}-\nu_{calc}$
7	5	3	8	←	6	4	2	7	5953.4660	-0.0044
7	5	3	7	←	6	4	2	6	5953.7493	-0.0051
10	1	9	11	←	9	2	8	10	6039.4406	-0.0057
9	3	6	10	←	8	4	5	9	6112.7118	0.0019
9	4	6	10	←	8	3	5	9	6193.2377	-0.0017
11	0	11	11	←	10	1	10	10	6267.6551	0.0044
11	1	11	11	←	10	0	10	10	6267.6551	0.0043
11	0	11	10	←	10	1	10	9	6267.6551	-0.0005
11	1	11	10	←	10	0	10	9	6267.6551	-0.0006
11	0	11	12	←	10	1	10	11	6267.6551	-0.0072
11	1	11	12	←	10	0	10	11	6267.6551	-0.0072
10	2	8	11	←	9	3	7	10	6367.3104	-0.0052
10	3	8	11	←	9	2	7	10	6368.3458	0.0013
7	5	2	8	←	6	4	3	7	6384.4557	-0.0024
8	5	4	9	←	7	4	3	8	6394.8373	0.0079
7	6	2	8	←	6	5	1	7	6414.6377	-0.0019
7	6	2	7	←	6	5	1	6	6414.7892	-0.0092
7	6	1	8	←	6	5	2	7	6458.9505	-0.0019
7	6	1	7	←	6	5	2	6	6459.0539	-0.0041
11	1	10	12	←	10	2	9	11	6594.2957	0.0196
10	3	7	9	←	9	4	6	8	6690.7746	-0.0063
7	7	1	8	←	6	6	0	7	6708.6851	-0.0115
7	7	0	7	←	6	6	1	6	6710.0404	-0.0158
10	4	7	11	←	9	3	6	10	6713.2936	0.0114
12	0	12	12	←	11	1	11	11	6822.5795	0.0031
12	1	12	12	←	11	0	11	11	6822.5795	0.0031
12	0	12	11	←	11	1	11	10	6822.5795	-0.0011
12	1	12	11	←	11	0	11	10	6822.5795	-0.0011
12	0	12	13	←	11	1	11	12	6822.5795	-0.0067
12	1	12	13	←	11	0	11	12	6822.5795	-0.0067
11	2	9	12	←	10	3	8	11	6921.9739	-0.0027
11	3	9	12	←	10	2	8	11	6922.1806	-0.0031
8	6	3	9	←	7	5	2	8	7028.1668	0.0124
8	6	3	8	←	7	5	2	7	7028.3921	0.0101
12	1	11	11	←	11	2	10	10	7149.1276	0.0060
12	2	11	11	←	11	1	10	10	7149.1276	0.0053
12	1	11	13	←	11	2	10	12	7149.1276	0.0027
12	2	11	13	←	11	1	10	12	7149.1276	0.0020
10	5	6	11	←	9	4	5	10	7173.6380	0.0104
10	5	6	10	←	9	4	5	9	7173.7187	0.0014
8	6	2	9	←	7	5	3	8	7244.4831	-0.0031
11	3	8	10	←	10	4	7	9	7250.8268	0.0092
11	4	8	12	←	10	3	7	11	7256.3701	-0.0048
13	0	13	13	←	12	1	12	12	7377.4958	-0.0024
13	1	13	13	←	12	0	12	12	7377.4958	-0.0024
13	0	13	12	←	12	1	12	11	7377.4958	-0.0060
13	1	13	12	←	12	0	12	11	7377.4958	-0.0060
13	0	13	14	←	12	1	12	13	7377.4958	-0.0109
13	1	13	14	←	12	0	12	13	7377.4958	-0.0109
8	7	2	9	←	7	6	1	8	7408.0391	-0.0087
8	7	1	9	←	7	6	2	8	7423.6114	-0.0090
8	7	1	8	←	7	6	2	7	7423.7337	0.0173
12	2	10	13	←	11	3	9	12	7476.5470	0.0074
9	6	4	10	←	8	5	3	9	7517.9759	0.0148
9	6	4	9	←	8	5	3	8	7518.2383	0.0133
11	4	7	12	←	10	5	6	11	7556.2740	0.0051
11	5	7	12	←	10	4	6	11	7637.7785	-0.0039
8	8	1	9	←	7	7	0	8	7686.9351	-0.0039
8	8	0	9	←	7	7	1	8	7687.3187	0.0139
13	1	12	13	←	12	2	11	12	7703.9789	0.0114
13	2	12	13	←	12	1	11	12	7703.9789	0.0112
13	1	12	12	←	12	2	11	11	7703.9789	-0.0049
13	2	12	12	←	12	1	11	11	7703.9789	-0.0050
13	1	12	14	←	12	2	11	13	7703.9789	-0.0079
13	2	12	14	←	12	1	11	13	7703.9789	-0.0080
14	0	14	14	←	13	1	13	13	7932.4087	-0.0067
14	1	14	14	←	13	0	13	13	7932.4087	-0.0067

J′	***K*a′**	***K*c′**	***F′***	←	***J″***	***K*a″**	***K*c″**	***F″***	ν_{obs}	$\nu_{obs}-\nu_{calc}$
14	0	14	13	←	13	1	13	12	7932.4087	-0.0099
14	1	14	13	←	13	0	13	12	7932.4087	-0.0099
14	0	14	15	←	13	1	13	14	7932.4087	-0.0142
14	1	14	15	←	13	0	13	14	7932.4087	-0.0142

Table S5. Measured rotational transitions (ν_{obs}) of the ^{13}C (7,9) isotopic species of the Py-(Bz)$_2$ trimer and residuals (ν_{obs} - ν_{calc}) (frequencies in MHz).

J′	*K*a′	*K*c′	*F′*	←	*J″*	*K*a″	*K*c″	*F″*	ν_{obs}	ν_{obs}-ν_{calc}
4	0	4	5	←	3	1	3	4	2373.0090	0.0014
4	1	4	5	←	3	0	3	4	2382.6166	-0.0131
4	1	3	5	←	3	2	2	4	2605.1442	-0.0104
5	0	5	6	←	4	1	4	5	2930.2007	0.0065
5	1	5	6	←	4	0	4	5	2932.1158	-0.0188
5	2	4	6	←	4	1	3	5	3293.0170	0.0117
4	3	2	5	←	3	2	1	4	3318.1276	0.0126
6	1	6	6	←	5	0	5	5	3484.7923	0.0090
6	1	6	7	←	5	0	5	6	3484.8198	0.0037
4	4	1	5	←	3	3	0	4	3753.9160	0.0114
4	4	1	4	←	3	3	0	3	3754.0864	-0.0026
4	4	0	5	←	3	3	1	4	3794.1158	0.0028
4	4	0	4	←	3	3	1	3	3794.2139	-0.0070
6	1	5	5	←	5	2	4	4	3804.2601	0.0025
6	2	5	7	←	5	1	4	6	3821.2943	-0.0113
6	2	4	7	←	5	3	3	6	4028.9051	0.0141
7	0	7	7	←	6	1	6	6	4038.0995	-0.0093
5	4	2	6	←	4	3	1	5	4362.9443	0.0041
7	1	6	8	←	6	2	5	7	4363.4400	0.0050
7	2	6	8	←	6	1	5	7	4367.2477	-0.0180
5	4	1	6	←	4	3	2	5	4601.6729	-0.0178
7	2	5	7	←	6	3	4	6	4659.5646	-0.0014
7	2	5	6	←	6	3	4	5	4659.6977	0.0031
5	5	1	6	←	4	4	0	5	4744.9186	0.0076
5	5	1	5	←	4	4	0	4	4745.0218	-0.0040
5	5	0	6	←	4	4	1	5	4758.5754	0.0154
8	1	7	9	←	7	2	6	8	4918.0330	-0.0003
8	2	7	9	←	7	1	6	8	4918.8112	-0.0023
9	0	9	9	←	8	1	8	8	5145.2572	0.0081
9	1	9	9	←	8	0	8	8	5145.2572	0.0065
9	0	9	8	←	8	1	8	7	5145.2572	0.0010
9	1	9	8	←	8	0	8	7	5145.2572	-0.0005
9	0	9	10	←	8	1	8	9	5145.2572	-0.0084
9	1	9	10	←	8	0	8	9	5145.2572	-0.0101
8	2	6	9	←	7	3	5	8	5238.3449	0.0058
8	3	6	9	←	7	2	5	8	5260.2797	0.0052
6	5	2	7	←	5	4	1	6	5399.6181	0.0092
9	1	8	10	←	8	2	7	9	5471.6495	0.0078
9	2	8	10	←	8	1	7	9	5471.7928	0.0024
6	5	1	5	←	5	4	2	4	5507.9313	0.0101
6	4	2	7	←	5	3	3	6	5577.0452	0.0005
6	6	1	6	←	5	5	0	5	5727.5705	-0.0103
6	6	0	6	←	5	5	1	5	5731.7642	-0.0027
9	2	7	8	←	8	3	6	7	5798.0645	-0.0182
9	3	7	8	←	8	2	6	7	5803.3394	-0.0101
7	5	3	7	←	6	4	2	6	5952.8914	-0.0007
10	1	9	9	←	9	2	8	8	6025.0965	0.0034
9	3	6	9	←	8	4	5	8	6096.1581	0.0095
11	0	11	11	←	10	1	10	10	6252.3728	0.0047
10	2	8	11	←	9	3	7	10	6352.5319	0.0001
10	3	8	11	←	9	2	7	10	6353.6798	-0.0046
7	5	2	8	←	6	4	3	7	6369.0794	-0.0091
7	6	2	8	←	6	5	1	7	6411.4106	0.0000
7	6	2	7	←	6	5	1	6	6411.5678	0.0004
7	6	1	8	←	6	5	2	7	6453.2761	0.0010
7	6	1	7	←	6	5	2	6	6453.3916	0.0097
11	1	10	12	←	10	2	9	11	6578.5657	0.0111
11	2	10	12	←	10	1	9	11	6578.5657	0.0064
10	3	7	11	←	9	4	6	10	6674.8163	0.0001
7	7	1	7	←	6	6	0	6	6706.7813	-0.0098
7	7	0	8	←	6	6	1	7	6707.9406	-0.0035
7	7	1	7	←	6	6	0	7	6707.9932	-0.0045
7	7	0	7	←	6	6	1	6	6707.9948	-0.0049

J'	K_a'	K_c'	F'	←	J''	K_a''	K_c''	F''	ν_{obs}	$\nu_{obs}-\nu_{calc}$
12	0	12	12	←	11	1	11	11	6805.9257	0.0022
12	1	12	12	←	11	0	11	11	6805.9257	0.0022
12	0	12	11	←	11	1	11	10	6805.9257	-0.0019
12	1	12	11	←	11	0	11	10	6805.9257	-0.0019
12	0	12	13	←	11	1	11	12	6805.9257	-0.0076
12	1	12	13	←	11	0	11	12	6805.9257	-0.0076
11	2	9	11	←	10	3	8	10	6905.8100	-0.0006
10	4	6	10	←	9	5	5	9	6907.0724	0.0015
10	5	6	11	←	9	4	5	10	7167.3294	-0.0090
10	5	6	10	←	9	4	5	9	7167.4455	0.0128
8	6	2	9	←	7	5	3	8	7232.0425	0.0081
11	3	8	12	←	10	4	7	11	7234.1221	-0.0029
11	4	8	12	←	10	3	7	11	7240.3548	0.0163
13	0	13	13	←	12	1	12	12	7359.4755	0.0006
13	1	13	13	←	12	0	12	12	7359.4755	0.0006
13	0	13	12	←	12	1	12	11	7359.4755	-0.0030
13	1	13	12	←	12	0	12	11	7359.4755	-0.0030
13	0	13	14	←	12	1	12	13	7359.4755	-0.0079
13	1	13	14	←	12	0	12	13	7359.4755	-0.0079
8	7	2	9	←	7	6	1	8	7404.0049	-0.0018
8	7	2	8	←	7	6	1	7	7404.1223	0.0020
8	7	1	9	←	7	6	2	8	7418.5051	-0.0031
8	7	1	8	←	7	6	2	7	7418.6046	0.0000
12	3	10	13	←	11	2	9	12	7459.0723	-0.0069
11	4	7	11	←	10	5	6	10	7536.2580	-0.0027
13	1	12	13	←	12	2	11	13	7684.8243	0.0132
13	2	12	13	←	12	1	11	13	7684.8243	0.0131
8	8	0	8	←	7	7	1	7	7685.2094	-0.0039
12	3	9	11	←	11	4	8	10	7788.0245	-0.0042
12	4	9	11	←	11	3	8	10	7789.4606	0.0003
14	0	14	14	←	13	1	13	13	7913.0168	-0.0046
14	1	14	14	←	13	0	13	13	7913.0168	-0.0046
14	0	14	13	←	13	1	13	12	7913.0168	-0.0078
14	1	14	13	←	13	0	13	12	7913.0168	-0.0078
14	0	14	15	←	13	1	13	14	7913.0168	-0.0120
14	1	14	15	←	13	0	13	14	7913.0168	-0.0120

Table S6. Measured rotational transitions (ν_{obs}) of the ^{13}C (8) isotopic species of the Py-(Bz)$_2$ timer and residuals (ν_{obs} - ν_{calc}) (frequencies in MHz).

J'	K_a'	K_c'	F'	←	J''	K_a''	K_c''	F''	ν_{obs}	ν_{obs}- ν_{calc}
5	0	5	4	←	4	1	4	3	2924.9286	0.0095
5	1	5	6	←	4	0	4	5	2926.9799	-0.0017
7	2	6	8	←	6	1	5	7	4360.7272	-0.0028
8	1	7	9	←	7	2	6	8	4910.2162	-0.0087
9	0	9	9	←	8	1	8	8	5135.5973	0.0160
9	1	9	9	←	8	0	8	8	5135.5973	0.0142
9	0	9	8	←	8	1	8	7	5135.5973	0.0089
9	1	9	8	←	8	0	8	7	5135.5973	0.0072
9	0	9	10	←	8	1	8	9	5135.5973	-0.0006
9	1	9	10	←	8	0	8	9	5135.5973	-0.0023
9	2	8	10	←	8	1	7	9	5462.8921	-0.0017
10	0	10	10	←	9	1	9	9	5688.0390	0.0117
10	1	10	10	←	9	0	9	9	5688.0390	0.0114
10	0	10	9	←	9	1	9	8	5688.0390	0.0059
10	1	10	9	←	9	0	9	8	5688.0390	0.0056
10	0	10	11	←	9	1	9	10	5688.0390	-0.0020
10	1	10	11	←	9	0	9	10	5688.0390	-0.0022
10	1	9	11	←	9	2	8	10	6015.0611	-0.0129
11	0	11	11	←	10	1	10	10	6240.4785	0.0067
11	1	11	11	←	10	0	10	10	6240.4785	0.0067
11	0	11	10	←	10	1	10	9	6240.4785	0.0018
11	1	11	10	←	10	0	10	9	6240.4785	0.0017
11	0	11	12	←	10	1	10	11	6240.4785	-0.0048
11	1	11	12	←	10	0	10	11	6240.4785	-0.0049
10	3	8	11	←	9	2	7	10	6344.5259	0.0063
7	5	2	8	←	6	4	3	7	6365.5963	0.0006
11	1	10	12	←	10	2	9	11	6567.4212	0.0052
11	2	10	12	←	10	1	9	11	6567.4212	0.0000
12	0	12	12	←	11	1	11	11	6792.9194	0.0055
12	1	12	12	←	11	0	11	11	6792.9194	0.0055
12	0	12	11	←	11	1	11	10	6792.9194	0.0013
12	1	12	11	←	11	0	11	10	6792.9194	0.0013
12	0	12	13	←	11	1	11	12	6792.9194	-0.0043
12	1	12	13	←	11	0	11	12	6792.9194	-0.0043
11	2	9	12	←	10	3	8	11	6895.4793	-0.0082
12	1	11	11	←	11	2	10	10	7119.7801	0.0059
12	2	11	11	←	11	1	10	10	7119.7801	0.0050
12	1	11	13	←	11	2	10	12	7119.7801	0.0026
12	2	11	13	←	11	1	10	12	7119.7801	0.0017
13	0	13	13	←	12	1	12	12	7345.3545	0.0019
13	1	13	13	←	12	0	12	12	7345.3545	0.0019
13	0	13	12	←	12	1	12	11	7345.3545	-0.0017
13	1	13	12	←	12	0	12	11	7345.3545	-0.0017
13	0	13	14	←	12	1	12	13	7345.3545	-0.0066
13	1	13	14	←	12	0	12	13	7345.3545	-0.0066
13	1	12	13	←	12	2	11	12	7672.1413	0.0071
13	2	12	13	←	12	1	11	12	7672.1413	0.0069
13	1	12	12	←	12	2	11	11	7672.1413	-0.0090
13	2	12	12	←	12	1	11	11	7672.1413	-0.0092
13	1	12	14	←	12	2	11	13	7672.1413	-0.0120
13	2	12	14	←	12	1	11	13	7672.1413	-0.0122
12	3	9	13	←	11	4	8	12	7777.3486	0.0007
12	4	9	13	←	11	3	8	12	7778.9209	0.0068
14	0	14	14	←	13	1	13	13	7897.7920	0.0048
14	1	14	14	←	13	0	13	13	7897.7920	0.0048
14	0	14	13	←	13	1	13	12	7897.7920	0.0017
14	1	14	13	←	13	0	13	12	7897.7920	0.0017
14	0	14	15	←	13	1	13	14	7897.7920	-0.0025
14	1	14	15	←	13	0	13	14	7897.7920	-0.0025

Table S7. Measured rotational transitions (ν_{obs}) of the ^{13}C (11) isotopic species of the Py-(Bz)$_2$ trimer and residuals (ν_{obs} - ν_{calc}) (frequencies in MHz).

J′	*K*a′	*K*c′	*F*′	←	*J*″	*K*a″	*K*c″	*F*″	ν_{obs}	ν_{obs}-ν_{calc}
4	0	4	5	←	3	1	3	4	2380.5650	-0.0010
4	1	4	5	←	3	0	3	4	2389.6738	0.0117
3	3	1	4	←	2	2	0	3	2754.0994	0.0064
3	3	1	3	←	2	2	0	2	2754.3746	-0.0081
3	3	0	4	←	2	2	1	3	2857.0910	0.0121
3	3	0	3	←	2	2	1	2	2857.2060	-0.0044
5	0	5	6	←	4	1	4	5	2939.1882	0.0000
5	1	5	6	←	4	0	4	5	2940.9700	-0.0168
5	2	4	6	←	4	1	3	5	3301.8368	0.0102
6	0	6	5	←	5	1	5	4	3495.0194	0.0130
6	0	6	7	←	5	1	5	6	3495.0194	-0.0065
6	1	6	5	←	5	0	5	4	3495.3346	0.0058
4	4	1	5	←	3	3	0	4	3760.3461	0.0118
4	4	1	4	←	3	3	0	3	3760.5233	0.0023
4	4	0	5	←	3	3	1	4	3802.2969	0.0008
4	4	0	4	←	3	3	1	3	3802.3976	-0.0046
5	3	3	6	←	4	2	2	5	3813.5273	0.0031
6	1	5	5	←	5	2	4	4	3816.9789	0.0001
6	2	5	7	←	5	1	4	6	3832.7903	-0.0191
7	0	7	8	←	6	1	6	7	4050.3250	0.0148
5	4	2	4	←	4	3	1	3	4369.9755	-0.0061
5	4	2	6	←	4	3	1	5	4370.0443	0.0043
5	4	2	5	←	4	3	1	4	4370.3495	-0.0002
7	1	6	8	←	6	2	5	7	4377.3750	-0.0021
7	2	6	8	←	6	1	5	7	4380.8449	-0.0188
5	4	1	6	←	4	3	2	5	4617.6826	-0.0008
7	2	5	7	←	6	3	4	6	4677.4306	-0.0055
7	2	5	8	←	6	3	4	7	4677.5571	0.0058
5	5	1	6	←	4	4	0	5	4753.3367	0.0036
5	5	1	5	←	4	4	0	4	4753.4509	0.0017
5	5	0	6	←	4	4	1	5	4767.8337	0.0194
8	1	7	9	←	7	2	6	8	4933.4792	-0.0075
8	2	7	9	←	7	1	6	8	4934.1774	-0.0054
9	0	9	8	←	8	1	8	7	5160.6889	0.0157
9	1	9	8	←	8	0	8	7	5160.6889	0.0144
9	0	9	10	←	8	1	8	9	5160.6889	0.0062
9	1	9	10	←	8	0	8	9	5160.6889	0.0048
8	2	6	8	←	7	3	5	7	5256.0895	-0.0008
8	2	6	9	←	7	3	5	8	5256.1603	0.0048
8	3	6	9	←	7	2	5	8	5276.1788	0.0057
6	5	2	7	←	5	4	1	6	5409.1719	0.0174
6	5	2	6	←	5	4	1	5	5409.3872	0.0022
9	2	8	10	←	8	1	7	9	5488.8236	-0.0008
6	5	1	7	←	5	4	2	6	5523.4240	0.0052
6	5	1	6	←	5	4	2	5	5523.5153	-0.0054
6	4	2	7	←	5	3	3	6	5604.8550	-0.0075
10	0	10	10	←	9	1	9	9	5715.8546	0.0064
10	1	10	10	←	9	0	9	9	5715.8546	0.0062
10	0	10	9	←	9	1	9	8	5715.8546	0.0005
10	1	10	9	←	9	0	9	8	5715.8546	0.0003
6	6	1	7	←	5	5	0	6	5737.7386	-0.0041
6	6	1	6	←	5	5	0	5	5737.8129	-0.0084
6	6	0	7	←	5	5	1	6	5742.2557	-0.0101
9	2	7	10	←	8	3	6	9	5816.9211	-0.0076
9	3	7	10	←	8	2	6	9	5821.6413	0.0027
7	5	3	8	←	6	4	2	7	5960.6185	-0.0050
7	5	3	7	←	6	4	2	6	5960.9101	0.0020
10	1	9	11	←	9	2	8	10	6043.7527	-0.0172
9	3	6	10	←	8	4	5	9	6119.5825	0.0106
11	0	11	11	←	10	1	10	10	6271.0324	0.0041
11	1	11	11	←	10	0	10	10	6271.0324	0.0040
11	0	11	10	←	10	1	10	9	6271.0324	-0.0008
11	1	11	10	←	10	0	10	9	6271.0324	-0.0009

J'	K_a'	K_c'	F'	←	J''	K_a''	K_c''	F''	ν_{obs}	$\nu_{obs}-\nu_{calc}$
11	0	11	12	←	10	1	10	11	6271.0324	-0.0075
11	1	11	12	←	10	0	10	11	6271.0324	-0.0075
10	2	8	11	←	9	3	7	10	6372.8360	-0.0039
10	3	8	11	←	9	2	7	10	6373.8426	-0.0074
7	5	2	8	←	6	4	3	7	6395.9011	0.0036
7	6	2	8	←	6	5	1	7	6423.8210	-0.0015
7	6	2	7	←	6	5	1	6	6423.9865	0.0047
7	6	1	8	←	6	5	2	7	6468.7703	-0.0001
7	6	1	7	←	6	5	2	6	6468.8745	-0.0012
11	1	10	10	←	10	2	9	9	6598.8617	0.0130
11	1	10	12	←	10	2	9	11	6598.8617	0.0102
11	2	10	10	←	10	1	9	9	6598.8617	0.0098
11	2	10	12	←	10	1	9	11	6598.8617	0.0063
10	3	7	9	←	9	4	6	8	6697.6179	-0.0079
7	7	1	7	←	6	6	0	6	6718.7123	0.0074
10	4	7	11	←	9	3	6	10	6719.8080	0.0087
7	7	0	7	←	6	6	1	6	6720.0117	-0.0181
12	0	12	12	←	11	1	11	11	6826.2044	-0.0009
12	1	12	12	←	11	0	11	11	6826.2044	-0.0009
12	0	12	11	←	11	1	11	10	6826.2044	-0.0051
12	1	12	11	←	11	0	11	10	6826.2044	-0.0051
11	3	9	12	←	10	2	8	11	6927.9557	0.0051
8	6	3	9	←	7	5	2	8	7037.2870	0.0073
8	6	3	8	←	7	5	2	7	7037.4948	-0.0131
12	1	11	11	←	11	2	10	10	7153.9536	0.0052
12	2	11	11	←	11	1	10	10	7153.9536	0.0045
12	1	11	13	←	11	2	10	12	7153.9536	0.0019
12	2	11	13	←	11	1	10	12	7153.9536	0.0013
11	4	8	12	←	10	3	7	11	7263.2840	0.0112
13	0	13	13	←	12	1	12	12	7381.3810	0.0029
13	1	13	13	←	12	0	12	12	7381.3810	0.0029
13	0	13	12	←	12	1	12	11	7381.3810	-0.0007
13	1	13	12	←	12	0	12	11	7381.3810	-0.0007
13	0	13	14	←	12	1	12	13	7381.3810	-0.0056
13	1	13	14	←	12	0	12	13	7381.3810	-0.0056
8	7	2	7	←	7	6	1	6	7418.8766	0.0032
8	7	1	9	←	7	6	2	8	7434.7101	-0.0050
8	7	1	8	←	7	6	2	7	7434.8103	-0.0008
12	2	10	13	←	11	3	9	12	7482.5598	-0.0019
8	8	1	9	←	7	7	0	8	7698.3435	-0.0083
8	8	0	9	←	7	7	1	8	7698.7302	0.0052
13	1	12	13	←	12	2	11	12	7709.0511	0.0056
13	2	12	13	←	12	1	11	12	7709.0511	0.0055
13	1	12	12	←	12	2	11	11	7709.0511	-0.0107
13	2	12	12	←	12	1	11	11	7709.0511	-0.0108
13	1	12	14	←	12	2	11	13	7709.0511	-0.0136
13	2	12	14	←	12	1	11	13	7709.0511	-0.0137
12	3	9	13	←	11	4	8	12	7813.1646	0.0151
12	4	9	11	←	11	3	8	10	7814.3811	-0.0005
14	0	14	14	←	13	1	13	13	7936.5455	-0.0002
14	1	14	14	←	13	0	13	13	7936.5455	-0.0002
14	0	14	13	←	13	1	13	12	7936.5455	-0.0035
14	1	14	13	←	13	0	13	12	7936.5455	-0.0035
14	0	14	15	←	13	1	13	14	7936.5455	-0.0077
14	1	14	15	←	13	0	13	14	7936.5455	-0.0077

Table S8. Measured rotational transitions (ν_{obs}) of the ^{13}C (23) isotopic species of the Py-(Bz)$_2$ trimer and residuals (ν_{obs} - ν_{calc}) (frequencies in MHz).

J′	*K*a′	*K*c′	*F′*	←	*J″*	*K*a″	*K*c″	*F″*	ν_{obs}	ν_{obs}-ν_{calc}
5	1	5	4	←	4	0	4	3	2925.1490	0.0092
6	0	6	5	←	5	1	5	4	3475.7586	0.0138
6	1	6	7	←	5	0	5	6	3476.2205	0.0116
4	4	1	5	←	3	3	0	4	3768.1020	-0.0191
6	1	5	5	←	5	2	4	4	3794.6406	0.0046
6	2	5	5	←	5	1	4	4	3814.9594	-0.0134
7	1	7	8	←	6	0	6	7	4028.0796	0.0140
7	2	6	8	←	6	1	5	7	4358.1833	-0.0060
5	5	0	6	←	4	4	1	5	4774.6022	-0.0101
6	4	3	7	←	5	3	2	6	4885.7856	-0.0046
8	2	7	9	←	7	1	6	8	4907.8413	-0.0025
9	0	9	9	←	8	1	8	8	5132.1418	0.0025
9	1	9	9	←	8	0	8	8	5132.1418	0.0002
9	0	9	8	←	8	1	8	7	5132.1418	-0.0044
9	1	9	8	←	8	0	8	7	5132.1418	-0.0067
9	0	9	10	←	8	1	8	9	5132.1418	-0.0140
9	1	9	10	←	8	0	8	9	5132.1418	-0.0163
8	2	6	8	←	7	3	5	7	5225.5408	-0.0107
8	3	6	9	←	7	2	5	8	5252.8184	-0.0003
9	1	8	10	←	8	2	7	9	5459.0141	-0.0004
9	2	8	10	←	8	1	7	9	5459.2115	-0.0055
10	0	10	10	←	9	1	9	9	5684.2203	0.0045
10	1	10	10	←	9	0	9	9	5684.2203	0.0041
10	0	10	9	←	9	1	9	8	5684.2203	-0.0012
10	1	10	9	←	9	0	9	8	5684.2203	-0.0016
10	0	10	11	←	9	1	9	10	5684.2203	-0.0091
10	1	10	11	←	9	0	9	10	5684.2203	-0.0095
6	6	1	7	←	5	5	0	6	5748.8391	0.0143
6	6	0	7	←	5	5	1	6	5752.4106	0.0087
9	3	7	10	←	8	2	6	9	5792.3240	-0.0041
10	1	9	11	←	9	2	8	10	6011.0080	0.0172
10	2	9	11	←	9	1	8	10	6011.0080	-0.0210
11	0	11	11	←	10	1	10	10	6236.2885	-0.0017
11	1	11	11	←	10	0	10	10	6236.2885	-0.0017
11	0	11	10	←	10	1	10	9	6236.2885	-0.0065
11	1	11	10	←	10	0	10	9	6236.2885	-0.0066
11	0	11	12	←	10	1	10	11	6236.2885	-0.0132
11	1	11	12	←	10	0	10	11	6236.2885	-0.0133
10	2	8	11	←	9	3	7	10	6338.9106	0.0088
10	3	8	10	←	9	2	7	9	6340.4137	-0.0152
11	1	10	10	←	10	2	9	9	6562.9715	0.0167
11	1	10	12	←	10	2	9	11	6562.9715	0.0131
11	2	10	10	←	10	1	9	9	6562.9715	0.0097
11	2	10	12	←	10	1	9	11	6562.9715	0.0061
10	4	7	11	←	9	3	6	10	6691.0651	-0.0064
12	0	12	12	←	11	1	11	11	6788.3586	-0.0028
12	1	12	12	←	11	0	11	11	6788.3586	-0.0028
12	0	12	11	←	11	1	11	10	6788.3586	-0.0070
12	1	12	11	←	11	0	11	10	6788.3586	-0.0070
12	0	12	13	←	11	1	11	12	6788.3586	-0.0127
12	1	12	13	←	11	0	11	12	6788.3586	-0.0127
11	2	9	12	←	10	3	8	11	6890.8185	0.0064
12	1	11	12	←	11	2	10	11	7114.9412	0.0186
12	2	11	12	←	11	1	10	11	7114.9412	0.0173
12	1	11	11	←	11	2	10	10	7114.9412	0.0000
12	2	11	11	←	11	1	10	10	7114.9412	-0.0011
12	1	11	13	←	11	2	10	12	7114.9412	-0.0032
12	2	11	13	←	11	1	10	12	7114.9412	-0.0045
11	3	8	12	←	10	4	7	11	7219.1218	0.0031
11	4	8	12	←	10	3	7	11	7227.5050	0.0046
8	6	2	9	←	7	5	3	8	7232.2096	-0.0039
13	0	13	13	←	12	1	12	12	7340.4290	0.0005
13	1	13	13	←	12	0	12	12	7340.4290	0.0005

J'	K_a'	K_c'	F'	←	J''	K_a''	K_c''	F''	ν_{obs}	$\nu_{obs}-\nu_{calc}$
13	0	13	12	←	12	1	12	11	7340.4290	-0.0030
13	1	13	12	←	12	0	12	11	7340.4290	-0.0030
13	0	13	14	←	12	1	12	13	7340.4290	-0.0079
13	1	13	14	←	12	0	12	13	7340.4290	-0.0079
9	6	4	10	←	8	5	3	9	7554.6471	0.0010
11	5	7	12	←	10	4	6	11	7626.0390	0.0055
13	1	12	12	←	12	2	11	11	7666.9524	0.0103
13	2	12	12	←	12	1	11	11	7666.9524	0.0101
13	1	12	14	←	12	2	11	13	7666.9524	0.0073
13	2	12	14	←	12	1	11	13	7666.9524	0.0071
14	0	14	14	←	13	1	13	13	7892.4798	-0.0105
14	1	14	14	←	13	0	13	13	7892.4798	-0.0105
14	0	14	13	←	13	1	13	12	7892.4798	-0.0136
14	1	14	13	←	13	0	13	12	7892.4798	-0.0136
14	0	14	15	←	13	1	13	14	7892.4798	-0.0179
14	1	14	15	←	13	0	13	14	7892.4798	-0.0179
13	2	11	14	←	12	3	10	13	7994.2654	0.0206
13	3	11	14	←	12	2	10	13	7994.2654	0.0073

Table S9. Measured rotational transitions (ν_{obs}) of the ^{13}C (24,28) isotopic species of the Py-(Bz)$_2$ trimer and residuals (ν_{obs} - ν_{calc}) (frequencies in MHz).

J'	*K*a'	*K*c'	*F'*	←	*J''*	*K*a''	*K*c''	*F''*	ν_{obs}	ν_{obs}-ν_{calc}
4	1	4	5	←	3	0	3	4	2381.7832	-0.0136
4	0	4	4	←	3	1	3	3	2370.8921	-0.0158
4	0	4	5	←	3	1	3	4	2370.9895	-0.0036
4	1	4	5	←	3	0	3	4	2381.7832	-0.0136
3	3	1	4	←	2	2	0	3	2758.9151	0.0081
3	3	1	3	←	2	2	0	2	2759.1849	-0.0060
3	3	0	4	←	2	2	1	3	2854.8611	0.0024
3	3	0	3	←	2	2	1	2	2854.9842	-0.0150
5	0	5	6	←	4	1	4	5	2928.2706	0.0001
5	1	5	4	←	4	0	4	3	2930.5141	0.0104
5	1	4	5	←	4	2	3	4	3222.3347	0.0040
5	2	3	6	←	4	3	2	5	3274.6473	0.0076
6	0	6	7	←	5	1	5	6	3482.3233	-0.0032
6	1	6	6	←	5	0	5	5	3482.7329	0.0094
4	3	1	4	←	3	2	2	3	3745.2035	0.0017
4	4	1	5	←	3	3	0	4	3766.4922	0.0099
4	4	1	4	←	3	3	0	3	3766.6681	0.0053
6	1	5	6	←	5	2	4	5	3800.5965	0.0190
6	1	5	5	←	5	2	4	4	3800.6614	-0.0003
4	4	0	5	←	3	3	1	4	3803.8199	0.0110
4	4	0	4	←	3	3	1	3	3803.9192	-0.0006
5	3	3	6	←	4	2	2	5	3817.5816	-0.0058
5	3	3	5	←	4	2	2	4	3817.8527	0.0140
6	2	5	7	←	5	1	4	6	3820.4506	-0.0095
7	1	7	8	←	6	0	6	7	4035.7919	0.0154
6	3	4	7	←	5	2	3	6	4269.0837	0.0040
6	3	4	6	←	5	2	3	5	4269.2227	0.0121
7	2	6	8	←	6	1	5	7	4365.0209	-0.0146
5	4	2	6	←	4	3	1	5	4377.4700	0.0029
5	4	2	5	←	4	3	1	4	4377.7745	0.0062
8	0	8	9	←	7	1	7	8	4588.9485	-0.0020
8	1	8	9	←	7	0	7	8	4588.9485	-0.0151
5	4	1	5	←	4	3	2	4	4601.7566	0.0091
7	2	5	7	←	6	3	4	6	4651.0979	-0.0088
5	5	1	6	←	4	4	0	5	4760.3903	0.0041
5	5	1	5	←	4	4	0	4	4760.5037	0.0047
5	5	0	6	←	4	4	1	5	4772.6648	0.0068
5	5	0	5	←	4	4	1	4	4772.7471	-0.0007
8	1	7	9	←	7	2	6	8	4914.9276	-0.0105
8	2	7	9	←	7	1	6	8	4915.9073	-0.0085
8	2	6	8	←	7	3	5	7	5233.2227	-0.0012
8	2	6	9	←	7	3	5	8	5233.3021	0.0109
8	3	6	9	←	7	2	5	8	5259.6358	0.0197
6	5	2	7	←	5	4	1	6	5416.4342	0.0073
6	5	2	6	←	5	4	1	5	5416.6390	-0.0080
9	1	8	10	←	8	2	7	9	5468.2725	-0.0010
9	2	8	10	←	8	1	7	9	5468.4708	0.0039
6	5	1	7	←	5	4	2	6	5514.8358	-0.0049
6	4	2	7	←	5	3	3	6	5560.7905	0.0019
10	0	10	10	←	9	1	9	9	5695.4053	0.0033
10	1	10	10	←	9	0	9	9	5695.4053	0.0030
10	0	10	9	←	9	1	9	8	5695.4053	-0.0023
10	1	10	9	←	9	0	9	8	5695.4053	-0.0027
10	0	10	11	←	9	1	9	10	5695.4053	-0.0103
10	1	10	11	←	9	0	9	10	5695.4053	-0.0106
6	6	1	7	←	5	5	0	6	5746.1309	-0.0205
6	6	1	6	←	5	5	0	5	5746.2208	-0.0077
6	6	0	7	←	5	5	1	6	5749.8039	0.0002
6	6	0	6	←	5	5	1	5	5749.8652	-0.0092
9	2	7	10	←	8	3	6	9	5794.0441	-0.0103
9	3	7	10	←	8	2	6	9	5800.6233	-0.0056
10	1	9	11	←	9	2	8	10	6021.4176	0.0106
9	3	6	9	←	8	4	5	8	6085.3273	-0.0106

J′	K_a'	K_c'	*F*′	←	*J*″	K_a''	K_c''	*F*″	ν_{obs}	$\nu_{obs}-\nu_{calc}$
9	3	6	10	←	8	4	5	9	6085.4441	0.0135
9	4	5	10	←	8	5	4	9	6124.6072	-0.0030
9	4	6	10	←	8	3	5	9	6188.6152	-0.0152
11	0	11	11	←	10	1	10	10	6248.6379	0.0031
11	1	11	11	←	10	0	10	10	6248.6379	0.0031
11	0	11	10	←	10	1	10	9	6248.6379	-0.0017
11	1	11	10	←	10	0	10	9	6248.6379	-0.0017
11	0	11	12	←	10	1	10	11	6248.6379	-0.0083
11	1	11	12	←	10	0	10	11	6248.6379	-0.0084
10	2	8	11	←	9	3	7	10	6348.5413	-0.0082
10	3	8	11	←	9	2	7	10	6350.0417	-0.0025
7	5	2	8	←	6	4	3	7	6360.2362	0.0031
7	6	2	8	←	6	5	1	7	6429.6324	-0.0060
7	6	2	7	←	6	5	1	6	6429.7950	0.0041
7	6	1	8	←	6	5	2	7	6466.3991	-0.0024
7	6	1	7	←	6	5	2	6	6466.5054	-0.0054
10	4	7	11	←	9	3	6	10	6699.1686	0.0035
7	4	3	8	←	6	3	4	7	6711.4215	-0.0077
12	0	12	12	←	11	1	11	11	6801.8663	0.0015
12	1	12	12	←	11	0	11	11	6801.8663	0.0015
12	0	12	11	←	11	1	11	10	6801.8663	-0.0026
12	1	12	11	←	11	0	11	10	6801.8663	-0.0026
12	0	12	13	←	11	1	11	12	6801.8663	-0.0083
12	1	12	13	←	11	0	11	12	6801.8663	-0.0083
11	3	9	12	←	10	2	8	11	6901.9297	0.0073
8	6	3	9	←	7	5	2	8	7049.8527	0.0049
8	6	3	8	←	7	5	2	7	7050.0592	-0.0035
12	1	11	11	←	11	2	10	10	7127.6877	0.0111
12	2	11	11	←	11	1	10	10	7127.6877	0.0099
12	1	11	13	←	11	2	10	12	7127.6877	0.0078
12	2	11	13	←	11	1	10	12	7127.6877	0.0066
11	3	8	10	←	10	4	7	9	7229.2149	-0.0034
8	6	2	9	←	7	5	3	8	7233.5474	-0.0024
8	6	2	8	←	7	5	3	7	7233.6252	0.0002
11	4	8	12	←	10	3	7	11	7237.2415	0.0079
13	0	13	13	←	12	1	12	12	7355.0872	-0.0036
13	1	13	13	←	12	0	12	12	7355.0872	-0.0036
13	0	13	12	←	12	1	12	11	7355.0872	-0.0072
13	1	13	12	←	12	0	12	11	7355.0872	-0.0072
13	0	13	14	←	12	1	12	13	7355.0872	-0.0121
13	1	13	14	←	12	0	12	13	7355.0872	-0.0121
8	7	2	9	←	7	6	1	8	7424.3407	0.0056
8	7	2	8	←	7	6	1	7	7424.4481	0.0017
8	7	1	9	←	7	6	2	8	7436.6491	-0.0043
8	7	1	8	←	7	6	2	7	7436.7460	-0.0044
12	2	10	13	←	11	3	9	12	7454.4627	0.0029
12	3	10	13	←	11	2	9	12	7454.5351	0.0111
11	4	7	11	←	10	5	6	10	7523.5462	0.0017
11	4	7	10	←	10	5	6	9	7523.6324	0.0118
9	6	4	9	←	8	5	3	8	7552.7271	-0.0022
13	1	12	12	←	12	2	11	11	7680.8336	-0.0038
13	2	12	12	←	12	1	11	11	7680.8336	-0.0040
13	1	12	14	←	12	2	11	13	7680.8336	-0.0068
13	2	12	14	←	12	1	11	13	7680.8336	-0.0070
8	8	1	9	←	7	7	0	8	7710.4350	0.0076
12	3	9	13	←	11	4	8	12	7783.2551	0.0022
12	4	9	13	←	11	3	8	12	7785.1634	-0.0095

Table S10. Measured rotational transitions (ν_{obs}) of the ^{13}C (25,27) isotopic species of the Py-(Bz)$_2$ trimer and residuals (ν_{obs} - ν_{calc}) (frequencies in MHz).

J'	K_a'	K_c'	F'	←	J''	K_a''	K_c''	F''	ν_{obs}	ν_{obs}- ν_{calc}
4	0	4	5	←	3	1	3	4	2377.3141	-0.0039
4	1	4	5	←	3	0	3	4	2387.5345	-0.0147
3	3	0	4	←	2	2	1	3	2857.4834	-0.0008
5	0	5	6	←	4	1	4	5	2935.8497	0.0022
5	1	5	4	←	4	0	4	3	2937.9342	0.0113
5	1	4	5	←	4	2	3	4	3232.0976	0.0064
5	2	4	6	←	4	1	3	5	3300.9563	0.0120
4	3	2	3	←	3	2	1	2	3329.5105	0.0136
4	3	2	5	←	3	2	1	4	3329.6045	0.0101
4	3	2	4	←	3	2	1	3	3329.9420	0.0072
6	0	6	7	←	5	1	5	6	3491.2882	-0.0123
6	1	6	6	←	5	0	5	5	3491.6653	0.0050
4	4	1	5	←	3	3	0	4	3767.1660	-0.0004
4	4	0	5	←	3	3	1	4	3805.8960	-0.0018
4	4	0	4	←	3	3	1	3	3806.0006	-0.0067
6	1	5	7	←	5	2	4	6	3810.6960	0.0068
5	3	3	5	←	4	2	2	4	3820.2207	0.0066
6	2	5	7	←	5	1	4	6	3829.1302	-0.0082
7	1	7	7	←	6	0	6	6	4046.1435	-0.0144
7	1	6	8	←	6	2	5	7	4371.4517	-0.0019
7	2	6	8	←	6	1	5	7	4375.6745	-0.0083
5	4	2	6	←	4	3	1	5	4378.5719	-0.0034
5	4	2	5	←	4	3	1	4	4378.8782	-0.0011
8	0	8	7	←	7	1	7	6	4600.8179	0.0127
8	1	8	8	←	7	0	7	7	4600.8179	0.0097
8	1	8	7	←	7	0	7	6	4600.8179	0.0011
8	0	8	9	←	7	1	7	8	4600.8179	0.0009
8	1	8	9	←	7	0	7	8	4600.8179	-0.0106
7	2	5	7	←	6	3	4	6	4665.1484	-0.0012
7	2	5	8	←	6	3	4	7	4665.2785	0.0085
7	3	5	8	←	6	2	4	7	4750.8764	-0.0131
5	5	1	6	←	4	4	0	5	4761.4177	0.0085
5	5	1	5	←	4	4	0	4	4761.5349	0.0120
5	5	0	6	←	4	4	1	5	4774.3495	0.0089
5	5	0	5	←	4	4	1	4	4774.4289	-0.0011
6	4	3	6	←	5	3	2	5	4886.6725	0.0045
8	1	7	9	←	7	2	6	8	4927.3065	-0.0004
8	2	7	9	←	7	1	6	8	4928.1827	-0.0026
9	0	9	9	←	8	1	8	8	5155.4996	0.0119
9	1	9	9	←	8	0	8	8	5155.4996	0.0100
9	0	9	8	←	8	1	8	7	5155.4996	0.0049
9	1	9	8	←	8	0	8	7	5155.4996	0.0030
9	0	9	10	←	8	1	8	9	5155.4996	-0.0046
9	1	9	10	←	8	0	8	9	5155.4996	-0.0065
8	2	6	8	←	7	3	5	7	5246.8883	-0.0125
8	2	6	9	←	7	3	5	8	5246.9776	0.0101
8	3	6	9	←	7	2	5	8	5271.1281	0.0112
8	3	5	8	←	7	4	4	7	5464.9850	-0.0015
8	3	5	9	←	7	4	4	8	5465.1681	-0.0111
9	1	8	10	←	8	2	7	9	5482.0698	0.0004
9	2	8	10	←	8	1	7	9	5482.2427	0.0027
6	5	1	7	←	5	4	2	6	5521.4336	0.0078
6	5	1	6	←	5	4	2	5	5521.5285	-0.0056
6	4	2	7	←	5	3	3	6	5578.5859	-0.0090
10	0	10	10	←	9	1	9	9	5710.1828	0.0062
10	1	10	10	←	9	0	9	9	5710.1828	0.0059
10	0	10	9	←	9	1	9	8	5710.1828	0.0004
10	1	10	9	←	9	0	9	8	5710.1828	0.0001
10	0	10	11	←	9	1	9	10	5710.1828	-0.0075
10	1	10	11	←	9	0	9	10	5710.1828	-0.0078
6	6	1	7	←	5	5	0	6	5747.3717	0.0019
6	6	1	6	←	5	5	0	5	5747.4516	0.0043
6	6	0	7	←	5	5	1	6	5751.2750	-0.0029

J'	K_a'	K_c'	F'	←	J''	K_a''	K_c''	F''	ν_{obs}	$\nu_{obs}-\nu_{calc}$
6	6	0	6	←	5	5	1	5	5751.3468	-0.0017
9	2	7	10	←	8	3	6	9	5808.5204	0.0020
9	3	7	10	←	8	2	6	9	5814.4457	0.0084
7	5	3	7	←	6	4	2	6	5975.7900	0.0036
10	1	9	11	←	9	2	8	10	6036.6503	-0.0044
9	4	6	10	←	8	3	5	9	6198.7054	-0.0184
11	0	11	11	←	10	1	10	10	6264.8724	0.0083
11	1	11	11	←	10	0	10	10	6264.8724	0.0083
11	0	11	10	←	10	1	10	9	6264.8724	0.0034
11	1	11	10	←	10	0	10	9	6264.8724	0.0033
11	0	11	12	←	10	1	10	11	6264.8724	-0.0032
11	1	11	12	←	10	0	10	11	6264.8724	-0.0032
10	2	8	9	←	9	3	7	8	6364.2802	0.0014
10	3	8	11	←	9	2	7	10	6365.5963	-0.0046
7	5	2	8	←	6	4	3	7	6375.9237	-0.0081
8	5	4	9	←	7	4	3	8	6421.5518	-0.0106
8	5	4	8	←	7	4	3	7	6421.8110	-0.0145
7	6	2	8	←	6	5	1	7	6432.5147	-0.0048
7	6	2	7	←	6	5	1	6	6432.6756	0.0016
7	6	1	8	←	6	5	2	7	6471.7035	-0.0015
7	6	1	7	←	6	5	2	6	6471.8099	-0.0033
11	1	10	10	←	10	2	9	9	6591.2509	0.0158
11	1	10	12	←	10	2	9	11	6591.2509	0.0122
11	2	10	10	←	10	1	9	9	6591.2509	0.0102
11	2	10	12	←	10	1	9	11	6591.2509	0.0066
10	4	7	11	←	9	3	6	10	6713.4308	-0.0035
7	7	0	8	←	6	6	1	7	6731.2061	0.0036
7	7	0	7	←	6	6	1	6	6731.2458	-0.0122
12	0	12	12	←	11	1	11	11	6819.5539	0.0048
12	1	12	12	←	11	0	11	11	6819.5539	0.0048
12	0	12	11	←	11	1	11	10	6819.5539	0.0006
12	1	12	11	←	11	0	11	10	6819.5539	0.0006
12	0	12	13	←	11	1	11	12	6819.5539	-0.0050
12	1	12	13	←	11	0	11	12	6819.5539	-0.0050
10	4	6	9	←	9	5	5	8	6908.9489	0.0115
8	6	3	8	←	7	5	2	7	7051.7468	0.0041
12	1	11	11	←	11	2	10	10	7145.8459	0.0072
12	2	11	11	←	11	1	10	10	7145.8459	0.0062
12	1	11	13	←	11	2	10	12	7145.8459	0.0039
12	2	11	13	←	11	1	10	12	7145.8459	0.0029
8	6	2	9	←	7	5	3	8	7245.8499	-0.0055
8	6	2	8	←	7	5	3	7	7245.9361	0.0098
11	3	8	12	←	10	4	7	11	7247.0460	-0.0006
11	4	8	12	←	10	3	7	11	7254.1606	0.0108
13	0	13	13	←	12	1	12	12	7374.2296	-0.0008
13	1	13	13	←	12	0	12	12	7374.2296	-0.0008
13	0	13	12	←	12	1	12	11	7374.2296	-0.0044
13	1	13	12	←	12	0	12	11	7374.2296	-0.0044
13	0	13	14	←	12	1	12	13	7374.2296	-0.0093
13	1	13	14	←	12	0	12	13	7374.2296	-0.0093
8	5	3	9	←	7	4	4	8	7418.5051	0.0002
8	7	2	9	←	7	6	1	8	7427.9274	0.0003
8	7	2	8	←	7	6	1	7	7428.0379	-0.0013
8	7	1	7	←	7	6	2	6	7441.2665	-0.0023
12	3	10	13	←	11	2	9	12	7473.1043	-0.0169
11	4	7	11	←	10	5	6	10	7545.6272	0.0040
11	4	7	12	←	10	5	6	11	7545.7035	0.0111
9	6	4	10	←	8	5	3	9	7550.6748	0.0184
13	1	12	13	←	12	2	11	12	7700.4557	0.0150
13	2	12	13	←	12	1	11	12	7700.4557	0.0149
13	1	12	12	←	12	2	11	11	7700.4563	-0.0004
13	2	12	12	←	12	1	11	11	7700.4563	-0.0006
13	1	12	14	←	12	2	11	13	7700.4563	-0.0034
13	2	12	14	←	12	1	11	13	7700.4563	-0.0036
8	8	1	9	←	7	7	0	8	7711.7476	0.0020
8	8	0	9	←	7	7	1	8	7712.0335	-0.0140
12	3	9	13	←	11	4	8	12	7802.3037	0.0019

J'	K_a'	K_c'	F'	←	J''	K_a''	K_c''	F''	ν_{obs}	$\nu_{obs}-\nu_{calc}$
14	0	14	14	←	13	1	13	13	7928.8992	-0.0083
14	1	14	14	←	13	0	13	13	7928.8992	-0.0083
14	0	14	13	←	13	1	13	12	7928.8992	-0.0115
14	1	14	13	←	13	0	13	12	7928.8992	-0.0115
14	0	14	15	←	13	1	13	14	7928.8992	-0.0158
14	1	14	15	←	13	0	13	14	7928.8992	-0.0158

Table S11. Measured rotational transitions (ν_{obs}) of the ^{13}C (26) isotopic species of the Py-(Bz)$_2$ trimer and residuals (ν_{obs} - ν_{calc}) (frequencies in MHz).

J′	*K*a′	*K*c′	*F′*	←	*J″*	*K*a″	*K*c″	*F″*	ν_{obs}	ν_{obs}-ν_{calc}
6	0	6	6	←	5	1	5	5	3493.7128	0.0086
6	1	6	6	←	5	0	5	5	3494.0751	0.0000
7	2	6	8	←	6	1	5	7	4379.5143	-0.0116
8	1	7	9	←	7	2	6	8	4931.5983	-0.0114
8	2	7	9	←	7	1	6	8	4932.4285	0.0003
9	0	9	9	←	8	1	8	8	5158.8208	0.0126
9	1	9	9	←	8	0	8	8	5158.8208	0.0109
9	0	9	8	←	8	1	8	7	5158.8208	0.0056
9	1	9	8	←	8	0	8	7	5158.8208	0.0039
9	0	9	10	←	8	1	8	9	5158.8208	-0.0039
9	1	9	10	←	8	0	8	9	5158.8208	-0.0056
9	2	8	10	←	8	1	7	9	5486.8070	-0.0025
10	0	10	10	←	9	1	9	9	5713.8022	0.0088
10	1	10	10	←	9	0	9	9	5713.8022	0.0086
10	0	10	9	←	9	1	9	8	5713.8022	0.0030
10	1	10	9	←	9	0	9	8	5713.8022	0.0027
10	0	10	11	←	9	1	9	10	5713.8022	-0.0048
10	1	10	11	←	9	0	9	10	5713.8022	-0.0051
9	3	7	10	←	8	2	6	9	5819.9947	-0.0095
10	1	9	11	←	9	2	8	10	6041.5300	-0.0026
11	0	11	11	←	10	1	10	10	6268.7846	0.0085
11	1	11	11	←	10	0	10	10	6268.7846	0.0084
11	0	11	10	←	10	1	10	9	6268.7846	0.0035
11	1	11	10	←	10	0	10	9	6268.7846	0.0035
11	0	11	12	←	10	1	10	11	6268.7846	-0.0031
11	1	11	12	←	10	0	10	11	6268.7846	-0.0031
10	2	8	11	←	9	3	7	10	6370.4169	-0.0080
10	3	8	11	←	9	2	7	10	6371.6356	-0.0064
7	6	1	8	←	6	5	2	7	6478.0269	-0.0014
7	6	1	7	←	6	5	2	6	6478.1356	0.0000
11	1	10	12	←	10	2	9	11	6596.4313	0.0181
11	2	10	10	←	10	1	9	9	6596.4313	0.0167
11	2	10	12	←	10	1	9	11	6596.4313	0.0131
12	0	12	12	←	11	1	11	11	6823.7633	0.0078
12	1	12	12	←	11	0	11	11	6823.7633	0.0078
12	0	12	11	←	11	1	11	10	6823.7633	0.0036
12	1	12	11	←	11	0	11	10	6823.7633	0.0036
12	0	12	13	←	11	1	11	12	6823.7633	-0.0020
12	1	12	13	←	11	0	11	12	6823.7633	-0.0020
11	2	9	12	←	10	3	8	11	6925.1788	0.0061
11	3	9	12	←	10	2	8	11	6925.4336	0.0101
12	1	11	11	←	11	2	10	10	7151.3187	0.0097
12	2	11	11	←	11	1	10	10	7151.3187	0.0088
12	1	11	13	←	11	2	10	12	7151.3187	0.0064
12	2	11	13	←	11	1	10	12	7151.3187	0.0055
11	3	8	12	←	10	4	7	11	7254.8546	0.0026
13	0	13	13	←	12	1	12	12	7378.7310	0.0006
13	1	13	13	←	12	0	12	12	7378.7310	0.0006
13	0	13	12	←	12	1	12	11	7378.7310	-0.0029
13	1	13	12	←	12	0	12	11	7378.7310	-0.0029
13	0	13	14	←	12	1	12	13	7378.7310	-0.0078
13	1	13	14	←	12	0	12	13	7378.7310	-0.0078
8	7	2	7	←	7	6	1	6	7433.4835	-0.0004
8	7	2	8	←	7	6	1	7	7433.5941	-0.0052
8	7	1	9	←	7	6	2	8	7447.6190	0.0079
13	1	12	12	←	12	2	11	11	7706.2279	0.0062
13	2	12	12	←	12	1	11	11	7706.2279	0.0061
13	1	12	14	←	12	2	11	13	7706.2279	0.0032
13	2	12	14	←	12	1	11	13	7706.2279	0.0031
14	0	14	14	←	13	1	13	13	7933.6860	-0.0137
14	1	14	14	←	13	0	13	13	7933.6860	-0.0137
14	0	14	13	←	13	1	13	12	7933.6860	-0.0169
14	1	14	13	←	13	0	13	12	7933.6860	-0.0169

Table S12. Measured rotational transitions (ν_{obs}) of the ^{15}N isotopic species of the Py-(Bz)$_2$ trimer and residuals (ν_{obs} - ν_{calc}) (frequencies in MHz).

J′	*K*$_a$′	*K*$_c$′	←	*J*″	*K*$_a$″	*K*$_c$″	ν_{obs}	ν_{obs}-ν_{calc}
5	0	5	←	4	1	4	2938.8017	-0.0074
5	1	5	←	4	0	4	2940.7829	0.0028
6	0	6	←	5	1	5	3494.6728	0.0019
6	1	6	←	5	0	5	3495.0195	-0.0126
7	0	7	←	6	1	6	4049.9235	-0.0051
7	1	7	←	6	0	6	4049.9977	0.0067
7	2	6	←	6	1	5	4380.6110	0.0056
5	5	0	←	4	4	1	4776.7932	-0.0013
9	0	9	←	8	1	8	5160.2228	0.0013
9	1	9	←	8	0	8	5160.2228	-0.0003
8	3	6	←	7	2	5	5276.9090	0.0095
10	0	10	←	9	1	9	5715.3538	-0.0051
10	1	10	←	9	0	9	5715.3538	-0.0053
11	0	11	←	10	1	10	6270.5002	0.0055
11	1	11	←	10	0	10	6270.5002	0.0055
10	3	8	←	9	2	7	6373.2426	-0.0171
11	1	10	←	10	2	9	6598.1852	0.0124
11	2	10	←	10	1	9	6598.1852	0.0076
12	0	12	←	11	1	11	6825.6174	-0.0101
12	1	12	←	11	0	11	6825.6174	-0.0101
12	1	11	←	11	2	10	7153.2365	0.0085
12	2	11	←	11	1	10	7153.2365	0.0076
13	0	13	←	12	1	12	7380.7764	0.0200
13	1	13	←	12	0	12	7380.7764	0.0200
14	0	14	←	13	1	13	7935.8643	-0.0159
14	1	14	←	13	0	13	7935.8643	-0.0159

Table S13. Measured rotational transitions (ν_{obs}) of the D (22) isotopic species of the Py-(Bz)$_2$ trimer and residuals (ν_{obs} - ν_{calc}) (frequencies in MHz).

J′	*K*a′	*K*c′	*F′*	←	*J″*	*K*a″	*K*c″	*F″*	ν_{obs}	ν_{obs}-ν_{calc}
4	0	4	4	←	3	1	3	3	2380.5368	0.0052
4	0	4	5	←	3	1	3	4	2380.6162	0.0009
4	1	4	5	←	3	0	3	4	2389.7901	0.0057
3	3	0	4	←	2	2	1	3	2857.5397	0.0107
5	0	5	6	←	4	1	4	5	2939.2938	0.0064
5	1	5	4	←	4	0	4	3	2941.0808	0.0039
6	0	6	5	←	5	1	5	4	3495.1526	0.0162
6	1	6	6	←	5	0	5	5	3495.4620	0.0120
4	4	1	5	←	3	3	0	4	3761.3470	0.0041
6	1	5	5	←	5	2	4	4	3817.0243	0.0067
6	2	5	7	←	5	1	4	6	3832.9934	-0.0196
7	0	7	7	←	6	1	6	6	4050.4272	-0.0126
7	1	6	8	←	6	2	5	7	4377.4973	0.0028
7	2	6	8	←	6	1	5	7	4381.0295	0.0027
8	0	8	7	←	7	1	7	6	4605.6853	0.0148
8	1	8	8	←	7	0	7	7	4605.6853	0.0145
8	1	8	7	←	7	0	7	6	4605.6853	0.0058
8	0	8	9	←	7	1	7	8	4605.6853	0.0030
8	1	8	9	←	7	0	7	8	4605.6853	-0.0059
7	2	5	8	←	6	3	4	7	4677.3375	-0.0011
5	5	1	6	←	4	4	0	5	4754.5769	0.0027
8	1	7	9	←	7	2	6	8	4933.6362	-0.0066
8	2	7	9	←	7	1	6	8	4934.3461	-0.0039
9	0	9	9	←	8	1	8	8	5160.8797	0.0091
9	1	9	9	←	8	0	8	8	5160.8797	0.0077
9	0	9	8	←	8	1	8	7	5160.8797	0.0020
9	1	9	8	←	8	0	8	7	5160.8797	0.0006
9	0	9	10	←	8	1	8	9	5160.8797	-0.0075
9	1	9	10	←	8	0	8	9	5160.8797	-0.0089
8	2	6	8	←	7	3	5	7	5256.1233	-0.0070
8	2	6	9	←	7	3	5	8	5256.1992	0.0035
8	3	6	9	←	7	2	5	8	5276.4744	0.0063
9	1	8	10	←	8	2	7	9	5488.8664	-0.0115
9	2	8	10	←	8	1	7	9	5489.0052	-0.0050
6	5	1	7	←	5	4	2	6	5524.0854	0.0007
6	5	1	6	←	5	4	2	5	5524.1958	0.0087
10	0	10	10	←	9	1	9	9	5716.0800	0.0026
10	1	10	10	←	9	0	9	9	5716.0800	0.0024
10	0	10	9	←	9	1	9	8	5716.0800	-0.0033
10	1	10	9	←	9	0	9	8	5716.0800	-0.0035
10	0	10	11	←	9	1	9	10	5716.0800	-0.0112
10	1	10	11	←	9	0	9	10	5716.0800	-0.0114
9	2	7	10	←	8	3	6	9	5817.0576	-0.0141
9	3	7	10	←	8	2	6	9	5821.8580	0.0036
9	3	6	8	←	8	4	5	7	6119.3228	-0.0005
9	4	6	10	←	8	3	5	9	6199.9129	-0.0052
11	0	11	11	←	10	1	10	10	6271.2910	0.0085
11	1	11	11	←	10	0	10	10	6271.2910	0.0085
11	0	11	10	←	10	1	10	9	6271.2910	0.0036
11	1	11	10	←	10	0	10	9	6271.2910	0.0035
11	0	11	12	←	10	1	10	11	6271.2910	-0.0030
11	1	11	12	←	10	0	10	11	6271.2910	-0.0030
10	2	8	11	←	9	3	7	10	6373.0232	-0.0047
10	3	8	11	←	9	2	7	10	6374.0414	-0.0149
7	6	2	8	←	6	5	1	7	6425.3504	0.0039
7	6	2	7	←	6	5	1	6	6425.4971	-0.0083
11	1	10	12	←	10	2	9	11	6599.0983	0.0141
11	2	10	12	←	10	1	9	11	6599.0983	0.0100
10	3	7	9	←	9	4	6	8	6697.6718	-0.0112
12	0	12	12	←	11	1	11	11	6826.4894	0.0048
12	1	12	12	←	11	0	11	11	6826.4894	0.0048
12	0	12	11	←	11	1	11	10	6826.4894	0.0005
12	1	12	11	←	11	0	11	10	6826.4894	0.0005

J'	K_a'	K_c'	F'	←	J''	K_a''	K_c''	F''	ν_{obs}	$\nu_{obs}-\nu_{calc}$
12	0	12	13	←	11	1	11	12	6826.4894	-0.0051
12	1	12	13	←	11	0	11	12	6826.4894	-0.0051
11	2	9	12	←	10	3	8	11	6927.9579	-0.0060
11	3	9	12	←	10	2	8	11	6928.1770	0.0062
12	1	11	11	←	11	2	10	10	7154.2198	0.0138
12	2	11	11	←	11	1	10	10	7154.2198	0.0131
12	1	11	13	←	11	2	10	12	7154.2198	0.0105
12	2	11	13	←	11	1	10	12	7154.2198	0.0098
8	6	2	9	←	7	5	3	8	7256.7350	0.0065
11	3	8	10	←	10	4	7	9	7257.9989	0.0060
13	0	13	13	←	12	1	12	12	7381.6853	0.0026
13	1	13	13	←	12	0	12	12	7381.6853	0.0026
13	0	13	12	←	12	1	12	11	7381.6853	-0.0010
13	1	13	12	←	12	0	12	11	7381.6853	-0.0010
13	0	13	14	←	12	1	12	13	7381.6853	-0.0059
13	1	13	14	←	12	0	12	13	7381.6853	-0.0059
8	7	2	9	←	7	6	1	8	7420.5672	-0.0037
8	7	2	8	←	7	6	1	7	7420.6882	0.0027
8	7	1	7	←	7	6	2	6	7436.2295	-0.0082
12	2	10	13	←	11	3	9	12	7482.8181	0.0159
11	5	7	10	←	10	4	6	9	7646.1848	0.0031
13	1	12	13	←	12	2	11	12	7709.3420	0.0138
13	2	12	13	←	12	1	11	12	7709.3420	0.0137
13	1	12	12	←	12	2	11	11	7709.3420	-0.0024
13	2	12	12	←	12	1	11	11	7709.3420	-0.0025
13	1	12	14	←	12	2	11	13	7709.3420	-0.0054
13	2	12	14	←	12	1	11	13	7709.3420	-0.0055
12	4	9	13	←	11	3	8	12	7814.6268	-0.0072
14	0	14	14	←	13	1	13	13	7936.8723	-0.0038
4	0	4	4	←	3	1	3	3	2380.5368	0.0052
4	0	4	5	←	3	1	3	4	2380.6162	0.0009

Table S14. Measured rotational transitions (ν_{obs}) of the D (29) isotopic species of the Py-(Bz)$_2$ trimer and residuals (ν_{obs} - ν_{calc}) (frequencies in MHz).

J'	K_a'	K_c'	F'	←	J''	K_a''	K_c''	F''	ν_{obs}	ν_{obs}-ν_{calc}
5	0	5	6	←	4	1	4	5	2910.4550	0.0022
5	1	5	6	←	4	0	4	5	2912.8441	-0.0095
6	0	6	5	←	5	1	5	4	3460.9859	0.0048
6	1	6	5	←	5	0	5	4	3461.4524	0.0091
7	2	6	8	←	6	1	5	7	4340.4555	0.0002
8	1	8	9	←	7	0	7	8	4560.5459	0.0071
8	1	7	9	←	7	2	6	8	4886.5440	-0.0188
8	2	7	9	←	7	1	6	8	4887.6296	-0.0003
9	0	9	9	←	8	1	8	8	5110.1961	0.0112
9	1	9	9	←	8	0	8	8	5110.1961	0.0088
9	0	9	8	←	8	1	8	7	5110.1961	0.0044
9	1	9	8	←	8	0	8	7	5110.1961	0.0019
9	0	9	10	←	8	1	8	9	5110.1961	-0.0052
9	1	9	10	←	8	0	8	9	5110.1961	-0.0076
8	3	6	9	←	7	2	5	8	5232.4986	-0.0056
9	1	8	10	←	8	2	7	9	5436.3550	-0.0113
9	2	8	10	←	8	1	7	9	5436.5740	-0.0063
10	0	10	10	←	9	1	9	9	5659.8772	0.0137
10	1	10	10	←	9	0	9	9	5659.8772	0.0133
10	0	10	9	←	9	1	9	8	5659.8772	0.0080
10	1	10	9	←	9	0	9	8	5659.8772	0.0076
10	0	10	11	←	9	1	9	10	5659.8772	0.0001
10	1	10	11	←	9	0	9	10	5659.8772	-0.0003
10	2	9	11	←	9	1	8	10	5985.9849	-0.0020
11	0	11	11	←	10	1	10	10	6209.5519	0.0111
11	1	11	11	←	10	0	10	10	6209.5519	0.0111
11	0	11	10	←	10	1	10	9	6209.5519	0.0063
11	1	11	10	←	10	0	10	9	6209.5519	0.0062
11	0	11	12	←	10	1	10	11	6209.5519	-0.0003
11	1	11	12	←	10	0	10	11	6209.5519	-0.0004
10	2	8	11	←	9	3	7	10	6313.1630	0.0075
10	3	8	11	←	9	2	7	10	6314.8073	-0.0011
11	1	10	12	←	10	2	9	11	6535.5224	0.0065
11	2	10	12	←	10	1	9	11	6535.5224	-0.0009
10	3	7	10	←	9	4	6	9	6632.2413	0.0136
10	3	7	11	←	9	4	6	10	6632.2757	-0.0058
7	7	1	7	←	6	6	0	6	6715.3155	-0.0029
7	7	0	8	←	6	6	1	7	6716.2219	0.0043
12	0	12	12	←	11	1	11	11	6759.2226	0.0067
12	1	12	12	←	11	0	11	11	6759.2226	0.0066
12	0	12	11	←	11	1	11	10	6759.2226	0.0025
12	1	12	11	←	11	0	11	10	6759.2226	0.0025
12	0	12	13	←	11	1	11	12	6759.2226	-0.0031
12	1	12	13	←	11	0	11	12	6759.2226	-0.0031
12	2	11	13	←	11	1	10	12	7085.1046	-0.0015
13	0	13	13	←	12	1	12	12	7308.8922	0.0043
13	1	13	13	←	12	0	12	12	7308.8922	0.0043
13	0	13	12	←	12	1	12	11	7308.8922	0.0007
13	1	13	12	←	12	0	12	11	7308.8922	0.0007
13	0	13	14	←	12	1	12	13	7308.8922	-0.0041
13	1	13	14	←	12	0	12	13	7308.8922	-0.0041
13	1	12	13	←	12	2	11	12	7634.7047	0.0141
13	2	12	13	←	12	1	11	12	7634.7047	0.0138
13	1	12	12	←	12	2	11	11	7634.7047	-0.0018
13	2	12	12	←	12	1	11	11	7634.7047	-0.0021
13	1	12	14	←	12	2	11	13	7634.7047	-0.0049
13	2	12	14	←	12	1	11	13	7634.7047	-0.0051
14	0	14	14	←	13	1	13	13	7858.5529	-0.0029
14	1	14	14	←	13	0	13	13	7858.5529	-0.0029
14	0	14	13	←	13	1	13	12	7858.5529	-0.0060
14	1	14	13	←	13	0	13	12	7858.5529	-0.0060
14	0	14	15	←	13	1	13	14	7858.5529	-0.0103
14	1	14	15	←	13	0	13	14	7858.5529	-0.0103

Table S15. Measured rotational transitions (ν_{obs}) of the D (30,34) isotopic species of the Py-(Bz)$_2$ trimer and residuals (ν_{obs} - ν_{calc}) (frequencies in MHz).

J'	K_a'	K_c'	F'	←	J''	K_a''	K_c''	F''	ν_{obs}	ν_{obs}-ν_{calc}
4	0	4	5	←	3	1	3	4	2365.8046	0.0007
4	1	4	5	←	3	0	3	4	2376.7911	-0.0075
3	3	1	4	←	2	2	0	3	2749.6298	0.0030
3	3	0	4	←	2	2	1	3	2843.8664	-0.0112
5	0	5	6	←	4	1	4	5	2922.2055	-0.0003
5	1	4	5	←	4	2	3	4	3213.0295	0.0067
5	1	4	6	←	4	2	3	5	3213.1799	-0.0120
6	0	6	6	←	5	1	5	5	3475.3146	0.0054
6	1	6	6	←	5	0	5	5	3475.7613	0.0065
4	4	1	5	←	3	3	0	4	3753.4352	0.0129
4	4	1	4	←	3	3	0	3	3753.6077	0.0056
4	4	0	5	←	3	3	1	4	3789.8372	0.0009
4	4	0	3	←	3	3	1	2	3789.8891	-0.0034
4	4	0	4	←	3	3	1	3	3789.9505	0.0026
6	1	5	6	←	5	2	4	5	3790.7055	0.0035
6	1	5	5	←	5	2	4	4	3790.7876	0.0011
6	2	5	7	←	5	1	4	6	3811.0724	-0.0124
7	1	7	7	←	6	0	6	6	4027.8387	0.0029
7	1	6	8	←	6	2	5	7	4349.7611	0.0014
7	2	6	8	←	6	1	5	7	4354.5358	-0.0034
5	4	2	6	←	4	3	1	5	4363.4950	-0.0054
5	4	2	5	←	4	3	1	4	4363.8023	0.0020
5	4	1	6	←	4	3	2	5	4582.7440	-0.0022
7	2	5	7	←	6	3	4	6	4636.8456	0.0122
5	5	1	6	←	4	4	0	5	4743.6023	0.0073
5	5	1	5	←	4	4	0	4	4743.7095	0.0023
5	5	0	5	←	4	4	1	4	4755.5731	0.0018
8	1	7	9	←	7	2	6	8	4903.3874	-0.0088
8	2	7	9	←	7	1	6	8	4904.4084	-0.0072
9	0	9	9	←	8	1	8	8	5132.3834	0.0136
9	1	9	9	←	8	0	8	8	5132.3834	0.0113
9	0	9	8	←	8	1	8	7	5132.3834	0.0067
9	1	9	8	←	8	0	8	7	5132.3834	0.0044
9	0	9	10	←	8	1	8	9	5132.3834	-0.0028
9	1	9	10	←	8	0	8	9	5132.3834	-0.0051
8	2	6	8	←	7	3	5	7	5218.6499	-0.0116
8	2	6	9	←	7	3	5	8	5218.7307	0.0015
8	3	6	9	←	7	2	5	8	5245.9294	0.0158
7	4	4	7	←	6	3	3	6	5303.9256	0.0085
6	5	2	7	←	5	4	1	6	5398.2194	0.0083
6	5	2	6	←	5	4	1	5	5398.4355	0.0056
9	1	8	10	←	8	2	7	9	5455.8054	0.0011
9	2	8	10	←	8	1	7	9	5456.0047	-0.0028
6	5	1	7	←	5	4	2	6	5493.7450	-0.0028
10	0	10	10	←	9	1	9	9	5684.6718	0.0075
10	1	10	10	←	9	0	9	9	5684.6718	0.0071
10	0	10	9	←	9	1	9	8	5684.6718	0.0017
10	1	10	9	←	9	0	9	8	5684.6718	0.0014
10	0	10	11	←	9	1	9	10	5684.6718	-0.0061
10	1	10	11	←	9	0	9	10	5684.6718	-0.0065
6	6	1	7	←	5	5	0	6	5725.7515	-0.0054
6	6	0	7	←	5	5	1	6	5729.2629	-0.0064
6	6	0	6	←	5	5	1	5	5729.3330	-0.0072
9	2	7	10	←	8	3	6	9	5778.8122	-0.0066
9	3	7	10	←	8	2	6	9	5785.6636	-0.0036
10	2	9	10	←	9	1	8	9	6008.0196	0.0110
9	3	6	8	←	8	4	5	7	6066.2360	0.0091
11	0	11	11	←	10	1	10	10	6236.9612	0.0041
11	1	11	11	←	10	0	10	10	6236.9612	0.0041
11	0	11	10	←	10	1	10	9	6236.9612	-0.0006
11	1	11	10	←	10	0	10	9	6236.9612	-0.0007
11	0	11	12	←	10	1	10	11	6236.9612	-0.0073
11	1	11	12	←	10	0	10	11	6236.9612	-0.0074
10	2	8	11	←	9	3	7	10	6332.4468	-0.0107

J'	K_a'	K_c'	F'	←	J''	K_a''	K_c''	F''	ν_{obs}	$\nu_{obs}-\nu_{calc}$
7	5	2	8	←	6	4	3	7	6332.5364	0.0007
10	3	8	11	←	9	2	7	10	6334.0203	-0.0074
8	5	4	9	←	7	4	3	8	6405.7836	-0.0151
7	6	2	8	←	6	5	1	7	6407.2097	-0.0007
7	6	2	7	←	6	5	1	6	6407.3609	-0.0010
7	6	1	8	←	6	5	2	7	6442.6254	-0.0026
7	6	1	7	←	6	5	2	6	6442.7416	0.0038
11	1	10	10	←	10	2	9	9	6560.1980	0.0153
11	1	10	12	←	10	2	9	11	6560.1980	0.0116
11	2	10	10	←	10	1	9	9	6560.1980	0.0082
11	2	10	12	←	10	1	9	11	6560.1980	0.0046
10	3	7	11	←	9	4	6	10	6649.2981	0.0014
10	4	7	10	←	9	3	6	9	6681.1279	-0.0176
7	7	1	7	←	6	6	0	6	6704.9275	-0.0056
7	7	0	8	←	6	6	1	7	6705.8545	0.0030
7	7	0	7	←	6	6	1	6	6705.9025	-0.0044
12	0	12	12	←	11	1	11	11	6789.2491	0.0020
12	1	12	12	←	11	0	11	11	6789.2491	0.0020
12	0	12	11	←	11	1	11	10	6789.2491	-0.0021
12	1	12	11	←	11	0	11	10	6789.2491	-0.0021
12	0	12	13	←	11	1	11	12	6789.2491	-0.0078
12	1	12	13	←	11	0	11	12	6789.2491	-0.0078
11	2	9	12	←	10	3	8	11	6884.5908	-0.0014
11	3	9	12	←	10	2	8	11	6884.9324	0.0036
8	6	3	9	←	7	5	2	8	7027.1719	0.0120
8	6	3	8	←	7	5	2	7	7027.3777	0.0046
12	1	11	11	←	11	2	10	10	7112.3995	0.0110
12	2	11	11	←	11	1	10	10	7112.3995	0.0098
12	1	11	13	←	11	2	10	12	7112.3995	0.0077
12	2	11	13	←	11	1	10	12	7112.3995	0.0065
11	3	8	10	←	10	4	7	9	7209.4095	-0.0065
11	4	8	12	←	10	3	7	11	7217.8356	0.0097
13	0	13	13	←	12	1	12	12	7341.5312	-0.0020
13	1	13	13	←	12	0	12	12	7341.5312	-0.0020
13	0	13	12	←	12	1	12	11	7341.5312	-0.0056
13	1	13	12	←	12	0	12	11	7341.5312	-0.0056
13	0	13	14	←	12	1	12	13	7341.5312	-0.0105
13	1	13	14	←	12	0	12	13	7341.5312	-0.0105
8	7	2	9	←	7	6	1	8	7397.9996	-0.0072
8	7	2	8	←	7	6	1	7	7398.1120	-0.0057
8	7	1	9	←	7	6	2	8	7409.7867	0.0000
8	7	1	8	←	7	6	2	7	7409.8784	-0.0054
12	2	10	13	←	11	3	9	12	7436.5187	0.0092
13	1	12	12	←	12	2	11	11	7664.6099	0.0009
13	2	12	12	←	12	1	11	11	7664.6099	0.0007
13	1	12	14	←	12	2	11	13	7664.6099	-0.0020
13	2	12	14	←	12	1	11	13	7664.6099	-0.0022
8	8	1	9	←	7	7	0	8	7683.0172	0.0047
8	8	0	9	←	7	7	1	8	7683.2814	0.0093
14	0	14	14	←	13	1	13	13	7893.8164	0.0016
14	1	14	14	←	13	0	13	13	7893.8164	0.0016
14	0	14	13	←	13	1	13	12	7893.8164	-0.0015
14	1	14	13	←	13	0	13	12	7893.8164	-0.0015
14	0	14	15	←	13	1	13	14	7893.8164	-0.0058
14	1	14	15	←	13	0	13	14	7893.8164	-0.0058

Table S16. Measured rotational transitions (ν_{obs}) of the D (31,33) isotopic species of the Py-(Bz)$_2$ trimer and residuals (ν_{obs} - ν_{calc}) (frequencies in MHz).

J'	K_a'	K_c'	F'	←	J''	K_a''	K_c''	F''	ν_{obs}	ν_{obs}-ν_{calc}
4	0	4	5	←	3	1	3	4	2377.2020	-0.0092
4	1	4	5	←	3	0	3	4	2387.1426	-0.0121
3	3	1	4	←	2	2	0	3	2750.2516	0.0078
3	3	1	3	←	2	2	0	2	2750.5389	0.0085
5	0	5	6	←	4	1	4	5	2935.8724	0.0068
5	1	5	6	←	4	0	4	5	2937.8805	-0.0157
6	0	6	5	←	5	1	5	4	3491.5145	0.0141
6	1	6	6	←	5	0	5	5	3491.8737	0.0101
4	4	1	5	←	3	3	0	4	3754.3409	0.0090
4	4	1	4	←	3	3	0	3	3754.5211	0.0060
4	4	0	5	←	3	3	1	4	3793.3094	-0.0001
4	4	0	4	←	3	3	1	3	3793.4212	0.0026
6	1	5	7	←	5	2	4	6	3808.8564	-0.0004
6	2	5	7	←	5	1	4	6	3826.6668	-0.0098
6	2	4	7	←	5	3	3	6	4028.1785	0.0167
7	0	7	7	←	6	1	6	6	4046.5271	-0.0032
6	3	4	7	←	5	2	3	6	4266.0829	0.0058
6	3	4	6	←	5	2	3	5	4266.2089	0.0051
5	4	2	5	←	4	3	1	4	4365.4423	-0.0051
7	1	6	6	←	6	2	5	5	4369.6369	-0.0017
7	2	6	8	←	6	1	5	7	4373.6885	-0.0102
5	4	1	6	←	4	3	2	5	4597.4817	-0.0042
5	4	1	5	←	4	3	2	4	4597.5851	0.0089
8	0	8	7	←	7	1	7	6	4601.4852	0.0124
8	1	8	8	←	7	0	7	7	4601.4852	0.0100
8	1	8	7	←	7	0	7	6	4601.4852	0.0014
8	0	8	9	←	7	1	7	8	4601.4852	0.0005
8	1	8	9	←	7	0	7	8	4601.4852	-0.0104
5	5	1	6	←	4	4	0	5	4745.0441	0.0131
5	5	1	5	←	4	4	0	4	4745.1555	0.0104
5	5	0	6	←	4	4	1	5	4758.1312	0.0100
5	5	0	5	←	4	4	1	4	4758.2092	-0.0011
8	1	7	9	←	7	2	6	8	4925.6570	-0.0119
8	2	7	9	←	7	1	6	8	4926.4877	-0.0179
9	0	9	9	←	8	1	8	8	5156.3909	0.0089
9	1	9	9	←	8	0	8	8	5156.3909	0.0071
9	0	9	8	←	8	1	8	7	5156.3909	0.0019
9	1	9	8	←	8	0	8	7	5156.3909	0.0001
9	0	9	10	←	8	1	8	9	5156.3909	-0.0076
9	1	9	10	←	8	0	8	9	5156.3909	-0.0094
8	2	6	8	←	7	3	5	7	5243.3051	0.0084
8	2	6	7	←	7	3	5	6	5243.3749	0.0096
8	3	6	9	←	7	2	5	8	5266.5541	0.0059
6	5	2	7	←	5	4	1	6	5401.0505	0.0139
6	5	2	6	←	5	4	1	5	5401.2685	0.0073
9	1	8	10	←	8	2	7	9	5480.6410	-0.0071
9	2	8	10	←	8	1	7	9	5480.8118	0.0023
6	5	1	7	←	5	4	2	6	5505.2951	0.0026
6	5	1	6	←	5	4	2	5	5505.4091	0.0093
10	0	10	10	←	9	1	9	9	5711.3067	0.0095
10	1	10	10	←	9	0	9	9	5711.3067	0.0092
10	0	10	9	←	9	1	9	8	5711.3067	0.0036
10	1	10	9	←	9	0	9	8	5711.3067	0.0034
10	0	10	11	←	9	1	9	10	5711.3067	-0.0042
10	1	10	11	←	9	0	9	10	5711.3067	-0.0045
6	6	1	6	←	5	5	0	5	5727.5342	-0.0011
6	6	0	7	←	5	5	1	6	5731.4272	-0.0097
6	6	0	6	←	5	5	1	5	5731.5045	-0.0031
9	2	7	10	←	8	3	6	9	5804.8384	-0.0104
9	3	7	10	←	8	2	6	9	5810.4814	-0.0092
7	5	3	8	←	6	4	2	7	5957.4150	-0.0022
7	5	3	7	←	6	4	2	6	5957.6980	0.0016
10	1	9	11	←	9	2	8	10	6035.4622	0.0025
9	3	6	10	←	8	4	5	9	6099.0043	0.0035

J'	K_a'	K_c'	F'	←	J''	K_a''	K_c''	F''	ν_{obs}	$\nu_{obs}-\nu_{calc}$
11	0	11	11	←	10	1	10	10	6266.2169	0.0065
11	1	11	11	←	10	0	10	10	6266.2169	0.0065
11	0	11	10	←	10	1	10	9	6266.2169	0.0016
11	1	11	10	←	10	0	10	9	6266.2169	0.0015
11	0	11	12	←	10	1	10	11	6266.2169	-0.0050
11	1	11	12	←	10	0	10	11	6266.2169	-0.0050
7	5	2	8	←	6	4	3	7	6360.6655	-0.0077
10	2	8	11	←	9	3	7	10	6360.7586	-0.0034
10	3	8	11	←	9	2	7	10	6362.0099	-0.0025
7	6	2	8	←	6	5	1	7	6411.9031	-0.0001
7	6	2	7	←	6	5	1	6	6412.0584	0.0000
7	6	1	8	←	6	5	2	7	6451.7394	-0.0014
7	6	1	7	←	6	5	2	6	6451.8379	-0.0106
11	1	10	10	←	10	2	9	9	6590.2863	0.0190
11	1	10	12	←	10	2	9	11	6590.2863	0.0153
11	2	10	10	←	10	1	9	9	6590.2863	0.0137
11	2	10	12	←	10	1	9	11	6590.2863	0.0101
10	3	7	9	←	9	4	6	8	6680.3117	-0.0107
7	7	1	8	←	6	6	0	7	6706.6152	-0.0105
7	7	1	7	←	6	6	0	6	6706.6854	0.0022
7	7	0	7	←	6	6	1	6	6707.8124	-0.0055
12	0	12	12	←	11	1	11	11	6821.1246	0.0041
12	1	12	12	←	11	0	11	11	6821.1246	0.0041
12	0	12	11	←	11	1	11	10	6821.1246	0.0000
12	1	12	11	←	11	0	11	10	6821.1246	0.0000
12	0	12	13	←	11	1	11	12	6821.1246	-0.0057
12	1	12	13	←	11	0	11	12	6821.1246	-0.0057
11	2	9	12	←	10	3	8	11	6915.4599	0.0085
11	3	9	12	←	10	2	8	11	6915.7179	0.0074
8	6	3	8	←	7	5	2	7	7029.8270	-0.0016
12	1	11	11	←	11	2	10	10	7145.0999	0.0025
12	2	11	11	←	11	1	10	10	7145.0999	0.0016
12	1	11	13	←	11	2	10	12	7145.0999	-0.0007
12	2	11	13	←	11	1	10	12	7145.0999	-0.0016
10	5	6	11	←	9	4	5	10	7177.9056	0.0058
8	6	2	9	←	7	5	3	8	7226.5904	-0.0023
11	3	8	12	←	10	4	7	11	7241.4804	-0.0022
13	0	13	13	←	12	1	12	12	7376.0279	0.0016
13	1	13	13	←	12	0	12	12	7376.0279	0.0016
13	0	13	12	←	12	1	12	11	7376.0279	-0.0019
13	1	13	12	←	12	0	12	11	7376.0279	-0.0019
13	0	13	14	←	12	1	12	13	7376.0279	-0.0068
13	1	13	14	←	12	0	12	13	7376.0279	-0.0068
8	7	2	9	←	7	6	1	8	7403.8995	-0.0044
8	7	2	8	←	7	6	1	7	7404.0189	0.0022
8	7	1	7	←	7	6	2	6	7417.5484	-0.0021
8	7	1	8	←	7	6	2	7	7417.6463	-0.0008
12	3	10	13	←	11	2	9	12	7470.0441	0.0000
11	4	7	10	←	10	5	6	9	7539.3719	0.0050
11	5	7	12	←	10	4	6	11	7634.2450	-0.0149
8	8	1	9	←	7	7	0	8	7684.7102	0.0002
8	8	0	9	←	7	7	1	8	7685.0128	-0.0082
13	1	12	13	←	12	2	11	12	7699.9437	0.0189
13	2	12	13	←	12	1	11	12	7699.9437	0.0188
13	1	12	12	←	12	2	11	11	7699.9437	0.0027
13	2	12	12	←	12	1	11	11	7699.9437	0.0026
13	1	12	14	←	12	2	11	13	7699.9437	-0.0002
13	2	12	14	←	12	1	11	13	7699.9437	-0.0004
12	4	9	13	←	11	3	8	12	7798.4467	-0.0016
14	0	14	14	←	13	1	13	13	7930.9311	0.0041
14	1	14	14	←	13	0	13	13	7930.9311	0.0041
14	0	14	13	←	13	1	13	12	7930.9311	0.0009
14	1	14	13	←	13	0	13	12	7930.9311	0.0009
14	0	14	15	←	13	1	13	14	7930.9311	-0.0033
14	1	14	15	←	13	0	13	14	7930.9311	-0.0033

Table S17. Measured rotational transitions (ν_{obs}) of the D (32) isotopic species of the Py-(Bz)$_2$ trimer and residuals (ν_{obs} - ν_{calc}) (frequencies in MHz).

J'	K_a'	K_c'	F'	←	J''	K_a''	K_c''	F''	ν_{obs}	ν_{obs}-ν_{calc}
4	0	4	5	←	3	1	3	4	2381.0713	-0.0141
4	1	4	5	←	3	0	3	4	2390.2206	-0.0116
4	1	3	4	←	3	2	2	4	2617.5400	-0.0047
4	1	3	5	←	3	2	2	4	2617.9603	0.0038
3	3	1	4	←	2	2	0	3	2755.0238	0.0101
3	3	1	3	←	2	2	0	2	2755.3149	0.0115
3	3	0	4	←	2	2	1	3	2857.8000	0.0055
3	3	0	3	←	2	2	1	2	2857.9458	0.0195
5	0	5	6	←	4	1	4	5	2939.8635	0.0012
5	1	5	6	←	4	0	4	5	2941.6617	-0.0125
5	1	4	5	←	4	2	3	4	3240.7895	-0.0023
5	1	4	6	←	4	2	3	5	3240.9453	-0.0080
5	2	4	6	←	4	1	3	5	3302.6834	0.0083
4	3	2	5	←	3	2	1	4	3324.9889	0.0088
6	0	6	5	←	5	1	5	4	3495.8184	-0.0035
6	1	6	5	←	5	0	5	4	3496.1498	0.0025
4	4	1	5	←	3	3	0	4	3761.5755	0.0059
4	4	1	4	←	3	3	0	3	3761.7741	0.0180
4	4	0	5	←	3	3	1	4	3803.3936	0.0054
4	4	0	4	←	3	3	1	3	3803.4795	-0.0150
6	1	5	6	←	5	2	4	5	3817.6640	0.0029
6	1	5	5	←	5	2	4	4	3817.7459	0.0027
6	2	5	7	←	5	1	4	6	3833.6766	-0.0119
7	0	7	8	←	6	1	6	7	4051.2820	0.0184
7	1	7	8	←	6	0	6	7	4051.3332	0.0146
6	3	4	7	←	5	2	3	6	4270.2704	0.0041
6	3	4	6	←	5	2	3	5	4270.3999	0.0122
5	4	2	6	←	4	3	1	5	4371.5362	0.0126
5	4	2	5	←	4	3	1	4	4371.8474	0.0142
7	1	6	8	←	6	2	5	7	4378.3122	-0.0041
7	2	6	8	←	6	1	5	7	4381.8234	-0.0111
8	0	8	8	←	7	1	7	7	4606.5818	0.0093
8	0	8	7	←	7	1	7	6	4606.5818	0.0006
8	1	8	8	←	7	0	7	7	4606.5818	0.0004
8	1	8	7	←	7	0	7	6	4606.5818	-0.0082
8	0	8	9	←	7	1	7	8	4606.5818	-0.0111
8	1	8	9	←	7	0	7	8	4606.5818	-0.0200
5	4	1	6	←	4	3	2	5	4618.4601	0.0000
5	4	1	5	←	4	3	2	4	4618.5341	-0.0083
7	2	5	7	←	6	3	4	6	4678.1650	0.0068
7	2	5	6	←	6	3	4	5	4678.2817	-0.0023
7	3	5	8	←	6	2	4	7	4752.8246	-0.0125
5	5	1	6	←	4	4	0	5	4754.8638	-0.0018
5	5	1	5	←	4	4	0	4	4754.9844	0.0027
5	5	0	6	←	4	4	1	5	4769.2839	0.0081
5	5	0	5	←	4	4	1	4	4769.3603	-0.0038
8	1	7	9	←	7	2	6	8	4934.5601	-0.0131
8	2	7	9	←	7	1	6	8	4935.2573	-0.0196
9	0	9	9	←	8	1	8	8	5161.9025	0.0082
9	1	9	9	←	8	0	8	8	5161.9025	0.0068
9	0	9	8	←	8	1	8	7	5161.9025	0.0011
9	1	9	8	←	8	0	8	7	5161.9025	-0.0002
9	0	9	10	←	8	1	8	9	5161.9025	-0.0084
9	1	9	10	←	8	0	8	9	5161.9025	-0.0098
8	2	6	8	←	7	3	5	7	5257.1127	0.0041
8	2	6	9	←	7	3	5	8	5257.1828	0.0090
8	3	6	9	←	7	2	5	8	5277.3699	0.0015
9	1	8	10	←	8	2	7	9	5489.9195	-0.0008
9	2	8	10	←	8	1	7	9	5490.0505	-0.0014
6	5	1	7	←	5	4	2	6	5524.6799	-0.0010
6	5	1	6	←	5	4	2	5	5524.7866	0.0035
6	4	2	7	←	5	3	3	6	5605.0390	0.0005
10	0	10	10	←	9	1	9	9	5717.2190	0.0048
10	1	10	10	←	9	0	9	9	5717.2190	0.0045

J'	K_a'	K_c'	F'	←	J''	K_a''	K_c''	F''	ν_{obs}	$\nu_{obs}-\nu_{calc}$
10	0	10	9	←	9	1	9	8	5717.2190	-0.0011
10	1	10	9	←	9	0	9	8	5717.2190	-0.0013
10	0	10	11	←	9	1	9	10	5717.2190	-0.0090
10	1	10	11	←	9	0	9	10	5717.2190	-0.0092
8	4	5	9	←	7	3	4	8	5729.8389	-0.0045
6	6	1	7	←	5	5	0	6	5739.5685	-0.0160
6	6	1	6	←	5	5	0	5	5739.6581	-0.0049
6	6	0	7	←	5	5	1	6	5744.0690	-0.0097
6	6	0	6	←	5	5	1	5	5744.1395	-0.0096
9	2	7	10	←	8	3	6	9	5818.1317	-0.0072
9	3	7	10	←	8	2	6	9	5822.8926	-0.0067
7	5	3	8	←	6	4	2	7	5962.8106	0.0016
10	1	9	11	←	9	2	8	10	6045.1219	-0.0118
10	2	9	11	←	9	1	8	10	6045.1736	0.0164
9	3	6	9	←	8	4	5	8	6120.4400	0.0066
9	3	6	8	←	8	4	5	7	6120.5253	0.0007
9	4	6	10	←	8	3	5	9	6200.8194	-0.0057
11	0	11	11	←	10	1	10	10	6272.5343	0.0017
11	1	11	11	←	10	0	10	10	6272.5343	0.0017
11	0	11	10	←	10	1	10	9	6272.5343	-0.0032
11	1	11	10	←	10	0	10	9	6272.5343	-0.0032
11	0	11	12	←	10	1	10	11	6272.5343	-0.0098
11	1	11	12	←	10	0	10	11	6272.5343	-0.0099
10	2	8	11	←	9	3	7	10	6374.2000	-0.0021
10	3	8	11	←	9	2	7	10	6375.2211	-0.0037
7	5	2	8	←	6	4	3	7	6396.5471	-0.0001
8	5	4	9	←	7	4	3	8	6403.9671	0.0013
8	5	4	8	←	7	4	3	7	6404.2476	0.0176
7	6	2	8	←	6	5	1	7	6425.8110	-0.0014
7	6	2	7	←	6	5	1	6	6425.9786	0.0071
7	6	1	8	←	6	5	2	7	6470.4903	-0.0018
7	6	1	7	←	6	5	2	6	6470.5965	-0.0010
11	1	10	10	←	10	2	9	9	6600.3627	0.0132
11	1	10	12	←	10	2	9	11	6600.3627	0.0095
11	2	10	10	←	10	1	9	9	6600.3627	0.0090
11	2	10	12	←	10	1	9	11	6600.3627	0.0055
10	3	7	11	←	9	4	6	10	6698.9080	0.0001
7	7	1	8	←	6	6	0	7	6720.8029	-0.0071
7	7	1	7	←	6	6	0	6	6720.8585	-0.0093
10	4	7	11	←	9	3	6	10	6721.3253	0.0110
7	7	0	8	←	6	6	1	7	6722.1234	-0.0033
7	7	0	7	←	6	6	1	6	6722.1733	-0.0091
12	0	12	12	←	11	1	11	11	6827.8477	-0.0004
12	1	12	12	←	11	0	11	11	6827.8477	-0.0004
12	0	12	11	←	11	1	11	10	6827.8477	-0.0047
12	1	12	11	←	11	0	11	10	6827.8477	-0.0047
12	0	12	13	←	11	1	11	12	6827.8477	-0.0103
12	1	12	13	←	11	0	11	12	6827.8477	-0.0103
11	2	9	12	←	10	3	8	11	6929.2510	0.0005
11	3	9	12	←	10	2	8	11	6929.4554	-0.0004
10	4	6	10	←	9	5	5	9	6940.3003	0.0092
10	4	6	11	←	9	5	5	10	6940.4315	0.0067
8	6	3	9	←	7	5	2	8	7039.7228	0.0192
8	6	3	8	←	7	5	2	7	7039.9489	0.0174
12	1	11	11	←	11	2	10	10	7155.5946	0.0060
12	2	11	11	←	11	1	10	10	7155.5946	0.0054
12	1	11	13	←	11	2	10	12	7155.5946	0.0028
12	2	11	13	←	11	1	10	12	7155.5946	0.0021
10	5	6	11	←	9	4	5	10	7182.6812	-0.0037
10	5	6	10	←	9	4	5	9	7182.7709	-0.0034
8	6	2	9	←	7	5	3	8	7257.6892	-0.0027
11	3	8	12	←	10	4	7	11	7259.3046	0.0017
11	4	8	12	←	10	3	7	11	7264.8305	0.0017
13	0	13	13	←	12	1	12	12	7383.1556	-0.0044
13	1	13	13	←	12	0	12	12	7383.1556	-0.0044
13	0	13	12	←	12	1	12	11	7383.1556	-0.0080
13	1	13	12	←	12	0	12	11	7383.1556	-0.0080
13	0	13	14	←	12	1	12	13	7383.1556	-0.0129

J′	*K*a′	*K*c′	*F*′	←	*J*″	*K*a″	*K*c″	*F*″	ν_{obs}	ν_{obs}-ν_{calc}
13	1	13	14	←	12	0	12	13	7383.1556	-0.0129
8	7	2	9	←	7	6	1	8	7421.1144	-0.0065
8	7	2	8	←	7	6	1	7	7421.2287	-0.0069
8	7	1	9	←	7	6	2	8	7436.8336	-0.0076
8	7	1	8	←	7	6	2	7	7436.9276	-0.0095
12	2	10	13	←	11	3	9	12	7484.2139	0.0116
11	4	7	12	←	10	5	6	11	7566.1010	0.0030
11	5	7	12	←	10	4	6	11	7647.2947	0.0026
8	8	1	9	←	7	7	0	8	7700.8418	-0.0003
8	8	0	9	←	7	7	1	8	7701.2035	-0.0083
13	1	12	13	←	12	2	11	12	7710.8403	0.0159
13	2	12	13	←	12	1	11	12	7710.8403	0.0158
13	1	12	12	←	12	2	11	11	7710.8403	-0.0004
13	2	12	12	←	12	1	11	11	7710.8403	-0.0005
13	1	12	14	←	12	2	11	13	7710.8403	-0.0033
13	2	12	14	←	12	1	11	13	7710.8403	-0.0034
12	3	9	13	←	11	4	8	12	7814.7991	0.0060
12	4	9	13	←	11	3	8	12	7816.0504	0.0074
14	0	14	14	←	13	1	13	13	7938.4567	-0.0105
14	1	14	14	←	13	0	13	13	7938.4567	-0.0105
14	0	14	13	←	13	1	13	12	7938.4567	-0.0137
14	1	14	13	←	13	0	13	12	7938.4567	-0.0137
14	0	14	15	←	13	1	13	14	7938.4567	-0.0180
14	1	14	15	←	13	0	13	14	7938.4567	-0.0180

1.2 Experimental rotational parameters

Table S18. Experimental rotational parameters of the $^{12}C/^{14}N/^{1}H$ (normal) isotopic species of the Py-$(Bz)_2$ trimer.

	$^{12}C/^{14}N/^{1}H$
A / MHz	491.42580(11)
B / MHz	404.123446(88)
C / MHz	278.195243(97)
Δ_J / kHz	0.04476(57)
Δ_{JK} / kHz	0.0619(20)
Δ_K/ kHz	-0.0396(14)
δ_J / kHz	0.01319(28)
δ_K / kHz[a]	[0]
χ_{aa} / MHz	-2.2464(23)
χ_{bb} / MHz	0.9352(33)
χ_{cc} / MHz	1.3112(33)
N_{lines}	317
σ_{fit} / kHz	6.3

[a] Could not be determined from the fit, value was set to 0.

Table S19. Experimental rotational parameters of the mono ^{13}C substituted isotopic species of the Py-$(Bz)_2$ trimer.

	^{13}C (1,3)	^{13}C (4,5)	^{13}C (6,10)	^{13}C (7,9)	^{13}C (8)	^{13}C (11)	^{13}C (23)
A / MHz	488.92147(13)	486.05524(14)	488.86629(14)	488.81236(16)	489.3753(50)	489.59364(15)	490.90551(35)
B / MHz	403.06369(31)	403.50341(29)	403.42360(29)	402.01899(28)	401.3752(23)	404.11757(33)	399.96713(73)
C / MHz	277.26608(18)	276.32381(20)	277.48051(20)	276.79624(22)	276.23792(28)	277.60710(22)	276.05537(31)
Δ_J / kHz	0.04460(68)	0.04335(73)	0.04268(76)	0.04360(79)	0.04144(89)	0.04389(79)	0.0448(10)
Δ_{JK} / kHz	[0.0619]	[0.0619]	[0.0619]	[0.0619]	[0.0619]	[0.0619]	[0.0619]
Δ_K/ kHz	[-0.0396]	[-0.0396]	[-0.0396]	[-0.0396]	[-0.0396]	[-0.0396]	[-0.0396]
δ_J / kHz	[0.01319]	[0.01319]	[0.01319]	[0.01319]	[0.01319]	[0.01319]	[0.01319]
δ_K / kHz[a]	[0]	[0]	[0]	[0]	[0]	[0]	[0]
χ_{aa} / MHz	[-2.2464]	[-2.2464]	[-2.2464]	[-2.2464]	[-2.2464]	[-2.2464]	[-2.2464]
χ_{bb} / MHz	[0.9352]	[0.9352]	[0.9352]	[0.9352]	[0.9352]	[0.9352]	[0.9352]
χ_{cc} / MHz	[1.3112]	[1.3112]	[1.3112]	[1.3112]	[1.3112]	[1.3112]	[1.3112]
N_{lines}	79	84	91	77	20	82	38
σ_{fit} / kHz	7.3	7.7	8.1	8.1	5.6	8.1	9.0

	^{13}C (24,28)	^{13}C (25,27)	^{13}C (26)
A / MHz	490.61130(15)	490.60602(13)	490.87486(46)
B / MHz	400.46903(21)	401.99319(21)	403.0457(13)
C / MHz	276.63350(21)	277.35985(19)	277.50965(35)
Δ_J / kHz	0.04330(81)	0.04202(72)	0.0454(11)
Δ_{JK} / kHz	[0.0619]	[0.0619]	[0.0619]
Δ_K/ kHz	[-0.0396]	[-0.0396]	[-0.0396]
δ_J / kHz	[0.01319]	[0.01319]	[0.01319]
δ_K / kHz[a]	[0]	[0]	[0]
χ_{aa} / MHz	[-2.2464]	[-2.2464]	[-2.2464]
χ_{bb} / MHz	[0.9352]	[0.9352]	[0.9352]
χ_{cc} / MHz	[1.3112]	[1.3112]	[1.3112]
N_{lines}	93	92	27
σ_{fit} / kHz	8.4	8.0	7.2

Parameters in brackets were kept at the normal species values.

Table S20. Experimental rotational parameters of the ^{15}N isotopic species of the Py-$(Bz)_2$ trimer.

	^{15}N
A / MHz	490.7179(14)
B / MHz	403.3051(36)
C / MHz	277.58586(52)
Δ_J / kHz	0.0442(15)
Δ_{JK} / kHz	[0.0619]
Δ_K/ kHz	[-0.0396]
δ_J / kHz	[0.01319]
δ_K / kHz[a]	[0]
N_{lines}	18
σ_{fit} / kHz	9.7

Parameters in brackets were kept at the normal species values.

Table S21. Experimental rotational parameters of the mono D substituted isotopic species of the Py-$(Bz)_2$ trimer.

	D (22)	D (29)	D (30,34)	D (31,33)	D (32)
A / MHz	489.73397(30)	489.73833(42)	488.87290(13)	488.81787(14)	489.75862(14)
B / MHz	404.03491(57)	398.21686(98)	398.70000(35)	401.45615(32)	404.12493(25)
C / MHz	277.61894(23)	274.85444(28)	276.16338(19)	277.47402(20)	277.67518(19)
Δ_J / kHz	0.04294(80)	0.04110(93)	0.04309(71)	0.04417(73)	0.04233(74)
Δ_{JK} / kHz	[0.0619]	[0.0619]	[0.0619]	[0.0619]	[0.0619]
Δ_K/ kHz	[-0.0396]	[-0.0396]	[-0.0396]	[-0.0396]	[-0.0396]
δ_J / kHz	[0.01319]	[0.01319]	[0.01319]	[0.01319]	[0.01319]
δ_K / kHz[a]	[0]	[0]	[0]	[0]	[0]
χ_{aa} / MHz	[-2.2464]	[-2.2464]	[-2.2464]	[-2.2464]	[-2.2464]
χ_{bb} / MHz	[0.9352]	[0.9352]	[0.9352]	[0.9352]	[0.9352]
χ_{cc} / MHz	[1.3112]	[1.3112]	[1.3112]	[1.3112]	[1.3112]
N_{lines}	79	27	80	84	110
σ_{fit} / kHz	7.3	6.8	7.0	7.6	8.6

Parameters in brackets were kept at the normal species values.

1.3 Least Squares Structural Analysis for the Py-(Bz)₂ cluster

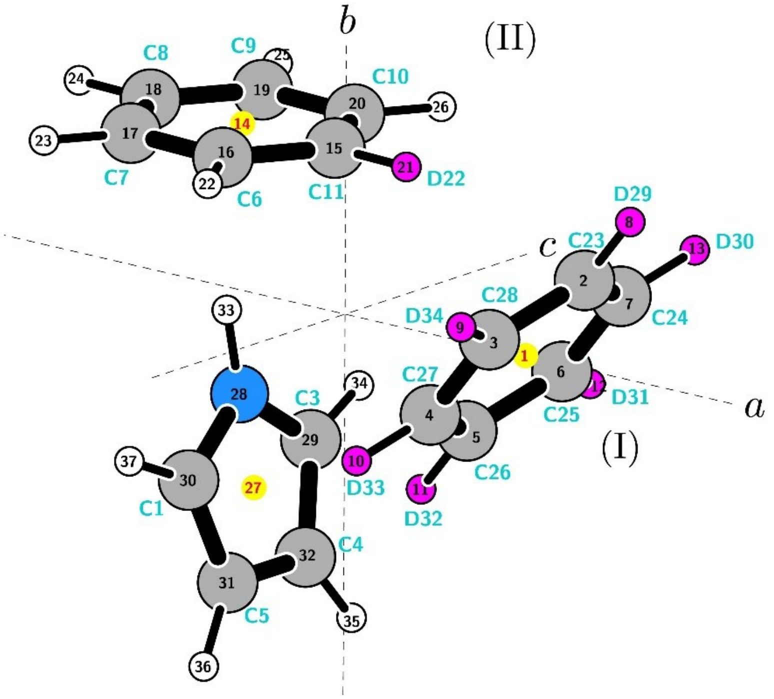

Figure S1. Atom numbering black and red used in the least-squares fit of the Py-$(Bz)_2$ structure.
Dummy atoms 1, 14, and 27 mark centers of mass of the three monomers. The orientation of the principal axes is indicated.
Singly substituted 13C isotopic species for all carbon atoms have been observed, as well as single D substituted species for hydrogen atoms indicated with magenta. Numbering used in the spectroscopic assignment in tables above is marked in turquoise.

This cluster contains 16 carbon atoms, and all singly substituted ^{13}C isotopic species have been observed in natural abundance. Since the cluster is of C_s symmetry six pairs of these carbons contain symmetry equivalent atoms, and there are two such pairs per each substituent molecule. There are, therefore, 10 distinct ^{13}C species.

This data is complemented by singly substituted species with hydrogen replaced by deuterium. These account for all possibilities in the benzene (I) unit, in which equivalence of two pairs of hydrogens leads to 4 distinct D species.

Finally, a single D species has been observed for the benzene (II) unit (at atom 21), and ^{15}N species for the pyrrole unit. Together with the parent isotopic species this leads to 1+10+1+5=17 distinct isotopic species available for structural analysis. In fact, the symmetry identical species have also been declared, in order to enforce C_s symmetry, leading to 17+6+2=25 isotopic species, corresponding to 75 experimental rotational constants used in the fit.

The heavy atom isotopic substitutions can, in principle, allow determination of the heavy atom backbones of the constituent molecules. Nevertheless, this is encumbered by several factors. The number of isotopic species is actually still too small for this task, so that intercorrelations between parameters of fit lead to rather poor parameter uncertainties. On the other hand, interactions between the constituent molecules are relatively weak and are not expected to affect the monomer geometries significantly. It is, therefore, most productive as well as chemically most informative, to use the available isotopic species to determine relative orientations of the three monomers in the cluster.

Under the overall cluster C_s symmetry, the orientation of a given constituent molecule relative to another is described by three structural parameters: their center of mass separation and two orientation angles in the symmetry (*ab*) plane. Determination of two sets of such parameters allows derivation of the third set. Two of the D species, for hydrogens 11 and 21 appeared to be outliers in the fit. This was successfully accounted for once it was realized that these were both in C-H bonds pointing towards another monomer, see Figure S2. In these two cases there would be expected to be an apparent contraction of intermolecular distance on

deuteration (the Ubbelohde effect) and a value of such contraction of 0.003 Å (similar to that for the $H_2O \cdots HCl$ cluster) rationalized the discrepancy.

An important decision in such a least squares fit concerns the assumptions used for the unfitted molecular parameters. In this case two clear alternatives were possible and have both been explored. The monomer molecular geometries could either be taken from the computation for the cluster, or the precise experimentally based equilibrium geometries available for pyrrole and benzene could be used. The results of alternative fits are intercompared in Table S22, and are there also compared with the computed values.

There is one final twist, which is associated with accounting for unknown vibrational contributions to ground state rotational constants. The most expedient way to do this is via Watson's r_m geometry as programmed into STRFIT. It turned out that only one of its key parameters, c_c, proved to be determinable. This is reasonable, since this parameter describes the correction necessary between the effective ground state C_s geometry and that in the fit. It is noted that the fit with experimental monomer geometries, which enforces C_s symmetry explicitly, requires a considerably smaller value of c_c than the fit with computed monomer geometries, which are not so constrained.

According to the above, the total number of parameters of fit is thus seven: six structural parameters defining the arrangement of molecules in the cluster, and one empirical c_c parameter. The total deviations of the structural fits and parameter uncertainties are similar for the two fits, except for the uncertainties on the derived angles A(1,27,14), A(28,27,14), A(27,14,15) (see Table S22 and Figure S2) which are significantly smaller for the experimental structure based fit. This is, therefore, the preferred fit and its key structural values are also summarized in Figure S1 and Figure 1. The confidence in center of mass separations appears to be reasonable since the values from the alternative fits agree to within or close to their nominal uncertainties from the fits. It is probably safe to assume that their actual precision is at the 0.01 Å level. The agreement between the orientation angles is variable, good for those that are explicitly fitted, but poorer for those in the pyrrole-benzene II relative orientation.

It is difficult to put a rigorous label, whether r_0, r_m, or r_e, on the reported least squares fits. The fits use uncorrected ground state rotational constants and it is noted that the fitted center of mass separations between the molecules are systematically larger than the calculated equilibrium values. The use of the effective c_c parameter and the deuterium contraction is only in the sense of mopping up relatively small effects on the overall deviation of fit. It is, therefore, reasonably safe to assume that even though the fit has some hybrid features it is mainly of r_0 character.

The abbreviated results file for the preferred least squares structural fit is included in Table S23.

Table S22. Comparison of the results of least squares structural fits for the Py-$(Bz)_2$ cluster with computed values.

```
////////////////////////////////////////////////////////////////
                         exptal re         ORCA          ORCA
Pyrr-(Benz)2             monomers (a,b)   monomers      calc (c)
////////////////////////////////////////////////////////////////

         R(14, 1) =       4.7351(34)      4.7297(33)     4.6512
         R(27, 1) =       4.7190(39)      4.7146(38)     4.7040
         R(27,14)         4.3814(27)      4.3731(26)     4.3559

      A(14, 1, 5) =      75.868(131)     76.307(127)    76.070
      A(15,14, 1) =      23.674(198)     23.800(164)    23.386
      A(27, 1,14) =      55.218(41)      55.167(40)     55.496
      A(28,27, 1) =      71.509(455)     71.822(443)    71.582
              c_c  =      0.0033(4)       0.0536(4)

     A(1,27,14)   =      62.578( 65)     57.128(174)    56.747
     A(28,27,14)  =       8.931(445)     14.909(877)    16.867
     A(27,14,15)  =      85.878(210)     84.869(421)    85.445

      Chi-squared =      0.2350          0.2213
 DevFit /uA^2     =      0.058791        0.057048
 DevFit /MHz      =      0.018895        0.019254
            NDEGf =     68(d)           68

////////////////////////////////////////////////////////////////
```

a – pyrrole geometry: A.G.Csaszar,J.Demaison, H.D.Rudolph, J.Phys.Chem.A 2015, 119,1731-1746
b – benzene geometry: J.Gauss, J.F.Stanton, J.Phys.Chem.A 2000, 104, 2865-2868.
c – refined using the DLPNO-CCSDS(T)/aug-cc-pVnZ approach
d – number of degrees of freedom (fit of 7 parameters to 75 experimental rotational constants)

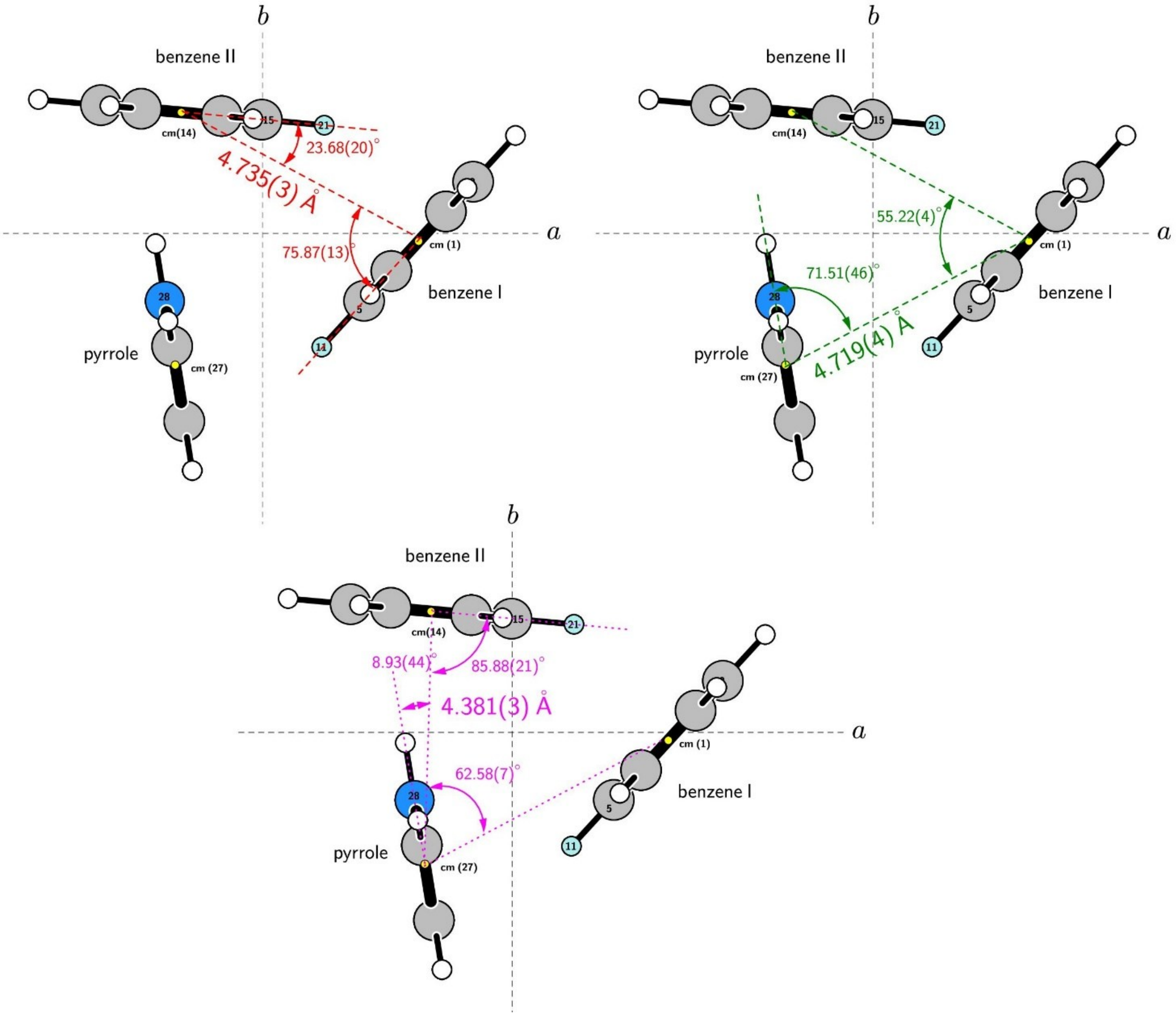

Figure S2. Structural parameters defining the relative orientation of the three molecules in the Py-$(Bz)_2$ cluster determined from the parameters of the preferred structural fit performed with the STRFIT program. Parameter uncertainties are from propagation of uncertainties on the Cartesian coordinates from the fit, evaluated with the EVAL program. The parameters defining the relative orientation of the two benzene molecules and of pyrrole-benzene I are fitted directly, while those for pyrrole-benzene II are derived from parameters of fit.

Table S23. The abbreviated results of the least-squares fit of the partial geometry of the Py-$(Bz)_2$ cluster with the STRFIT program.

```
 ___________________________________________________________________________
|                                                                           |
|    STRFIT - General structure fitting program using CART definitions      |
|___________________________________________________________________________|
 version 4.VII.2025                                          Zbigniew KISIEL

______________________________________________________________________________

Pyrrole-(Benzene)2 using re^SE monomers
______________________________________________________________________________

!
!    starting structure =  re^SE monomers
!

NUMBER OF ATOMS = 37   (including  3 dummy atoms)

  NO  NA  NB  NC       NO.NA     NO.NA.NB   NO.NA.NB.NC        MASS

   1   0   0   0     0.000000     0.000000     0.000000      0.0000000
   2   1   0   0     1.391400     0.000000     0.000000     12.0000000
   3   1   2   0     1.391400    60.000000     0.000000     12.0000000
   4   1   3   2     1.391400    60.000000  -180.000000     12.0000000
   5   1   4   3     1.391400    60.000000  -180.000000     12.0000000
   6   1   5   4     1.391400    60.000000   180.000000     12.0000000
   7   1   6   5     1.391400    60.000000  -180.000000     12.0000000
   8   2   7   6     1.080200   120.000000   180.000000      1.0078250
   9   3   2   7     1.080200   120.000000   180.000000      1.0078250
  10   4   3   2     1.080200   120.000000   180.000000      1.0078250
  11   5   4   3     1.080200   120.000000   180.000000      1.0078250
  12   6   5   4     1.080200   120.000000   180.000000      1.0078250
  13   7   6   5     1.080200   120.000000   180.000000      1.0078250
  14   1   5   6     4.651161    76.070290   -90.000000      0.0000000
  15  14   1   5     1.391400    23.385672  -180.000000     12.0000000
  16  15  14   1     1.391400    60.000000    90.000000     12.0000000
  17  16  14  15     1.391400    60.000000   180.000000     12.0000000
  18  17  14  16     1.391400    60.000000   180.000000     12.0000000
  19  18  14  17     1.391400    60.000000   180.000000     12.0000000
  20  19  14  18     1.391400    60.000000   180.000000     12.0000000
  21  15  20  19     1.080200   120.000000   180.000000      1.0078250
  22  16  15  20     1.080200   120.000000   180.000000      1.0078250
  23  17  16  15     1.080200   120.000000   180.000000      1.0078250
  24  18  17  16     1.080200   120.000000   180.000000      1.0078250
  25  19  18  17     1.080200   120.000000   180.000000      1.0078250
  26  20  19  18     1.080200   120.000000   180.000000      1.0078250
  27   1  14  24     4.703994    55.495850     0.000000      0.0000000
  28  27   1  14     1.117136    71.490000     0.000000     14.0030740
  29  28  27  14     1.369400    54.904500   -90.000000     12.0000000
  30  28  27  29     1.369400    54.904500   180.000000     12.0000000
  31  30  28  27     1.372300   107.762000     0.000000     12.0000000
  32  29  28  27     1.372300   107.762000     0.000000     12.0000000
  33  28  27  30     1.000860   180.000000     0.000000      1.0078250
  34  29  28  33     1.075320   120.990000     0.000000      1.0078250
  35  32  29  28     1.075270   125.940000   180.000000      1.0078250
  36  31  30  28     1.075270   125.940000   180.000000      1.0078250
  37  30  28  29     1.075320   120.990000   180.000000      1.0078250
!
!
!                     column:  32    38           51                    72                  91
!                               |     |            |                     |                   |
!                               --++--++++++++++++++                     cccccccccccccccccccc
!                                          value                             descriptor

---------------------------------------------
TOTAL NUMBER OF STRUCTURAL PARAMETERS:     9
---------------------------------------------
```

```
Parameters to be fitted:
          R(14, 1) =     4.651161
       A(14, 1, 5) =    76.100000
       A(15,14, 1) =    25.000000
          R(27, 1) =     4.703994
       A(27, 1,14) =    55.500000
       A(28,27, 1) =    71.490000
              c_c  =     0.000000

Fixed parameters:
              c_a  =     0.000000
              c_b  =     0.000000
---------------------------------------------

!
!c_a = c_b = c_c                -410 0   0.000000
!
!
!                    column:  32  36            50            64            78
!                              |   |             |             |             |
!                              ++--++++++++++++++--------------++++++++++++++
!
!                                   expt.  B0       g_bb         calc Be-B0
!

-------------------------------------------------------------------------------------
TOTAL NUMBER OF SPECTROSCOPIC CONSTANTS:    75

Isotopic    B_expt     Ib_expt      dI          dB      dB_el      B_corr     Ib_corr
species                          (or g_bb)
-------------------------------------------------------------------------------------
     A     491.42580 1028.39332   0.00000     0.00000  0.00000    491.42580 1028.39332
     B     404.12345 1250.55601   0.00000     0.00000  0.00000    404.12345 1250.55601
     C     278.19524 1816.63428   0.00000     0.00000  0.00000    278.19524 1816.63428
 2   A     488.92147 1033.66091   0.00000     0.00000  0.00000    488.92147 1033.66091
     B     403.06369 1253.84405   0.00000     0.00000  0.00000    403.06369 1253.84405
     C     277.26608 1822.72209   0.00000     0.00000  0.00000    277.26608 1822.72209
 3   A     490.71790 1029.87686   0.00000     0.00000  0.00000    490.71790 1029.87686
     B     403.30510 1253.09353   0.00000     0.00000  0.00000    403.30510 1253.09353
     C     277.58586 1820.62231   0.00000     0.00000  0.00000    277.58586 1820.62231
 4   A     488.92147 1033.66091   0.00000     0.00000  0.00000    488.92147 1033.66091
     B     403.06369 1253.84405   0.00000     0.00000  0.00000    403.06369 1253.84405
     C     277.26608 1822.72209   0.00000     0.00000  0.00000    277.26608 1822.72209
 5   A     486.05524 1039.75632   0.00000     0.00000  0.00000    486.05524 1039.75632
     B     403.50341 1252.47767   0.00000     0.00000  0.00000    403.50341 1252.47767
     C     276.32381 1828.93761   0.00000     0.00000  0.00000    276.32381 1828.93761
 6   A     486.05524 1039.75632   0.00000     0.00000  0.00000    486.05524 1039.75632
     B     403.50341 1252.47767   0.00000     0.00000  0.00000    403.50341 1252.47767
     C     276.32381 1828.93761   0.00000     0.00000  0.00000    276.32381 1828.93761
 7   A     488.86629 1033.77758   0.00000     0.00000  0.00000    488.86629 1033.77758
     B     403.42360 1252.72545   0.00000     0.00000  0.00000    403.42360 1252.72545
     C     277.48051 1821.31354   0.00000     0.00000  0.00000    277.48051 1821.31354
 8   A     488.81236 1033.89163   0.00000     0.00000  0.00000    488.81236 1033.89163
     B     402.01899 1257.10233   0.00000     0.00000  0.00000    402.01899 1257.10233
     C     276.79624 1825.81602   0.00000     0.00000  0.00000    276.79624 1825.81602
 9   A     489.51560 1032.40634   0.00000     0.00000  0.00000    489.51560 1032.40634
     B     401.28530 1259.40076   0.00000     0.00000  0.00000    401.28530 1259.40076
     C     276.23790 1829.50641   0.00000     0.00000  0.00000    276.23790 1829.50641
10   A     488.81236 1033.89163   0.00000     0.00000  0.00000    488.81236 1033.89163
     B     402.01899 1257.10233   0.00000     0.00000  0.00000    402.01899 1257.10233
     C     276.79624 1825.81602   0.00000     0.00000  0.00000    276.79624 1825.81602
11   A     488.86629 1033.77758   0.00000     0.00000  0.00000    488.86629 1033.77758
     B     403.42360 1252.72545   0.00000     0.00000  0.00000    403.42360 1252.72545
     C     277.48051 1821.31354   0.00000     0.00000  0.00000    277.48051 1821.31354
12   A     489.59364 1032.24178   0.00000     0.00000  0.00000    489.59364 1032.24178
     B     404.11757 1250.57421   0.00000     0.00000  0.00000    404.11757 1250.57421
     C     277.60710 1820.48301   0.00000     0.00000  0.00000    277.60710 1820.48301
13   A     490.90551 1029.48327   0.00000     0.00000  0.00000    490.90551 1029.48327
     B     399.96713 1263.55136   0.00000     0.00000  0.00000    399.96713 1263.55136
     C     276.05537 1830.71610   0.00000     0.00000  0.00000    276.05537 1830.71610
14   A     490.61130 1030.10063   0.00000     0.00000  0.00000    490.61130 1030.10063
```

```
     B     400.46903 1261.96777   0.00000    0.00000  0.00000    400.46903 1261.96777
     C     276.63350 1826.89013   0.00000    0.00000  0.00000    276.63350 1826.89013
15   A     490.60602 1030.11172   0.00000    0.00000  0.00000    490.60602 1030.11172
     B     401.99319 1257.18301   0.00000    0.00000  0.00000    401.99319 1257.18301
     C     277.35985 1822.10587   0.00000    0.00000  0.00000    277.35985 1822.10587
16   A     490.87486 1029.54755   0.00000    0.00000  0.00000    490.87486 1029.54755
     B     403.04570 1253.90002   0.00000    0.00000  0.00000    403.04570 1253.90002
     C     277.50965 1821.12229   0.00000    0.00000  0.00000    277.50965 1821.12229
17   A     490.60602 1030.11172   0.00000    0.00000  0.00000    490.60602 1030.11172
     B     401.99319 1257.18301   0.00000    0.00000  0.00000    401.99319 1257.18301
     C     277.35985 1822.10587   0.00000    0.00000  0.00000    277.35985 1822.10587
18   A     490.61130 1030.10063   0.00000    0.00000  0.00000    490.61130 1030.10063
     B     400.46903 1261.96777   0.00000    0.00000  0.00000    400.46903 1261.96777
     C     276.63350 1826.89013   0.00000    0.00000  0.00000    276.63350 1826.89013
19   A     489.75862 1031.89406   0.00000    0.00000  0.00000    489.75862 1031.89406
     B     404.12493 1250.55143   0.00000    0.00000  0.00000    404.12493 1250.55143
     C     277.67518 1820.03667   0.00000    0.00000  0.00000    277.67518 1820.03667
20   A     488.81787 1033.87998   0.00000    0.00000  0.00000    488.81787 1033.87998
     B     401.45615 1258.86479   0.00000    0.00000  0.00000    401.45615 1258.86479
     C     277.47402 1821.35614   0.00000    0.00000  0.00000    277.47402 1821.35614
21   A     488.81787 1033.87998   0.00000    0.00000  0.00000    488.81787 1033.87998
     B     401.45615 1258.86479   0.00000    0.00000  0.00000    401.45615 1258.86479
     C     277.47402 1821.35614   0.00000    0.00000  0.00000    277.47402 1821.35614
22   A     488.87290 1033.76360   0.00000    0.00000  0.00000    488.87290 1033.76360
     B     398.70000 1267.56712   0.00000    0.00000  0.00000    398.70000 1267.56712
     C     276.16338 1830.00009   0.00000    0.00000  0.00000    276.16338 1830.00009
23   A     488.87290 1033.76360   0.00000    0.00000  0.00000    488.87290 1033.76360
     B     398.70000 1267.56712   0.00000    0.00000  0.00000    398.70000 1267.56712
     C     276.16338 1830.00009   0.00000    0.00000  0.00000    276.16338 1830.00009
24   A     489.73833 1031.93681   0.00000    0.00000  0.00000    489.73833 1031.93681
     B     398.21686 1269.10500   0.00000    0.00000  0.00000    398.21686 1269.10500
     C     274.85444 1838.71510   0.00000    0.00000  0.00000    274.85444 1838.71510
25   A     489.73397 1031.94600   0.00000    0.00000  0.00000    489.73397 1031.94600
     B     404.03491 1250.83006   0.00000    0.00000  0.00000    404.03491 1250.83006
     C     277.61894 1820.40537   0.00000    0.00000  0.00000    277.61894 1820.40537
-------------------------------------------------------------------------------------
    B_corr = B_expt + dB - dB_el, Ib_corr=505379.01/B_corr
    g_bb is dimensionles, dB_el= = 0.000544617 * g_bb * B, where B=B_expt+dB
or
    Ib_corr = Ib_expt + dI,        B_corr =505379.01/Ib_corr


-------------------------------------------------------
DEFINITIONS OF SUBSTITUTED ISOTOPIC SPECIES
-------------------------------------------------------
!
!    2 = 13C-1 (Pyr)
!
ISOTOPIC SPECIES  2,  changes from parent species:
atom no.,parameter no.,value    30  4       13.0033548
!
!    3 = 15N-2 (Pyr)
!
ISOTOPIC SPECIES  3,  changes from parent species:
atom no.,parameter no.,value    28  4       15.0001089
!
!    4 = 13C-3 (Pyr)
!
ISOTOPIC SPECIES  4,  changes from parent species:
atom no.,parameter no.,value    29  4       13.0033548
!
!    5 = 13C-4 (Pyr)
!
ISOTOPIC SPECIES  5,  changes from parent species:
atom no.,parameter no.,value    32  4       13.0033548
!
!    6 = 13C-5 (Pyr)
!
ISOTOPIC SPECIES  6,  changes from parent species:
atom no.,parameter no.,value    31  4       13.0033548
!
!    7 = 13C-6 (Bz)
```

```
!
ISOTOPIC SPECIES  7,  changes from parent species:
atom no.,parameter no.,value    16  4       13.0033548
!
!    8 = 13C-7 (Bz)
!
ISOTOPIC SPECIES  8,  changes from parent species:
atom no.,parameter no.,value    17  4       13.0033548
!
!    9 = 13C-8 (Bz)
!
ISOTOPIC SPECIES  9,  changes from parent species:
atom no.,parameter no.,value    18  4       13.0033548
!
!   10 = 13C-9 (Bz)
!
ISOTOPIC SPECIES 10,  changes from parent species:
atom no.,parameter no.,value    19  4       13.0033548
!
!   11 = 13C-10 (Bz)
!
ISOTOPIC SPECIES 11,  changes from parent species:
atom no.,parameter no.,value    20  4       13.0033548
!
!   12 = 13C-11 (Bz)
!
ISOTOPIC SPECIES 12,  changes from parent species:
atom no.,parameter no.,value    15  4       13.0033548
!
!   13 = 13C-23 (Bz)
!
ISOTOPIC SPECIES 13,  changes from parent species:
atom no.,parameter no.,value     2  4       13.0033548
!
!   14 = 13C-24 (Bz)
!
ISOTOPIC SPECIES 14,  changes from parent species:
atom no.,parameter no.,value     3  4       13.0033548
!
!   15 = 13C-25 (Bz)
!
ISOTOPIC SPECIES 15,  changes from parent species:
atom no.,parameter no.,value     4  4       13.0033548
!
!   16 = 13C-26 (Bz)
!
ISOTOPIC SPECIES 16,  changes from parent species:
atom no.,parameter no.,value     5  4       13.0033548
!
!   17 = 13C-27 (Bz)
!
ISOTOPIC SPECIES 17,  changes from parent species:
atom no.,parameter no.,value     6  4       13.0033548
!
!   18 = 13C-28 (Bz)
!
ISOTOPIC SPECIES 18,  changes from parent species:
atom no.,parameter no.,value     7  4       13.0033548
!
!   19 = D-32 (Bz)
!
ISOTOPIC SPECIES 19,  changes from parent species:
atom no.,parameter no.,value    11  4        2.0141018
atom no.,parameter no.,value    27  1       -0.0060000
atom no.,parameter no.,value    27  2        0.0400000
!
!   20 = D-31 (Bz)
!
ISOTOPIC SPECIES 20,  changes from parent species:
atom no.,parameter no.,value    10  4        2.0141018
!
!   21 = D-33 (Bz)
```

```
!
ISOTOPIC SPECIES 21,  changes from parent species:
atom no.,parameter no.,value    12  4       2.0141018
!
!   22 = D-30 (Bz)
!
ISOTOPIC SPECIES 22,  changes from parent species:
atom no.,parameter no.,value     9  4       2.0141018
!
!   23 = D-34 (Bz)
!
ISOTOPIC SPECIES 23,  changes from parent species:
atom no.,parameter no.,value    13  4       2.0141018
!
!   24 = D-29 (Bz)
!
ISOTOPIC SPECIES 24,  changes from parent species:
atom no.,parameter no.,value     8  4       2.0141018
!
!   25 = D-22 (Bz)
!
ISOTOPIC SPECIES 25,  changes from parent species:
atom no.,parameter no.,value    21  4       2.0141018
atom no.,parameter no.,value    14  1      -0.0025000
atom no.,parameter no.,value    14  2       0.0300000
-------------------------------------------------------

fit after:   6 iterations,  ALAMDA=  0.10E-08

   Number of fitted spectroscopic constants = 75
                Number of parameters of fit =  7
               Number of degrees of freedom = 68

FINAL RESULTS OF LEAST SQUARES FIT:

          R(14, 1) =     4.735118 +-  0.001275   BBcm-BAcm
       A(14, 1, 5) =    75.868290 +-  0.112913   BBcm-BAcm-BAplane
       A(15,14, 1) =    23.673822 +-  0.180519   BBplane-BBcm-BAcm
          R(27, 1) =     4.718997 +-  0.001400   PYcm-BAcm
       A(27, 1,14) =    55.217682 +-  0.013904   PYcm-BAcm_BAplane
       A(28,27, 1) =    71.508647 +-  0.382494   N-Pycm-BAcm
              c_a  = [   0.000000 ]    FIXED
              c_b  = [   0.000000 ]    FIXED
              c_c  =     0.003331 +-  0.000471

     Chi-squared =        0.2296785880     = Sum( (Iobs-calc)**2 )
Deviation of fit =        0.058117 uA^2    = Sqrt(Chisq/Ndegf),  Ndegf= 68

Note that the fit is to moments of inertia but it also corresponds to:
Deviation of fit =        0.018745 MHz     = Sqrt( Sum( (Bo-c)**2 )/Ndegf )
```

Ni	Axis	Iobs	Icalc	Io-c	Bobs	Bcalc	Bo-c
1	a	1028.39332	1028.37645	0.01687	491.42580	491.43386	-0.00806
1	b	1250.55601	1250.51594	0.04007	404.12345	404.13640	-0.01295
1	c	1816.63428	1816.57563	0.05865	278.19524	278.20422	-0.00898
2	a	1033.66091	1033.71928	-0.05837	488.92147	488.89386	0.02761
2	b	1253.84405	1253.82935	0.01470	403.06369	403.06842	-0.00473
2	c	1822.72209	1822.74877	-0.02668	277.26608	277.26202	0.00406
3	a	1029.87686	1029.93975	-0.06289	490.71790	490.68793	0.02997
3	b	1253.09353	1253.02095	0.07257	403.30510	403.32846	-0.02336
3	c	1820.62231	1820.64410	-0.02179	277.58586	277.58254	0.00332
4	a	1033.66091	1033.71928	-0.05837	488.92147	488.89386	0.02761
4	b	1253.84405	1253.82935	0.01470	403.06369	403.06842	-0.00473
4	c	1822.72209	1822.74877	-0.02668	277.26608	277.26202	0.00406
5	a	1039.75632	1039.80154	-0.04522	486.05524	486.03410	0.02114
5	b	1252.47767	1252.43537	0.04229	403.50341	403.51704	-0.01363
5	c	1828.93761	1828.93004	0.00758	276.32381	276.32495	-0.00114
6	a	1039.75632	1039.80154	-0.04522	486.05524	486.03410	0.02114
6	b	1252.47767	1252.43537	0.04229	403.50341	403.51704	-0.01363
6	c	1828.93761	1828.93004	0.00758	276.32381	276.32495	-0.00114

7	a	1033.77758	1033.81405	-0.03647	488.86629	488.84904	0.01725
7	b	1252.72545	1252.71323	0.01222	403.42360	403.42753	-0.00393
7	c	1821.31354	1821.33313	-0.01959	277.48051	277.47753	0.00298
8	a	1033.89163	1033.87461	0.01702	488.81236	488.82041	-0.00805
8	b	1257.10233	1257.12375	-0.02142	402.01899	402.01214	0.00685
8	c	1825.81602	1825.82076	-0.00474	276.79624	276.79552	0.00072
9	a	1032.40634	1032.44215	-0.03580	489.51560	489.49862	0.01698
9	b	1259.40076	1259.36526	0.03550	401.28530	401.29661	-0.01131
9	c	1829.50641	1829.49114	0.01527	276.23790	276.24021	-0.00231
10	a	1033.89163	1033.87461	0.01702	488.81236	488.82041	-0.00805
10	b	1257.10233	1257.12375	-0.02142	402.01899	402.01214	0.00685
10	c	1825.81602	1825.82076	-0.00474	276.79624	276.79552	0.00072
11	a	1033.77758	1033.81405	-0.03647	488.86629	488.84904	0.01725
11	b	1252.72545	1252.71323	0.01222	403.42360	403.42753	-0.00393
11	c	1821.31354	1821.33313	-0.01959	277.48051	277.47753	0.00298
12	a	1032.24178	1032.30446	-0.06268	489.59364	489.56391	0.02973
12	b	1250.57421	1250.54440	0.02980	404.11757	404.12720	-0.00963
12	c	1820.48301	1820.53225	-0.04924	277.60710	277.59959	0.00751
13	a	1029.48327	1029.47749	0.00578	490.90551	490.90827	-0.00276
13	b	1263.55136	1263.58461	-0.03325	399.96713	399.95660	0.01053
13	c	1830.71610	1830.74589	-0.02979	276.05537	276.05088	0.00449
14	a	1030.10063	1030.08949	0.01114	490.61130	490.61660	-0.00530
14	b	1261.96777	1261.99804	-0.03027	400.46903	400.45942	0.00961
14	c	1826.89013	1826.90844	-0.01831	276.63350	276.63073	0.00277
15	a	1030.11172	1030.09551	0.01621	490.60602	490.61374	-0.00772
15	b	1257.18301	1257.18887	-0.00585	401.99319	401.99132	0.00187
15	c	1822.10587	1822.08769	0.01818	277.35985	277.36262	-0.00277
16	a	1029.54755	1029.50719	0.04036	490.87486	490.89410	-0.01924
16	b	1253.90002	1253.93118	-0.03116	403.04570	403.03568	0.01002
16	c	1821.12229	1821.12179	0.00051	277.50965	277.50973	-0.00008
17	a	1030.11172	1030.09551	0.01621	490.60602	490.61374	-0.00772
17	b	1257.18301	1257.18887	-0.00585	401.99319	401.99132	0.00187
17	c	1822.10587	1822.08769	0.01818	277.35985	277.36262	-0.00277
18	a	1030.10063	1030.08949	0.01114	490.61130	490.61660	-0.00530
18	b	1261.96777	1261.99804	-0.03027	400.46903	400.45942	0.00961
18	c	1826.89013	1826.90844	-0.01831	276.63350	276.63073	0.00277
19	a	1031.89406	1031.90737	-0.01331	489.75862	489.75230	0.00632
19	b	1250.55143	1250.67953	-0.12810	404.12493	404.08354	0.04139
19	c	1820.03667	1820.27028	-0.23361	277.67518	277.63954	0.03564
20	a	1033.87998	1033.83609	0.04389	488.81787	488.83862	-0.02075
20	b	1258.86479	1258.89557	-0.03079	401.45615	401.44633	0.00982
20	c	1821.35614	1821.29451	0.06163	277.47402	277.48341	-0.00939
21	a	1033.87998	1033.83609	0.04389	488.81787	488.83862	-0.02075
21	b	1258.86479	1258.89557	-0.03079	401.45615	401.44633	0.00982
21	c	1821.35614	1821.29451	0.06163	277.47402	277.48341	-0.00939
22	a	1033.76360	1033.76886	-0.00526	488.87290	488.87041	0.00249
22	b	1267.56712	1267.48602	0.08110	398.70000	398.72551	-0.02551
22	c	1830.00009	1829.91619	0.08390	276.16338	276.17604	-0.01266
23	a	1033.76360	1033.76886	-0.00526	488.87290	488.87041	0.00249
23	b	1267.56712	1267.48602	0.08110	398.70000	398.72551	-0.02551
23	c	1830.00009	1829.91619	0.08390	276.16338	276.17604	-0.01266
24	a	1031.93681	1031.76729	0.16952	489.73833	489.81880	-0.08047
24	b	1269.10500	1269.26252	-0.15751	398.21686	398.16744	0.04942
24	c	1838.71510	1838.71391	0.00120	274.85444	274.85462	-0.00018
25	a	1031.94600	1031.88634	0.05966	489.73397	489.76228	-0.02831
25	b	1250.83006	1250.78106	0.04900	404.03491	404.05074	-0.01583
25	c	1820.40537	1820.35079	0.05459	277.61894	277.62727	-0.00833

Correlation coefficients:

		1	2	3	4	5	6	7
1:	R(14, 1)	1.000						
2:	A(14, 1, 5)	0.017	1.000					
3:	A(15,14, 1)	0.399	0.062	1.000				
4:	R(27, 1)	-0.891	0.377	-0.466	1.000			
5:	A(27, 1,14)	-0.278	-0.837	0.118	-0.188	1.000		
6:	A(28,27, 1)	-0.783	-0.006	-0.010	0.560	0.507	1.000	
7:	c_c	-0.014	-0.001	0.000	-0.014	-0.003	0.000	1.000

Final principal coordinates of parent:

ATOM NO.	A	B	C	MASS
1	2.726258	0.004112	0.000000	0.0000000
2	3.608142	1.080344	0.000000	12.0000000
3	3.167200	0.542228	1.204988	12.0000000
4	2.285316	-0.534004	1.204988	12.0000000
5	1.844374	-1.072119	0.000000	12.0000000
6	2.285316	-0.534004	-1.204988	12.0000000
7	3.167200	0.542228	-1.204988	12.0000000
8	4.292784	1.915866	0.000000	1.0078250
9	3.509521	0.959989	2.140468	1.0078250
10	1.942995	-0.951765	2.140468	1.0078250
11	1.159731	-1.907641	0.000000	1.0078250
12	1.942995	-0.951765	-2.140468	1.0078250
13	3.509521	0.959989	-2.140468	1.0078250
14	-1.558201	2.020237	0.000000	0.0000000
15	-0.167294	1.983176	0.000000	12.0000000
16	-0.862748	2.001706	1.204988	12.0000000
17	-2.253654	2.038767	1.204988	12.0000000
18	-2.949107	2.057298	0.000000	12.0000000
19	-2.253654	2.038767	-1.204988	12.0000000
20	-0.862748	2.001706	-1.204988	12.0000000
21	0.912522	1.954403	0.000000	1.0078250
22	-0.322839	1.987320	2.140468	1.0078250
23	-2.793562	2.053153	2.140468	1.0078250
24	-4.028924	2.086070	0.000000	1.0078250
25	-2.793562	2.053153	-2.140468	1.0078250
26	-0.322839	1.987320	-2.140468	1.0078250
27	-1.359790	-2.356638	0.000000	0.0000000
28	-1.583012	-1.262031	0.000000	14.0030740
29	-1.425692	-2.033478	-1.120436	12.0000000
30	-1.425692	-2.033478	1.120436	12.0000000
31	-1.163937	-3.317039	0.711583	12.0000000
32	-1.163937	-3.317039	-0.711583	12.0000000
33	-1.783000	-0.281355	0.000000	1.0078250
34	-1.512793	-1.606363	-2.103441	1.0078250
35	-0.991730	-4.161487	-1.354590	1.0078250
36	-0.991730	-4.161487	1.354590	1.0078250
37	-1.512793	-1.606363	2.103441	1.0078250

Principal coordinates and estimated uncertainties:

ATOM NO.	A	dA	B	dB	C	dC	MASS
1	2.72626	0.00036	0.00411	0.00344	0.00000	0.00000	0.000000
2	3.60814	0.00195	1.08034	0.00427	0.00000	0.00000	12.000000
3	3.16720	0.00091	0.54223	0.00378	1.20499	0.00000	12.000000
4	2.28532	0.00131	-0.53400	0.00331	1.20499	0.00000	12.000000
5	1.84437	0.00237	-1.07212	0.00341	0.00000	0.00000	12.000000
6	2.28532	0.00131	-0.53400	0.00331	-1.20499	0.00000	12.000000
7	3.16720	0.00091	0.54223	0.00378	-1.20499	0.00000	12.000000
8	4.29278	0.00360	1.91587	0.00523	0.00000	0.00000	1.007825
9	3.50952	0.00171	0.95999	0.00415	2.14047	0.00000	1.007825
10	1.94299	0.00213	-0.95176	0.00337	2.14047	0.00000	1.007825
11	1.15973	0.00402	-1.90764	0.00396	0.00000	0.00000	1.007825
12	1.94299	0.00213	-0.95176	0.00337	-2.14047	0.00000	1.007825
13	3.50952	0.00171	0.95999	0.00415	-2.14047	0.00000	1.007825
14	-1.55820	0.00321	2.02024	0.00187	0.00000	0.00000	0.000000
15	-0.16729	0.00317	1.98318	0.00451	0.00000	0.00000	12.000000
16	-0.86275	0.00319	2.00171	0.00267	1.20499	0.00000	12.000000
17	-2.25365	0.00324	2.03877	0.00307	1.20499	0.00000	12.000000
18	-2.94911	0.00326	2.05730	0.00498	0.00000	0.00000	12.000000
19	-2.25365	0.00324	2.03877	0.00307	-1.20499	0.00000	12.000000
20	-0.86275	0.00319	2.00171	0.00267	-1.20499	0.00000	12.000000
21	0.91252	0.00314	1.95440	0.00773	0.00000	0.00000	1.007825
22	-0.32284	0.00318	1.98732	0.00407	2.14047	0.00000	1.007825
23	-2.79356	0.00325	2.05315	0.00453	2.14047	0.00000	1.007825
24	-4.02892	0.00330	2.08607	0.00823	0.00000	0.00000	1.007825
25	-2.79356	0.00325	2.05315	0.00453	-2.14047	0.00000	1.007825
26	-0.32284	0.00318	1.98732	0.00407	-2.14047	0.00000	1.007825
27	-1.35979	0.00387	-2.35664	0.00190	0.00000	0.00000	0.000000

28	-1.58301	0.00830	-1.26203	0.00298	0.00000	0.00000	14.003074
29	-1.42569	0.00432	-2.03348	0.00216	-1.12044	0.00000	12.000000
30	-1.42569	0.00432	-2.03348	0.00216	1.12044	0.00000	12.000000
31	-1.16394	0.00802	-3.31704	0.00173	0.71158	0.00000	12.000000
32	-1.16394	0.00802	-3.31704	0.00173	-0.71158	0.00000	12.000000
33	-1.78300	0.01472	-0.28136	0.00421	0.00000	0.00000	1.007825
34	-1.51279	0.00627	-1.60636	0.00259	-2.10344	0.00000	1.007825
35	-0.99173	0.01350	-4.16149	0.00236	-1.35459	0.00000	1.007825
36	-0.99173	0.01350	-4.16149	0.00236	1.35459	0.00000	1.007825
37	-1.51279	0.00627	-1.60636	0.00259	2.10344	0.00000	1.007825

NOTES: 1/ only the uncertainties for those coordinates which are completely defined by the fitted internals should be trusted
2/ the uncertainties are somewhat limited by the linear approximation coord=(d coord/d parameter)*parameter used for evaluation
3/ only the effect of the internals R, A, and D is propagated

Terms in I.fitted = I.rigid + eps

Ni	(I_a)rig	(I_b)rig	(I_c)rig	eps_a	eps_b^**	eps_c	Modified atom
1	1028.37645	1250.51594	1816.43365	0.00000	0.00000	0.14198	
2	1033.71928	1253.82935	1822.60656	0.00000	0.00000	0.14221	30
3	1029.93975	1253.02095	1820.50197	0.00000	0.00000	0.14214	28
4	1033.71928	1253.82935	1822.60656	0.00000	0.00000	0.14221	29
5	1039.80154	1252.43537	1828.78758	0.00000	0.00000	0.14246	32
6	1039.80154	1252.43537	1828.78758	0.00000	0.00000	0.14246	31
7	1033.81405	1252.71323	1821.19097	0.00000	0.00000	0.14216	16
8	1033.87461	1257.12375	1825.67843	0.00000	0.00000	0.14233	17
9	1032.44215	1259.36526	1829.34866	0.00000	0.00000	0.14248	18
10	1033.87461	1257.12375	1825.67843	0.00000	0.00000	0.14233	19
11	1033.81405	1252.71323	1821.19097	0.00000	0.00000	0.14216	20
12	1032.30446	1250.54440	1820.39012	0.00000	0.00000	0.14213	15
13	1029.47749	1263.58461	1830.60336	0.00000	0.00000	0.14253	2
14	1030.08949	1261.99804	1826.76606	0.00000	0.00000	0.14238	3
15	1030.09550	1257.18887	1821.94550	0.00000	0.00000	0.14219	4
16	1029.50719	1253.93118	1820.97963	0.00000	0.00000	0.14215	5
17	1030.09550	1257.18887	1821.94550	0.00000	0.00000	0.14219	6
18	1030.08949	1261.99804	1826.76606	0.00000	0.00000	0.14238	7
19	1031.90737	1250.67953	1820.12816	0.00000	0.00000	0.14212	11 27 27
20	1033.83608	1258.89557	1821.15235	0.00000	0.00000	0.14215	10
21	1033.83608	1258.89557	1821.15235	0.00000	0.00000	0.14215	12
22	1033.76885	1267.48602	1829.77371	0.00001	0.00000	0.14248	9
23	1033.76885	1267.48602	1829.77371	0.00001	0.00000	0.14248	13
24	1031.76729	1269.26252	1838.57107	0.00000	0.00000	0.14284	8
25	1031.88634	1250.78106	1820.20866	0.00000	0.00000	0.14212	21 14 14

** Only this value is the total EPSILON for the degenerate constant in linear and symmetric tops, the other values do not contain the rm() c and d contributions.

Table S24. Substitution coordinates (r_s) of the heavy atoms (N, C) from the general Kraitchman equations using the rotational constants of the single ^{13}C and ^{15}N isotopologues from Table S19 and Table S20.

	a	*δa*	*b*	*δb*	*c*	*δc*
C(1,3)	1.41985	0.00110	2.01662	0.00077	1.11684	0.00140
N(2)	1.58839	0.00212	1.22258	0.00280	0.12929	0.02636
C(4,5)	1.16690	0.00134	3.30838	0.00047	0.70820	0.00221
C(6,10)	0.84777	0.00184	1.98859	0.00079	1.20425	0.00130
C(7,9)	2.24005	0.00070	2.03831	0.00077	1.20529	0.00131
C(8)	2.89936	0.00121	2.11669	0.00174	0.01363·*i**	0.26574?
C(11)	0.09533	0.01651	1.96061	0.00080	0.09496	0.01662
C(23)	3.59787	0.00047	1.07386	0.00161	0.04192	0.04091
C(24,28)	3.15610	0.00049	0.53638	0.00292	1.20502	0.00130
C(25,27)	2.27595	0.00068	0.53636	0.00290	1.20363	0.00129
C(26)	1.82356	0.00105	1.08061	0.00179	0.07165	0.02690

*set to zero for distance evaluation.

Table S25. Substitution coordinates (r_s) of the H atoms from the general Kraitchman equations using the rotational constants of the single D isotopologues from Table S21.

	a	*δa*	*b*	*δb*	*c*	*δc*
H(22)	0.49183	0.00330	1.87681	0.00087	0.16713	0.00974
H(32)	0.22488·*i**	0.00692?	1.85653	0.00084	0.21695	0.00718
H(31,33)	1.93072	0.00081	0.97806	0.00160	2.13497	0.00074
H(30,34)	3.51594	0.00045	0.94985	0.00166	2.13967	0.00073
H(29)	4.26799	0.00042	1.95606	0.00093	0.07732	0.02319

*set to zero for distance evaluation.

2 $(Py)_2$-Bz trimer

2.1 Measured rotational transitions and fits

Table S26. Measured rotational transitions (ν_{obs}) of the $^{12}C/^{14}N/^{1}H$ isotopic species of the $(Py)_2$-Bz trimer and residuals (ν_{obs} - ν_{calc}) (frequencies in MHz).

J'	K_a'	K_c'	I'	F'	←	J''	K_a''	K_c''	I''	F''	ν_{obs}	ν_{obs}-ν_{calc}
3	1	3	3	3	←	2	1	2	2	3	2114.9545	-0.0051
3	1	3	3	3	←	2	1	2	2	2	2115.1545	-0.0041
3	1	3	2	2	←	2	1	2	1	1	2115.2106	-0.0053
3	1	3	4	5	←	2	1	2	3	4	2115.4590	-0.0083
3	1	3	2	1	←	2	1	2	1	1	2115.7205	-0.0004
3	1	3	2	3	←	2	1	2	3	3	2115.8926	0.0014
3	1	3	2	2	←	2	1	2	2	2	2116.1271	-0.0004
3	1	3	4	3	←	2	1	2	2	3	2116.2380	-0.0028
3	0	3	4	4	←	2	0	2	3	4	2145.6766	-0.0023
3	0	3	3	3	←	2	0	2	2	2	2146.1706	0.0019
3	0	3	4	5	←	2	0	2	3	4	2146.4098	0.0036
3	0	3	2	1	←	2	0	2	1	1	2146.6118	0.0027
3	0	3	2	3	←	2	0	2	2	3	2146.7988	-0.0121
3	0	3	2	2	←	2	0	2	2	2	2147.1560	0.0000
3	0	3	4	3	←	2	0	2	3	3	2147.2817	-0.0034
3	2	2	3	3	←	2	2	1	2	2	2371.5060	-0.0047
3	2	2	4	4	←	2	2	1	3	3	2371.5787	0.0055
3	2	2	3	4	←	2	2	1	2	3	2372.1309	-0.0001
3	2	2	4	5	←	2	2	1	3	4	2372.2437	0.0110
3	2	2	4	3	←	2	2	1	3	2	2372.5736	-0.0028
3	2	2	2	1	←	2	2	1	1	0	2372.6717	0.0160
2	2	0	1	2	←	1	0	1	0	1	2380.3949	0.0140
2	2	0	3	4	←	1	0	1	2	3	2380.5640	0.0035
2	2	0	3	3	←	1	0	1	2	2	2381.8613	0.0000
3	1	2	3	3	←	2	1	1	2	3	2515.8368	0.0127
3	1	2	3	3	←	2	1	1	3	2	2516.1072	0.0084
3	1	2	3	4	←	2	1	1	2	3	2516.2225	0.0067
3	1	2	4	5	←	2	1	1	3	4	2516.3464	-0.0081
3	1	2	4	3	←	2	1	1	3	2	2516.4803	0.0026
3	1	2	2	3	←	2	1	1	1	2	2516.5505	-0.0048
3	1	2	4	3	←	2	1	1	3	3	2516.6929	-0.0039
3	1	2	2	1	←	2	1	1	1	1	2516.7535	0.0007
3	2	1	2	2	←	2	2	0	2	2	2596.9054	-0.0001
3	2	1	2	2	←	2	2	0	2	1	2597.1450	0.0024
3	2	1	4	4	←	2	2	0	3	3	2597.2227	0.0027
3	2	1	3	3	←	2	2	0	2	2	2597.4270	0.0004
3	2	1	4	4	←	2	2	0	2	3	2597.6037	-0.0152
3	2	1	3	2	←	2	2	0	2	1	2597.7010	0.0051
3	2	1	4	5	←	2	2	0	3	4	2597.9198	0.0016
3	2	1	3	4	←	2	2	0	2	3	2598.0274	-0.0042
3	2	1	4	3	←	2	2	0	3	2	2598.2353	0.0000
3	2	1	3	2	←	2	2	0	3	2	2598.6103	-0.0090
3	2	2	3	4	←	2	1	1	2	3	2696.9505	0.0036
3	2	2	4	5	←	2	1	1	3	4	2697.2535	0.0128
3	2	2	4	4	←	2	1	1	3	3	2697.4403	-0.0005
4	1	4	5	5	←	3	1	3	4	5	2772.7681	-0.0016
4	1	4	4	4	←	3	1	3	3	3	2773.3726	0.0005
4	1	4	5	6	←	3	1	3	4	5	2773.5494	0.0002
4	1	4	3	2	←	3	1	3	2	2	2773.9988	0.0089
4	1	4	3	3	←	3	1	3	3	3	2774.3481	-0.0073
4	0	4	5	5	←	3	0	3	4	5	2781.5472	-0.0034
4	0	4	4	4	←	3	0	3	4	4	2781.8073	0.0038
4	0	4	4	4	←	3	0	3	3	3	2782.1639	-0.0007
4	0	4	3	2	←	3	0	3	2	2	2782.7564	-0.0094
4	0	4	3	4	←	3	0	3	3	4	2782.8679	-0.0058
4	1	4	4	4	←	3	0	3	3	3	2784.6206	-0.0027
4	1	3	5	5	←	3	2	2	4	4	3026.6157	0.0040
4	1	3	5	6	←	3	2	2	4	5	3026.9853	-0.0066
4	1	3	5	4	←	3	2	2	4	3	3027.0752	-0.0126
4	2	3	4	4	←	3	2	2	3	3	3097.3483	0.0006

J'	K_a'	K_c'	I'	F'	←	J''	K_a''	K_c''	I''	F''	ν_{obs}	$\nu_{obs}-\nu_{calc}$
4	2	3	5	5	←	3	2	2	4	4	3097.4321	-0.0110
4	2	3	4	5	←	3	2	2	3	4	3097.6060	0.0011
4	2	3	5	4	←	3	2	2	4	3	3097.7677	-0.0040
4	1	3	4	4	←	3	1	2	3	4	3207.3178	0.0035
4	1	3	4	4	←	3	1	2	2	3	3207.4976	0.0020
4	1	3	4	5	←	3	1	2	3	4	3207.6900	-0.0011
4	1	3	3	4	←	3	1	2	3	3	3208.1167	-0.0048
4	1	3	5	4	←	3	1	2	4	4	3208.3143	-0.0010
4	1	3	3	2	←	3	1	2	2	2	3208.3713	-0.0008
3	3	1	3	4	←	2	2	0	2	3	3236.7378	-0.0019
3	3	1	4	5	←	2	2	0	3	4	3236.8267	0.0094
3	3	1	4	4	←	2	2	0	3	3	3237.0060	0.0146
4	3	2	4	4	←	3	3	1	4	4	3263.5953	0.0052
4	3	2	3	4	←	3	3	1	3	4	3263.8390	0.0054
4	3	2	4	5	←	3	3	1	3	4	3264.0116	0.0043
4	3	2	5	4	←	3	3	1	4	3	3264.2750	-0.0050
4	2	3	4	5	←	3	1	2	3	4	3278.3234	-0.0125
3	3	0	4	5	←	2	2	1	3	4	3345.8649	-0.0077
3	3	0	3	4	←	2	2	1	2	3	3346.0113	-0.0130
4	3	1	3	3	←	3	3	0	3	3	3404.9102	-0.0043
4	3	1	5	5	←	3	3	0	4	4	3405.2245	-0.0057
4	3	1	4	4	←	3	3	0	3	3	3405.3764	0.0017
4	3	1	4	3	←	3	3	0	2	2	3405.4789	-0.0144
4	3	1	4	4	←	3	3	0	4	4	3405.6015	0.0128
4	3	1	5	5	←	3	3	0	3	4	3405.8009	0.0048
4	3	1	5	6	←	3	3	0	4	5	3405.9103	0.0023
4	3	1	4	5	←	3	3	0	3	4	3406.0284	0.0034
4	3	1	5	4	←	3	3	0	4	3	3406.1427	0.0044
4	3	1	4	3	←	3	3	0	4	3	3406.5068	-0.0040
5	1	5	6	6	←	4	1	4	5	6	3422.4378	-0.0094
5	1	5	5	5	←	4	1	4	5	5	3422.6789	0.0050
5	1	5	5	5	←	4	1	4	4	4	3423.1570	0.0079
5	1	5	6	7	←	4	1	4	5	6	3423.2499	-0.0176
5	1	5	4	3	←	4	1	4	3	3	3423.8218	-0.0022
5	1	5	4	5	←	4	1	4	4	5	3423.8971	0.0033
5	1	5	5	4	←	4	1	4	4	4	3424.1484	0.0100
4	3	1	4	3	←	3	3	0	3	2	3406.3065	-0.0003
5	0	5	6	7	←	4	1	4	5	6	3422.7740	-0.0102
5	0	5	6	6	←	4	0	4	5	6	3424.4119	-0.0089
5	0	5	5	5	←	4	0	4	5	5	3424.6518	0.0019
5	0	5	5	5	←	4	0	4	4	4	3425.1343	0.0101
5	1	5	5	5	←	4	0	4	4	4	3425.6200	0.0122
5	1	5	6	7	←	4	0	4	5	6	3425.7103	-0.0146
5	0	5	4	5	←	4	0	4	4	5	3425.8674	0.0008
4	2	2	3	3	←	3	2	1	3	3	3467.1449	-0.0010
4	2	2	3	4	←	3	2	1	3	4	3467.3233	-0.0028
4	2	2	5	4	←	3	2	1	3	3	3467.4922	0.0038
4	2	2	3	2	←	3	2	1	2	1	3467.9069	-0.0142
4	2	2	4	5	←	3	2	1	4	5	3468.1695	0.0020
4	2	2	5	5	←	3	2	1	4	4	3467.6124	-0.0066
4	2	2	3	3	←	3	2	1	2	2	3467.6620	-0.0050
4	2	2	5	6	←	3	2	1	4	5	3467.8080	-0.0170
3	2	1	2	3	←	2	0	2	1	2	3474.6011	-0.0118
3	2	1	4	5	←	2	0	2	3	4	3474.8788	0.0040
3	2	1	4	4	←	2	0	2	2	3	3475.1068	-0.0073
3	2	1	3	4	←	2	0	2	2	3	3475.5152	-0.0116
3	2	1	3	3	←	2	0	2	2	2	3476.0823	-0.0027
3	3	0	4	5	←	2	1	1	3	4	3670.8690	-0.0117
3	3	0	2	3	←	2	1	1	1	2	3671.0366	0.0026
3	3	0	4	4	←	2	1	1	3	3	3671.8905	-0.0094
3	3	0	2	2	←	2	1	1	1	1	3671.9775	0.0084
5	1	4	5	5	←	4	2	3	4	4	3764.1773	0.0040
5	1	4	6	6	←	4	2	3	5	5	3764.2572	0.0046
5	1	4	6	7	←	4	2	3	5	6	3764.4276	0.0117
5	2	4	6	6	←	4	2	3	5	6	3784.6363	-0.0016
5	2	4	5	5	←	4	2	3	4	4	3784.8044	0.0000
5	2	4	6	7	←	4	2	3	5	6	3785.0417	0.0029
5	2	4	4	3	←	4	2	3	4	3	3785.1917	-0.0012

J'	K_a'	K_c'	I'	F'	←	J''	K_a''	K_c''	I''	F''	ν_{obs}	$\nu_{obs}-\nu_{calc}$
5	2	4	5	4	←	4	2	3	4	4	3785.2782	-0.0048
5	2	4	6	5	←	4	2	3	5	5	3785.3783	-0.0011
5	1	4	5	5	←	4	1	3	4	5	3834.5470	-0.0139
5	1	4	5	6	←	4	1	3	4	5	3834.9666	-0.0137
5	1	4	6	6	←	4	1	3	5	5	3835.0964	0.0123
5	1	4	5	4	←	4	1	3	4	4	3835.4435	-0.0061
5	1	4	6	5	←	4	1	3	5	5	3835.6018	0.0070
5	2	4	5	5	←	4	1	3	4	4	3855.5728	0.0038
5	3	3	6	6	←	4	3	2	5	5	4040.2407	-0.0004
5	3	3	5	6	←	4	3	2	4	5	4040.4825	-0.0111
5	3	3	6	7	←	4	3	2	5	6	4040.5422	-0.0034
6	1	6	7	7	←	5	1	5	6	7	4069.6856	0.0001
6	0	6	7	7	←	5	0	5	6	7	4070.0837	0.0040
6	0	6	6	6	←	5	0	5	6	6	4070.2853	-0.0044
6	1	6	6	6	←	5	1	5	5	5	4070.4494	0.0012
6	1	6	7	8	←	5	1	5	6	7	4070.5499	0.0161
6	0	6	6	6	←	5	0	5	5	5	4070.8433	0.0008
6	0	6	7	8	←	5	0	5	6	7	4070.9430	0.0149
6	1	6	5	4	←	5	1	5	5	4	4071.1723	0.0049
6	1	6	6	5	←	5	1	5	5	5	4071.4432	0.0025
6	0	6	5	4	←	5	0	5	5	4	4071.5712	0.0097
5	4	2	6	6	←	4	4	1	5	5	4121.4614	-0.0068
5	4	2	6	7	←	4	4	1	5	6	4122.0381	0.0167
5	4	2	6	5	←	4	4	1	5	4	4122.1700	-0.0096
5	4	1	6	5	←	4	4	0	5	4	4190.2630	-0.0025
5	2	3	5	5	←	4	2	2	4	5	4244.6932	0.0052
5	2	3	5	5	←	4	2	2	4	4	4244.8565	-0.0010
5	2	3	5	6	←	4	2	2	4	5	4245.0322	-0.0039
5	2	3	6	6	←	4	2	2	5	5	4245.0969	0.0061
5	2	3	6	7	←	4	2	2	5	6	4245.1578	0.0073
5	2	3	4	5	←	4	2	2	3	4	4245.2365	-0.0142
5	2	3	5	6	←	4	2	2	5	6	4245.3825	0.0039
5	2	3	6	5	←	4	2	2	5	5	4245.4699	0.0011
5	3	2	6	6	←	4	3	1	5	5	4345.5713	-0.0101
5	3	2	5	5	←	4	3	1	4	4	4345.6393	0.0017
5	3	2	6	7	←	4	3	1	5	6	4345.8598	-0.0003
5	3	2	5	4	←	4	3	1	4	3	4345.9432	-0.0021
4	4	1	5	5	←	3	3	0	4	4	4418.9768	-0.0139
6	1	5	5	6	←	5	2	4	4	5	4442.3447	-0.0026
6	1	5	6	6	←	5	1	4	5	6	4462.3802	0.0027
6	1	5	7	7	←	5	1	4	6	7	4462.4799	0.0077
6	1	5	6	6	←	5	1	4	5	5	4462.7950	-0.0018
6	1	5	6	7	←	5	1	4	5	6	4462.8731	0.0145
6	1	5	7	8	←	5	1	4	6	7	4462.9612	-0.0015
6	2	5	7	8	←	5	1	4	6	7	4468.0128	0.0093
4	3	1	5	4	←	3	1	2	4	3	4560.2702	0.0100
4	3	1	3	4	←	3	1	2	2	3	4560.3699	-0.0028
4	3	1	5	6	←	3	1	2	4	5	4560.4360	0.0017
4	3	1	4	3	←	3	1	2	3	2	4560.5093	0.0014
4	3	1	4	5	←	3	1	2	3	4	4560.6506	0.0010
4	3	1	5	5	←	3	1	2	4	4	4560.9247	0.0080
4	3	1	4	4	←	3	1	2	3	3	4561.1684	-0.0023
6	2	4	6	6	←	5	3	3	6	5	4680.8627	-0.0080
7	1	7	8	8	←	6	1	6	7	8	4716.3706	-0.0035
7	0	7	8	8	←	6	0	6	7	8	4716.4561	0.0086
7	1	7	7	7	←	6	1	6	7	7	4716.5672	-0.0002
7	0	7	7	7	←	6	0	6	7	7	4716.6442	0.0033
7	1	7	7	7	←	6	1	6	6	6	4717.1862	0.0082
7	0	7	7	8	←	6	0	6	6	7	4717.2748	-0.0012
6	3	4	6	6	←	5	3	3	5	5	4771.1663	0.0014
6	3	4	7	7	←	5	3	3	6	6	4771.2590	-0.0001
6	3	4	6	7	←	5	3	3	5	6	4771.3224	-0.0097
6	3	4	7	8	←	5	3	3	6	7	4771.4204	0.0063
6	2	4	6	7	←	5	2	3	5	6	4911.4941	0.0047
6	4	3	6	6	←	5	4	2	5	5	4941.9127	0.0074
6	4	3	7	7	←	5	4	2	5	6	4942.1151	0.0076
6	4	3	7	8	←	5	4	2	6	7	4942.2403	0.0057
6	4	3	7	6	←	5	4	2	6	5	4942.2966	-0.0007

J'	K_a'	K_c'	I'	F'	←	J''	K_a''	K_c''	I''	F''	ν_{obs}	$\nu_{obs}-\nu_{calc}$
6	5	2	7	7	←	5	5	1	6	6	4957.9195	0.0022
6	5	2	7	8	←	5	5	1	6	7	4958.4234	-0.0030
6	5	2	7	6	←	5	5	1	6	5	4958.5438	-0.0016
6	5	1	7	7	←	5	5	0	6	6	4984.3766	-0.0078
6	5	1	7	8	←	5	5	0	6	7	4984.9041	-0.0041
6	5	1	7	6	←	5	5	0	6	5	4985.0283	-0.0018
6	3	4	7	7	←	5	2	3	6	6	5002.0389	0.0169
7	1	6	7	7	←	6	2	5	6	6	5097.2393	0.0028
7	1	6	8	9	←	6	2	5	7	8	5097.3747	0.0028
7	2	6	6	6	←	6	2	5	5	6	5097.9298	-0.0037
7	2	6	7	7	←	6	2	5	7	7	5098.0578	-0.0027
7	2	6	7	6	←	6	2	5	7	6	5098.1247	0.0019
7	2	6	7	7	←	6	2	5	6	6	5098.3425	-0.0015
7	2	6	7	8	←	6	2	5	6	7	5098.4079	0.0015
7	2	6	8	7	←	6	2	5	7	6	5098.4814	0.0048
7	2	6	6	5	←	6	2	5	6	5	5098.8223	0.0066
7	2	6	7	6	←	6	2	5	6	6	5098.9645	-0.0019
4	4	0	5	6	←	3	2	1	4	5	5099.3014	0.0006
7	1	6	6	6	←	6	1	5	5	6	5101.8660	-0.0004
7	1	6	7	7	←	6	1	5	7	7	5101.9871	-0.0123
7	1	6	8	9	←	6	1	5	7	8	5102.4094	-0.0031
7	1	6	6	5	←	6	1	5	6	5	5102.7553	0.0113
7	2	6	8	9	←	6	1	5	7	8	5103.5103	-0.0096
5	4	2	5	6	←	4	3	1	4	5	5134.8046	0.0007
5	4	2	6	7	←	4	3	1	5	6	5134.9584	-0.0107
5	4	2	6	6	←	4	3	1	5	5	5135.2245	-0.0040
6	4	2	7	7	←	5	4	1	6	6	5149.9265	0.0138
6	4	2	6	6	←	5	4	1	5	5	5150.0535	0.0109
6	4	2	7	8	←	5	4	1	6	7	5150.3072	0.0048
6	4	2	7	6	←	5	4	1	6	5	5150.3868	0.0161
6	4	2	6	7	←	5	4	1	6	7	5150.6932	0.0059
6	3	3	5	6	←	5	3	2	5	6	5216.3042	0.0036
6	3	3	7	7	←	5	3	2	6	6	5216.6490	-0.0139
6	3	3	7	8	←	5	3	2	6	7	5216.7793	0.0021
8	0	8	9	9	←	7	0	7	8	9	5362.9785	-0.0006
8	0	8	8	8	←	7	0	7	8	8	5363.1476	-0.0099
8	1	8	9	10	←	7	1	7	8	9	5363.8462	-0.0042
8	0	8	9	10	←	7	0	7	8	9	5363.8462	-0.0172
5	4	1	6	7	←	4	3	2	5	6	5386.4551	0.0095
5	4	1	5	6	←	4	3	2	4	5	5386.5778	0.0052
5	4	1	5	5	←	4	3	2	4	4	5386.7029	-0.0040
4	4	1	5	4	←	3	2	2	4	3	5392.2668	0.0041
4	4	1	5	6	←	3	2	2	4	5	5392.4955	-0.0004
4	4	1	4	5	←	3	2	2	3	4	5392.7254	0.0059
4	4	1	5	5	←	3	2	2	4	4	5393.4396	-0.0102
4	4	1	4	4	←	3	2	2	3	3	5393.6545	0.0021
7	2	5	7	7	←	6	3	4	6	6	5433.2983	-0.0011
7	2	5	8	9	←	6	3	4	7	8	5433.4914	0.0094
7	3	5	7	7	←	6	3	4	6	6	5460.9468	0.0029
7	3	5	7	8	←	6	3	4	6	7	5461.0384	0.0036
7	3	5	8	9	←	6	3	4	7	8	5461.1259	0.0055
7	2	5	7	8	←	6	2	4	6	7	5523.6570	0.0079
7	3	5	7	8	←	6	2	4	6	7	5551.2659	0.0000
5	5	1	5	6	←	4	4	0	4	5	5584.6678	0.0019
5	5	1	6	6	←	4	4	0	5	5	5584.7653	0.0041
5	5	0	6	7	←	4	4	1	5	6	5598.0309	-0.0047
5	5	0	6	6	←	4	4	1	5	5	5598.1044	-0.0086
5	3	2	6	7	←	4	1	3	5	6	5698.4235	0.0072
5	3	2	4	4	←	4	1	3	3	3	5698.5796	0.0073
5	3	2	6	6	←	4	1	3	5	5	5698.6601	0.0007
5	3	2	5	5	←	4	1	3	4	4	5699.1051	0.0028
7	4	4	8	8	←	6	4	3	7	7	5721.8435	-0.0105
6	4	3	7	8	←	5	3	2	6	7	5731.3481	0.0045
8	2	7	7	7	←	7	2	6	6	7	5745.3447	0.0001
8	2	7	8	8	←	7	2	6	8	8	5745.4828	0.0083
8	1	7	9	10	←	7	2	6	8	9	5745.7014	-0.0128
8	2	7	9	10	←	7	2	6	8	9	5745.9322	-0.0085
8	1	7	9	9	←	7	1	6	8	9	5746.2217	-0.0059

J'	K_a'	K_c'	I'	F'	←	J''	K_a''	K_c''	I''	F''	ν_{obs}	$\nu_{obs}-\nu_{calc}$
8	2	7	7	6	←	7	2	6	7	6	5746.3562	0.0056
8	1	7	7	8	←	7	1	6	6	7	5746.8120	-0.0111
8	1	7	8	7	←	7	1	6	7	7	5747.3685	-0.0116
7	6	2	8	8	←	6	6	1	7	7	5783.5332	0.0009
7	6	2	8	9	←	6	6	1	7	8	5783.9973	-0.0017
7	6	2	8	7	←	6	6	1	7	6	5784.0914	-0.0001
7	6	1	8	8	←	6	6	0	7	7	5792.5486	0.0036
7	6	1	7	8	←	6	6	0	6	7	5793.0216	-0.0029
7	6	1	8	7	←	6	6	0	7	6	5793.1100	-0.0007
7	5	3	7	8	←	6	5	2	7	7	5807.8874	-0.0071
7	5	3	8	9	←	6	5	2	7	8	5808.1769	0.0068
5	4	1	5	4	←	4	2	2	4	3	5821.3571	0.0000
5	4	1	6	7	←	4	2	2	5	6	5821.5771	-0.0013
5	4	1	4	5	←	4	2	2	3	4	5821.6330	-0.0084
5	4	1	6	6	←	4	2	2	5	5	5822.2052	0.0113
7	5	2	8	8	←	6	5	1	7	7	5919.0593	-0.0006
7	5	2	7	7	←	6	5	1	6	6	5919.1478	0.0031
7	5	2	8	9	←	6	5	1	7	8	5919.4290	-0.0018
7	5	2	8	7	←	6	5	1	7	6	5919.4968	0.0042
7	5	2	7	6	←	6	5	1	6	5	5919.5879	0.0057
7	3	4	7	8	←	6	3	3	6	7	5972.6728	-0.0036
7	3	4	6	6	←	6	3	3	5	5	5972.7886	0.0101
9	0	9	8	9	←	8	0	8	7	8	6010.4420	0.0013
7	4	3	8	8	←	6	4	2	7	7	6104.9924	0.0172
7	4	3	8	9	←	6	4	2	7	8	6105.1454	-0.0091
7	4	3	7	7	←	6	4	2	7	7	6105.3851	-0.0171
8	2	6	8	9	←	7	3	5	7	8	6117.0605	0.0005
8	2	6	9	10	←	7	3	5	8	9	6117.1448	0.0104
8	3	6	8	8	←	7	3	5	7	7	6124.2180	-0.0036
8	3	6	8	9	←	7	3	5	7	8	6124.2901	0.0035
8	3	6	9	10	←	7	3	5	8	9	6124.3743	0.0078
8	2	6	8	9	←	7	2	5	7	8	6144.6770	0.0002
8	2	6	9	10	←	7	2	5	8	9	6144.7665	-0.0062
8	3	6	8	9	←	7	2	5	7	8	6151.9015	-0.0019
8	3	6	9	10	←	7	2	5	8	9	6152.0013	-0.0035
9	2	8	10	9	←	8	2	7	9	8	6392.5131	-0.0099
9	1	8	8	9	←	8	1	7	7	8	6392.6948	-0.0141
5	4	2	4	3	←	4	2	3	3	2	6416.5816	-0.0079
5	4	2	6	5	←	4	2	3	5	4	6416.6686	-0.0017
5	4	2	6	7	←	4	2	3	5	6	6416.8071	-0.0011
5	4	2	5	4	←	4	2	3	4	3	6416.9673	0.0110
5	4	2	5	6	←	4	2	3	4	5	6417.1247	0.0072
5	4	2	4	4	←	4	2	3	3	3	6417.4030	-0.0144
5	4	2	6	6	←	4	2	3	5	5	6417.4737	-0.0011
5	4	2	5	5	←	4	2	3	4	4	6417.7631	-0.0021
8	4	5	8	8	←	7	4	4	7	7	6452.7902	0.0097
6	5	1	7	8	←	5	4	2	6	7	6460.9204	-0.0021
6	4	2	7	8	←	5	3	3	6	7	6496.2066	0.0043
6	4	2	6	7	←	5	3	3	5	6	6496.5075	0.0085
8	7	2	9	9	←	7	7	1	8	8	6604.8150	-0.0112
8	7	2	9	10	←	7	7	1	8	9	6605.2483	-0.0061
8	7	1	9	9	←	7	7	0	8	8	6607.6458	0.0001
8	7	1	9	10	←	7	7	0	8	9	6608.0803	0.0045
8	7	1	8	7	←	7	7	0	7	6	6608.1651	0.0128
8	3	5	7	7	←	7	3	4	6	7	6613.3934	-0.0030
8	3	5	8	9	←	7	3	4	7	8	6613.4879	0.0050
8	3	5	9	10	←	7	3	4	8	9	6613.6192	0.0038
8	5	4	9	9	←	7	5	3	8	8	6632.0616	-0.0013
8	5	4	9	10	←	7	5	3	8	9	6632.2348	-0.0098
8	6	3	9	9	←	7	6	2	8	8	6648.5660	0.0013
8	6	3	7	8	←	7	6	2	6	7	6648.8862	0.0133
10	1	10	11	10	←	9	1	9	10	9	6657.0362	0.0041
10	1	10	9	10	←	9	1	9	8	9	6657.0362	0.0023
10	0	10	11	10	←	9	0	9	10	9	6657.0362	0.0037
10	0	10	9	10	←	9	0	9	8	9	6657.0362	0.0019
8	6	2	9	9	←	7	6	1	8	8	6696.7342	-0.0085
8	6	2	8	8	←	7	6	1	7	7	6696.8161	0.0011
8	6	2	9	10	←	7	6	1	8	9	6697.1237	0.0141

J'	K_a'	K_c'	I'	F'	←	J''	K_a''	K_c''	I''	F''	ν_{obs}	$\nu_{obs}-\nu_{calc}$
8	6	2	8	7	←	7	6	1	7	6	6697.2443	0.0009
8	4	5	9	10	←	7	3	4	8	9	6716.7744	-0.0053
6	4	2	7	6	←	5	2	3	6	5	6726.6452	-0.0044
6	4	2	7	8	←	5	2	3	6	7	6726.7274	-0.0029
6	4	2	6	7	←	5	2	3	5	6	6726.8839	-0.0033
6	4	2	6	6	←	5	2	3	5	5	6727.2233	-0.0024
6	6	1	7	8	←	5	5	0	6	7	6741.1653	-0.0174
6	6	1	7	7	←	5	5	0	6	6	6741.2449	-0.0016
6	6	0	7	8	←	5	5	1	6	7	6745.0696	-0.0054
6	6	0	7	7	←	5	5	1	6	6	6745.1371	0.0019
9	2	7	10	9	←	8	3	6	9	8	6773.7772	-0.0054
9	2	7	8	9	←	8	3	6	7	8	6773.7772	-0.0104
9	3	7	10	9	←	8	3	6	9	8	6775.4933	0.0007
9	3	7	8	9	←	8	3	6	7	8	6775.4933	-0.0044
9	2	7	10	9	←	8	2	6	9	8	6781.0142	0.0002
9	2	7	8	9	←	8	2	6	7	8	6781.0142	-0.0061
9	3	7	10	9	←	8	2	6	9	8	6782.7188	-0.0050
9	3	7	8	9	←	8	2	6	7	8	6782.7188	-0.0118
8	4	4	8	8	←	7	4	3	7	7	6967.2929	0.0001
8	4	4	9	10	←	7	4	3	8	9	6967.3917	0.0013
10	1	9	9	9	←	9	1	8	8	8	7038.9796	0.0001
10	2	9	11	10	←	9	2	8	10	9	7038.9796	0.0063
10	2	9	9	10	←	9	2	8	8	9	7038.9796	0.0039
9	3	6	10	9	←	8	4	5	9	8	7109.2080	0.0026
9	3	6	8	7	←	8	4	5	7	6	7109.2080	-0.0056
9	4	6	9	9	←	8	4	5	8	8	7141.6065	0.0016
9	4	6	9	10	←	8	4	5	8	9	7141.6770	0.0066
9	4	6	10	9	←	8	4	5	9	8	7141.7539	0.0071
9	4	6	8	9	←	8	4	5	7	8	7141.7539	0.0029
9	3	6	9	10	←	8	3	5	8	9	7212.2511	-0.0007
9	4	6	9	8	←	8	3	5	8	7	7244.7799	0.0076
9	4	6	10	10	←	8	3	5	9	9	7244.9151	0.0013
6	5	1	6	5	←	5	3	2	5	4	7249.7741	-0.0021
6	5	1	7	8	←	5	3	2	6	7	7250.0430	0.0113
6	5	1	6	6	←	5	3	2	5	5	7250.5063	-0.0072
6	5	1	7	7	←	5	3	2	6	6	7250.6823	0.0059
11	1	11	10	10	←	10	1	10	9	9	7303.6263	0.0045
11	0	11	10	10	←	10	0	10	9	9	7303.6263	0.0045
9	5	5	10	10	←	8	5	4	9	9	7411.2645	0.0021
10	2	8	11	10	←	9	3	7	10	9	7422.2104	-0.0047
10	2	8	9	10	←	9	3	7	8	9	7422.2104	-0.0086
10	3	8	9	10	←	9	3	7	8	9	7422.5876	-0.0078
10	2	8	11	10	←	9	2	7	10	9	7423.9237	-0.0013
10	2	8	9	10	←	9	2	7	8	9	7423.9237	-0.0055
10	3	8	10	10	←	9	2	7	9	9	7424.2140	0.0158
9	8	2	10	10	←	8	8	1	9	9	7424.7785	-0.0050
9	8	2	9	10	←	8	8	1	8	9	7425.1732	-0.0023
9	8	1	10	10	←	8	8	0	9	9	7425.6095	-0.0057
9	8	1	9	10	←	8	8	0	8	9	7426.0126	0.0041
7	5	2	7	8	←	6	4	3	6	7	7438.2577	-0.0100
9	7	3	10	10	←	8	7	2	9	9	7475.0984	0.0032
9	7	3	9	10	←	8	7	2	8	9	7475.4081	0.0062
9	7	2	10	10	←	8	7	1	9	9	7493.2251	0.0031
9	7	2	9	10	←	8	7	1	8	9	7493.5401	-0.0069
9	6	4	9	9	←	8	6	3	8	8	7504.7981	-0.0102
9	6	4	8	9	←	8	6	3	7	8	7505.0127	-0.0096
7	6	2	8	9	←	6	5	1	7	8	7540.2730	-0.0005
7	6	2	8	8	←	6	5	1	7	7	7540.3978	0.0034
6	4	3	7	8	←	5	2	4	6	7	7574.0127	0.0086
6	4	3	6	5	←	5	2	4	5	4	7574.3039	0.0033
6	4	3	6	7	←	5	2	4	5	6	7574.4017	0.0013
6	4	3	6	6	←	5	2	4	5	5	7574.8650	-0.0010
7	6	1	8	9	←	6	5	2	7	8	7579.6599	-0.0061
7	6	1	8	8	←	6	5	2	7	7	7579.7546	-0.0081
11	2	10	10	10	←	10	2	9	9	9	7685.3999	-0.0120
11	1	10	10	9	←	10	1	9	9	8	7685.4552	0.0134
9	4	5	9	10	←	8	4	4	8	9	7697.2047	-0.0016
9	4	5	10	10	←	8	4	4	9	9	7697.3116	0.0133

J'	K_a'	K_c'	I'	F'	←	J''	K_a''	K_c''	I''	F''	ν_{obs}	$\nu_{obs}-\nu_{calc}$
10	3	7	11	10	←	9	4	6	10	9	7795.1326	-0.0010
10	3	7	9	10	←	9	4	6	8	9	7795.1326	-0.0048
10	4	7	9	8	←	9	4	6	8	7	7804.0544	-0.0073
10	3	7	9	10	←	9	3	6	8	9	7827.6856	0.0020
10	4	7	11	10	←	9	3	6	10	9	7836.6128	0.0174
10	4	7	9	10	←	9	3	6	8	9	7836.6128	0.0075
9	5	4	10	10	←	8	5	3	9	9	7870.3430	-0.0017
9	5	4	9	10	←	8	5	3	8	9	7870.4757	-0.0142
7	7	1	8	9	←	6	6	0	7	8	7894.2332	0.0088
7	7	0	8	9	←	6	6	1	7	8	7895.2989	0.0092
12	1	12	11	10	←	11	1	11	10	9	7950.2147	0.0001
12	0	12	11	10	←	11	0	11	10	9	7950.2147	0.0001

Table S27. Measured rotational transitions (ν_{obs}) of the ^{13}C (1,3) isotopic species of the $(Py)_2$-Bz trimer and residuals (ν_{obs} - ν_{calc}) (frequencies in MHz).

J'	*K*a'	*K*c'	*I'*	*F'*	←	*J''*	*K*a''	*K*c''	*I''*	*F''*	ν_{obs}	ν_{obs}- ν_{calc}
4	1	4	5	6	←	3	1	3	4	5	2764.7979	0.0040
4	0	4	5	6	←	3	0	3	4	5	2772.8981	-0.0099
4	1	3	4	5	←	3	1	2	3	4	3196.1860	0.0097
5	1	5	5	5	←	4	1	4	4	4	3412.1290	0.0113
5	0	5	6	7	←	4	0	4	5	6	3413.9944	-0.0119
5	2	4	5	5	←	4	2	3	4	4	3773.5769	0.0112
5	2	4	4	4	←	4	2	3	4	4	3773.7901	-0.0077
6	1	6	6	6	←	5	1	5	5	5	4057.2859	-0.0062
6	1	6	7	8	←	5	1	5	6	7	4057.3640	-0.0137
6	0	6	6	6	←	5	0	5	5	5	4057.6352	-0.0005
5	2	3	6	7	←	4	2	2	5	6	4233.1774	-0.0144
6	2	5	6	6	←	5	2	4	5	5	4432.9348	-0.0045
6	2	5	6	7	←	5	2	4	5	6	4433.0297	0.0057
6	2	5	7	8	←	5	2	4	6	7	4433.1174	0.0052
7	0	7	7	8	←	6	0	6	6	7	4702.0263	-0.0101
7	2	6	7	7	←	6	2	5	6	6	5081.5747	-0.0109
7	2	6	8	9	←	6	2	5	7	8	5081.7307	0.0099
7	1	6	7	6	←	6	1	5	6	5	5085.0902	0.0062
8	1	8	9	10	←	7	1	7	8	9	5346.5506	-0.0123
7	3	5	8	8	←	6	3	4	7	7	5444.5495	-0.0029
7	3	5	8	9	←	6	3	4	7	8	5444.6416	-0.0038
7	4	4	8	9	←	6	4	3	7	8	5710.4735	0.0025
8	2	7	7	8	←	7	2	6	6	7	5726.9926	-0.0009
9	0	9	10	10	←	8	0	8	9	9	5991.0979	0.0072
8	3	6	9	10	←	7	3	5	8	9	6104.4515	0.0166
9	2	8	8	9	←	8	2	7	7	8	6371.4922	-0.0030
8	3	5	9	10	←	7	3	4	8	9	6585.9682	-0.0089
10	0	10	11	10	←	9	0	9	10	9	6635.6335	0.0014
10	0	10	9	10	←	9	0	9	8	9	6635.6335	-0.0003
10	1	10	11	10	←	9	1	9	10	9	6635.6335	0.0017
10	1	10	9	10	←	9	1	9	8	9	6635.6335	-0.0001
10	2	9	9	10	←	9	2	8	8	9	7015.8935	0.0037
9	4	6	9	10	←	8	4	5	8	9	7119.6912	-0.0075
9	4	6	8	9	←	8	4	5	7	8	7119.7757	-0.0046
11	0	11	10	10	←	10	0	10	9	9	7280.1705	0.0054
11	1	11	10	10	←	10	1	10	9	9	7280.1705	0.0055
10	3	8	11	10	←	9	3	7	10	9	7397.7881	0.0167
10	3	8	9	10	←	9	3	7	8	9	7397.7881	0.0129
10	2	8	11	10	←	9	2	7	10	9	7398.8891	0.0119
11	2	10	10	10	←	10	2	9	9	9	7660.2729	-0.0026
12	0	12	11	10	←	11	0	11	10	9	7924.6984	-0.0028
12	1	12	11	10	←	11	1	11	10	9	7924.6984	-0.0028

Table S28. Measured rotational transitions (ν_{obs}) of the ^{13}C (4,5) isotopic species of the $(Py)_2$-Bz trimer and residuals (ν_{obs} - ν_{calc}) (frequencies in MHz).

J′	*K*a′	*K*c′	*I′*	*F′*	←	*J″*	*K*a″	*K*c″	*I″*	*F″*	ν_{obs}	ν_{obs}- ν_{calc}
3	0	3	4	5	←	2	0	2	3	4	2132.0479	-0.0097
4	0	4	5	6	←	3	0	3	4	5	2764.2995	-0.0028
5	1	5	5	5	←	4	1	4	4	4	3401.7038	-0.0077
5	0	5	5	5	←	4	0	4	4	4	3403.3498	0.0164
6	1	6	6	6	←	5	1	5	5	5	4044.6933	-0.0097
6	1	6	7	8	←	5	1	5	6	7	4044.7855	-0.0032
6	2	5	6	6	←	5	2	4	5	5	4421.1411	-0.0113
6	2	5	5	4	←	5	2	4	4	3	4421.3447	0.0115
7	0	7	7	7	←	6	0	6	6	6	4687.2635	-0.0068
7	1	6	8	9	←	6	1	5	7	8	5070.5602	-0.0053
8	1	8	9	10	←	7	1	7	8	9	5329.6801	-0.0148
8	2	7	8	9	←	7	2	6	7	8	5710.4734	0.0133
9	0	9	9	10	←	8	0	8	8	9	5972.0868	0.0114
8	3	6	8	8	←	7	3	5	7	7	6088.7247	0.0150
8	3	6	9	10	←	7	3	5	8	9	6088.8463	-0.0080
7	4	3	8	8	←	6	4	2	7	7	6103.3983	-0.0048
9	2	8	9	10	←	8	2	7	8	9	6352.8189	-0.0033
9	1	8	9	10	←	8	1	7	8	9	6352.9523	0.0015
8	3	5	8	9	←	7	3	4	7	8	6565.9767	-0.0145
10	0	10	11	10	←	9	0	9	10	9	6614.4924	-0.0061
10	0	10	9	10	←	9	0	9	8	9	6614.4924	-0.0079
9	3	7	9	10	←	8	3	6	8	9	6734.6767	0.0019
9	2	7	9	10	←	8	2	6	8	9	6738.8125	-0.0054
9	2	7	10	9	←	8	2	6	9	8	6738.8961	0.0055
9	2	7	8	9	←	8	2	6	7	8	6738.8961	-0.0004
10	1	9	10	10	←	9	1	8	9	9	6995.0841	-0.0006
10	1	9	11	10	←	9	1	8	10	9	6995.1579	0.0017
11	0	11	10	10	←	10	0	10	9	9	7256.8963	-0.0023
10	2	8	9	9	←	9	2	7	8	8	7378.2777	0.0153
11	2	10	10	10	←	10	2	9	9	9	7637.4004	0.0084
11	1	10	10	10	←	10	1	9	9	9	7637.3997	0.0034
10	4	7	9	9	←	9	4	6	9	9	7758.7332	0.0036
12	0	12	11	10	←	11	0	11	10	9	7899.2978	-0.0041

Table S29. Measured rotational transitions (ν_{obs}) of the ^{13}C (6,10) isotopic species of the $(Py)_2$-Bz trimer and residuals (ν_{obs} - ν_{calc}) (frequencies in MHz).

J'	*K*a'	*K*c'	*I'*	*F'*	←	*J''*	*K*a''	*K*c''	*I''*	*F''*	ν_{obs}	ν_{obs}- ν_{calc}
4	1	4	5	6	←	3	1	3	4	5	2765.2522	0.0007
5	0	5	5	5	←	4	0	4	4	4	3414.9792	0.0006
6	1	6	6	6	←	5	1	5	5	5	4058.4730	-0.0046
6	0	6	7	8	←	5	0	5	6	7	4058.9696	0.0131
7	0	7	8	9	←	6	0	6	7	8	4703.5021	-0.0142
6	2	4	6	7	←	5	2	3	5	6	4895.1593	-0.0130
8	1	8	9	10	←	7	1	7	8	9	5348.2095	-0.0120
7	3	5	7	7	←	6	3	4	6	6	5443.2266	0.0031
7	3	4	7	8	←	6	3	3	6	7	5952.2780	-0.0111
9	1	9	9	10	←	8	1	8	8	9	5992.9746	0.0139
9	0	9	9	10	←	8	0	8	8	9	5992.9746	0.0117
9	2	8	9	10	←	8	2	7	8	9	6373.0240	-0.0016
9	1	8	9	10	←	8	1	7	8	9	6373.2153	0.0076
8	3	5	8	9	←	7	3	4	7	8	6591.4211	-0.0010
10	1	10	11	10	←	9	1	9	10	9	6637.7444	-0.0005
10	1	10	9	10	←	9	1	9	8	9	6637.7444	-0.0023
10	0	10	11	10	←	9	0	9	10	9	6637.7444	-0.0009
10	0	10	9	10	←	9	0	9	8	9	6637.7444	-0.0027
9	3	7	9	10	←	8	3	6	8	9	6753.9820	0.0117
8	5	3	9	10	←	7	5	2	8	9	6885.3341	0.0010
9	3	6	9	10	←	8	3	5	8	9	7188.6226	0.0081
10	3	8	9	9	←	9	3	7	8	8	7399.2558	-0.0146
10	2	8	10	9	←	9	2	7	9	8	7400.5879	0.0064
11	2	10	10	10	←	10	2	9	9	9	7662.3161	0.0126
10	3	7	9	9	←	9	3	6	8	8	7802.3065	0.0029
12	1	12	11	10	←	11	1	11	10	9	7927.2570	-0.0122
12	0	12	11	10	←	11	0	11	10	9	7927.2570	-0.0122

Table S30. Measured rotational transitions (ν_{obs}) of the ^{13}C (7,9) isotopic species of the $(Py)_2$-Bz trimer and residuals (ν_{obs} - ν_{calc}) (frequencies in MHz).

J'	*K*a'	*K*c'	*I'*	*F'*	←	*J''*	*K*a''	*K*c''	*I''*	*F''*	ν_{obs}	ν_{obs}-ν_{calc}
4	1	4	5	6	←	3	1	3	4	5	2757.7072	0.0070
5	1	5	5	5	←	4	1	4	4	4	3403.9801	0.0066
5	1	5	6	7	←	4	1	4	5	6	3404.0815	-0.0104
4	2	2	5	6	←	3	2	1	4	5	3439.6698	0.0114
5	1	4	6	7	←	4	1	3	5	6	3814.9654	-0.0128
5	3	3	6	7	←	4	3	2	5	6	4010.4162	-0.0101
5	3	2	6	7	←	4	3	1	5	6	4305.5315	-0.0137
6	2	4	6	5	←	5	2	3	5	4	4885.1871	-0.0052
6	4	3	7	8	←	5	4	2	6	7	4902.2027	0.0023
6	3	3	7	6	←	5	3	2	6	5	5175.1835	0.0008
8	1	8	9	10	←	7	1	7	8	9	5334.1448	0.0040
8	0	8	9	10	←	7	0	7	8	9	5334.1448	-0.0118
7	3	5	8	9	←	6	3	4	7	8	5427.2235	-0.0119
7	2	5	8	9	←	6	2	4	7	8	5495.3894	-0.0089
7	3	4	8	9	←	6	3	3	7	8	5935.1128	0.0133
9	0	9	10	10	←	8	0	8	9	9	5977.1929	0.0099
8	2	6	9	10	←	7	2	5	8	9	6111.4606	0.0134
8	4	5	9	10	←	7	4	4	8	9	6409.4914	-0.0094
10	1	10	11	10	←	9	1	9	10	9	6620.2404	0.0045
10	1	10	9	10	←	9	1	9	8	9	6620.2404	0.0027
10	0	10	11	10	←	9	0	9	10	9	6620.2404	0.0040
10	0	10	9	10	←	9	0	9	8	9	6620.2404	0.0022
8	4	4	9	10	←	7	4	3	8	9	6913.5293	0.0114
11	1	11	10	10	←	10	1	10	9	9	7263.2857	0.0035
11	0	11	10	10	←	10	0	10	9	9	7263.2857	0.0034
12	1	12	11	10	←	11	1	11	10	9	7906.3267	-0.0051
12	0	12	11	10	←	11	0	11	10	9	7906.3267	-0.0051

Table S31. Measured rotational transitions (ν_{obs}) of the ^{13}C (8) isotopic species of the $(Py)_2$-Bz trimer and residuals (ν_{obs} - ν_{calc}) (frequencies in MHz).

J′	*K*a′	*K*c′	*I′*	*F′*	←	*J″*	*K*a″	*K*c″	*I″*	*F″*	ν_{obs}	ν_{obs}-ν_{calc}
5	0	5	5	5	←	4	0	4	4	4	3399.5702	0.0082
5	4	1	6	7	←	4	4	0	5	6	4137.0833	-0.0149
5	4	1	6	5	←	4	4	0	5	4	4137.2496	-0.0115
7	1	7	7	7	←	6	1	6	6	6	4681.4262	-0.0058
7	0	7	7	8	←	6	0	6	6	7	4681.5498	-0.0022
7	1	6	8	9	←	6	1	5	7	8	5065.6217	-0.0017
8	1	8	9	10	←	7	1	7	8	9	5323.1150	-0.0084
8	2	7	8	8	←	7	2	6	7	7	5703.3998	-0.0027
8	2	7	9	8	←	7	2	6	8	7	5703.4909	-0.0171
8	1	7	8	8	←	7	1	6	7	7	5704.5521	0.0120
7	6	2	8	8	←	6	6	1	7	7	5716.8676	0.0021
9	1	9	9	10	←	8	1	8	8	9	5964.7224	0.0170
9	0	9	9	10	←	8	0	8	8	9	5964.7214	0.0128
8	3	6	9	9	←	7	3	5	8	8	6078.9012	0.0068
9	2	8	9	10	←	8	2	7	8	9	6345.1349	0.0177
9	1	8	9	10	←	8	1	7	8	9	6345.3798	0.0156
8	7	1	9	10	←	7	7	0	8	9	6531.1721	0.0000
10	1	10	11	10	←	9	1	9	10	9	6606.3327	0.0007
10	1	10	9	10	←	9	1	9	8	9	6606.3327	-0.0011
10	0	10	11	10	←	9	0	9	10	9	6606.3315	-0.0009
10	0	10	9	10	←	9	0	9	8	9	6606.3315	-0.0028
9	2	7	9	10	←	8	2	6	8	9	6733.1638	0.0025
9	2	7	8	9	←	8	2	6	7	8	6733.2435	0.0005
8	5	3	9	9	←	7	5	2	8	8	6815.0363	0.0080
8	4	4	7	8	←	7	4	3	6	7	6899.9318	0.0104
10	2	9	11	10	←	9	2	8	10	9	6986.6130	-0.0158
9	4	6	8	9	←	8	4	5	8	9	7086.5369	-0.0082
11	1	11	10	10	←	10	1	10	9	9	7247.9366	0.0017
11	0	11	10	10	←	10	0	10	9	9	7247.9362	0.0012
10	2	8	9	10	←	9	2	7	8	9	7370.4205	-0.0189
11	1	10	10	10	←	10	1	9	9	9	7628.0887	0.0061
12	1	12	11	10	←	11	1	11	10	9	7889.5340	-0.0072
12	0	12	11	10	←	11	0	11	10	9	7889.5333	-0.0079

Table S32. Measured rotational transitions (ν_{obs}) of the ^{13}C (11) isotopic species of the $(Py)_2$-Bz trimer and residuals (ν_{obs} - ν_{calc}) (frequencies in MHz).

J′	*K*a′	*K*c′	*I′*	*F′*	←	*J″*	*K*a″	*K*c″	*I″*	*F″*	ν_{obs}	ν_{obs}- ν_{calc}
4	2	2	5	5	←	3	2	1	4	4	3464.5307	-0.0030
4	2	2	5	6	←	3	2	1	4	5	3464.7428	0.0044
7	0	7	7	8	←	6	0	6	6	7	4706.2900	0.0039
6	2	4	6	7	←	5	2	3	5	6	4900.6326	-0.0134
6	2	4	7	8	←	5	2	3	6	7	4900.8166	0.0149
8	0	8	8	9	←	7	0	7	7	8	5351.2766	-0.0070
8	1	8	8	9	←	7	1	7	7	8	5351.2766	0.0048
7	3	5	6	6	←	6	3	4	5	5	5450.8463	-0.0030
9	1	9	9	10	←	8	1	8	8	9	5996.3148	0.0033
9	0	9	9	10	←	8	0	8	8	9	5996.3148	0.0013
10	1	10	11	10	←	9	1	9	10	9	6641.3612	-0.0029
10	1	10	9	10	←	9	1	9	8	9	6641.3612	-0.0047
10	0	10	11	10	←	9	0	9	10	9	6641.3612	-0.0032
10	0	10	9	10	←	9	0	9	8	9	6641.3612	-0.0051
11	1	11	10	10	←	10	1	10	9	9	7286.3825	-0.0106
11	0	11	10	10	←	10	0	10	9	9	7286.3825	-0.0107
12	1	12	11	10	←	11	1	11	10	9	7931.4357	0.0104
12	0	12	11	10	←	11	0	11	10	9	7931.4357	0.0104

Table S33. Measured rotational transitions (ν_{obs}) of the ^{13}C (23,27) isotopic species of the $(Py)_2$-Bz trimer and residuals (ν_{obs} - ν_{calc}) (frequencies in MHz).

J'	*K*a'	*K*c'	*I'*	*F'*	←	*J''*	*K*a''	*K*c''	*I''*	*F''*	ν_{obs}	ν_{obs}-ν_{calc}
4	0	4	4	4	←	3	0	3	3	3	2771.3682	0.0053
4	1	3	4	5	←	3	1	2	3	4	3194.6335	0.0157
4	1	3	5	6	←	3	1	2	4	5	3194.7923	-0.0113
5	1	5	5	5	←	4	1	4	4	4	3409.6216	0.0133
5	1	5	6	7	←	4	1	4	5	6	3409.7247	-0.0020
5	0	5	5	5	←	4	0	4	4	4	3411.7190	0.0135
5	0	5	6	7	←	4	0	4	5	6	3411.8144	-0.0084
4	2	2	5	6	←	3	2	1	4	5	3448.6359	0.0093
4	2	2	5	5	←	3	2	1	4	4	3448.4122	-0.0064
5	1	4	6	7	←	4	1	3	5	6	3820.4512	0.0065
6	1	6	6	6	←	5	1	5	5	5	4054.4639	0.0035
6	1	6	7	8	←	5	1	5	6	7	4054.5517	0.0056
6	0	6	6	6	←	5	0	5	5	5	4054.8889	0.0019
6	2	5	7	8	←	5	2	4	6	7	4428.9363	-0.0153
6	1	5	7	7	←	5	1	4	6	6	4445.3589	-0.0055
7	0	7	8	9	←	6	0	6	7	8	4698.8453	-0.0137
7	1	6	8	9	←	6	1	5	7	8	5082.0685	0.0129
8	1	8	9	10	←	7	1	7	8	9	5342.8955	-0.0048
8	2	7	9	10	←	7	2	6	8	9	5722.8653	-0.0098
8	1	7	9	10	←	7	1	6	8	9	5723.8277	-0.0149
9	0	9	9	10	←	8	0	8	8	9	5987.0010	0.0171
10	1	10	11	10	←	9	1	9	10	9	6631.1096	0.0022
10	1	10	9	10	←	9	1	9	8	9	6631.1096	0.0004
10	0	10	11	10	←	9	0	9	10	9	6631.1086	0.0008
10	0	10	9	10	←	9	0	9	8	9	6631.1086	-0.0010
9	2	7	9	10	←	8	2	6	8	9	6753.7214	-0.0015
10	2	9	11	10	←	9	2	8	10	9	7010.9535	0.0003
10	2	9	9	10	←	9	2	8	8	9	7010.9535	-0.0021
9	4	6	9	10	←	8	4	5	8	9	7110.3963	0.0008
11	1	11	10	10	←	10	1	10	9	9	7275.2118	0.0021
11	0	11	10	10	←	10	0	10	9	9	7275.2113	0.0015
10	3	8	9	10	←	9	3	7	8	9	7392.4906	-0.0018
10	2	8	9	10	←	9	2	7	8	9	7393.9868	0.0071
12	1	12	11	10	←	11	1	11	10	9	7919.3109	-0.0043
12	0	12	11	10	←	11	0	11	10	9	7919.3104	-0.0048

Table S34. Measured rotational transitions (ν_{obs}) of the ^{13}C (25,26) isotopic species of the $(Py)_2$-Bz trimer and residuals (ν_{obs} - ν_{calc}) (frequencies in MHz).

J'	K_a'	K_c'	I'	F'	←	J''	K_a''	K_c''	I''	F''	ν_{obs}	ν_{obs}- ν_{calc}
4	1	4	5	6	←	3	1	3	4	5	2753.3939	0.0053
4	0	4	5	6	←	3	0	3	4	5	2762.2682	-0.0134
5	0	5	5	5	←	4	0	4	4	4	3400.2403	0.0039
4	2	2	5	6	←	3	2	1	4	5	3442.8214	-0.0039
5	1	4	5	6	←	4	1	3	4	5	3808.5151	0.0133
6	1	6	6	6	←	5	1	5	5	5	4040.7192	-0.0153
6	1	6	7	8	←	5	1	5	6	7	4040.8296	0.0094
6	0	6	6	5	←	5	0	5	5	4	4041.1434	0.0010
6	2	5	7	8	←	5	2	4	6	7	4415.7324	-0.0085
7	1	7	7	7	←	6	1	6	6	6	4682.6722	0.0044
7	0	7	8	9	←	6	0	6	7	8	4682.8154	0.0069
7	2	6	7	6	←	6	2	5	6	5	5062.0372	-0.0046
7	2	6	8	9	←	6	2	5	7	8	5062.1060	-0.0122
7	1	6	7	7	←	6	1	5	6	6	5066.0018	-0.0147
8	1	8	9	10	←	7	1	7	8	9	5324.5366	-0.0041
7	3	5	7	8	←	6	3	4	6	7	5422.5053	0.0124
7	3	4	7	8	←	6	3	3	6	7	5931.7319	0.0092
8	3	6	8	9	←	7	3	5	7	8	6081.1764	-0.0064
8	3	6	9	10	←	7	3	5	8	9	6081.2665	0.0038
9	2	8	9	10	←	8	2	7	8	9	6346.5588	0.0079
10	1	10	10	10	←	9	1	9	9	9	6608.0803	-0.0158
10	1	9	10	9	←	9	1	8	9	8	6988.2351	-0.0073
11	1	11	10	10	←	10	1	10	9	9	7249.9277	0.0159
11	0	11	10	10	←	10	0	10	9	9	7249.9277	0.0159
10	3	8	9	9	←	9	3	7	8	8	7370.0131	0.0029
10	2	8	9	9	←	9	2	7	8	8	7371.3865	-0.0093
12	1	12	11	10	←	11	1	11	10	9	7891.7158	0.0113
12	0	12	11	10	←	11	0	11	10	9	7891.7158	0.0112

Table S35. Measured rotational transitions (ν_{obs}) of the D (12) isotopic species of the $(Py)_2$-Bz trimer and residuals (ν_{obs} - ν_{calc}) (frequencies in MHz).

J′	*K*a′	*K*c′	*I′*	*F′*	←	*J″*	*K*a″	*K*c″	*I″*	*F″*	ν_{obs}	ν_{obs}-ν_{calc}
3	1	3	4	5	←	2	1	2	3	4	2087.9867	-0.0035
3	2	2	3	4	←	2	2	1	3	4	2335.1963	0.0081
4	1	4	4	4	←	3	1	3	3	3	2739.0414	-0.0070
4	1	4	5	5	←	3	1	3	4	4	2739.1618	0.0043
4	1	4	5	6	←	3	1	3	4	5	2739.2407	0.0152
5	1	5	5	5	←	4	1	4	4	4	3381.4332	0.0063
5	0	5	6	6	←	4	0	4	5	5	3384.1293	0.0074
4	2	2	5	5	←	3	2	1	4	4	3408.9961	-0.0046
4	2	2	5	6	←	3	2	1	4	5	3409.2202	0.0056
5	2	4	5	5	←	4	2	3	4	4	3736.1833	-0.0035
5	2	4	6	7	←	4	2	3	5	6	3736.4342	0.0133
5	1	4	5	6	←	4	1	3	4	5	3795.3963	-0.0072
5	1	4	6	6	←	4	1	3	5	5	3795.5321	0.0174
5	3	3	6	6	←	4	3	2	5	5	3977.0055	-0.0149
6	1	6	6	6	←	5	1	5	5	5	4020.9185	-0.0047
6	0	6	6	6	←	5	0	5	5	5	4021.4851	-0.0044
5	2	3	6	7	←	4	2	2	5	6	4188.1161	-0.0088
6	2	5	6	6	←	5	2	4	5	5	4393.1796	0.0113
6	2	5	6	7	←	5	2	4	5	6	4393.2517	-0.0014
6	2	5	7	8	←	5	2	4	6	7	4393.3413	0.0004
6	1	5	6	6	←	5	1	4	5	5	4413.3959	0.0012
6	1	5	6	7	←	5	1	4	5	6	4413.4512	-0.0013
6	1	5	7	8	←	5	1	4	6	7	4413.5535	-0.0071
7	1	7	7	7	←	6	1	6	6	6	4659.7004	0.0061
7	0	7	8	9	←	6	0	6	7	8	4659.8661	-0.0072
6	3	4	7	8	←	5	3	3	6	7	4704.5297	-0.0087
6	2	4	6	7	←	5	2	3	5	6	4861.3793	0.0069
7	2	6	7	7	←	6	2	5	6	6	5037.5856	-0.0060
7	2	6	7	8	←	6	2	5	6	7	5037.6522	-0.0012
7	2	6	8	9	←	6	2	5	7	8	5037.7272	0.0003
7	1	6	8	9	←	6	1	5	7	8	5043.2891	-0.0032
6	3	3	7	7	←	5	3	2	6	6	5130.0257	-0.0138
6	3	3	7	8	←	5	3	2	6	7	5130.1569	-0.0084
8	0	8	9	10	←	7	0	7	8	9	5298.3868	0.0008
7	3	5	7	7	←	6	3	4	6	6	5391.5220	0.0005
7	3	5	7	8	←	6	3	4	6	7	5391.6197	0.0051
7	3	5	8	9	←	6	3	4	7	8	5391.6993	0.0016
7	2	5	7	8	←	6	2	4	6	7	5469.7009	0.0060
7	2	5	8	9	←	6	2	4	7	8	5469.8211	-0.0092
7	4	4	8	9	←	6	4	3	7	8	5632.9685	-0.0078
7	6	2	6	6	←	6	6	1	5	5	5674.0380	0.0040
8	1	7	8	8	←	7	1	6	7	7	5678.8817	0.0124
8	1	7	9	10	←	7	1	6	8	9	5678.9696	-0.0084
7	5	3	6	7	←	6	5	2	5	6	5701.7947	0.0150
7	5	2	8	9	←	6	5	1	7	8	5789.7692	0.0067
9	0	9	9	10	←	8	0	8	8	9	5936.9417	0.0179
7	4	3	8	8	←	6	4	2	7	7	5982.7836	-0.0076
8	3	6	8	7	←	7	3	5	8	7	6050.7651	-0.0051
8	3	6	8	9	←	7	3	5	7	8	6050.8445	0.0071
8	3	6	9	8	←	7	3	5	8	7	6050.9247	0.0061
8	2	6	8	9	←	7	2	5	7	8	6078.9613	-0.0024
8	2	6	9	10	←	7	2	5	8	9	6079.0675	0.0033
9	2	8	10	9	←	8	2	7	9	8	6316.2859	-0.0131
9	1	8	8	9	←	8	1	7	7	8	6316.5942	-0.0126
8	4	5	8	8	←	7	4	4	7	7	6363.2863	-0.0095
8	3	5	8	9	←	7	3	4	7	8	6551.2409	-0.0173
10	1	10	11	10	←	9	1	9	10	9	6575.5185	-0.0008
10	1	10	9	10	←	9	1	9	8	9	6575.5185	-0.0027
10	0	10	11	10	←	9	0	9	10	9	6575.5183	-0.0017
10	0	10	9	10	←	9	0	9	8	9	6575.5183	-0.0036
9	3	7	8	9	←	8	3	6	7	8	6695.9628	-0.0014
9	2	7	8	9	←	8	2	6	7	8	6704.2915	-0.0078
8	4	4	9	10	←	7	4	3	8	9	6855.2759	0.0004
10	2	9	11	10	←	9	2	8	10	9	6954.7285	-0.0136

J'	K_a'	K_c'	I'	F'	←	J''	K_a''	K_c''	I''	F''	ν_{obs}	$\nu_{obs}-\nu_{calc}$
10	1	9	11	10	←	9	1	8	10	9	6954.8118	0.0051
9	4	6	10	10	←	8	4	5	9	9	7051.9803	-0.0007
9	4	6	10	9	←	8	4	5	9	8	7052.0488	-0.0068
9	3	6	9	10	←	8	3	5	8	9	7144.9931	0.0144
11	0	11	10	10	←	10	0	10	9	9	7214.0954	-0.0002
9	5	5	10	10	←	8	5	4	9	9	7296.9964	-0.0046
9	5	5	9	8	←	8	5	4	8	8	7297.1161	0.0193
10	3	8	9	10	←	9	3	7	8	9	7335.6905	-0.0043
10	2	8	9	10	←	9	2	7	8	9	7337.8884	0.0026
9	4	5	9	10	←	8	4	4	8	9	7608.1020	0.0111
10	4	7	9	8	←	9	4	6	9	8	7711.6277	0.0085
12	1	12	11	10	←	11	1	11	10	9	7852.6738	-0.0013
12	0	12	11	10	←	11	0	11	10	9	7852.6733	-0.0018
11	2	9	10	10	←	10	2	8	9	9	7974.4717	0.0093

Table S36. Measured rotational transitions (ν_{obs}) of the D (13,15) isotopic species of the $(Py)_2$-Bz trimer and residuals (ν_{obs} - ν_{calc}) (frequencies in MHz).

J′	*K*a′	*K*c′	*I′*	*F′*	←	*J″*	*K*a″	*K*c″	*I″*	*F″*	ν_{obs}	ν_{obs}-ν_{calc}
3	1	3	4	4	←	2	1	2	2	3	2097.0710	0.0151
3	1	3	4	5	←	2	1	2	3	4	2097.1872	-0.0106
3	2	2	3	2	←	2	2	1	2	2	2344.3639	-0.0012
3	2	2	4	4	←	2	2	1	3	3	2344.4275	0.0003
3	2	2	3	4	←	2	2	1	2	3	2344.9888	0.0038
3	2	2	4	3	←	2	2	1	3	2	2345.4220	-0.0083
3	1	2	4	5	←	2	1	1	3	4	2488.1274	0.0037
4	1	4	4	4	←	3	1	3	3	3	2750.9579	-0.0059
4	1	4	4	5	←	3	1	3	3	4	2751.0694	0.0196
4	1	4	5	6	←	3	1	3	4	5	2751.1463	0.0053
4	0	4	4	4	←	3	0	3	3	3	2760.7712	0.0055
4	0	4	5	6	←	3	0	3	4	5	2760.9177	-0.0157
4	2	3	4	4	←	3	2	2	3	3	3065.3855	-0.0020
4	2	3	5	5	←	3	2	2	4	4	3065.4674	-0.0141
4	2	3	4	5	←	3	2	2	3	4	3065.6513	0.0048
4	2	3	5	4	←	3	2	2	4	3	3065.8110	-0.0011
4	3	1	5	6	←	3	3	0	4	5	3353.2125	-0.0019
5	1	5	6	6	←	4	1	4	5	5	3396.4479	0.0011
5	0	5	5	5	←	4	0	4	4	4	3398.7013	0.0166
5	0	5	6	7	←	4	0	4	5	6	3398.7857	-0.0163
4	2	2	4	4	←	3	2	1	3	4	3422.2921	0.0064
5	2	4	5	5	←	4	2	3	4	4	3749.5179	-0.0005
5	2	4	4	4	←	4	2	3	4	4	3749.7586	0.0127
5	1	4	5	6	←	4	1	3	4	5	3804.3839	-0.0092
5	3	3	6	6	←	4	3	2	5	5	3993.1107	0.0049
5	3	3	6	7	←	4	3	2	5	6	3993.4027	-0.0089
5	3	3	5	5	←	4	3	2	5	4	3993.4975	-0.0086
6	1	6	6	6	←	5	1	5	5	5	4039.0974	0.0093
6	1	6	7	8	←	5	1	5	6	7	4039.1926	0.0188
6	0	6	6	6	←	5	0	5	5	5	4039.5695	-0.0042
6	0	6	7	8	←	5	0	5	6	7	4039.6657	0.0064
5	4	2	6	6	←	4	4	1	5	5	4066.2761	0.0077
5	4	2	6	7	←	4	4	1	5	6	4066.8303	0.0098
5	4	1	6	7	←	4	4	0	5	6	4126.5497	0.0101
5	2	3	6	7	←	4	2	2	5	6	4199.4257	0.0029
5	2	3	4	5	←	4	2	2	3	4	4199.5199	-0.0037
5	3	2	6	6	←	4	3	1	5	5	4280.9821	-0.0116
5	3	2	5	5	←	4	3	1	4	4	4281.0499	-0.0035
5	3	2	6	7	←	4	3	1	5	6	4281.2814	0.0056
6	2	5	6	7	←	5	2	4	5	6	4408.6431	-0.0058
6	2	5	7	6	←	5	2	4	6	5	4408.7388	0.0017
7	0	7	7	7	←	6	0	6	6	6	4681.2431	-0.0066
6	3	4	6	6	←	5	3	3	5	5	4720.9728	0.0060
6	3	4	7	7	←	5	3	3	6	6	4721.0554	-0.0034
6	3	4	6	7	←	5	3	3	5	6	4721.1263	-0.0102
6	3	4	7	8	←	5	3	3	6	7	4721.2112	-0.0048
6	2	4	6	7	←	5	2	3	5	6	4869.6794	0.0068
6	2	4	7	8	←	5	2	3	6	7	4869.8337	0.0092
6	4	3	6	6	←	5	4	2	5	5	4878.8388	0.0000
6	5	2	6	6	←	5	5	1	5	5	4889.6686	-0.0028
6	5	2	7	8	←	5	5	1	6	7	4890.1763	-0.0032
6	5	2	7	6	←	5	5	1	6	5	4890.2974	-0.0009
6	5	1	7	8	←	5	5	0	6	7	4912.3141	-0.0187
7	2	6	7	7	←	6	2	5	6	6	5055.6954	-0.0073
7	2	6	7	8	←	6	2	5	6	7	5055.7681	0.0033
7	2	6	6	7	←	6	2	5	5	6	5055.8379	-0.0040
7	1	6	8	9	←	6	1	5	7	8	5060.6363	-0.0034
6	4	2	7	7	←	5	4	1	5	6	5067.3046	0.0041
6	4	2	5	6	←	5	4	1	4	5	5067.5608	0.0082
6	4	2	6	7	←	5	4	1	5	6	5067.6654	-0.0089
6	3	3	7	7	←	5	3	2	6	6	5149.4523	-0.0074
6	3	3	7	8	←	5	3	2	6	7	5149.5872	0.0061
8	1	8	9	10	←	7	1	7	8	9	5323.1511	0.0104
8	0	8	9	10	←	7	0	7	8	9	5323.1511	-0.0072

J′	*K*a′	*K*c′	*I*′	*F*′	←	*J*″	*K*a″	*K*c″	*I*″	*F*″	ν_{obs}	ν_{obs}-ν_{calc}
7	3	5	7	7	←	6	3	4	6	6	5408.7889	-0.0004
7	3	5	7	8	←	6	3	4	6	7	5408.8837	0.0021
7	3	5	8	9	←	6	3	4	7	8	5408.9681	0.0024
7	2	5	7	8	←	6	2	4	6	7	5479.7299	-0.0073
7	2	5	8	9	←	6	2	4	7	8	5479.8736	0.0026
7	4	4	8	8	←	6	4	3	7	7	5654.8217	-0.0055
7	4	4	6	7	←	6	4	3	5	6	5655.0060	-0.0022
8	2	7	7	8	←	7	2	6	6	7	5698.8397	-0.0107
8	1	7	9	10	←	7	1	6	8	9	5699.9719	-0.0080
7	6	2	8	8	←	6	6	1	7	7	5703.1806	0.0029
7	6	1	8	8	←	6	6	0	7	7	5710.3835	-0.0009
7	6	1	8	7	←	6	6	0	7	6	5710.9531	0.0068
7	5	3	7	7	←	6	5	2	6	6	5729.2266	0.0036
7	5	3	8	9	←	6	5	2	7	8	5729.5267	-0.0128
7	5	3	8	7	←	6	5	2	7	6	5729.5963	-0.0008
7	5	2	8	8	←	6	5	1	7	7	5824.7098	-0.0182
7	5	2	7	7	←	6	5	1	6	6	5824.8142	0.0075
7	5	2	8	9	←	6	5	1	7	8	5825.0879	-0.0058
7	5	2	7	8	←	6	5	1	6	7	5825.1758	0.0084
7	5	2	7	6	←	6	5	1	6	5	5825.2529	0.0143
7	3	4	7	8	←	6	3	3	6	7	5911.5368	-0.0031
7	3	4	8	9	←	6	3	3	7	8	5911.6350	0.0001
7	4	3	8	8	←	6	4	2	7	7	6013.6892	0.0089
7	4	3	8	9	←	6	4	2	7	8	6013.8648	-0.0011
8	3	6	8	8	←	7	3	5	7	7	6069.4977	-0.0006
8	3	6	8	9	←	7	3	5	7	8	6069.5644	0.0009
8	3	6	9	10	←	7	3	5	8	9	6069.6487	0.0057
8	2	6	8	9	←	7	2	5	7	8	6094.0798	-0.0080
8	2	6	9	10	←	7	2	5	8	9	6094.1809	-0.0054
9	1	8	10	9	←	8	1	7	9	8	6341.0315	-0.0109
9	2	8	10	9	←	8	2	7	9	8	6340.7867	-0.0096
8	4	5	8	8	←	7	4	4	7	7	6384.6373	0.0106
8	7	2	8	8	←	7	7	1	7	7	6512.9523	-0.0093
8	7	2	8	9	←	7	7	1	7	8	6513.3881	0.0030
8	7	1	8	8	←	7	7	0	7	7	6515.1164	-0.0051
8	7	1	8	9	←	7	7	0	7	8	6515.5398	-0.0064
8	5	4	9	10	←	7	5	3	8	9	6547.4052	-0.0089
8	6	3	9	9	←	7	6	2	8	8	6555.6581	-0.0021
8	3	5	8	9	←	7	3	4	7	8	6559.6978	-0.0023
8	3	5	9	10	←	7	3	4	8	9	6559.8358	0.0051
8	6	2	8	8	←	7	6	1	7	7	6594.9645	-0.0143
10	1	10	11	10	←	9	1	9	10	9	6606.9460	0.0082
10	1	10	9	10	←	9	1	9	8	9	6606.9460	0.0064
10	0	10	11	10	←	9	0	9	10	9	6606.9460	0.0077
10	0	10	9	10	←	9	0	9	8	9	6606.9460	0.0058
9	3	7	8	9	←	8	3	6	7	8	6717.1206	-0.0042
9	2	7	8	9	←	8	2	6	7	8	6724.1286	-0.0104
8	5	3	9	9	←	7	5	2	8	8	6795.4735	-0.0089
8	5	3	8	8	←	7	5	2	7	7	6795.5645	0.0096
8	5	3	9	10	←	7	5	2	8	9	6795.7313	0.0162
8	4	4	9	8	←	7	4	3	8	7	6880.0049	0.0104
10	1	9	10	9	←	9	1	8	9	8	6982.5749	0.0092
9	4	6	9	10	←	8	4	5	8	9	7072.9309	-0.0054
9	4	6	10	9	←	8	4	5	9	8	7073.0205	0.0093
9	3	6	9	10	←	8	3	5	8	9	7155.5859	-0.0026
9	3	6	10	10	←	8	3	5	9	9	7155.6997	0.0106
11	0	11	10	10	←	10	0	10	9	9	7248.8426	0.0075
9	5	5	10	10	←	8	5	4	9	9	7324.6902	0.0111
9	5	5	8	9	←	8	5	4	7	8	7324.7994	-0.0077
10	3	8	9	10	←	9	3	7	8	9	7359.8720	-0.0077
10	2	8	9	10	←	9	2	7	8	9	7361.6561	-0.0069
9	7	3	10	10	←	8	7	2	9	9	7369.3586	-0.0122
9	7	3	9	10	←	8	7	2	8	9	7369.6864	0.0112
9	7	2	10	10	←	8	7	1	9	9	7383.4518	0.0082
9	7	2	9	10	←	8	7	1	8	9	7383.7575	-0.0050
9	6	4	8	9	←	8	6	3	7	8	7402.8178	-0.0068
9	4	5	9	10	←	8	4	4	8	9	7622.8424	-0.0035
10	4	7	10	10	←	9	4	6	10	10	7733.3764	0.0167

J'	K_a'	K_c'	I'	F'	←	J''	K_a''	K_c''	I''	F''	ν_{obs}	$\nu_{obs}-\nu_{calc}$
10	4	7	9	10	←	9	4	6	8	9	7733.4790	-0.0053
9	5	4	10	10	←	8	5	3	9	9	7753.1560	0.0056
9	5	4	8	9	←	8	5	3	7	8	7753.2869	0.0041
10	3	7	11	10	←	9	3	6	10	9	7762.9819	0.0135
12	1	12	11	10	←	11	1	11	10	9	7890.7398	0.0042

Table S37. Measured rotational transitions (ν_{obs}) of the D (14,16) isotopic species of the $(Py)_2$-Bz trimer and residuals (ν_{obs} - ν_{calc}) (frequencies in MHz).

J′	*K*a′	*K*c′	*I*′	*F*′	←	*J*″	*K*a″	*K*c″	*I*″	*F*″	ν_{obs}	ν_{obs}-ν_{calc}
3	1	3	4	4	←	2	1	2	3	3	2108.3258	0.0024
3	1	3	4	5	←	2	1	2	3	4	2108.4450	-0.0133
3	0	3	4	5	←	2	0	2	3	4	2138.6963	0.0141
3	1	2	4	4	←	2	1	1	3	3	2504.1962	0.0064
3	1	2	4	5	←	2	1	1	3	4	2504.3271	-0.0029
4	1	4	4	4	←	3	1	3	3	3	2764.5062	-0.0057
4	1	4	5	5	←	3	1	3	3	4	2764.6121	-0.0091
4	1	4	5	6	←	3	1	3	4	5	2764.6899	0.0008
4	0	4	3	3	←	3	0	3	2	2	2773.0475	0.0073
4	2	3	4	4	←	3	2	2	3	3	3084.6392	0.0047
4	2	3	5	5	←	3	2	2	4	4	3084.7116	-0.0186
4	2	3	4	5	←	3	2	2	3	4	3084.8941	0.0025
4	2	3	5	4	←	3	2	2	4	3	3085.0552	-0.0035
4	3	1	5	5	←	3	3	0	4	4	3390.4121	-0.0061
4	3	1	4	4	←	3	3	0	3	3	3390.5656	0.0024
4	3	1	4	5	←	3	3	0	3	4	3391.2127	-0.0010
5	1	5	5	5	←	4	1	4	4	4	3412.6180	0.0164
5	1	5	6	7	←	4	1	4	5	6	3412.7059	-0.0142
5	0	5	5	5	←	4	0	4	4	4	3414.5233	0.0184
4	2	2	5	5	←	3	2	1	4	4	3450.8203	0.0003
4	2	2	5	6	←	3	2	1	4	5	3451.0107	-0.0146
5	2	4	5	5	←	4	2	3	4	4	3769.6746	0.0045
5	2	4	5	6	←	4	2	3	4	5	3769.7885	-0.0148
5	2	4	6	7	←	4	2	3	5	6	3769.9077	0.0034
5	1	4	5	6	←	4	1	3	4	5	3818.4479	-0.0148
5	1	4	6	6	←	4	1	3	5	5	3818.5777	0.0118
5	3	3	6	6	←	4	3	2	5	5	4022.6507	0.0121
5	3	3	6	7	←	4	3	2	5	6	4022.9424	-0.0004
6	1	6	6	6	←	5	1	5	5	5	4058.2756	-0.0027
6	1	6	7	8	←	5	1	5	6	7	4058.3730	0.0090
6	0	6	6	6	←	5	0	5	5	5	4058.6545	-0.0014
6	0	6	7	8	←	5	0	5	6	7	4058.7520	0.0106
5	4	2	6	6	←	4	4	1	5	5	4103.6683	0.0011
5	4	2	6	7	←	4	4	1	5	6	4104.2106	-0.0097
5	4	1	5	5	←	4	4	0	4	4	4171.8268	-0.0198
5	4	1	6	7	←	4	4	0	5	6	4172.3786	0.0037
5	2	3	5	6	←	4	2	2	4	5	4223.9751	-0.0043
5	2	3	6	6	←	4	2	2	5	5	4224.0443	0.0083
5	2	3	6	7	←	4	2	2	5	6	4224.1107	0.0159
5	3	2	6	6	←	4	3	1	5	5	4325.4362	-0.0110
5	3	2	5	5	←	4	3	1	4	4	4325.5129	0.0100
5	3	2	6	7	←	4	3	1	5	6	4325.7224	-0.0029
6	2	5	6	6	←	5	2	4	5	5	4430.0630	0.0055
6	2	5	6	7	←	5	2	4	5	6	4430.1417	-0.0004
6	2	5	7	8	←	5	2	4	6	7	4430.2301	-0.0001
6	1	5	6	6	←	5	1	4	5	5	4445.0957	-0.0064
6	1	5	6	7	←	5	1	4	5	6	4445.1773	0.0132
6	1	5	7	8	←	5	1	4	6	7	4445.2653	-0.0026
7	1	7	7	7	←	6	1	6	6	6	4703.4072	0.0023
7	1	7	8	9	←	6	1	6	7	8	4703.4889	0.0190
6	3	4	6	6	←	5	3	3	5	5	4750.4199	0.0084
6	3	4	7	7	←	5	3	3	6	6	4750.5075	0.0015
6	3	4	6	7	←	5	3	3	5	6	4750.5827	0.0043
6	3	4	7	8	←	5	3	3	6	7	4750.6663	0.0056
6	2	4	6	7	←	5	2	3	5	6	4887.4206	0.0048
6	2	4	7	8	←	5	2	3	6	7	4887.5620	-0.0089
6	4	3	7	7	←	5	4	2	6	6	4920.1796	0.0066
6	4	3	7	8	←	5	4	2	6	7	4920.4977	0.0020
6	5	2	7	6	←	5	5	1	6	5	4937.2552	-0.0148
6	5	1	7	7	←	5	5	0	6	6	4963.3115	0.0094
6	5	1	5	6	←	5	5	0	4	5	4963.8290	0.0005
6	5	1	5	4	←	5	5	0	4	3	4963.9641	0.0080
7	2	6	7	7	←	6	2	5	6	6	5079.4274	-0.0120
7	2	6	7	8	←	6	2	5	6	7	5079.5110	0.0093

J'	K_a'	K_c'	I'	F'	←	J''	K_a''	K_c''	I''	F''	ν_{obs}	ν_{obs}- ν_{calc}
7	2	6	8	9	←	6	2	5	7	8	5079.5727	-0.0019
7	1	6	8	9	←	6	1	5	7	8	5083.3390	-0.0052
6	4	2	7	8	←	5	4	1	6	7	5127.9064	0.0071
6	3	3	7	7	←	5	3	2	6	6	5191.0550	-0.0170
6	3	3	7	8	←	5	3	2	6	7	5191.1879	0.0025
8	1	8	9	10	←	7	1	7	8	9	5348.4680	-0.0104
7	3	5	7	7	←	6	3	4	6	6	5437.5606	0.0042
7	3	5	7	8	←	6	3	4	6	7	5437.6496	0.0024
7	3	5	8	9	←	6	3	4	7	8	5437.7391	0.0061
7	2	5	7	8	←	6	2	4	6	7	5498.2544	0.0051
8	2	7	9	10	←	7	2	6	8	9	5725.3830	-0.0128
8	1	7	9	8	←	7	1	6	8	7	5726.2227	-0.0084
7	6	2	8	9	←	6	6	1	7	8	5759.3068	0.0100
7	6	2	7	6	←	6	6	1	6	5	5759.3879	-0.0030
7	6	1	7	8	←	6	6	0	6	7	5768.4457	0.0029
7	5	3	8	9	←	6	5	2	7	8	5782.8206	-0.0010
7	5	3	8	7	←	6	5	2	7	6	5782.8782	-0.0005
7	5	2	8	8	←	6	5	1	7	7	5894.1201	0.0017
7	5	2	8	9	←	6	5	1	7	8	5894.4862	-0.0036
7	3	4	7	8	←	6	3	3	6	7	5942.3000	-0.0066
7	3	4	8	9	←	6	3	3	7	8	5942.4202	0.0092
9	1	9	9	10	←	8	1	8	8	9	5993.4676	0.0193
9	0	9	9	10	←	8	0	8	8	9	5993.4675	0.0170
7	4	3	8	8	←	6	4	2	7	7	6076.5265	0.0099
7	4	3	6	7	←	6	4	2	5	6	6076.6979	0.0033
8	3	6	8	8	←	7	3	5	7	7	6098.7003	-0.0077
8	3	6	7	8	←	7	3	5	6	7	6098.8567	-0.0044
8	2	6	9	10	←	7	2	5	8	9	6118.4378	-0.0085
8	7	2	8	9	←	7	7	1	7	8	6577.1151	0.0047
8	3	5	8	9	←	7	3	4	7	8	6580.1130	0.0047
8	5	4	9	9	←	7	5	3	8	8	6602.4721	-0.0066
8	5	4	9	10	←	7	5	3	8	9	6602.6645	0.0041
8	6	3	9	9	←	7	6	2	8	8	6619.8470	0.0028
10	1	10	11	10	←	9	1	9	10	9	6638.4621	-0.0014
10	1	10	9	10	←	9	1	9	8	9	6638.4621	-0.0032
10	0	10	11	10	←	9	0	9	10	9	6638.4619	-0.0019
10	0	10	9	10	←	9	0	9	8	9	6638.4619	-0.0037
8	6	2	8	8	←	7	6	1	7	7	6668.6204	0.0130
9	3	7	8	9	←	8	3	6	7	8	6748.1796	-0.0099
9	2	7	8	9	←	8	2	6	7	8	6753.4401	-0.0143
8	5	3	9	9	←	7	5	2	8	8	6879.2050	0.0103
8	5	3	8	8	←	7	5	2	7	7	6879.2827	0.0167
8	5	3	9	10	←	7	5	2	8	9	6879.4314	0.0054
8	4	4	9	10	←	7	4	3	8	9	6932.7229	0.0047
10	2	9	11	10	←	9	2	8	10	9	7015.2255	-0.0021
9	4	6	9	9	←	8	4	5	9	9	7109.9219	0.0049
9	4	6	8	9	←	8	4	5	7	8	7109.9982	0.0018
9	3	6	9	10	←	8	3	5	8	9	7177.9056	-0.0034
9	3	6	10	10	←	8	3	5	9	9	7178.0190	0.0166
11	1	11	10	10	←	10	1	10	9	9	7283.4552	0.0003
11	0	11	10	10	←	10	0	10	9	9	7283.4550	0.0001
9	5	5	10	10	←	8	5	4	9	9	7377.5681	-0.0020
9	5	5	10	9	←	8	5	4	9	8	7377.6939	-0.0062
10	3	8	9	10	←	9	3	7	8	9	7393.6306	-0.0106
10	2	8	11	10	←	9	2	7	10	9	7394.8925	-0.0074
9	7	3	10	10	←	8	7	2	9	9	7443.0428	0.0074
9	7	3	8	7	←	8	7	2	7	6	7443.3736	-0.0101
9	6	4	9	9	←	8	6	3	8	8	7471.7374	-0.0113
9	6	4	8	9	←	8	6	3	7	8	7471.9491	-0.0136
9	6	3	9	10	←	8	6	2	8	9	7633.6271	-0.0194
9	4	5	9	10	←	8	4	4	8	9	7657.2626	-0.0013
9	4	5	10	10	←	8	4	4	9	9	7657.3691	0.0111
10	4	7	9	10	←	9	4	6	8	9	7770.1036	-0.0044
10	3	7	9	10	←	9	3	6	8	9	7792.6757	-0.0016
9	5	4	8	8	←	8	5	3	7	7	7833.3192	0.0030
9	5	4	9	10	←	8	5	3	8	9	7833.4421	-0.0132
12	1	12	11	10	←	11	1	11	10	9	7928.4453	-0.0040
12	0	12	11	10	←	11	0	11	10	9	7928.4451	-0.0042

Table S38. Measured rotational transitions (ν_{obs}) of the D (24) isotopic species of the $(Py)_2$-Bz trimer and residuals (ν_{obs} - ν_{calc}) (frequencies in MHz).

J'	*K*a'	*K*c'	*I'*	*F'*	←	*J''*	*K*a''	*K*c''	*I''*	*F''*	ν_{obs}	ν_{obs}-ν_{calc}
5	1	5	6	7	←	4	1	4	5	6	3415.5490	-0.0092
5	0	5	5	5	←	4	0	4	4	4	3417.1980	0.0037
6	1	6	6	6	←	5	1	5	5	5	4061.1263	0.0006
6	1	6	7	8	←	5	1	5	6	7	4061.2232	0.0119
6	0	6	6	6	←	5	0	5	5	5	4061.4633	-0.0014
6	0	6	7	8	←	5	0	5	6	7	4061.5649	0.0147
7	1	7	7	7	←	6	1	6	6	6	4706.2975	-0.0009
7	1	7	8	9	←	6	1	6	7	8	4706.3693	0.0059
7	0	7	7	7	←	6	0	6	6	6	4706.3693	0.0097
8	1	8	9	10	←	7	1	7	8	9	5351.4194	-0.0090
8	0	8	9	10	←	7	0	7	8	9	5351.4194	-0.0195
9	1	9	9	10	←	8	1	8	8	9	5996.4771	0.0200
9	0	9	9	10	←	8	0	8	8	9	5996.4771	0.0183
10	1	10	11	10	←	9	1	9	10	9	6641.5349	0.0038
10	1	10	9	10	←	9	1	9	8	9	6641.5349	0.0019
10	0	10	11	10	←	9	0	9	10	9	6641.5349	0.0035
10	0	10	9	10	←	9	0	9	8	9	6641.5349	0.0017
11	1	11	10	10	←	10	1	10	9	9	7286.5860	0.0048
11	0	11	10	10	←	10	0	10	9	9	7286.5860	0.0047
12	1	12	11	10	←	11	1	11	10	9	7931.6370	0.0025
12	0	12	11	10	←	11	0	11	10	9	7931.6370	0.0025
6	2	4	6	7	←	5	2	3	5	6	4899.0282	0.0021
7	2	5	7	8	←	6	2	4	6	7	5508.8475	0.0079
10	2	8	9	10	←	9	2	7	8	9	7407.6791	0.0008
10	3	8	9	10	←	9	3	7	8	9	7406.5891	-0.0045
9	3	7	8	9	←	8	3	6	7	8	6761.2294	0.0011
8	3	6	8	8	←	7	3	5	7	7	6112.1400	-0.0084
8	3	6	8	9	←	7	3	5	7	8	6112.2139	0.0006
8	3	6	9	10	←	7	3	5	8	9	6112.3004	0.0070
3	0	3	3	4	←	2	0	2	2	3	2140.8827	0.0224
3	2	2	3	4	←	2	2	1	3	4	2371.2569	0.0059
3	1	2	2	2	←	2	1	1	1	2	2514.1407	0.0074
3	1	2	4	5	←	2	1	1	3	4	2514.3031	0.0137
4	1	4	4	4	←	3	1	3	3	3	2767.4309	-0.0055
4	1	4	5	6	←	3	1	3	4	5	2767.6037	-0.0098
4	0	4	3	3	←	3	0	3	2	2	2775.5002	-0.0123
4	2	3	5	5	←	3	2	2	4	5	3094.0079	-0.0167
4	1	3	4	5	←	3	1	2	3	4	3200.5610	0.0232
4	1	3	5	5	←	3	1	2	4	4	3200.7000	0.0104
4	2	2	5	6	←	3	2	1	4	5	3468.8247	-0.0143
5	2	4	5	5	←	4	2	3	4	4	3778.4447	-0.0025
5	2	4	6	6	←	4	2	3	5	5	3778.5592	0.0118
5	2	4	6	7	←	4	2	3	5	6	3778.6897	0.0081
5	1	4	5	6	←	4	1	3	4	5	3825.0723	-0.0152
5	1	4	6	6	←	4	1	3	5	5	3825.1869	-0.0010
5	1	4	4	5	←	4	1	3	3	4	3825.2705	0.0051
5	2	3	5	6	←	4	2	2	4	5	4239.8647	-0.0010
5	2	3	6	7	←	4	2	2	5	6	4239.9835	-0.0014
5	3	2	6	7	←	4	3	1	5	6	4352.2434	-0.0069
6	2	5	6	7	←	5	2	4	5	6	4438.3669	0.0008
6	2	5	7	8	←	5	2	4	6	7	4438.4518	-0.0025
6	1	5	6	6	←	5	1	4	5	5	4452.2240	-0.0089
6	1	5	7	8	←	5	1	4	6	7	4452.3957	-0.0030
6	4	3	5	5	←	5	4	2	4	4	4943.3670	-0.0021
7	2	6	7	7	←	6	2	5	6	6	5087.4076	-0.0020
7	2	6	7	8	←	6	2	5	6	7	5087.4716	-0.0004
7	2	6	8	9	←	6	2	5	7	8	5087.5455	0.0007
7	1	6	7	7	←	6	1	5	6	6	5090.8102	-0.0002
7	1	6	8	9	←	6	1	5	7	8	5090.9433	-0.0012
6	3	3	5	6	←	5	3	2	4	5	5217.2194	-0.0198
7	3	5	7	7	←	6	3	4	6	6	5451.9200	0.0039
7	3	5	7	8	←	6	3	4	6	7	5452.0094	0.0031
7	3	5	8	9	←	6	3	4	7	8	5452.1026	0.0098
7	2	5	8	9	←	6	2	4	7	8	5508.9634	-0.0050

J′	*K*a′	*K*c′	*I′*	*F′*	←	*J″*	*K*a″	*K*c″	*I″*	*F″*	ν_{obs}	$\nu_{obs}-\nu_{calc}$
7	4	4	8	8	←	6	4	3	7	7	5719.5361	0.0035
8	2	7	9	10	←	7	2	6	8	9	5733.3090	-0.0115
8	1	7	9	10	←	7	1	6	8	9	5734.0491	-0.0079
7	3	4	7	6	←	6	3	3	6	5	5962.8364	-0.0064
7	3	4	6	6	←	6	3	3	5	5	5962.9651	0.0065
7	4	3	8	8	←	6	4	2	7	7	6113.8718	0.0182
7	4	3	8	9	←	6	4	2	7	8	6114.0244	-0.0039
8	2	6	9	10	←	7	2	5	8	9	6130.0852	-0.0058
9	2	8	10	9	←	8	2	7	9	8	6378.3224	-0.0116
9	1	8	8	9	←	8	1	7	7	8	6378.4728	-0.0121
8	3	5	8	7	←	7	3	4	7	6	6594.8365	0.0055
8	3	5	9	10	←	7	3	4	8	9	6594.9645	-0.0044
9	2	7	8	9	←	8	2	6	7	8	6765.8645	-0.0106
8	4	4	9	10	←	7	4	3	8	9	6965.8226	-0.0083
10	2	9	11	10	←	9	2	8	10	9	7023.2483	0.0021
9	4	6	8	9	←	8	4	5	7	8	7129.7826	0.0043
9	3	6	8	7	←	8	3	5	8	7	7192.2450	0.0107
9	4	5	9	10	←	8	4	4	8	9	7681.1555	-0.0075
9	4	5	10	10	←	8	4	4	9	9	7681.2748	0.0097
10	4	7	9	10	←	9	4	6	8	9	7788.6422	-0.0060
10	3	7	9	10	←	9	3	6	8	9	7808.7320	0.0000
9	5	4	9	10	←	8	5	3	8	9	7880.8867	0.0074

2.2 Experimental rotational parameters

Table S39. Experimental rotational parameters of the $^{12}C/^{14}N/^{1}H$ (normal) isotopic species of the $(Py)_2$-Bz trimer.

	$^{12}C/^{14}N/^{1}H$
A / MHz	575.75643(16)
B / MHz	467.37093(12)
C / MHz	323.31877(11)
Δ_J / kHz	0.05231(64)
Δ_{JK} / kHz[a]	[0]
Δ_K/ kHz	0.0400(45)
δ_J / kHz	0.01615(46)
δ_K / kHz	-0.0102(24)
χ_{aa} (1) / MHz	-2.0039(33)
χ_{bb} (1) / MHz	0.6676(51)
χ_{cc} (1) / MHz	1.3363(51)
χ_{aa} (2) / MHz	-0.3643(41)
χ_{bb} (2) / MHz	-0.9591(51)
χ_{cc} (2) / MHz	1.3233(51)
N_{lines}	395
σ_{fit} / kHz	7.2

[a] Could not be determined from the fit, value was set to 0.

Table S40. Experimental rotational parameters of the mono ^{13}C substituted isotopic species of the $(Py)_2$-Bz trimer.

	^{13}C (1,3)	^{13}C (4,5)	^{13}C (6,10)	^{13}C (7,9)	^{13}C (8)	^{13}C (11)
A / MHz	571.6586(22)	569.2285(17)	573.5620(13)	574.4079(20)	575.5618(20)	573.9014(28)
B / MHz	466.85500(95)	466.89201(63)	465.68316(50)	463.29610(45)	462.31116(40)	467.1214(10)
C / MHz	322.29022(17)	321.22358(18)	322.40419(21)	321.54743(23)	320.82592(18)	322.53825(22)
Δ_J / kHz	[0.05231]	[0.05231]	[0.05231]	[0.05231]	[0.05231]	[0.05231]
Δ_{JK} / kHz[a]	[0]	[0]	[0]	[0]	[0]	[0]
Δ_K/ kHz	[0.0400]	[0.0400]	[0.0400]	[0.0400]	[0.0400]	[0.0400]
δ_J / kHz	[0.01615]	[0.01615]	[0.01615]	[0.01615]	[0.01615]	[0.01615]
δ_K / kHz	[-0.0102]	[-0.0102]	[-0.0102]	[-0.0102]	[-0.0102]	[-0.0102]
χ_{aa} (1) / MHz	[-2.0039]	[-2.0039]	[-2.0039]	[-2.0039]	[-2.0039]	[-2.0039]
χ_{bb} (1) / MHz	[0.6676]	[0.6676]	[0.6676]	[0.6676]	[0.6676]	[0.6676]
χ_{cc} (1) / MHz	[1.3363]	[1.3363]	[1.3363]	[1.3363]	[1.3363]	[1.3363]
χ_{aa} (2) / MHz	[-0.3643]	[-0.3643]	[-0.3643]	[-0.3643]	[-0.3643]	[-0.3643]
χ_{bb} (2) / MHz	[-0.9591]	[-0.9591]	[-0.9591]	[-0.9591]	[-0.9591]	[-0.9591]
χ_{cc} (2) / MHz	[1.3233]	[1.3233]	[1.3233]	[1.3233]	[1.3233]	[1.3233]
N_{lines}	36	31	22	21	31	11
σ_{fit} / kHz	8.6	8.8	9.2	9.2	10.0	8.0

	^{13}C (23,27)	^{13}C (25,26)
A / MHz	574.3932(27)	572.6179(41)
B / MHz	464.6035(10)	464.0177(15)
C / MHz	322.07527(16)	320.91877(23)
Δ_J / kHz	[0.05231]	[0.05231]
Δ_{JK} / kHz[a]	[0]	[0]
Δ_K/ kHz	[0.0400]	[0.0400]
δ_J / kHz	[0.01615]	[0.01615]
δ_K / kHz	[-0.0102]	[-0.0102]
χ_{aa} (1) / MHz	[-2.0039]	[-2.0039]
χ_{bb} (1) / MHz	[0.6676]	[0.6676]
χ_{cc} (1) / MHz	[1.3363]	[1.3363]
χ_{aa} (2) / MHz	[-0.3643]	[-0.3643]
χ_{bb} (2) / MHz	[-0.9591]	[-0.9591]
χ_{cc} (2) / MHz	[1.3233]	[1.3233]
N_{lines}	32	26
σ_{fit} / kHz	8.7	9.7

Parameters in brackets were kept at the normal species values.

Table S41. Experimental rotational parameters of the mono D substituted isotopic species of the $(Py)_2$-Bz trimer.

	D (12)	D (13,15)	D (14,16)	D (24)
A / MHz	575.76578(95)	572.66518(68)	571.09432(67)	572.62828(86)
B / MHz	459.02864(22)	460.66800(14)	464.87669(14)	467.81379(26)
C / MHz	319.31291(13)	320.97301(14)	322.51948(12)	322.54866(15)
Δ_J / kHz	[0.05231]	[0.05231]	[0.05231]	[0.05231]
Δ_{JK} / kHz[a]	[0]	[0]	[0]	[0]
Δ_K/ kHz	[0.0400]	[0.0400]	[0.0400]	[0.0400]
δ_J / kHz	[0.01615]	[0.01615]	[0.01615]	[0.01615]
δ_K / kHz	[-0.0102]	[-0.0102]	[-0.0102]	[-0.0102]
χ_{aa} (1) / MHz	[-2.0039]	[-2.0039]	[-2.0039]	[-2.0039]
χ_{bb} (1) / MHz	[0.6676]	[0.6676]	[0.6676]	[0.6676]
χ_{cc} (1) / MHz	[1.3363]	[1.3363]	[1.3363]	[1.3363]
χ_{aa} (2) / MHz	[-0.3643]	[-0.3643]	[-0.3643]	[-0.3643]
χ_{bb} (2) / MHz	[-0.9591]	[-0.9591]	[-0.9591]	[-0.9591]
χ_{cc} (2) / MHz	[1.3233]	[1.3233]	[1.3233]	[1.3233]
N_{lines}	76	133	130	78
σ_{fit} / kHz	8.4	8.3	9.0	9.0

Parameters in brackets were kept at the normal species values.

2.3 Least Squares Structural Analysis for the $(Py)_2$-Bz cluster

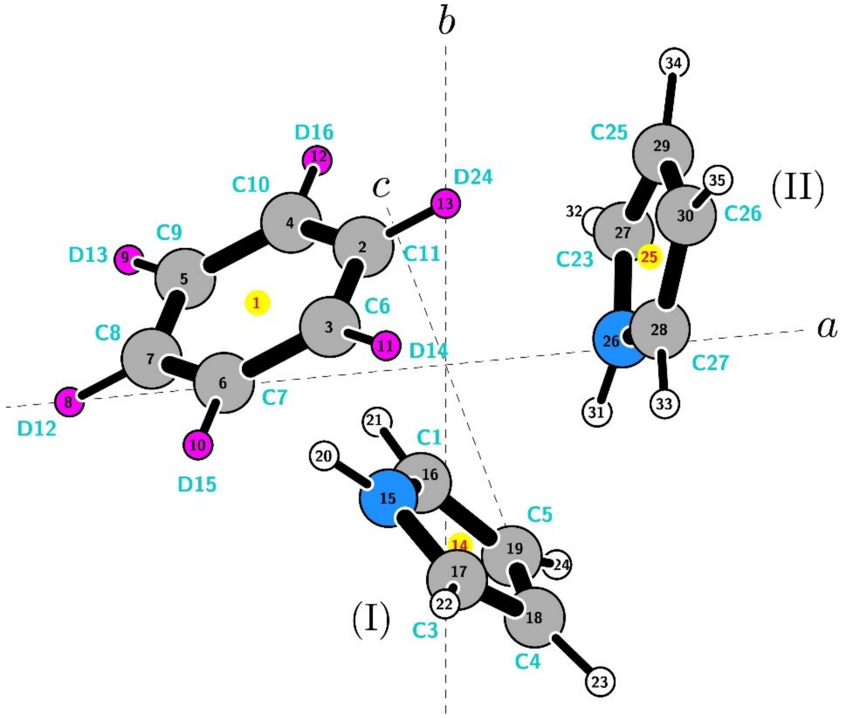

Figure S3. Atom numbering used in the least-squares fit of the $(Py)_2$-Bz structure. Dummy atoms 1, 14, and 25 mark centers of mass of the three monomers. The orientation of the principal axes is indicated.
Singly substituted ^{13}C isotopic species for all carbon atoms have been observed, as well as single D substituted species for hydrogen atoms indicated with magenta. Numbering used in the spectroscopic assignment in tables above is marked in turquoise.

This cluster contains 14 carbon atoms, and ^{13}C singly isotopic species have been observed for all. Since the cluster is of C_s symmetry six pairs of these carbons contain symmetry equivalent atoms, and there are two such pairs per each substituent molecule. There are, therefore, 8 distinct ^{13}C species.

This data is complemented by singly substituted species with hydrogen replaced by deuterium, all in the benzene unit, in which equivalence of two pairs of hydrogens leads to 4 distinct D species.

Together with the parent isotopic species this leads to 1+8+4=13 distinct isotopic species available for structural analysis. In fact, the symmetry identical species have also been declared, in order to enforce C_s symmetry, leading to 13+6+2=21 isotopic species, corresponding to 63 experimental rotational constants used in the fit.

The arguments concerning the choices made in performing the analysis were the same as detailed above for the Py-$(Bz)_2$ cluster. Intercomparison, reported in Table S42, between the results of the two alternative least squares fits indicates a clearer preference for the fits based on experimental r_e monomer structures, as apparent from significantly smaller deviations of fit. The key structural parameters for this fit are given in Figure S4 and Figure 1.

The abbreviated results file for the preferred least squares structural fit is included in Table S43.

Table S42. Comparison of the results of least squares structural fits for the $(Py)_2$-Bz cluster with computed values.

(Pyrr)2-Benz	exptal re monomers(a,b)	ORCA monomers	ORCA calc(c)
R(14, 1) =	4.2627(72)	4.2604(89)	4.2269
R(25, 1) =	4.6297(61)	4.6150(77)	4.6123
R(25,14)	4.2718(73)	4.2730(95)	4.2581
A(14, 1, 2) =	82.077(298)	82.408(376)	81.400
A(15,14, 1) =	15.134(589)	19.390(418)	19.591
A(25, 1,14) =	57.054(136)	57.393(172)	57.399
A(26,25, 1) =	71.504(670)	72.038(890)	73.614
c_c =	-0.0014(7)	0.0404(10)	
A(1,25,14) =	57.054(136)	57.128(174)	56.747
A(26,25,14) =	14.450(661)	14.909(877)	16.867
A(25,14,15) =	80.839(592)	84.869(421)	85.445
Chi-squared =	0.3472	0.6283	
DevFit /uA^2 =	0.078743	0.105924	
DevFit /MHz =	0.035697	0.056153	
NDEGf =	56(d)	56	

a - pyrrole geometry: A.G.Csaszar,J.Demaison, H.D.Rudolph, J.Phys.Chem.A 2015, 119,1731-1746

b - benzene geometry: J.Gauss, J.F.Stanton, J.Phys.Chem.A 2000, 104, 2865-2868.

c - refined using the DLPNO-CCSDS(T)/aug-cc-pVnZ approach

d - number of degrees of freedom (fit of 7 parameters to 63 experimental rotational constants)

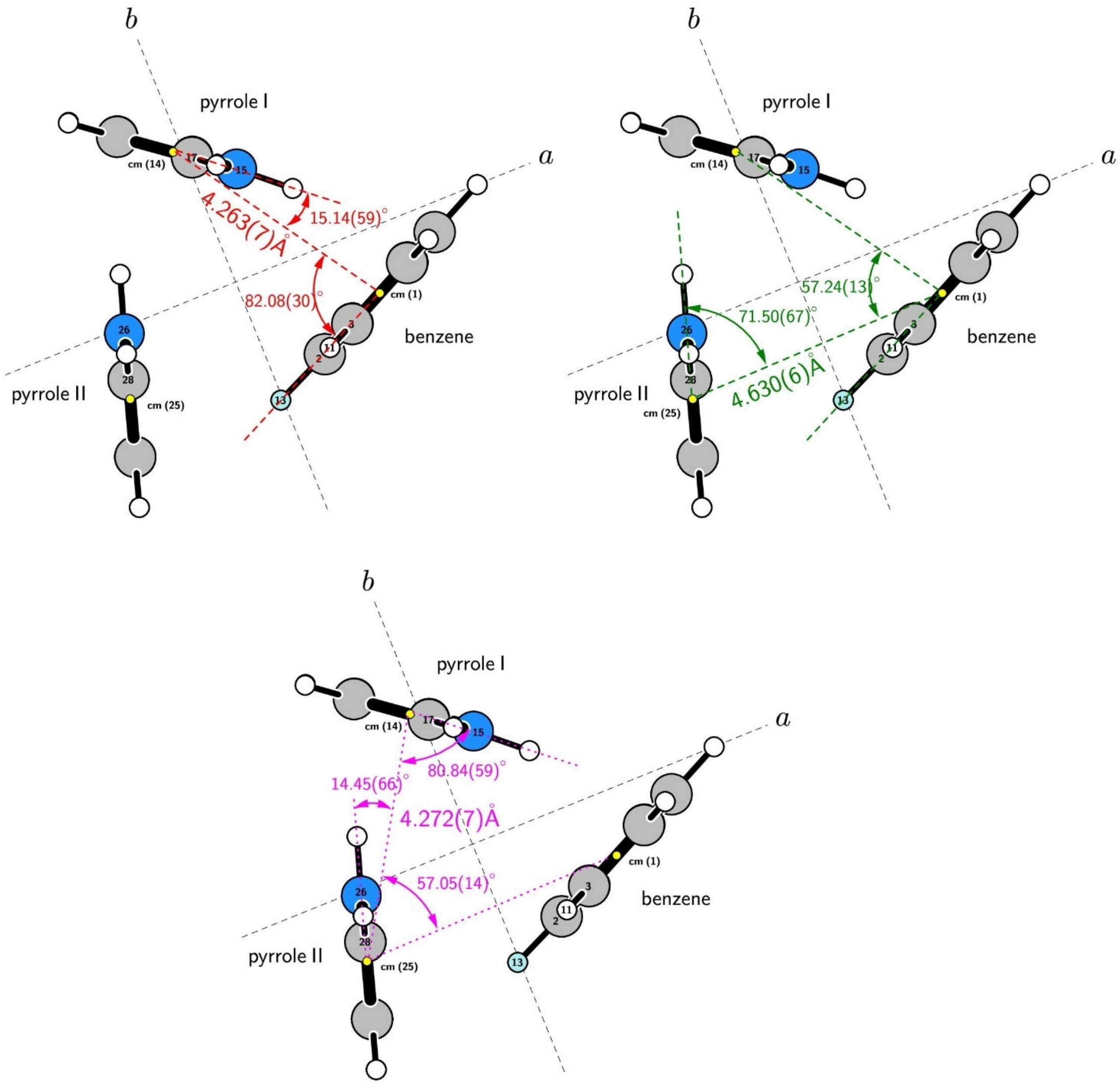

Figure S4. Structural parameters defining the relative orientation of the three molecules in the $(Py)_2$-Bz cluster determined from the parameters of the preferred structural fit performed with the STRFIT program. Parameter uncertainties are from propagation of uncertainties on the Cartesian coordinates from the fit, evaluated with the EVAL program.

Table S43. The abbreviated results of the least-squares fit of the partial geometry of the $(Py)_2$-Bz cluster with the STRFIT program.

```
 ______________________________________________________________________________
|                                                                              |
|    STRFIT - General structure fitting program using CART definitions         |
|______________________________________________________________________________|
 version 4.VII.2025                                            Zbigniew KISIEL

_______________________________________________________________________________

(Pyrrole)2-Benzene
_______________________________________________________________________________

!
!    starting structure =  published re^SE structures for both molecules
!

NUMBER OF ATOMS = 35   (including  3 dummy atoms)

  NO  NA  NB  NC       NO.NA     NO.NA.NB   NO.NA.NB.NC        MASS

   1   0   0   0     0.000000     0.000000     0.000000      0.0000000
   2   1   0   0     1.391400     0.000000     0.000000     12.0000000
   3   2   1   0     1.391400    60.000000     0.000000     12.0000000
   4   2   3   1     1.391400   120.000000     0.000000     12.0000000
   5   4   2   1     1.391400   120.000000     0.000000     12.0000000
   6   3   2   1     1.391400   120.000000     0.000000     12.0000000
   7   6   3   1     1.391400   120.000000     0.000000     12.0000000
   8   7   5   1     1.080200   120.000000  -180.000000      1.0078250
   9   5   4   1     1.080200   120.000000  -180.000000      1.0078250
  10   6   3   1     1.080200   120.000000   180.000000      1.0078250
  11   3   2   1     1.080200   120.000000   180.000000      1.0078250
  12   4   2   1     1.080200   120.000000  -180.000000      1.0078250
  13   2   1   4     1.080200   180.000000   -90.000000      1.0078250
  14   1   2   4     4.226896    81.399510    90.000000      0.0000000
  15  14   1   7     1.117136    19.591276     0.000000     14.0030740
  16  15  14   1     1.369400    54.904500    90.000000     12.0000000
  17  15  14   1     1.369400    54.904500   -90.000000     12.0000000
  18  17  15  14     1.372300   107.762000     0.000000     12.0000000
  19  16  15  14     1.372300   107.762000     0.000000     12.0000000
  20  15  14   1     1.000860   180.000000     0.000000      1.0078250
  21  16  15  14     1.075320   120.990000  -180.000000      1.0078250
  22  17  15  14     1.075320   120.990000   180.000000      1.0078250
  23  18  17  15     1.075270   125.940000  -180.000000      1.0078250
  24  19  16  15     1.075270   125.940000   180.000000      1.0078250
  25   1  14  15     4.612289    57.398661   180.000000      0.0000000
  26  25   1  14     1.117136    73.614080     0.000000     14.0030740
  27  26  25   1     1.369400    54.904500    90.000000     12.0000000
  28  26  25   1     1.369400    54.904500   -90.000000     12.0000000
  29  27  26  25     1.372300   107.762000     0.000000     12.0000000
  30  28  26  25     1.372300   107.762000     0.000000     12.0000000
  31  26  25   1     1.000860   180.000000     0.000000      1.0078250
  32  27  29  30     1.075320   131.250000   180.000000      1.0078250
  33  28  30  29     1.075320   131.250000  -180.000000      1.0078250
  34  29  27  26     1.075270   125.940000   180.000000      1.0078250
  35  30  28  26     1.075270   125.940000  -180.000000      1.0078250
!
!
!                    column:  32    38           51                   72                 91
!                              |     |            |                    |                  |
!                              --++--+++++++++++++                     ccccccccccccccccccc
!                                       value                              descriptor

---------------------------------------------
TOTAL NUMBER OF STRUCTURAL PARAMETERS:     9
---------------------------------------------
```

```
Parameters to be fitted:
          R(14, 1) =     4.265000
       A(14, 1, 2) =    82.060000
       A(15,14, 1) =    15.000000
          R(25, 1) =     4.628000
       A(25, 1,14) =    57.210000
       A(26,25, 1) =    71.000000
             c_c  =     0.000000

Fixed parameters:
             c_a  =     0.000000
             c_b  =     0.000000
---------------------------------------------

!
!
!
!                    column:  32  36            50            64            78
!                              |   |             |             |             |
!                              ++--++++++++++++++--------------++++++++++++++
!
!                                  expt.  B0       g_bb         calc Be-B0
!

-------------------------------------------------------------------------------------
TOTAL NUMBER OF SPECTROSCOPIC CONSTANTS:    63
```

Isotopic species		B_expt	Ib_expt	dI (or g_bb)	dB	dB_el	B_corr	Ib_corr
	A	575.75643	877.76529	0.00000	0.00000	0.00000	575.75643	877.76529
	B	467.37093	1081.32316	0.00000	0.00000	0.00000	467.37093	1081.32316
	C	323.31877	1563.09827	0.00000	0.00000	0.00000	323.31877	1563.09827
2	A	571.65860	884.05739	0.00000	0.00000	0.00000	571.65860	884.05739
	B	466.85500	1082.51815	0.00000	0.00000	0.00000	466.85500	1082.51815
	C	322.29022	1568.08671	0.00000	0.00000	0.00000	322.29022	1568.08671
3	A	571.65860	884.05739	0.00000	0.00000	0.00000	571.65860	884.05739
	B	466.85500	1082.51815	0.00000	0.00000	0.00000	466.85500	1082.51815
	C	322.29022	1568.08671	0.00000	0.00000	0.00000	322.29022	1568.08671
4	A	569.22850	887.83153	0.00000	0.00000	0.00000	569.22850	887.83153
	B	466.89201	1082.43234	0.00000	0.00000	0.00000	466.89201	1082.43234
	C	321.22358	1573.29362	0.00000	0.00000	0.00000	321.22358	1573.29362
5	A	569.22850	887.83153	0.00000	0.00000	0.00000	569.22850	887.83153
	B	466.89201	1082.43234	0.00000	0.00000	0.00000	466.89201	1082.43234
	C	321.22358	1573.29362	0.00000	0.00000	0.00000	321.22358	1573.29362
6	A	573.56200	881.12359	0.00000	0.00000	0.00000	573.56200	881.12359
	B	465.68316	1085.24218	0.00000	0.00000	0.00000	465.68316	1085.24218
	C	322.40419	1567.53239	0.00000	0.00000	0.00000	322.40419	1567.53239
7	A	574.40790	879.82601	0.00000	0.00000	0.00000	574.40790	879.82601
	B	463.29610	1090.83372	0.00000	0.00000	0.00000	463.29610	1090.83372
	C	321.54743	1571.70906	0.00000	0.00000	0.00000	321.54743	1571.70906
8	A	575.56180	878.06211	0.00000	0.00000	0.00000	575.56180	878.06211
	B	462.31116	1093.15771	0.00000	0.00000	0.00000	462.31116	1093.15771
	C	320.82592	1575.24370	0.00000	0.00000	0.00000	320.82592	1575.24370
9	A	574.40790	879.82601	0.00000	0.00000	0.00000	574.40790	879.82601
	B	463.29610	1090.83372	0.00000	0.00000	0.00000	463.29610	1090.83372
	C	321.54743	1571.70906	0.00000	0.00000	0.00000	321.54743	1571.70906
10	A	573.56200	881.12359	0.00000	0.00000	0.00000	573.56200	881.12359
	B	465.68316	1085.24218	0.00000	0.00000	0.00000	465.68316	1085.24218
	C	322.40419	1567.53239	0.00000	0.00000	0.00000	322.40419	1567.53239
11	A	573.90140	880.60250	0.00000	0.00000	0.00000	573.90140	880.60250
	B	467.12140	1081.90079	0.00000	0.00000	0.00000	467.12140	1081.90079
	C	322.53825	1566.88086	0.00000	0.00000	0.00000	322.53825	1566.88086
12	A	574.39320	879.84853	0.00000	0.00000	0.00000	574.39320	879.84853
	B	464.60350	1087.76410	0.00000	0.00000	0.00000	464.60350	1087.76410
	C	322.07527	1569.13323	0.00000	0.00000	0.00000	322.07527	1569.13323
13	A	572.61790	882.57634	0.00000	0.00000	0.00000	572.61790	882.57634
	B	464.01770	1089.13735	0.00000	0.00000	0.00000	464.01770	1089.13735
	C	320.91877	1574.78794	0.00000	0.00000	0.00000	320.91877	1574.78794
14	A	572.61790	882.57634	0.00000	0.00000	0.00000	572.61790	882.57634
	B	464.01770	1089.13735	0.00000	0.00000	0.00000	464.01770	1089.13735
	C	320.91877	1574.78794	0.00000	0.00000	0.00000	320.91877	1574.78794

```
15   A     574.39320  879.84853   0.00000    0.00000  0.00000    574.39320  879.84853
     B     464.60350 1087.76410   0.00000    0.00000  0.00000    464.60350 1087.76410
     C     322.07527 1569.13323   0.00000    0.00000  0.00000    322.07527 1569.13323
16   A     575.76578  877.75104   0.00000    0.00000  0.00000    575.76578  877.75104
     B     459.02864 1100.97490   0.00000    0.00000  0.00000    459.02864 1100.97490
     C     319.31291 1582.70773   0.00000    0.00000  0.00000    319.31291 1582.70773
17   A     572.66518  882.50347   0.00000    0.00000  0.00000    572.66518  882.50347
     B     460.66800 1097.05690   0.00000    0.00000  0.00000    460.66800 1097.05690
     C     320.97301 1574.52183   0.00000    0.00000  0.00000    320.97301 1574.52183
18   A     572.66518  882.50347   0.00000    0.00000  0.00000    572.66518  882.50347
     B     460.66800 1097.05690   0.00000    0.00000  0.00000    460.66800 1097.05690
     C     320.97301 1574.52183   0.00000    0.00000  0.00000    320.97301 1574.52183
19   A     571.09432  884.93090   0.00000    0.00000  0.00000    571.09432  884.93090
     B     464.87669 1087.12487   0.00000    0.00000  0.00000    464.87669 1087.12487
     C     322.51948 1566.97205   0.00000    0.00000  0.00000    322.51948 1566.97205
20   A     571.09432  884.93090   0.00000    0.00000  0.00000    571.09432  884.93090
     B     464.87669 1087.12487   0.00000    0.00000  0.00000    464.87669 1087.12487
     C     322.51948 1566.97205   0.00000    0.00000  0.00000    322.51948 1566.97205
21   A     572.62828  882.56034   0.00000    0.00000  0.00000    572.62828  882.56034
     B     467.81379 1080.29951   0.00000    0.00000  0.00000    467.81379 1080.29951
     C     322.54866 1566.83029   0.00000    0.00000  0.00000    322.54866 1566.83029
-------------------------------------------------------------------------------------
    B_corr = B_expt + dB - dB_el, Ib_corr=505379.01/B_corr
    g_bb is dimensionles, dB_el= = 0.000544617 * g_bb * B, where B=B_expt+dB
or
    Ib_corr = Ib_expt + dI,        B_corr =505379.01/Ib_corr


-------------------------------------------------------
DEFINITIONS OF SUBSTITUTED ISOTOPIC SPECIES
-------------------------------------------------------
!
!  Declarations of isotopic species
!
!
!    2 = 13C-1 (Pyr)
!
ISOTOPIC SPECIES  2,  changes from parent species:
atom no.,parameter no.,value    16  4       13.0033548
!
!    3 = 13C-3 (Pyr)
!
ISOTOPIC SPECIES  3,  changes from parent species:
atom no.,parameter no.,value    17  4       13.0033548
!
!    4 = 13C-4 (Pyr)
!
ISOTOPIC SPECIES  4,  changes from parent species:
atom no.,parameter no.,value    18  4       13.0033548
!
!    5 = 13C-5 (Pyr)
!
ISOTOPIC SPECIES  5,  changes from parent species:
atom no.,parameter no.,value    19  4       13.0033548
!
!    6 = 13C-6 (Bz)
!
ISOTOPIC SPECIES  6,  changes from parent species:
atom no.,parameter no.,value     3  4       13.0033548
!
!    7 = 13C-7 (Bz)
!
ISOTOPIC SPECIES  7,  changes from parent species:
atom no.,parameter no.,value     6  4       13.0033548
!
!    8 = 13C-8 (Bz)
!
ISOTOPIC SPECIES  8,  changes from parent species:
atom no.,parameter no.,value     7  4       13.0033548
!
!    9 = 13C-9 (Bz)
!
```

```
ISOTOPIC SPECIES  9,  changes from parent species:
atom no.,parameter no.,value     5  4       13.0033548
!
!   10 = 13C-10 (Bz)
!
ISOTOPIC SPECIES 10,  changes from parent species:
atom no.,parameter no.,value     4  4       13.0033548
!
!   11 = 13C-11 (Bz)
!
ISOTOPIC SPECIES 11,  changes from parent species:
atom no.,parameter no.,value     2  4       13.0033548
!
!   12 = 13C-23 (Pyr)
!
ISOTOPIC SPECIES 12,  changes from parent species:
atom no.,parameter no.,value    27  4       13.0033548
!
!   13 = 13C-25 (Pyr)
!
ISOTOPIC SPECIES 13,  changes from parent species:
atom no.,parameter no.,value    29  4       13.0033548
!
!   14 = 13C-26 (Pyr)
!
ISOTOPIC SPECIES 14,  changes from parent species:
atom no.,parameter no.,value    30  4       13.0033548
!
!   15 = 13C-27 (Pyr)
!
ISOTOPIC SPECIES 15,  changes from parent species:
atom no.,parameter no.,value    28  4       13.0033548
!
!   16 = D (12)
!
ISOTOPIC SPECIES 16,  changes from parent species:
atom no.,parameter no.,value     8  4        2.0141018
!
!   17 = D (13)
!
ISOTOPIC SPECIES 17,  changes from parent species:
atom no.,parameter no.,value     9  4        2.0141018
!
!   18 = D (15)
!
ISOTOPIC SPECIES 18,  changes from parent species:
atom no.,parameter no.,value    10  4        2.0141018
!
!   19 = D (14)
!
ISOTOPIC SPECIES 19,  changes from parent species:
atom no.,parameter no.,value    11  4        2.0141018
!
!   20 = D (16)
!
ISOTOPIC SPECIES 20,  changes from parent species:
atom no.,parameter no.,value    12  4        2.0141018
!
!   21 = D (24)
!
ISOTOPIC SPECIES 21,  changes from parent species:
atom no.,parameter no.,value    13  4        2.0141018
                                25  1       -0.0030000
atom no.,parameter no.,value    25  2        0.0000000
-------------------------------------------------------

fit after:   6 iterations,  ALAMDA=  0.10E-08

   Number of fitted spectroscopic constants = 63
                Number of parameters of fit =  7
               Number of degrees of freedom = 56
```

```
FINAL RESULTS OF LEAST SQUARES FIT:

        R(14, 1) =     4.262722 +-  0.003117   PAcm-Bcm       4.262
     A(14, 1, 2) =    82.077437 +-  0.176314   PAcm-Bcm-B_C  82.06
     A(15,14, 1) =    15.135296 +-  0.639153   PA_N-PAcm-Bcm 19.591
        R(25, 1) =     4.629726 +-  0.002284   PBcm-Bcm       4.629
     A(25, 1,14) =    57.242736 +-  0.057443   PBcm-Bcm-B-C  57.243
     A(26,25, 1) =    71.503717 +-  0.568268   PB_N-PBcm-Bcm 71.503
             c_a  = [   0.000000 ]    FIXED
             c_b  = [   0.000000 ]    FIXED
             c_c  =    -0.001370 +-  0.000751

     Chi-squared =        0.3472266929     = Sum( (Iobs-calc)**2 )
 Deviation of fit =        0.078743 uA^2    = Sqrt(Chisq/Ndegf),  Ndegf= 56

 Note that the fit is to moments of inertia but it also corresponds to:
 Deviation of fit =        0.035697 MHz     = Sqrt( Sum( (Bo-c)**2 )/Ndegf )
```

Ni	Axis	Iobs	Icalc	Io-c	Bobs	Bcalc	Bo-c
1	a	877.76529	877.78681	-0.02152	575.75643	575.74232	0.01411
1	b	1081.32316	1081.22556	0.09759	467.37093	467.41312	-0.04219
1	c	1563.09827	1563.03465	0.06362	323.31877	323.33193	-0.01316
2	a	884.05739	884.02062	0.03677	571.65860	571.68238	-0.02378
2	b	1082.51815	1082.47249	0.04566	466.85500	466.87469	-0.01969
2	c	1568.08671	1568.03372	0.05299	322.29022	322.30111	-0.01089
3	a	884.05739	884.02062	0.03677	571.65860	571.68238	-0.02378
3	b	1082.51815	1082.47249	0.04566	466.85500	466.87469	-0.01969
3	c	1568.08671	1568.03372	0.05299	322.29022	322.30111	-0.01089
4	a	887.83153	887.77517	0.05636	569.22850	569.26464	-0.03614
4	b	1082.43234	1082.59857	-0.16623	466.89201	466.82032	0.07169
4	c	1573.29362	1573.40533	-0.11170	321.22358	321.20077	0.02281
5	a	887.83153	887.77517	0.05636	569.22850	569.26464	-0.03614
5	b	1082.43234	1082.59857	-0.16623	466.89201	466.82032	0.07169
5	c	1573.29362	1573.40533	-0.11170	321.22358	321.20077	0.02281
6	a	881.12359	881.13055	-0.00696	573.56200	573.55747	0.00453
6	b	1085.24218	1085.23164	0.01055	465.68316	465.68769	-0.00453
6	c	1567.53239	1567.50657	0.02581	322.40419	322.40950	-0.00531
7	a	879.82601	879.79448	0.03153	574.40790	574.42849	-0.02059
7	b	1090.83372	1090.76632	0.06740	463.29610	463.32473	-0.02863
7	c	1571.70906	1571.72025	-0.01119	321.54743	321.54514	0.00229
8	a	878.06211	877.99195	0.07016	575.56180	575.60779	-0.04599
8	b	1093.15771	1093.24060	-0.08288	462.31116	462.27611	0.03505
8	c	1575.24370	1575.25462	-0.01091	320.82592	320.82370	0.00222
9	a	879.82601	879.79448	0.03153	574.40790	574.42849	-0.02059
9	b	1090.83372	1090.76632	0.06740	463.29610	463.32473	-0.02863
9	c	1571.70906	1571.72025	-0.01119	321.54743	321.54514	0.00229
10	a	881.12359	881.13055	-0.00696	573.56200	573.55747	0.00453
10	b	1085.24218	1085.23164	0.01055	465.68316	465.68769	-0.00453
10	c	1567.53239	1567.50657	0.02581	322.40419	322.40950	-0.00531
11	a	880.60250	880.64188	-0.03937	573.90140	573.87574	0.02566
11	b	1081.90079	1082.17832	-0.27753	467.12140	467.00160	0.11980
11	c	1566.88086	1566.84240	0.03845	322.53825	322.54617	-0.00792
12	a	879.84853	879.77613	0.07239	574.39320	574.44046	-0.04726
12	b	1087.76410	1087.83895	-0.07485	464.60350	464.57153	0.03197
12	c	1569.13323	1569.15329	-0.02006	322.07527	322.07115	0.00412
13	a	882.57634	882.64610	-0.06976	572.61790	572.57264	0.04526
13	b	1089.13735	1089.15991	-0.02255	464.01770	464.00809	0.00961
13	c	1574.78794	1574.83660	-0.04866	320.91877	320.90885	0.00992
14	a	882.57634	882.64610	-0.06976	572.61790	572.57264	0.04526
14	b	1089.13735	1089.15991	-0.02255	464.01770	464.00809	0.00961
14	c	1574.78794	1574.83660	-0.04866	320.91877	320.90885	0.00992
15	a	879.84853	879.77613	0.07239	574.39320	574.44046	-0.04726
15	b	1087.76410	1087.83895	-0.07485	464.60350	464.57153	0.03197
15	c	1569.13323	1569.15329	-0.02006	322.07527	322.07115	0.00412
16	a	877.75104	877.78691	-0.03587	575.76578	575.74225	0.02353
16	b	1100.97490	1100.93514	0.03976	459.02864	459.04522	-0.01658
16	c	1582.70773	1582.74398	-0.03625	319.31291	319.30560	0.00731
17	a	882.50347	882.57362	-0.07015	572.66518	572.61966	0.04552
17	b	1097.05690	1096.91418	0.14273	460.66800	460.72794	-0.05994
17	c	1574.52183	1574.48510	0.03672	320.97301	320.98050	-0.00749

18	a	882.50347	882.57362	-0.07015	572.66518	572.61966	0.04552
18	b	1097.05690	1096.91418	0.14273	460.66800	460.72794	-0.05994
18	c	1574.52183	1574.48510	0.03672	320.97301	320.98050	-0.00749
19	a	884.93090	884.99904	-0.06814	571.09432	571.05035	0.04397
19	b	1087.12487	1087.03818	0.08668	464.87669	464.91376	-0.03707
19	c	1566.97205	1566.94929	0.02276	322.51948	322.52416	-0.00468
20	a	884.93090	884.99904	-0.06814	571.09432	571.05035	0.04397
20	b	1087.12487	1087.03818	0.08668	464.87669	464.91376	-0.03707
20	c	1566.97205	1566.94929	0.02276	322.51948	322.52416	-0.00468
21	a	882.56034	882.49838	0.06197	572.62828	572.66849	-0.04021
21	b	1080.29951	1080.25712	0.04239	467.81379	467.83215	-0.01836
21	c	1566.83029	1566.77771	0.05258	322.54866	322.55948	-0.01082

Correlation coefficients:

		1	2	3	4	5	6	7
1:	R(14, 1)	1.000						
2:	A(14, 1, 2)	-0.068	1.000					
3:	A(15,14, 1)	0.188	-0.092	1.000				
4:	R(25, 1)	-0.645	0.389	-0.790	1.000			
5:	A(25, 1,14)	-0.580	-0.326	0.605	-0.248	1.000		
6:	A(26,25, 1)	-0.842	0.105	0.053	0.508	0.527	1.000	
7:	c_c	-0.011	-0.001	-0.001	-0.012	-0.001	0.000	1.000

Final principal coordinates of parent:

ATOM NO.	A	B	C	MASS
1	-2.218389	1.080621	0.000000	0.0000000
2	-0.969882	1.694807	0.000000	12.0000000
3	-1.594135	1.387714	1.204988	12.0000000
4	-1.594135	1.387714	-1.204988	12.0000000
5	-2.842642	0.773528	-1.204988	12.0000000
6	-2.842642	0.773528	1.204988	12.0000000
7	-3.466896	0.466435	0.000000	12.0000000
8	-4.436162	-0.010383	0.000000	1.0078250
9	-3.327275	0.535119	-2.140468	1.0078250
10	-3.327275	0.535119	2.140468	1.0078250
11	-1.109502	1.626123	2.140468	1.0078250
12	-1.109502	1.626123	-2.140468	1.0078250
13	-0.000616	2.171624	0.000000	1.0078250
14	0.172495	-2.448468	0.000000	0.0000000
15	-0.673835	-1.719278	0.000000	14.0030740
16	-0.077366	-2.233190	-1.120436	12.0000000
17	-0.077366	-2.233190	1.120436	12.0000000
18	0.915060	-3.088255	0.711583	12.0000000
19	0.915060	-3.088255	-0.711583	12.0000000
20	-1.432075	-1.065985	0.000000	1.0078250
21	-0.407604	-1.948660	-2.103441	1.0078250
22	-0.407604	-1.948660	2.103441	1.0078250
23	1.567971	-3.650797	1.354590	1.0078250
24	1.567971	-3.650797	-1.354590	1.0078250
25	2.410034	1.190468	0.000000	0.0000000
26	2.080867	0.122928	0.000000	14.0030740
27	2.312854	0.875298	-1.120436	12.0000000
28	2.312854	0.875298	1.120436	12.0000000
29	2.698843	2.127121	-0.711583	12.0000000
30	2.698843	2.127121	0.711583	12.0000000
31	1.785960	-0.833498	0.000000	1.0078250
32	2.184403	0.458712	-2.103426	1.0078250
33	2.184403	0.458712	2.103426	1.0078250
34	2.952784	2.950687	-1.354590	1.0078250
35	2.952784	2.950687	1.354590	1.0078250

Principal coordinates and estimated uncertainties:

ATOM NO.	A	dA	B	dB	C	dC	MASS
1	-2.21839	0.00396	1.08062	0.00541	0.00000	0.00000	0.000000
2	-0.96988	0.00638	1.69481	0.00442	0.00000	0.00000	12.000000

3	-1.59414	0.00512	1.38771	0.00404	1.20499	0.00000	12.000000
4	-1.59414	0.00512	1.38771	0.00404	-1.20499	0.00000	12.000000
5	-2.84264	0.00301	0.77353	0.00764	-1.20499	0.00000	12.000000
6	-2.84264	0.00301	0.77353	0.00764	1.20499	0.00000	12.000000
7	-3.46690	0.00252	0.46643	0.01017	0.00000	0.00000	12.000000
8	-4.43616	0.00315	-0.01038	0.01434	0.00000	0.00000	1.007825
9	-3.32728	0.00258	0.53512	0.00959	-2.14047	0.00000	1.007825
10	-3.32728	0.00258	0.53512	0.00959	2.14047	0.00000	1.007825
11	-1.10950	0.00609	1.62612	0.00417	2.14047	0.00000	1.007825
12	-1.10950	0.00609	1.62612	0.00417	-2.14047	0.00000	1.007825
13	-0.00062	0.00841	2.17162	0.00751	0.00000	0.00000	1.007825
14	0.17250	0.00887	-2.44847	0.00163	0.00000	0.00000	0.000000
15	-0.67383	0.01148	-1.71928	0.01010	0.00000	0.00000	14.003074
16	-0.07737	0.00909	-2.23319	0.00402	-1.12044	0.00000	12.000000
17	-0.07737	0.00909	-2.23319	0.00402	1.12044	0.00000	12.000000
18	0.91506	0.01118	-3.08825	0.00639	0.71158	0.00000	12.000000
19	0.91506	0.01118	-3.08825	0.00639	-0.71158	0.00000	12.000000
20	-1.43208	0.01660	-1.06599	0.01790	0.00000	0.00000	1.007825
21	-0.40760	0.01015	-1.94866	0.00737	-2.10344	0.00000	1.007825
22	-0.40760	0.01015	-1.94866	0.00737	2.10344	0.00000	1.007825
23	1.56797	0.01542	-3.65080	0.01308	1.35459	0.00000	1.007825
24	1.56797	0.01542	-3.65080	0.01308	-1.35459	0.00000	1.007825
25	2.41003	0.00458	1.19047	0.00577	0.00000	0.00000	0.000000
26	2.08087	0.01263	0.12293	0.00706	0.00000	0.00000	14.003074
27	2.31285	0.00644	0.87530	0.00602	-1.12044	0.00000	12.000000
28	2.31285	0.00644	0.87530	0.00602	1.12044	0.00000	12.000000
29	2.69884	0.00777	2.12712	0.00584	-0.71158	0.00000	12.000000
30	2.69884	0.00777	2.12712	0.00584	0.71158	0.00000	12.000000
31	1.78596	0.02121	-0.83350	0.00897	0.00000	0.00000	1.007825
32	2.18440	0.00974	0.45871	0.00653	-2.10343	0.00000	1.007825
33	2.18440	0.00974	0.45871	0.00653	2.10343	0.00000	1.007825
34	2.95278	0.01481	2.95069	0.00682	-1.35459	0.00000	1.007825
35	2.95278	0.01481	2.95069	0.00682	1.35459	0.00000	1.007825

NOTES: 1/ only the uncertainties for those coordinates which are completely defined by the fitted internals should be trusted
2/ the uncertainties are somewhat limited by the linear approximation coord=(d coord/d parameter)*parameter used for evaluation
3/ only the effect of the internals R, A, and D is propagated

---

Terms in I.fitted = I.rigid + eps

Ni	(I_a)rig	(I_b)rig	(I_c)rig	eps_a	eps_b^**	eps_c	Modified atom
1	877.78681	1081.22556	1563.08879	0.00000	0.00000	-0.05414	
2	884.02062	1082.47249	1568.08795	0.00000	0.00000	-0.05423	16
3	884.02062	1082.47249	1568.08795	0.00000	0.00000	-0.05423	17
4	887.77517	1082.59857	1573.45965	0.00000	0.00000	-0.05432	18
5	887.77517	1082.59857	1573.45965	0.00000	0.00000	-0.05432	19
6	881.13055	1085.23164	1567.56080	0.00000	0.00000	-0.05422	3
7	879.79448	1090.76632	1571.77454	0.00000	0.00000	-0.05429	6
8	877.99195	1093.24060	1575.30897	0.00000	0.00000	-0.05436	7
9	879.79448	1090.76632	1571.77454	0.00000	0.00000	-0.05429	5
10	881.13055	1085.23164	1567.56080	0.00000	0.00000	-0.05422	4
11	880.64188	1082.17832	1566.89661	0.00000	0.00000	-0.05421	2
12	879.77613	1087.83895	1569.20754	0.00000	0.00000	-0.05425	27
13	882.64610	1089.15991	1574.89095	0.00000	0.00000	-0.05435	29
14	882.64610	1089.15991	1574.89095	0.00000	0.00000	-0.05435	30
15	879.77613	1087.83895	1569.20754	0.00000	0.00000	-0.05425	28
16	877.78691	1100.93514	1582.79846	0.00000	0.00000	-0.05449	8
17	882.57363	1096.91418	1574.53944	-0.00001	0.00000	-0.05434	9
18	882.57363	1096.91418	1574.53944	-0.00001	0.00000	-0.05434	10
19	884.99904	1087.03819	1567.00350	0.00000	0.00000	-0.05421	11
20	884.99904	1087.03819	1567.00350	0.00000	0.00000	-0.05421	12
21	882.49838	1080.25712	1566.83192	0.00000	0.00000	-0.05421	13 25 25

** Only this value is the total EPSILON for the degenerate constant in linear and symmetric tops, the other values do not contain the rm() c and d contributions.

Table S44. Substitution coordinates (r_s) of the heavy atoms (C) from the general Kraitchman equations using the rotational constants of the single ^{13}C isotopologues from Table S40.

	a	δ*a*	*b*	δ*b*	*c*	δ*c*
C(1,3)	0.23014·*i**	0.00788?	2.24394	0.00081	1.12429	0.00163
C(4,5)	0.76838	0.00219	3.09979	0.00055	0.70775	0.00242
C(6,10)	1.57223	0.00104	1.39905	0.00117	1.19770	0.00137
C(7,9)	2.82862	0.00061	0.77611	0.00226	1.22534	0.00142
C(8)	3.44096	0.00050	0.56743	0.00308	0.08464·*i**	0.02043?
C(11)	0.86685	0.00225	1.74290	0.00113	0.43068·*i**	0.00456?
C(23,27)	2.27434	0.00085	0.92682	0.00210	1.12158	0.00173
C(25,26)	2.68228	0.00087	2.12175	0.00114	0.69117	0.00348

*set to zero for distance evaluation.

Table S45. Substitution coordinates (r_s) of the H from the H atoms from the general Kraitchman equations using the rotational constants of the single D isotopologues from Table S41.

	a	δ*a*	*b*	δ*b*	*c*	δ*c*
H(12)	4.42833	0.00035	0.17592·*i**	0.00897?	0.11997	0.01308
H(13,15)	3.33270	0.00046	0.47257	0.00327	2.14318	0.00072
H(14,16)	1.10847	0.00139	1.61435	0.00095	2.13843	0.00072
H(24)	1.00860·*i**	0.00155?	2.17795	0.00072	0.14081	0.01110

*set to zero for distance evaluation.

3 $(Py)_2$-$(Bz)_2$ tetramer

3.1 Measured rotational transitions and fits

Table S46. Measured rotational transitions (ν_{obs}) of the $^{12}C/^{14}N/^{1}H$ isotopic species of the $(Py)_2$-$(Bz)_2$ tetramer and residuals (ν_{obs} - ν_{calc}) (frequencies in MHz).

J'	*K*a'	*K*c'	←	*J''*	*K*a''	*K*c''	ν_{obs}	ν_{obs}-ν_{calc}
14	0	14	←	13	0	13	4230.0875	-0.0188
11	0	11	←	10	0	10	3347.8923	0.0178
21	0	21	←	20	0	20	6296.3625	0.0082
22	0	22	←	21	0	21	6591.7625	0.0039
15	0	15	←	14	0	14	4524.9000	0.0019
20	0	20	←	19	0	19	6000.9875	0.0188
16	0	16	←	15	0	15	4819.8875	-0.0135
17	0	17	←	16	0	16	5115.0500	0.0000
18	0	18	←	17	0	17	5410.3000	0.0021
11	1	11	←	10	1	10	3337.0750	0.0161
12	1	12	←	11	1	11	3634.1000	-0.0050
13	1	13	←	12	1	12	3930.6500	-0.0043
14	1	14	←	13	1	13	4226.8250	-0.0178
15	1	15	←	14	1	14	4522.7625	-0.0148
16	1	16	←	15	1	15	4818.5375	-0.0002
17	1	17	←	16	1	16	5114.2000	0.0186
18	1	18	←	17	1	17	5409.7625	0.0141
19	1	19	←	18	1	18	5705.2750	0.0089
11	1	10	←	10	1	9	3556.1125	-0.0052
12	1	11	←	11	1	10	3853.7375	0.0136
14	1	13	←	13	1	12	4438.5500	0.0134
15	1	14	←	14	1	13	4728.8375	0.0062
16	1	15	←	15	1	14	5019.2500	-0.0047
17	1	16	←	16	1	15	5310.3500	-0.0045
18	1	17	←	17	1	16	5602.2750	-0.0294
20	1	19	←	19	1	18	6188.4750	-0.0265
21	1	21	←	20	1	20	6296.2250	0.0052
21	1	20	←	20	1	19	6482.4750	0.0069
22	1	21	←	21	1	20	6776.8375	0.0088
23	1	22	←	22	1	21	7071.5125	0.0376
24	1	24	←	23	1	23	7182.5375	-0.0296
16	2	15	←	15	2	14	4992.7375	0.090
17	2	16	←	16	2	15	5291.1625	0.0169
18	2	17	←	17	2	16	5588.7500	-0.0087
19	2	18	←	18	2	17	5885.6875	-0.0029
21	2	20	←	20	2	19	6478.1375	-0.0321
15	2	13	←	14	2	12	4933.8125	0.0001
16	2	14	←	15	2	13	5238.3500	0.0129
17	2	15	←	16	2	14	5536.6875	-0.0204
20	2	18	←	19	2	17	6407.6375	0.0105

Table S47. Experimental rotational parameters of the $^{12}C/^{14}N/^{1}H$ (normal) isotopic species of the $(Py)_2$-$(Bz)_2$ tetramer.

	$^{12}C/^{14}N/^{1}H$
A / MHz	477.877(57)
B / MHz	173.1012(57)
C / MHz	147.73408(90)
Δ_J / kHz	0.1789(80)
Δ_{JK} / kHz	-2.28(64)
Δ_K/ kHz	105.7(78)
δ_J / kHz	0.0888(42)
δ_K / kHz	1.36(37)
N_{lines}	40
σ_{fit} / kHz	15.5

4 Theoretical calculations

4.1 Methodology

Structures were filtered through a computational funnel protocol, whereby increasingly costly calculations were performed on low energy structures.[1] First, the potential energy surfaces of each complex were sampled using OGOLEM,[2] an evolutionary genetic algorithm, and the semiempirical methods GFN2-xTB[3-4] and PM7[5]. Each potential energy surface was sampled four times per cluster per semiempirical method, with a pool size of 1000 and 20,000 global iterations. Unique structures, those with energy differences larger than 0.2 kcal/mol and rotational constants different by more than 5%, were optimized in Gaussian 16[6] with ωB97X-D[7-9] and the 6-31++G**[10-13] basis set. This method is widely used in studies of atmospheric clusters,[1] and reproduces geometries of water clusters well.[14] Conformational sampling identified 1652 (Py-Bz_2), 597 (Py_2-Bz), 1200 (Py_2-Bz_2), 1387 (Py_3-Bz), and 2458 (Bz_3-Py) unique structures. After optimizing these with ωB97X-D/6-31+G*, there were 53 (Py-Bz_2), 43 (Py_2-Bz), 206 (Py_2-Bz_2), 229 (Py_3-Bz), and 186 (Bz_3-Py) unique structures. DLPNO-CCSD(T) energies were computed for those with DFT electronic energies within 6 kcal/mol of the global minimum structure. Electronic energies were recomputed with domain-based local pair natural orbital coupled-cluster with single, double, and perturbative triple excitations (DLPNO-CCSD(T))[15-18] in ORCA[19] using Dunning's augmented correlation-consistent basis sets[20-21] at the quadruple-zeta level. Augmentation of diffuse functions was applied to heavy, non-hydrogen atoms only, thus the basis sets are referred to as haug-cc-pVQZ.

Enthalpic and entropic contributions to the Gibbs free energies were obtained by the ωB97X-D/6-31++G** frequencies and the thermo.pl script from the National Institute of Science and Technology. The frequencies were scaled by 0.971[22] to account for anharmonicity. The Boltzmann-weighted populations for each conformer were computed according to

$$BP = \frac{e^{-\Delta G°_i/RT}}{\sum_i e^{-\Delta G°_i/RT}},$$

where $\Delta G°_i$ is the Gibbs free energy of conformer *i* relative to the global minimum, *R* is the gas constant, and *T* is temperature. In Tables S48-S52 and Figures S5-S9, we report structures and properties of the isomers with relative Boltzmann populations over 0.5%.

The binding energies of monomer A, $E_{bind}(A)$, were computed according to

$$E_{bind}(A) = E_{el}(ABC) - E_{el}(BC) - E_{el}(A)$$

for the trimers, where $E_{el}(ABC)$ is the electronic energy of the trimer with monomers A, B and C, $E_{el}(BC)$ is the electronic energy of the dimer with monomers B and C, and $E_{el}(A)$ is the electronic energy of monomer A. This expression can similarly be written for the tetramers, composed of monomers A, B, C, and D:

$$E_{bind}(A) = E_{el}(ABCD) - E_{el}(BCD) - E_{el}(A)$$

For the minimum energy structures of $(Py)_2$-Bz, Py-$(Bz)_2$, and $(Py)_2$-$(Bz)_2$, we also computed the many-body expansion, using non-additive interaction energies and assuming rigid monomers. The two-body interaction energy is

$$E_{2B} = \sum_{i<j} \Delta E(i,j) = \sum_{i<j} E_{el}(i,j) - E_{el}(i) - E_{el}(j),$$

the non-additive three-body interaction energy is

$$E_{3B} = \sum_{i<j<k} \Delta E(i,j,k) - \Delta E(i,j) - \Delta E(j,k) - \Delta E(i,k),$$

and the non-additive four-body interaction energy is

$$E_{4B} = \sum_{i<j<k<l} \Delta E(i,j,k,l) - E_{3B}.$$

The observed trimers contained a mirror plane, thus were of the C_s point group. To obtain the C_s $(Py)_2$-Bz and Py-$(Bz)_2$ trimers computationally, we symmetrized the lowest-energy asymmetric trimers in GaussView and optimized the transition states in Gaussian with wB97X-D/6-31++G** and the superfine integration grid. The C_s geometry of $(Py)_2$-Bz is a transition state with one imaginary frequency. The C_s geometry of Py-$(Bz)_2$ is a third order saddle point with imaginary frequencies 23.4i, 22.5i, and 9.7i cm^{-1}. All three motions have been visualized and correspond to the cluster breaking the mirror plane. A true transition state (with one imaginary frequency) between the two asymmetric structures was located, but this transition state has C_1 symmetry according to wB97X-D. Finally, a C_s geometry was located for $(Py)_2$-$(Bz)_2$ with three imaginary frequencies, 27.7i, 7.0i, and 3.4i cm^{-1}.

References

1. Elm, J.; Ayoubi, D.; Engsvang, M.; Jensen, A. B.; Knattrup, Y.; Kubečka, J.; Bready, C. J.; Fowler, V. R.; Harold, S. E.; Longsworth, O. M.; et al. Quantum chemical modeling of organic enhanced atmospheric nucleation: A critical review. *WIREs Comput Mol Sci.* **2023**, 13 (5), e1662.
2. Dieterich, J. M.; Hartke, B., OGOLEM: Global cluster structure optimisation for arbitrary mixtures of flexible molecules. A multiscaling, object-oriented approach. *Mol. Phys.* **2010**, 108, 279-291.
3. Grimme, S., Exploration of Chemical Compound, Conformer, and Reaction Space with Meta-Dynamics Simulations Based on Tight-Binding Quantum Chemical Calculations. *J Chem Theory Comput.* **2019**, 15, 2847-2862.
4. Bannwarth, C.; Ehlert, S.; Grimme, S., GFN2-xTB-An Accurate and Broadly Parametrized Self-Consistent Tight-Binding Quantum Chemical Method with Multipole Electrostatics and Density-Dependent Dispersion Contributions. *J Chem Theory Comput.* **2019**, 15, 1652-1671.
5. Stewart, JJ., Optimization of parameters for semiempirical methods VI: more modifications to the NDDO approximations and re-optimization of parameters. *J Mol Model.* **2013**; 19, 1-32.
6. Frisch, M. J.; Trucks, G. W.; Schlegel, H. B.; Scuseria, G. E.; Robb, M. A.; Cheeseman, J. R.; Scalmani, G.; Barone, V.; Petersson, G. A.; Nakatsuji, H. L., X., et al. Gaussian 16, Revision B.01; Gaussian Inc: Wallingford, CT, 2016.
7. Chai, J. D.; Head-Gordon, M., Systematic optimization of long-range corrected hybrid density functionals. *J. Chem. Phys.* **2008**, 128, 084106.
8. Chai, J. D.; Head-Gordon, M., Long-range corrected hybrid density functionals with damped atom-atom dispersion corrections. *Phys Chem Chem Phys* **2008**, 10, 6615-6620.

9. Grimme, S.; Antony, J.; Ehrlich, S.; Krieg, H., A consistent and accurate ab initio parametrization of density functional dispersion correction (DFT-D) for the 94 elements H-Pu. *J. Chem. Phys.* **2010**, 132, 154104.
10. Hariharan, P. C.; Pople, J. A., The influence of polarization functions on molecular orbital hydrogenation energies. *Theoretica chimica acta* **1973**, 28, 213-222.
11. Ditchfield, R.; Hehre, W. J.; Pople, J. A., Self-Consistent Molecular-Orbital Methods. IX. An Extended Gaussian-Type Basis for Molecular-Orbital Studies of Organic Molecules. *J. Chem. Phys.* **1971**, 54, 724-728.
12. Hehre, W. J.; Ditchfield, R.; Pople, J. A., Self—Consistent Molecular Orbital Methods. XII. Further Extensions of Gaussian—Type Basis Sets for Use in Molecular Orbital Studies of Organic Molecules. *J. Chem. Phys.* **1972**, 56, 2257-2261.
13. Frisch, M. J.; Pople, J. A.; Binkley, J. S., Self-consistent molecular orbital methods 25. Supplementary functions for Gaussian basis sets. *J. Chem. Phys.* **1984**, 80, 3265-3269.
14. Petty, B.T., Fowler, V.R., Ryu, A., Glick, C.S., Rock, C.A., Wange, Q., Tschumper, G.S., Shields, G.C. Reliable Structures and Electronic Energies of Small Water Clusters Using Density Functional and Local Correlation Coupled Cluster Model Chemistries. *J. Phys. Chem. A* **2025**, 129, 9291-9302.
15. Riplinger, C.; Pinski, P.; Becker, U.; Valeev, E. F.; Neese, F., Sparse maps--A systematic infrastructure for reduced-scaling electronic structure methods. II. Linear scaling domain based pair natural orbital coupled cluster theory. *J. Chem. Phys.* **2016**, 144, 024109.
16. Liakos, D. G.; Sparta, M.; Kesharwani, M. K.; Martin, J. M. L.; Neese, F., Exploring the Accuracy Limits of Local Pair Natural Orbital Coupled-Cluster Theory. *J Chem Theory Comput.* **2015**, 11, 1525-1539.
17. Riplinger, C.; Sandhoefer, B.; Hansen, A.; Neese, F., Natural triple excitations in local coupled cluster calculations with pair natural orbitals. *J. Chem. Phys.* **2013**, 139, 134101.
18. Riplinger, C.; Neese, F., An efficient and near linear scaling pair natural orbital based local coupled cluster method. *J. Chem. Phys.* **2013**, 138, 034106.
19. Neese, F.; Wennmohs, F.; Becker, U.; Riplinger, C., The ORCA quantum chemistry program package. *J. Chem. Phys.* **2020**, 152, 224108.
20. Dunning, T. H. Gaussian basis sets for use in correlated molecular calculations. I. The atoms boron through neon and hydrogen. *J. Chem. Phys.* **1989**, *90* (2), 1007–1023.
21. Kendall, R. A.; Dunning, T. H., Jr.; Harrison, R. J. Electron affinities of the first-row atoms revisited. Systematic basis sets and wave functions. *J. Chem. Phys.* **1992**, *96* (9), 6796–6806.
22. Kanchanakungwankul, S.; Bao, J. L.; Alecu, I. M.; Lynch, B. J.; Zhao, Y.; Truhlar, D. G. *Database of Frequency Scale Factors for Electronic Model Chemistries*. 2021. https://comp.chem.umn.edu/freqscale/210722_Database_of_Freq_Scale_Factors_v5.pdf

4.2 Py-$(Bz)_2$

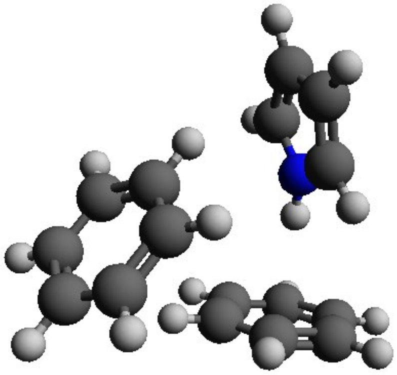

PM7-Pyr-Bz.3-69-19695

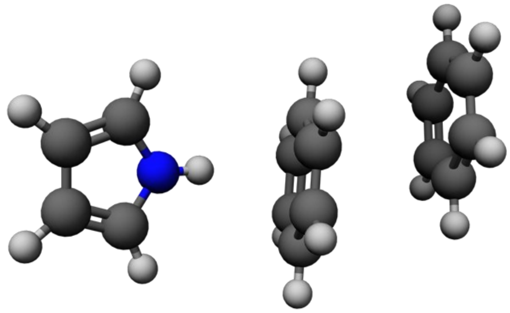

Pyr-Bz-63-15155

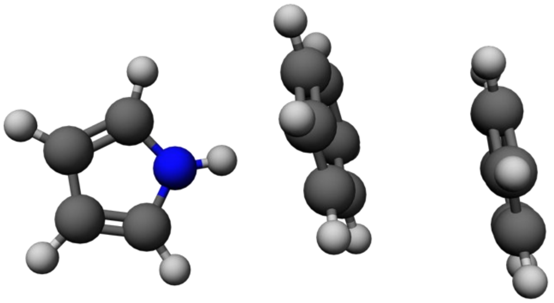

Pyr-Bz.2-62-18973

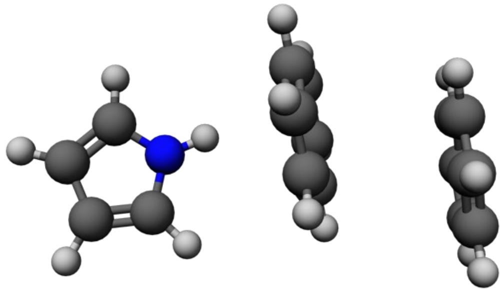

Pyr-Bz.3-108-20657

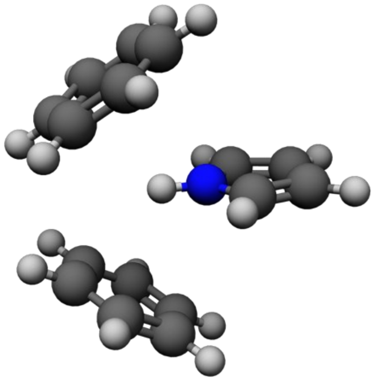

Pyr-Bz-0-16021

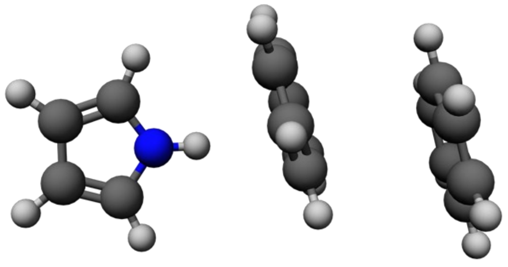

Pyr-Bz-74-20153

Figure S5. The six lowest-energy isomers of Py-$(Bz)_2$.

Table S48. Calculated energies, rotational constants, and principal axis dipole moments of Py-$(Bz)_2$. Electronic energies, Gibbs free energies, and Boltzmann populations at 100 K are relative to the global-minimum isomer sampled.

Energetics

Structure	ΔE_{el} (kcal/mol)	ΔG 100 K (kcal/mol)	Relative Boltzmann Population 100 K
PM7-Pyr-Bz.3-69-19695	0.00	0.00	100 %
Pyr-Bz-63-15155	3.68	1.83	0.01%
Pyr-Bz.2-62-18973	3.65	2.00	0.00%
Pyr-Bz.3-108-20657	3.67	2.05	0.00%
Pyr-Bz-0-16021	2.59	2.10	0.00%
Pyr-Bz-74-20153	3.59	2.10	0.00%

Rotational Constants (MHz)

Structure	A	B	C
PM7-Pyr-Bz.3-69-19695	496.7183513	412.2930906	283.496352
C_s	493.0630719	411.4518634	282.4105460
Pyr-Bz-63-15155	1149.9698622	201.1956783	194.7126945
Pyr-Bz.2-62-18973	1162.6027526	200.3715266	194.2096786
Pyr-Bz.3-108-20657	1104.5498299	202.9468203	195.2928824
Pyr-Bz-0-16021	581.1288608	405.8278456	306.0532051
Pyr-Bz-74-20153	1173.4681101	196.6386947	191.2891737

Principal Axis Dipole Moments (Debye)

Structure	X	Y	Z	Overall
PM7-Pyr-Bz.3-69-19695	-0.3386971	-1.9940882	0.2987573	2.0445927
C_s	0.2930230	2.0444287	-0.0000000	2.0653211
Pyr-Bz-63-15155	-2.6937892	0.4729127	-0.1706232	2.7403027
Pyr-Bz.2-62-18973	2.7121504	0.425478	-0.1127408	2.7476357
Pyr-Bz.3-108-20657	2.6438645	0.8399599	-0.0434635	2.7744263
Pyr-Bz-0-16021	0.1277025	-1.6923533	0.0036683	1.6971685
Pyr-Bz-74-20153	2.7841397	0.2370842	-0.1979589	2.8012194

4.3 $(Py)_2$-Bz

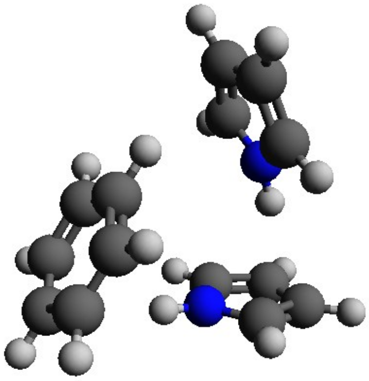

PM7-Pyr-Bz.1-0-15773

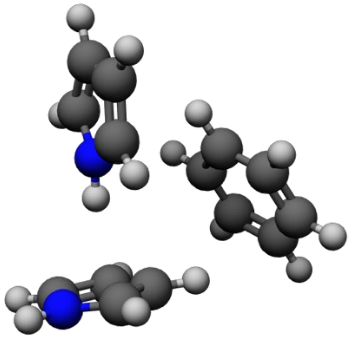

PM7-Pyr-Bz-160-17405

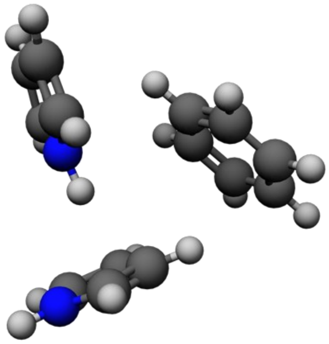

PM7-Pyr-Bz.3-109-13617

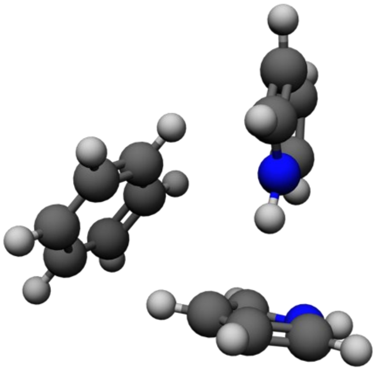

PM7-Pyr-Bz.1-191-13673

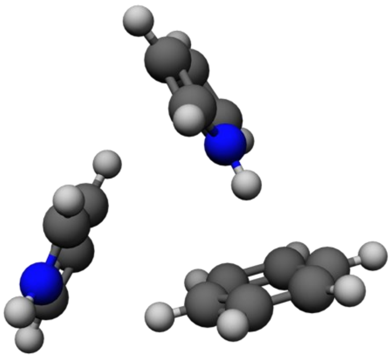

PM7-Pyr-Bz.1-258-17041

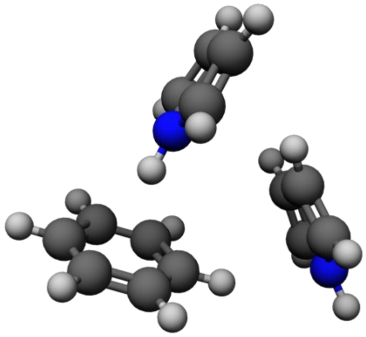

PM7-Pyr-Bz.1-150-16914

Figure S6. The six lowest-energy isomers of $(Py)_2$-Bz.

Table S49. Calculated energies, rotational constants, and principal axis dipole moments of $(Py)_2$-Bz. Electronic energies, Gibbs free energies, and Boltzmann populations at 100 K are relative to the global-minimum isomer sampled.

Energetics

Structure	ΔE_{el} (kcal/mol)	ΔG 100 K (kcal/mol)	Relative Boltzmann Population 100 K
PM7-Pyr-Bz.1-0-15773	0.00	0.00	100 %
PM7-Pyr-Bz-160-17405	4.23	3.89	0.00%
PM7-Pyr-Bz.3-109-13617	4.28	4.06	0.00%
PM7-Pyr-Bz.1-191-13673	4.40	4.09	0.00%
PM7-Pyr-Bz.1-258-17041	5.33	4.65	0.00%
PM7-Pyr-Bz.1-150-16914	5.35	4.91	0.00%

Rotational Constants (MHz)

Structure	A	B	C
PM7-Pyr-Bz.1-0-15773	577.457118	473.206354	326.64178
C_s transition state	577.4082969	472.6061116	326.4563247
PM7-Pyr-Bz-160-17405	538.6366418	464.3564962	308.5767659
PM7-Pyr-Bz.3-109-13617	531.7104852	465.5319918	306.8864725
PM7-Pyr-Bz.1-191-13673	545.6305327	459.7644807	309.4761167
PM7-Pyr-Bz.1-258-17041	532.4735779	472.2783313	310.241531
PM7-Pyr-Bz.1-150-16914	527.8183418	469.2129596	307.8204123

Principal Axis Dipole Moments (Debye)

Structure	X	Y	Z	Overall
PM7-Pyr-Bz.1-0-15773	2.212015	0.5609957	0.0363499	2.2823339
C_s transition state	-2.2199724	0.5424701	-0.0000820	2.2852902
PM7-Pyr-Bz-160-17405	-1.0692385	2.8576227	-0.3590433	3.0721638
PM7-Pyr-Bz.3-109-13617	-0.9733874	-2.9733279	0.6243668	3.1902971
PM7-Pyr-Bz.1-191-13673	-0.658665	2.4813851	-0.8484966	2.7038969
PM7-Pyr-Bz.1-258-17041	0.1593654	-2.8281461	1.6363653	3.2713146
PM7-Pyr-Bz.1-150-16914	0.2475196	-3.3766611	-0.9893573	3.5273126

4.4 $(Py)_2$-$(Bz)_2$

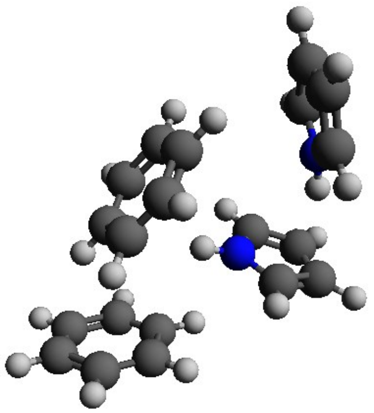

PM7-Pyr-Bz.1-417-393

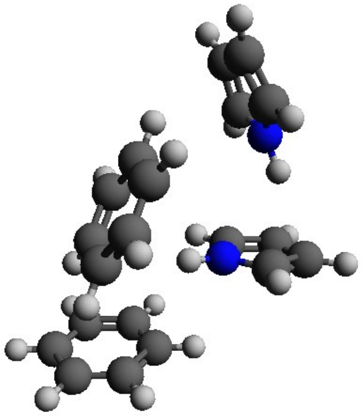

PM7-Pyr-Bz.2-293-18338

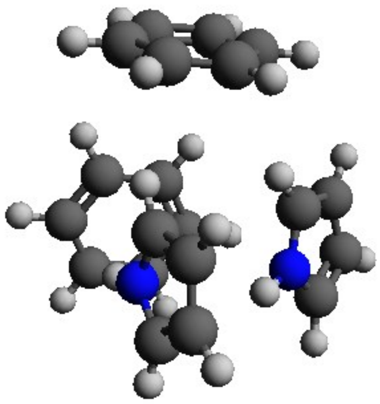

PM7-Pyr-Bz.1-164-20724

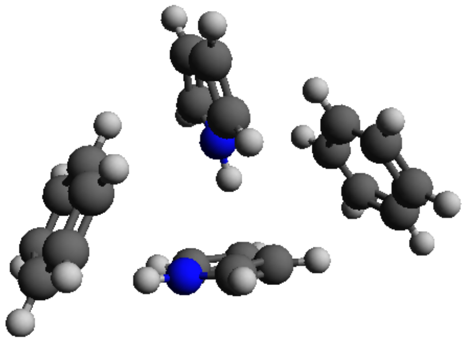

PM7-Pyr-Bz.1-272-20075

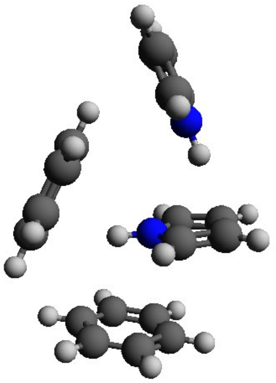

PM7-Pyr-Bz-169-20873

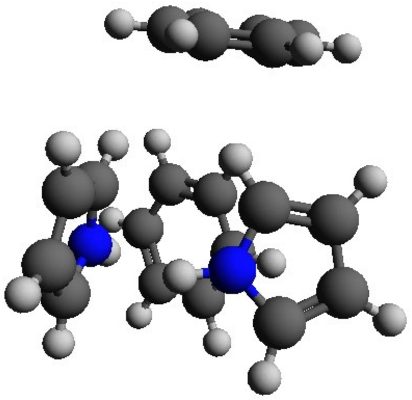

PM7-Pyr-Bz.1-103-17093

Figure S7. The isomers of $(Py)_2$-$(Bz)_2$ with relative Boltzmann populations above 0.5%.

Table S50. Calculated energies, rotational constants, and principal axis dipole moments of $(Py)_2$-$(Bz)_2$. Electronic energies, Gibbs free energies, and Boltzmann populations at 100 K are relative to the global-minimum isomer sampled.

Energetics

Structure	ΔE_{el} (kcal/mol)	ΔG 100 K (kcal/mol)	Relative Boltzmann Population 100 K
PM7-Pyr-Bz.1-417-393	0.00	0.00	100 %
PM7-Pyr-Bz.2-293-18338	0.31	0.34	17.70%
PM7-Pyr-Bz.1-164-20724	0.61	0.51	7.85%
PM7-Pyr-Bz.1-272-20075	0.28	0.77	2.09%
PM7-Pyr-Bz-169-20873	0.56	0.83	1.51%
PM7-Pyr-Bz.1-103-17093	0.44	1.04	0.53%

Rotational Constants (MHz)

Structure	A	B	C
PM7-Pyr-Bz.1-417-393	480.806752	174.827378	150.012111
C_s transition state	477.8151555	176.6482855	151.0691317
PM7-Pyr-Bz.2-293-18338	446.604427	176.982941	154.427719
PM7-Pyr-Bz.1-164-20724	295.781431	232.603778	212.687133
PM7-Pyr-Bz.1-272-20075	466.526411	178.395869	151.414996
PM7-Pyr-Bz-169-20873	439.80441	199.602571	163.176593
PM7-Pyr-Bz.1-103-17093	303.421896	227.481354	207.061966

Principal Axis Dipole Moments (Debye)

Structure	X	Y	Z	Overall
PM7-Pyr-Bz.1-417-393	-2.4441932	0.2330308	-0.0018779	2.4552774
C_s transition state	-2.3644303	0.3198629	0.0000000	2.3859679
PM7-Pyr-Bz.2-293-18338	2.3667563	-0.290705	0.8481113	2.5308768
PM7-Pyr-Bz.1-164-20724	-0.0273392	-2.0350821	0.6488282	2.1361846
PM7-Pyr-Bz.1-272-20075	-1.7170384	1.4461052	-0.1779378	2.251911
PM7-Pyr-Bz-169-20873	-2.2849702	0.7147729	0.2274342	2.4049357
PM7-Pyr-Bz.1-103-17093	0.2018416	2.1004193	0.0630476	2.1110368

4.5 Py-$(Bz)_3$

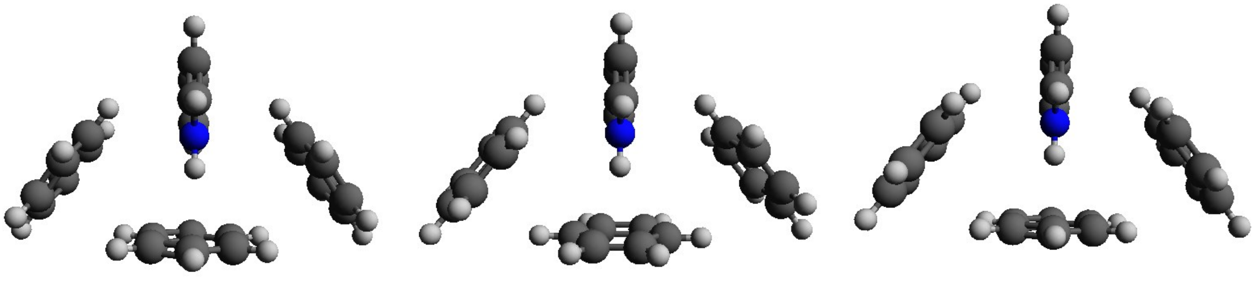

PM7-Pyr-Bz.2-278-11301 PM7-Pyr-Bz-104-20560 PM7-Pyr-Bz.1-106-20903

Figure S8. The isomers of Py-$(Bz)_3$ with relative Boltzmann populations above 0.5%.

Table S51. Calculated energies, rotational constants, and principal axis dipole moments of Py-$(Bz)_3$. Electronic energies, Gibbs free energies, and Boltzmann populations at 100 K are relative to the global-minimum isomer sampled.

Energetics

Structure	ΔE_{el} (kcal/mol)	ΔG 100 K (kcal/mol)	Relative Boltzmann Population 100 K
PM7-Pyr-Bz.2-278-11301	0.40	0.00	100 %
PM7-Pyr-Bz-104-20560	0.00	0.32	19.61%
PM7-Pyr-Bz.1-106-20903	0.48	0.46	9.91%

Rotational Constants (MHz)

Structure	A	B	C
PM7-Pyr-Bz.2-278-11301	419.15592	164.682805	141.521347
PM7-Pyr-Bz-104-20560	439.772693	160.896846	138.275108
PM7-Pyr-Bz.1-106-20903	415.602688	165.661096	143.232845

Principal Axis Dipole Moments (Debye)

Structure	X	Y	Z	Overall
PM7-Pyr-Bz.2-278-11301	0.0342094	1.6727291	-0.1798952	1.6827226
PM7-Pyr-Bz-104-20560	0.0340021	1.6593042	0.2134192	1.6733183
PM7-Pyr-Bz.1-106-20903	-0.0001217	1.7208238	-0.2399238	1.7374689

4.6 $(Py)_3$-Bz

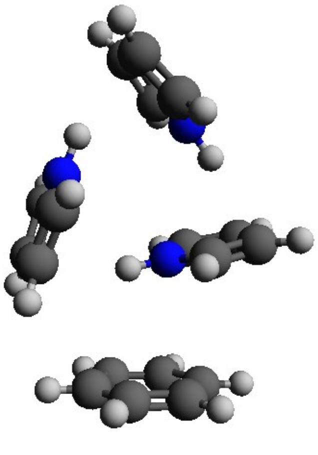

PM7-Pyr-Bz.1-11-20232

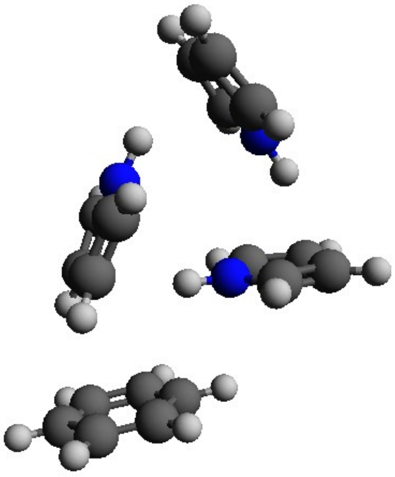

PM7-Pyr-Bz.1-290-18715

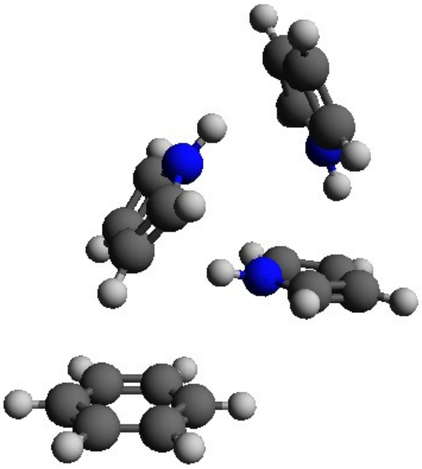

PM7-Pyr-Bz.1-345-16021

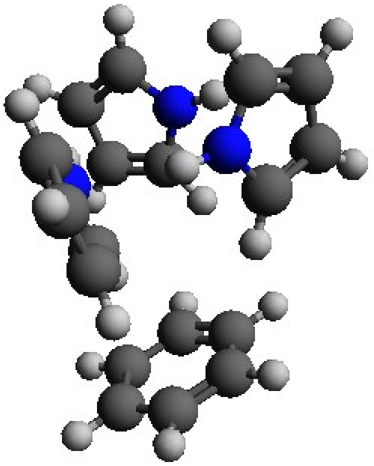

Pyr-Bz.2-682-284

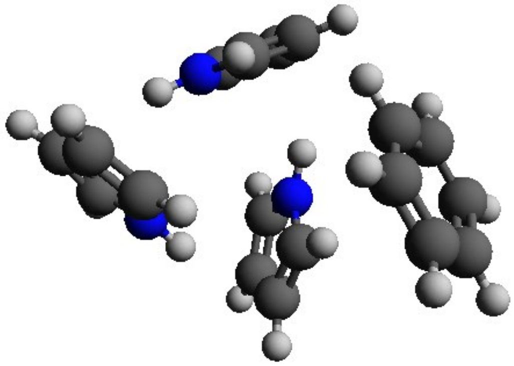

PM7-Pyr-Bz.2-250-18789

Figure S9. The isomers of $(Py)_3$-Bz with relative Boltzmann populations above 0.5%.

Table S52. Calculated energies, rotational constants, and principal axis dipole moments of $(Py)_3$-Bz. Electronic energies, Gibbs free energies, and Boltzmann populations at 100 K are relative to the global-minimum isomer sampled.

**Energetics**				
Structure	ΔE_{el} (kcal/mol)	ΔG 100 K (kcal/mol)	Relative Boltzmann Population 100 K	
PM7-Pyr-Bz.1-11-20232	0.00	0.00	100 %	
PM7-Pyr-Bz.1-290-18715	0.18	0.05	78.01%	
PM7-Pyr-Bz.1-345-16021	0.19	0.15	47.05%	
Pyr-Bz.2-682-284	0.08	0.59	5.03%	
PM7-Pyr-Bz.2-250-18789	0.64	0.94	0.88%	
**Rotational Constants (MHz)**				
Structure	A	B	C	
PM7-Pyr-Bz.1-11-20232	503.957018	216.661438	178.603417	
PM7-Pyr-Bz.1-290-18715	512.795522	194.337208	164.887787	
PM7-Pyr-Bz.1-345-16021	512.373717	192.056835	163.638884	
Pyr-Bz.2-682-284	356.882606	236.388277	227.626539	
PM7-Pyr-Bz.2-250-18789	394.0908	216.591327	199.753953	
**Principal Axis Dipole Moments (Debye)**				
Structure	X	Y	Z	Overall
PM7-Pyr-Bz.1-11-20232	0.2392528	-0.2615937	0.0000763	0.3545041
PM7-Pyr-Bz.1-290-18715	-0.2059625	-0.0529555	-0.0565913	0.2200623
PM7-Pyr-Bz.1-345-16021	-0.2581807	-0.055363	0.0488537	0.2685312
Pyr-Bz.2-682-284	-0.0225197	-0.0413167	0.3623169	0.3653597
PM7-Pyr-Bz.2-250-18789	-0.5327378	-0.2188086	0.0266368	0.5765382

4.7 Many-Body Expansion

Table S53. Non-additive two- (E_{2B}), three- (E_{3B}), and four-body (E_{4B}) interaction energies computed with DLPNO-CCSD(T)/haug-cc-pVQZ, in kJ/mol.

	E_{2B}	E_{3B}	E_{4B}
Py-$(Bz)_2$	-52.671	-2.357	n/a
$(Py)_2$-Bz	-67.448	-4.461	n/a
$(Py)_2$-$(Bz)_2$	-93.768	-3.653	0.380
