# Supplementary material for: Space filling shapes the interaction networks in mixed pyrrole-benzene trimers and tetramers
Source: Commun Chem. 2026 Apr 17;9:213. doi: 10.1038/s42004-026-02027-1 (PMC13272942; doi:10.1038/s42004-026-02027-1)
Supplement: Supplementary file 2 — Description of Additional Supplementary Files [file 42004_2026_2027_MOESM2_ESM.docx]

**Description of Additional Supplementary Files:**

**File:** Supplementary Data 1

**Description: Theoretical coordinates of all the clusters from quantum-chemical calculations at the ωB97X-D functional and the 6 -31 + +G basis set.**
